# Direct synthesis of ethers from alcohols & aldehydes enabled by an oxocarbenium ion interception strategy†

**DOI:** 10.1039/d4sc06203e

**Published:** 2025-03-04

**Authors:** Dara T. Curran, Marcin Szydło, Helge Müller-Bunz, Kirill Nikitin, Peter A. Byrne

**Affiliations:** a Centre for Synthesis & Chemical Biology, School of Chemistry, University College Dublin Belfield Dublin 4 Ireland peter.byrne@ucd.ie kirill.nikitin@ucd.ie; b SSPC, the Research Ireland Centre for Pharmaceuticals Ireland

## Abstract

A new method has been established for formation of ethers from aldehydes and alcohols – a net reductive etherification. Reactions of these entities with phosphines in the presence of acid enable formation of α-(alkoxyalkyl)phosphonium salts, which, upon hydrolysis, result in formation of ether products in isolated yields of 63–92%. Formation and hydrolysis of the α-(alkoxyalkyl)phosphonium salts were done in an efficient telescoped two-step, one-pot process that does not require inert atmosphere conditions. Formation of the key phosphonium salt intermediates was found to occur in preference to acetal formation and is proposed based on both experimental and computational evidence to involve interception of oxocarbenium ions formed by reaction of the aldehyde, alcohol and acid by phosphine. This method represents the first instance in which net reductive etherifications have been achieved without the requirement for use of hydrides or hydrogen as reductants, and exhibits excellent functional group tolerance, thus enabling facile hydride-free synthesis of ethers. These are amongst the most important functional groups in organic synthesis. The new etherification method also enables deuteride-free synthesis of deuterated ethers.

## Abstract 2

While a combination of alcohol and aldehyde under acidic conditions typically gives an acetal, interception of the intermediate oxocarbenium ion by a phosphine followed by hydrolysis leads to ethers without the need for a hydride donor.

## Graphical abstract

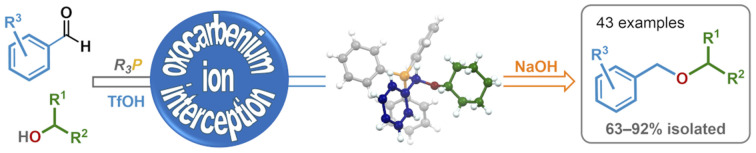

## Introduction

Ethers are among the most ubiquitous of pharmacophores in pharmaceutical molecules^1^ and, consequently, establishing sustainable routes to their syntheses is of the utmost importance. Of the various classical methods for synthesising dialkyl or alkyl aryl ethers,^2^ Williamson etherifications are by far the most widely used for ether formation in industrial pharmaceutical synthetic processes. In general, these are known to have poor functional group tolerance, and hence it has often been necessary to conduct ether syntheses in the early stages of a synthetic route to a drug.^3^

**Scheme 1:**
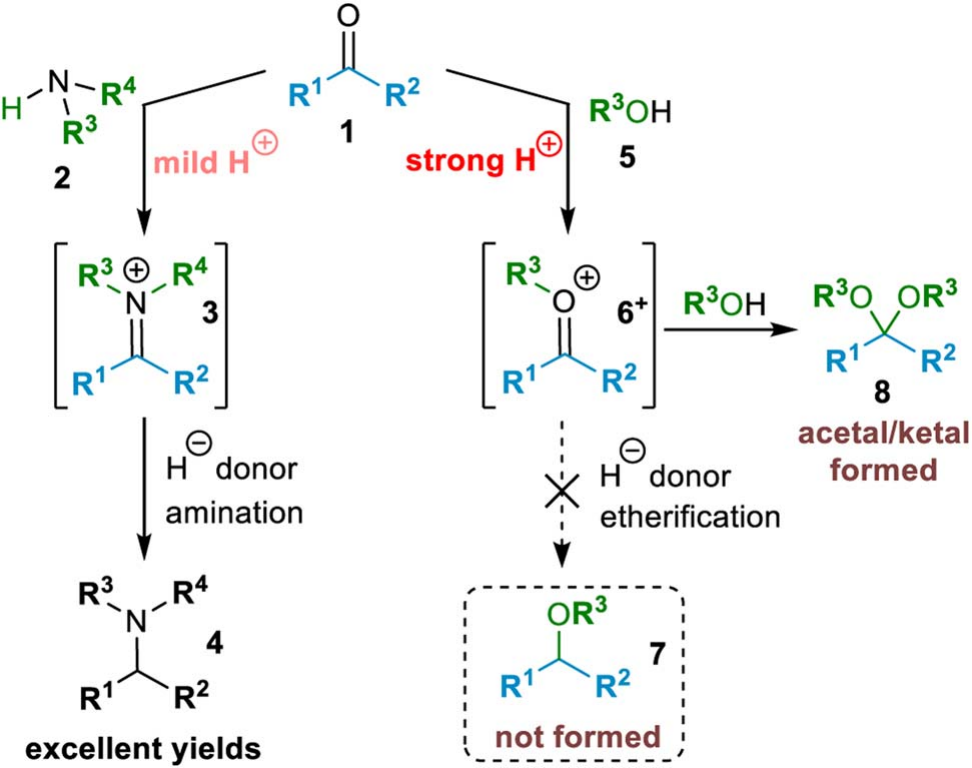
Diverse behaviour of carbonyl compounds (1): stable iminium ion 3 leads to amination but transient oxocarbenium ion 6^+^ gives acetal 8.

By comparison, for amines, the strategy of reductive amination of carbonyl compounds (1) is a key enabling process in organonitrogen chemistry to achieve controlled net alkylation (*e.g.*, selective formation of tertiary amines (4) from secondary amines (2); see Scheme 1).^4^ It is fundamentally efficient and simple as it involves formation of an imine or iminium ion (3; see Scheme 1), followed by facile reduction of the C=N bond. By analogy with the reductive amination strategy, combination of an alcohol (5) with a carbonyl compound (1) to yield an *O*-alkyloxocarbenium ion (6^+^) (see Scheme 1 for compound structures) followed by formal hydride reduction of the cationic [C=O–R^3^]^+^ moiety appears to afford a very direct means of forming ethers (7).^5,6^ This is attractive because both alcohols and aldehydes are naturally abundant,^7^ can be sustainably derived from biomass,^7,8^ and are generally readily available.^8,9^ However, as a consequence of the higher electronegativity of O, alcohols are far less reactive than amines as nucleophiles, and hence formation of oxocarbenium ion 6^+^ from alcohol 5 and carbonyl compound 1 requires the presence of a strong acid to catalyse and drive the reaction, and even then only miniscule concentrations of 6^+^ are present at one time in such reactions. Consequently, efficient *in situ* reduction of *O*-alkyloxocarbenium ions (6^+^) cannot easily be achieved with common hydride donor reducing agents because these reagents would be quenched in the presence of strong acid. Hence, in practice, formation of an acetal or ketal (8) is observed in reactions of this type Scheme 1.^10^

**Scheme 2:**
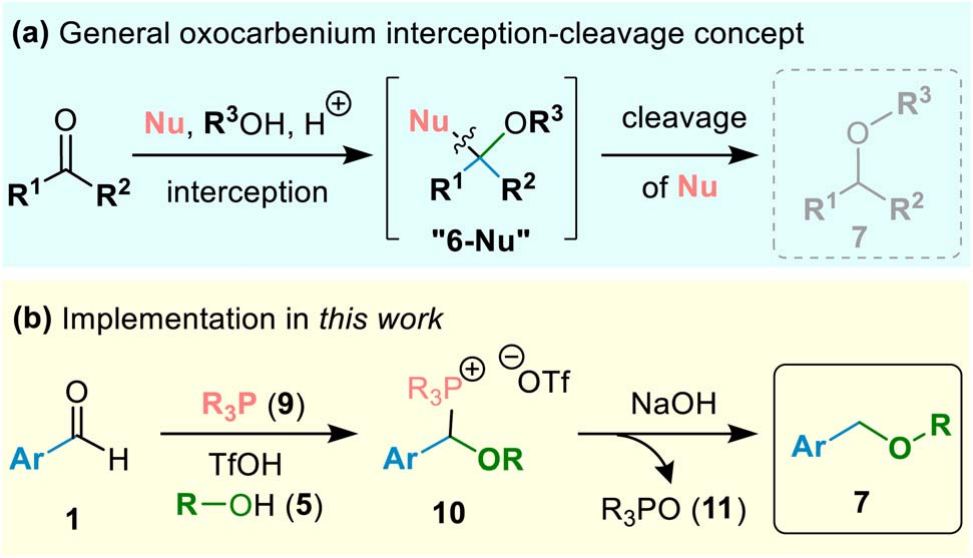
Novel strategy: (a) sacrificial nucleophile, Nu, enables oxocarbenium ion interception to generate stable intermediate, 6-Nu, followed by release of desired ether 7; (b) phosphine-mediated one-pot hydrolytic etherification of aromatic aldehydes reported herein.

Recognising the above limitation, we decided to pursue an entirely different strategy for net reductive etherification (Scheme 2a) involving, first, capture of the oxocarbenium ion with a suitable sacrificial nucleophile Nu to form an intermediate [6-Nu] and, second, release of the desired ether (7) upon controlled decomposition of [6-Nu], without the need to employ a hydride reducing agent. We considered that a weakly-basic nucleophile such as a phosphine (R_3_P; 9)^11^ could efficiently compete with alcohol 5 to react with *O*-alkyloxocarbenium ion even under acidic conditions to form a relatively stable phosphonium species, 10,^12^ which, upon hydrolytic work-up, could give ethers (7) by selective cleavage of the *P-*(α-alkoxyalkyl) group from phosphorus, as shown in Scheme 2b (see further details on phosphonium salt hydrolysis below).^13^ Thus, we decided to investigate the formation and hydrolysis of (α-alkoxyalkyl)phosphonium salts (10), and, as will be described below, ultimately developed a direct one-pot method for formation of ethers (7) from alcohols (5) and aldehydes (1; Scheme 2b).

**Scheme 3:**
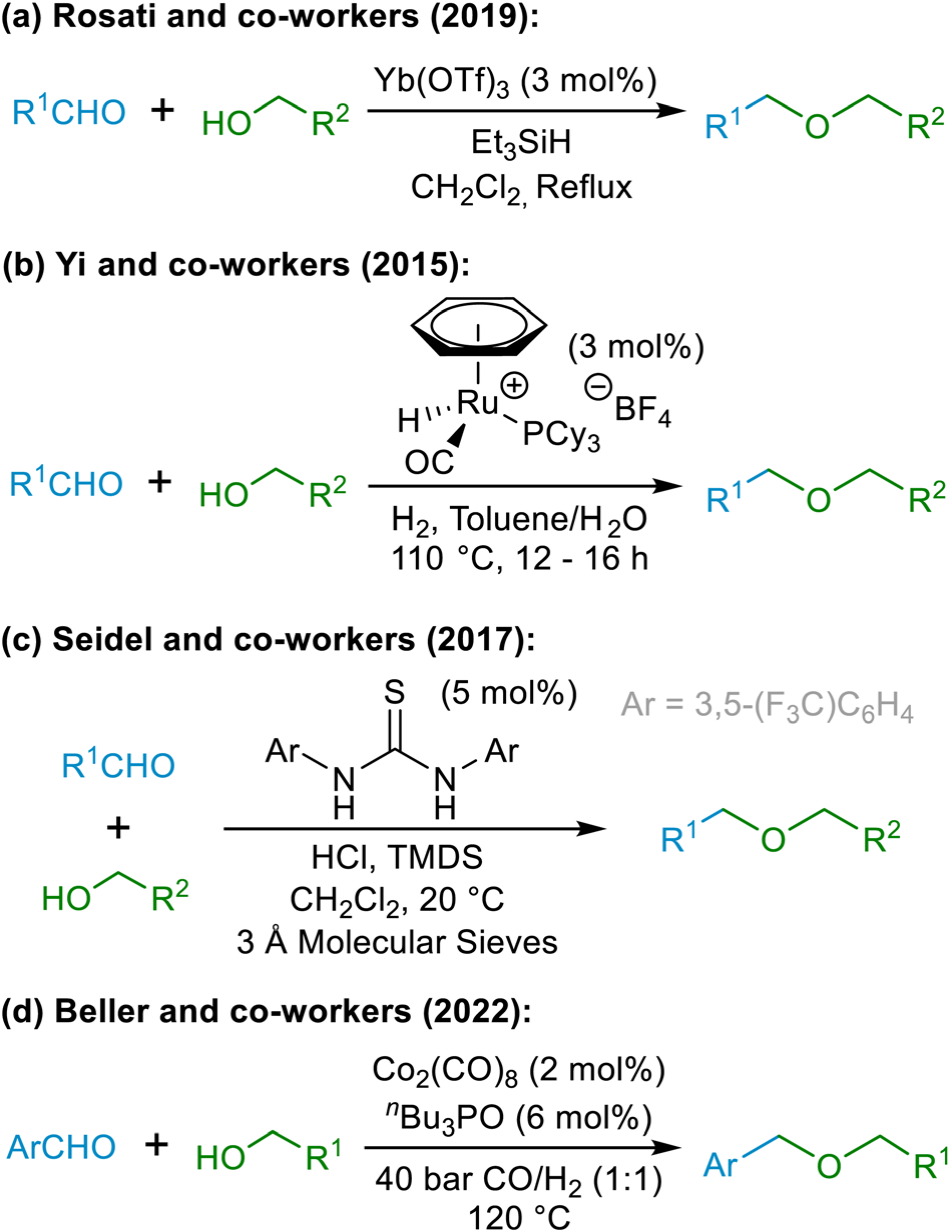
Selected examples of recent reductive etherification strategies.

Although direct reductive etherification cannot be achieved in the same manner that reductive amination can, alternative approaches have been developed to achieve net reductive etherification using alcohols (5) and carbonyl compounds (1) as the starting materials, as shown in the examples in Scheme 3.^14^ All of the existing reductive approaches for synthesising ethers require the use of hydride reagents or H_2_ gas in combination with a catalyst, as exemplified by the representatives shown in Scheme 3. The new hydride-free etherification methodology described herein, based around our novel oxocarbenium ion interception strategy, is thus completely distinct from the existing methods. Due to the unique manner in which ether formation is achieved in this chemistry, the proposed method has the capacity to become a highly used complementary tool to existing reductive etherifications^14,15^ and to standard etherification and protection approaches,^16–22^ in particular as it exhibits excellent functional group tolerance that is distinct from other etherification methods – for example, tolerating the presence of alkenes, alkynes, nitriles, ketones, amides, carboxylic acids, and aryl halides that may be unstable to the conditions employed in reductive etherification methodologies involving transition metal catalysis and H_2_ gas as the reductant (*vide infra*).

## Results and discussion

### Optimisation of (α-alkoxyalkyl)phosphonium salt formation

**Scheme 4:**
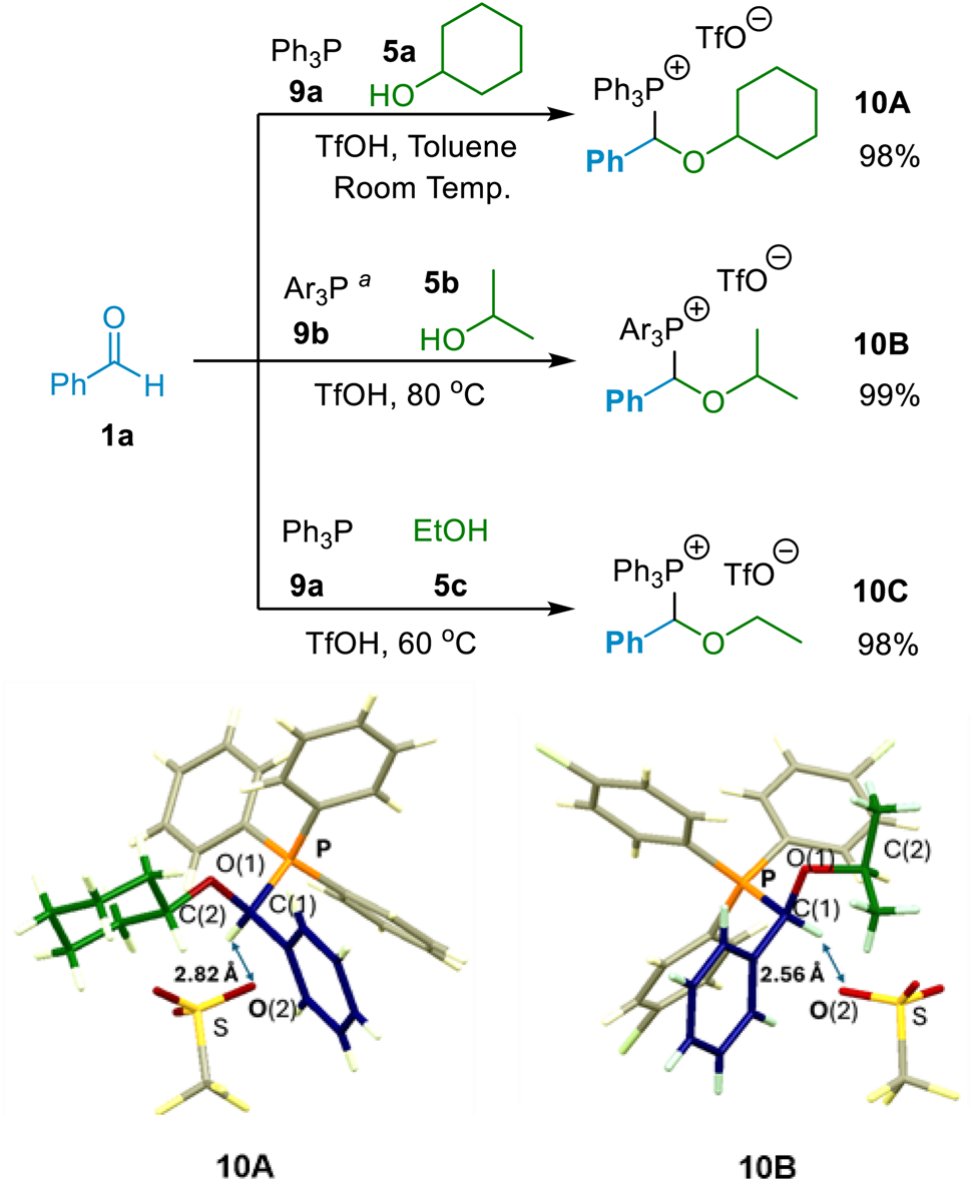
Direct formation of (α-alkoxyalkyl)phosphonium salts 10A–10C. X-ray crystal structures of 10A and 10B are also shown. In each of these, the aromatic aldehyde core fragment (blue) and aliphatic alcohol fragment (green) are linked by an oxygen O(1), red, to form an ether-like moiety which is released upon hydrolytic cleavage of the P–C(1) bond.^*a*^ Ar = *p*-ClC_6_H_4_ (*p*-chlorophenyl).

Our initial investigations in this project were focused on determining whether (α-alkoxyalkyl)phosphonium salts (10) could be formed in reactions involving an aldehyde (1), a phosphine (9) and an alcohol (5) under acidic conditions. The experiments undertaken showed that (α-alkoxyalkyl)phosphonium salts were indeed formed in high yields in such reactions, thereby demonstrating that phosphines do possess the capacity to function to capture transiently formed oxocarbenium ions in the manner outlined in Scheme 2 above, thereby directing the reaction away from acetal formation.^12*h*–*k*^ Reaction of aldehyde 1a + Ph_3_P (9a) + cyclohexanol (5a) in toluene solvent at room temperature, and in the presence of TfOH, led to a 98% isolated yield of crystalline (α-alkoxyalkyl)phosphonium salt 10A (Scheme 4). The X-ray crystal structure of this entity in Scheme 4 clearly shows the connectivity within the α-alkoxyalkyl moiety that gets released as the ether product upon hydrolysis of 10A. The other characterisation data recorded for this compound is also consistent with the structure of 10A. Similarly, treatment of tris(*p*-chlorophenyl)phosphine (9b) with 1a in isopropanol (5b) under similar conditions led to quantitative formation of (α-alkoxyalkyl)phosphonium triflate salt 10B (Scheme 4). The structure of this was again verified spectroscopically, and an X-ray crystal structure of 10B is shown in Scheme 4. Treatment of aldehyde 1a and phosphine 9a with TfOH in ethanol (5c) also led to quantitative formation of (α-ethoxybenzyl)phosphonium salt 10C (Scheme 4). Crystalline material was not obtained in this instance, but the ^31^P, ^1^H and ^13^C NMR spectral data recorded of the isolated product were consistent with the structure of 10C.

**Scheme 5:**
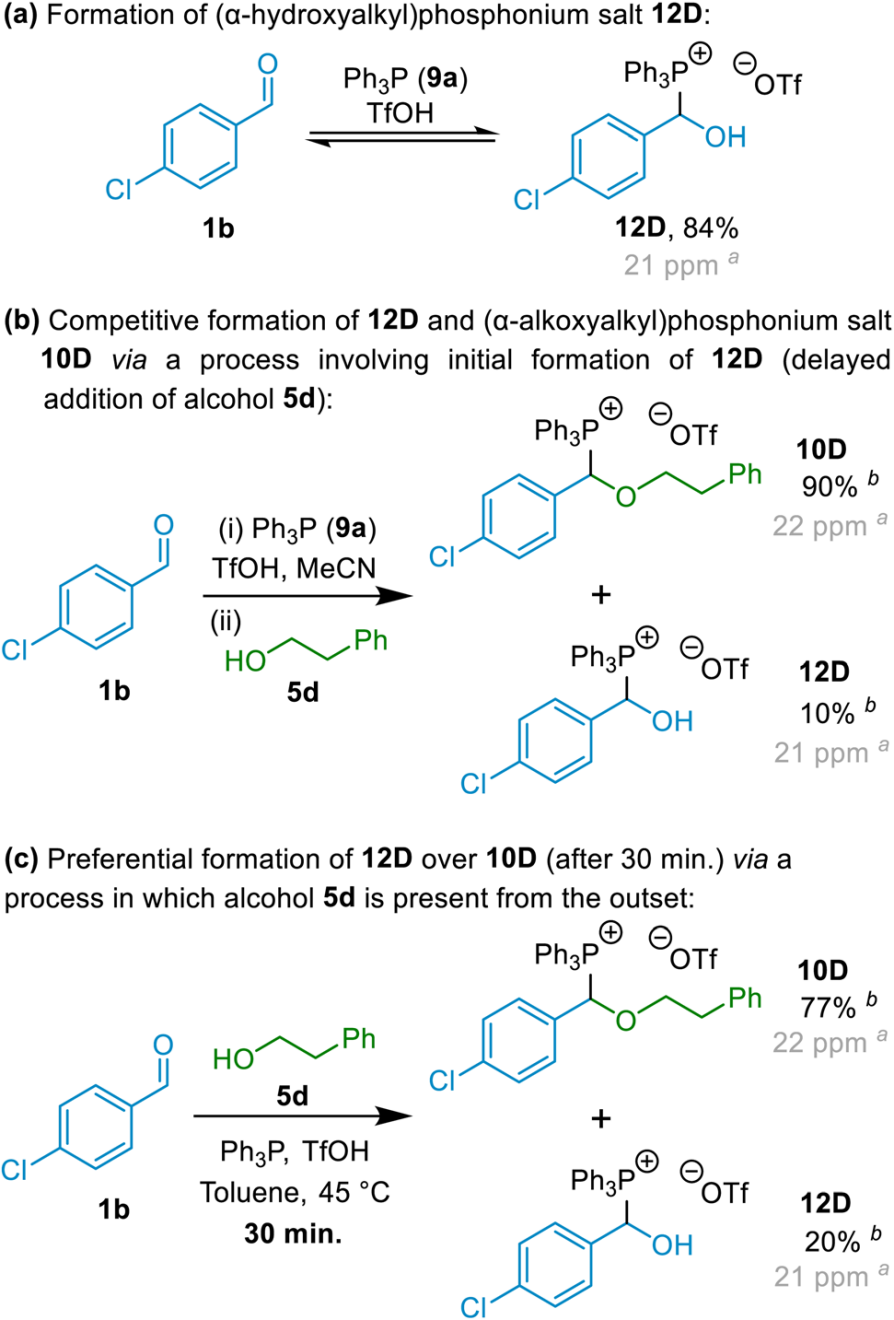
Model reactions employed for optimisation of (α-alkoxyalkyl)phosphonium salt formation. ^*a*^Minor variations in the *δ*_P_ values for these compounds were observed between experiments;^26^^*b*31^P NMR spectral yield; the mass balance in this experiment was accounted for by [Ph_3_PH]OTf.

Having established that (α-alkoxyalkyl)phosphonium salt formation was possible (in principle by interception of an oxocarbenium ion), and indeed apparently highly favourable, our next focus was to establish a general set of reaction conditions that could be applied to produce high yields of (α-alkoxyalkyl)phosphonium salts derived from a wide range of alcohol and aldehyde starting materials. We elected to employ aromatic aldehyde 1b, primary alcohol 5d and phosphine 9a in our optimisation experiments (with acid also being present), and thus aimed for formation of (α-alkoxyalkyl)phosphonium salt 10D (Scheme 5), and conducted a series of experiments involving variation of reaction solvent, temperature and time, as well as variation in the reaction mixture concentration and in the relative amounts of each of the reagents employed.^23^

In the absence of alcohol, mixing of aldehyde 1b, phosphine 9a and TfOH at 20 °C resulted in the formation of (α-hydroxyalkyl)phosphonium salt 12D in 84% NMR spectral yield after 4 hours (see Scheme 5a; *δ*_P_ = *ca.* 21 ppm).^24^ This was entirely consistent with the observations of Masarwa and co-workers^25^ for reactions of phosphines with aldehydes in the presence of acid, which also resulted in formation of a species that was identified as an (α-hydroxyalkyl)phosphonium salt (*δ*_P_ = *ca.* 23 ppm) by those authors.^25^ Upon addition of alcohol 5d to the above reaction mixture, (α-alkoxyalkyl)phosphonium 10D became the major species present (NMR spectral yield of 90% recorded after 24 hours – see Scheme 5b; *δ*_P_ = *ca.* 22 ppm for 10D), with only a small quantity of (α-hydroxyalkyl)phosphonium salt 12D remaining. However, in experiments in which alcohol 5d was added at the outset along with the aldehyde, Ph_3_P and TfOH, (α-alkoxyalkyl)phosphonium salt 10D was observed to be the major species present in the reaction mixture even at the early stages of the reaction, with only minor quantities of (α-hydroxyalkyl)phosphonium salt 12D also being present (see Scheme 5c; similar results were also obtained using MeCN solvent).^27^ This indicates that 12D need not necessarily be an intermediate on the pathway to 10D. Indeed, for reasons discussed below (see details on mechanistic investigations), we consider it highly unlikely that formation of (α-alkoxyalkyl)phosphonium salts such as 10D occur *via* processes in which (α-hydroxyalkyl)phosphonium salts (12) are involved as intermediates; instead, (α-alkoxyalkyl)phosphonium salts and (α-hydroxyalkyl)phosphonium salts form in competition with each other, with (α-hydroxyalkyl)phosphonium salt formation being reversible, and formation of (α-alkoxyalkyl)phosphonium salts generally predominating.

Formation of 10D was found to be effective in a variety of solvents,^28^ but in all cases, a reasonable excess of alcohol 5d was required for high yields of (α-alkoxyalkyl)phosphonium salt to be achieved (1.8 to 2 equivalents relative to aldehyde).^23^ Ultimately, we settled on conducting our reactions in MeCN (for reasons that will be detailed below) at 45 °C using a moderate excess of the alcohol reactant. This allowed us to synthesise various (α-alkoxyalkyl)phosphonium salts and hence ethers in high yields (*vide infra*). In addition, in our optimisation studies, we also observed that good, albeit lower, conversion to 10D can be achieved using 1.2 equivalents of alcohol (77% ^31^P NMR spectral yield), or if 1.0 equivalent of alcohol and 1.5 equivalents of aldehyde are used, a 75% ^31^P NMR spectral yield can be obtained.^23^

### Optimisation of (α-alkoxyalkyl)phosphonium salt hydrolysis

Next, we focused our attention on development of reaction conditions for ether formation by *in situ* hydrolysis of (α-alkoxyalkyl)phosphonium salt 10 (without isolation of the phosphonium salt) that had been formed using the approach described above. Phosphonium salt hydrolysis is a well-established process, but has only been studied in detail for phosphonium salts containing simple *P*-alkyl and *P*-aryl groups.^13^ Hydrolytic cleavage of a *P*-(alkoxyalkyl) group from a phosphonium salt appears never to have been reported or attempted. However, it was anticipated that the *P*-(alkoxybenzyl) group of the (α-alkoxyalkyl)phosphonium salts synthesised in this project would be cleaved from phosphorus selectively due to the preference typically exhibited in phosphonium salt hydrolyses for cleavage of benzylic P–C bonds (over *P*-aryl or simple *P*-alkyl groups).^13^ For example, we have successfully employed such a *P*-quaternisation-hydrolysis strategy in the preparation of otherwise inaccessible ferrocene-derived structures.^29^ In addition, hydrolysis of phosphonium ylides derived from (α-(alkylthio)alkyl)phosphonium salts was reported by Masarwa and co-workers as we neared completion of the research reported herein, leading to formation of thioethers.^30,31^

**Scheme 6:**
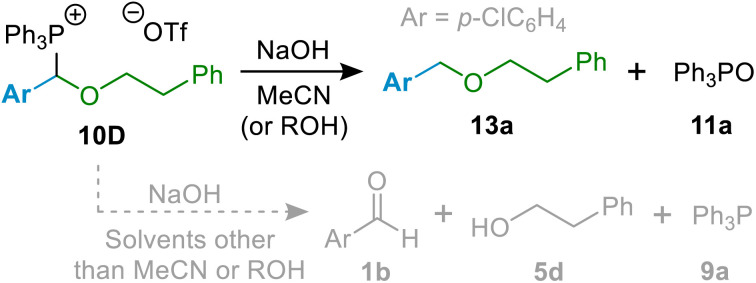
Successful hydrolytic cleavage of 10D provides a model reaction for optimisation of (α-alkoxyalkyl)phosphonium salt hydrolysis.

We observed that under hydrolytic conditions in the maority of solvents tested, (α-alkoxyalkyl)phosphonium salts 10 do furnish the expected ether (*e.g.*, 13a; see Scheme 6), in relatively low amounts, but also undergo a reverse reaction leading to the re-formation of aldehyde 1, phosphine 9 and alcohol 5, *i.e.*, the starting materials form the first step of the reaction. This is consistent with previous observations on hydrolysis of α-alkoxyphosphonium salts (formed by reactions of phosphines with acetals) to form aldehydes.^12*i*^ However, we found that if hydrolysis of 10D (and, subsequently, other salts 10) is carried out in polar MeCN or alcohol as solvent, predominant formation of ether 13a and phosphine oxide 11a is observed, as per the process envisaged in Scheme 2b above.

**Fig. 1:**
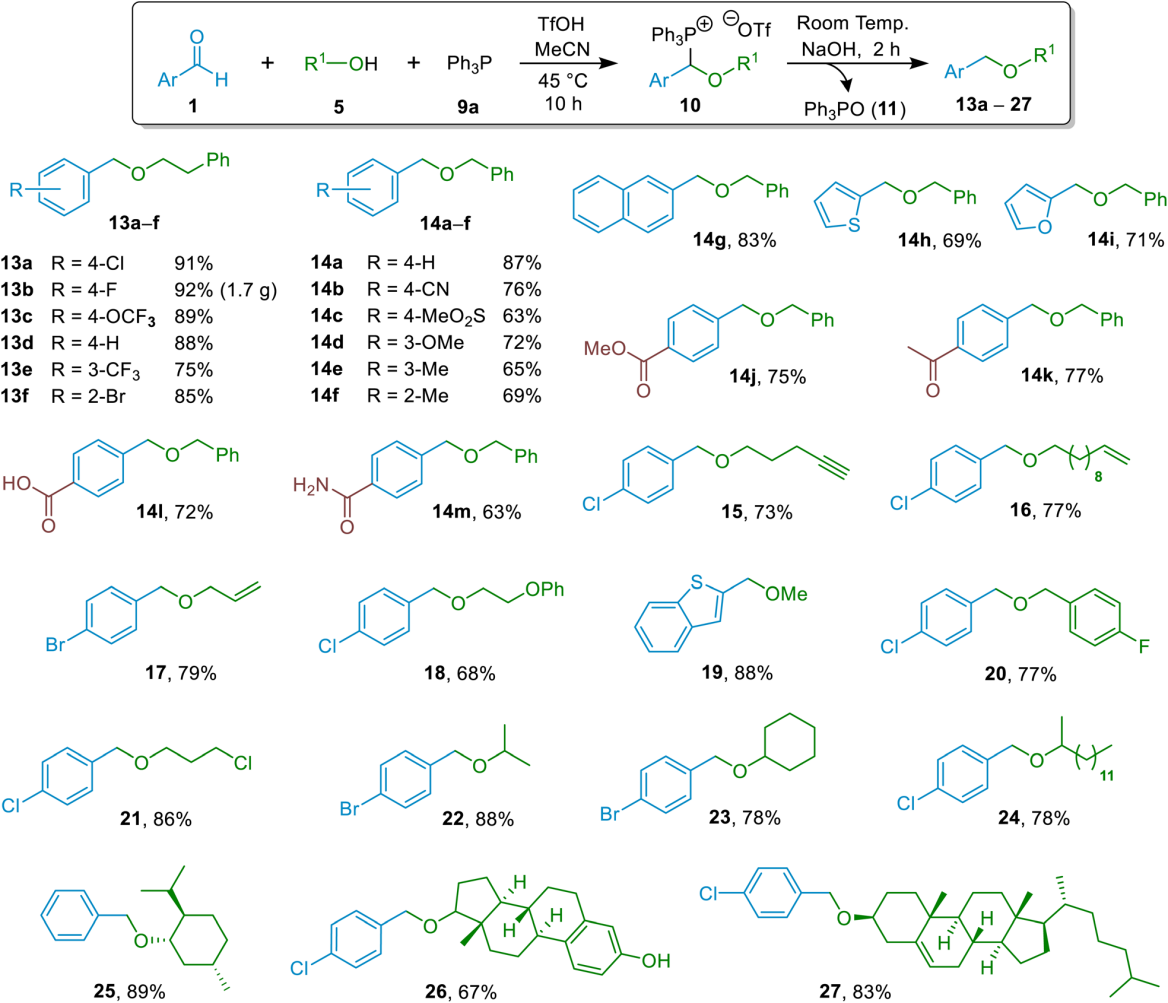
Substrate scope for hydrolytic etherification reactions of aromatic aldehydes and alcohols (isolated yields are shown).

Since MeCN could be used as the solvent both for the (α-alkoxyalkyl)phosphonium salt formation and for the hydrolysis reaction to form the ether product, we ultimately settled on a one-pot process in which the two reactions were telescoped, and applied this for the synthesis of an array of ethers containing a variety of different functional groups (see Fig. 1 below). It was also possible to conduct telescoped one-pot processes using alcohol solvents, but we only applied this approach when the ether derivatives of the alcohol in question were sought.

### Elaboration of substrate scope

Having established a general approach for the construction of ethers through the hydrolytic etherification method discussed above, we undertook an investigation into whether this methodology could be applied successfully across a broad range of alcohol and aldehyde starting materials. For most reactions, the general conditions employed involved using aldehyde (1.0 equivalent), phosphine (1.1 equivalents), TfOH (1.2 equivalents) and alcohol (1.8 equivalents) for 12 h at 45 °C for (α-alkoxyalkyl)phosphonium salt formation, followed by treatment of this salt with NaOH (2.8 equivalents) for 1.5 h at 20 °C. These conditions were employed across the whole range of target ethers 13a–h, 14a–p, and 15–27 (unless stated otherwise) and were not optimised for individual substrates. A notable variation of the general conditions was that use of easily handled tertiary phosphonium salt [Ph_3_PH]OTf as the dual source of TfOH and Ph_3_P (9a)^32^ allowed comparable yields to be obtained (see Section 10 of the ESI†). In addition, it was shown that MsOH could be used in place of TfOH in our etherification reactions, enabling formation of an (α-alkoxyalkyl)phosphonium salt and hence ether 13a in an 89% isolated yield from a reaction involving Ph_3_P, alcohol 5d and aldehyde 1b (compared with 91% using TfOH see Fig. 1) – see details in Section 9 of the ESI.†

The results of our substrate scope elaboration are summarised in Fig. 1. The methodology was applied successfully to synthesis of ethers derived from various aliphatic primary alcohols containing different functional groups in their structures (13a–f, 14a–m, 15–21; Fig. 1), giving isolated yields ranging from 63–92%. The ethers derived from 2-phenylethan-1-ol (13a–f) and methanol (19) were prepared in excellent yields, 75–92%. In most cases, syntheses of ethers derived from benzyl alcohol gave high yields (63–83%; products 14a–m). As the preparation of each individual ether was not optimised, in certain cases (*e.g.*, 14b, 14m), the lower yields observed could arise due to hydrolytic instability of certain functional groups. The methodology was shown to be applicable on gram scale through the synthesis of 13b in a 92% yield. Reactions producing ethers 15–17 derived from alcohols that also contain unsaturated functional groups in their structure (alkenes/alkynes) work quite well (yields 73–79%) despite the possibility of side-reactions involving phosphine quaternisation with these reactive alcohols under the reaction conditions used (*i.e.*, since acid is present). Successful preparation of the ester-containing ether 14j in 75% yield is attributable to selective base hydrolysis of phosphonium moiety of the precursor (α-alkoxyalkyl)phosphonium salt in preference to hydrolysis of the ester group of that species. Another important highlight of the hydrolytic method is that etherification of an aldehyde can be achieved chemoselectively even in the presence of a ketone, as demonstrated in the synthesis of ketone-containing ether 14k. This sets it apart from known reductive etherification protocols, (see Scheme 3 for comparison). Furthermore, the facile etherification of carboxylate-substituted aldehyde leading to ether 14l underscores the synthetically orthogonal nature of phosphorus-assisted hydrolytic etherification. We believe that the predominant unwanted process that competes with ether formation in these reactions (which may thus lead to reductions in yields) in most cases is the base-assisted reversal of the (α-alkoxyalkyl)phosphonium salt intermediates (10) to starting materials (phosphine + aldehyde + alcohol) under the conditions used for (α-alkoxyalkyl)phosphonium salt hydrolysis (Scheme 6), as exemplified in the ESI, Section 11.†

Moving to ether derivatives of secondary alcohols, high yields were obtained in most cases (between 78% and 89% for ethers 22–25 and 27) in perfect agreement with quantitative formation of the (α-alkoxyalkyl)phosphonium salt intermediates (10) that function as precursors to these ethers, as discussed above (Scheme 4). This includes high yielding preparations and isolations of ether derivatives of menthol and cholesterol (25 and 27). Conversely, the methodology was found to be ineffective if phenols or tertiary alcohols were employed as the R–OH reactant.^33^ This lack of reactivity towards phenols, however, was exploited to selectively benzylate the secondary aliphatic alcohol group of estradiol in preference to the phenol hydroxy group, leading to isolation of ether 26 in a 67% yield. Such chemoselectivity could not be achieved with many existing methods for synthesis of ethers; for example, any attempt to benzylate an aliphatic alcohol in the presence of an unprotected phenol using Williamson etherification conditions would lead to competitive benzylation of both hydroxy groups.

**Scheme 7:**
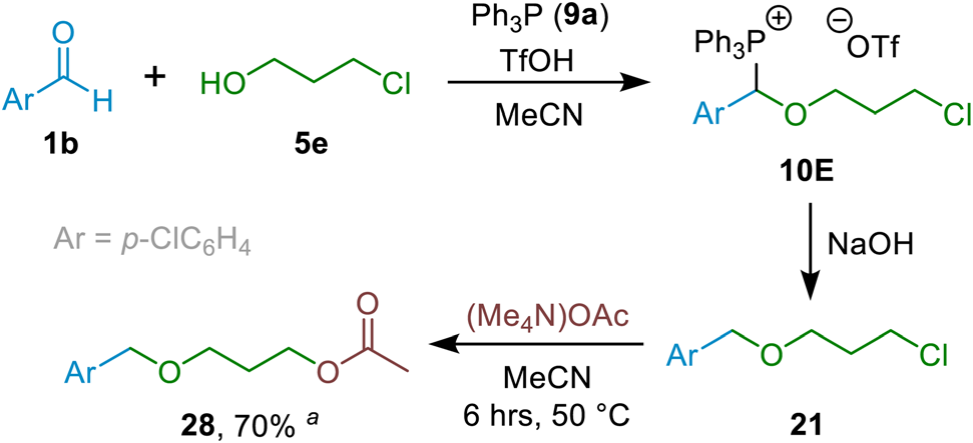
Derivatisation of 21 with (Me_4_N)OAc to give 28, highlighting the ability of the protocol to enable late stage functionalisation.^*a* 1^H NMR spectral yield.

A particularly striking result was the formation of ether 21 starting from aldehyde 1b and alkyl halide-containing alcohol 5e (Scheme 7). The fact that the new hydrolytic etherification methodology is tolerant of alkyl chlorides can be exploited to enable further functionalisation of the product subsequent to the etherification step, as was demonstrated through the formation of ester-containing ether 28 (which would be a major challenge using some existing methodologies) from 21 by nucleophilic substitution of the alkyl chloride by acetate in a 70% NMR spectral yield (Scheme 7).

### Selective incorporation of deuterium into ether products

**Fig. 2:**
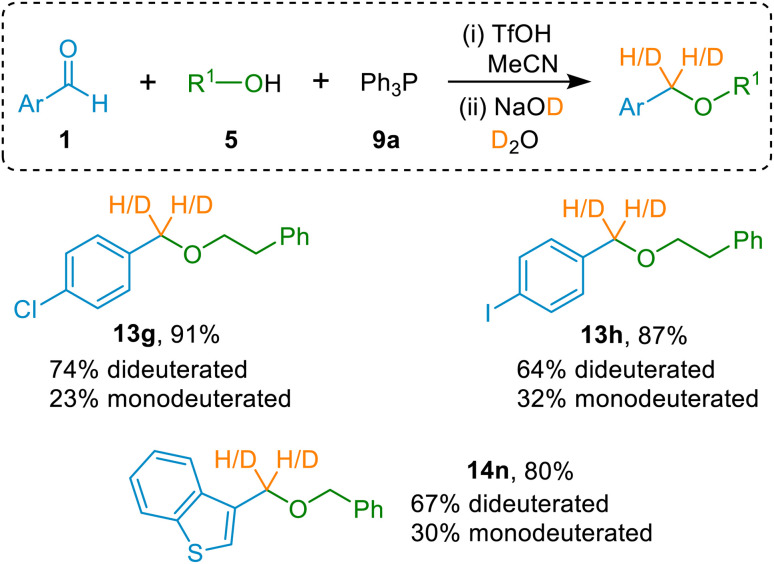
Selectively-deuterated ether products synthesised by using NaOD-mediated deuterolysis of (α-alkoxyalkyl)phosphonium salts as the second step of the etherification protocol. Minor quantities of non-deuterated product (≤4%) were also formed.

Since ether formation in the methodology described above involves simple hydrolysis of (α-alkoxyalkyl)phosphonium salts using aqueous NaOH, we identified that if a NaOD solution in D_2_O were used in place of aqueous NaOH, the possibility existed to effect (α-alkoxyalkyl)phosphonium salt deuterolysis and thus achieve selective deuterium incorporation into our ether products. We thus synthesised three representative (α-alkoxyalkyl)phosphonium salts *via* our standard protocol, and subjected each phosphonium salt to deuterolysis using NaOD in D_2_O. As anticipated, this did indeed result in formation of ether products in high yields with selective deuterium incorporation in the benzylic position (see Fig. 2). In each case, the major product was the dideuterated ether (constituting 64–74% of the ether product), with almost all of the remainder (23–32%) being monodeuterated ether, and a small portion being non-deuterated. Details on the mechanism of this reaction are given later in this article. The hydrogen atoms that are competitively installed instead of deuterium in the monodeuterated and non-deuterated products are likely to originate primarily from HDO formed through NaOD-mediated deprotonation of the (α-alkoxyalkyl)phosphonium salt intermediates of these reactions. This method enables deuterium incorporation to be achieved during the ether-forming step, thus obviating the need for prior preparation of deuterated precursors, and does not require the use of deuteride reagents.

### Ether formation using silyl ethers as starting materials

**Fig. 3:**
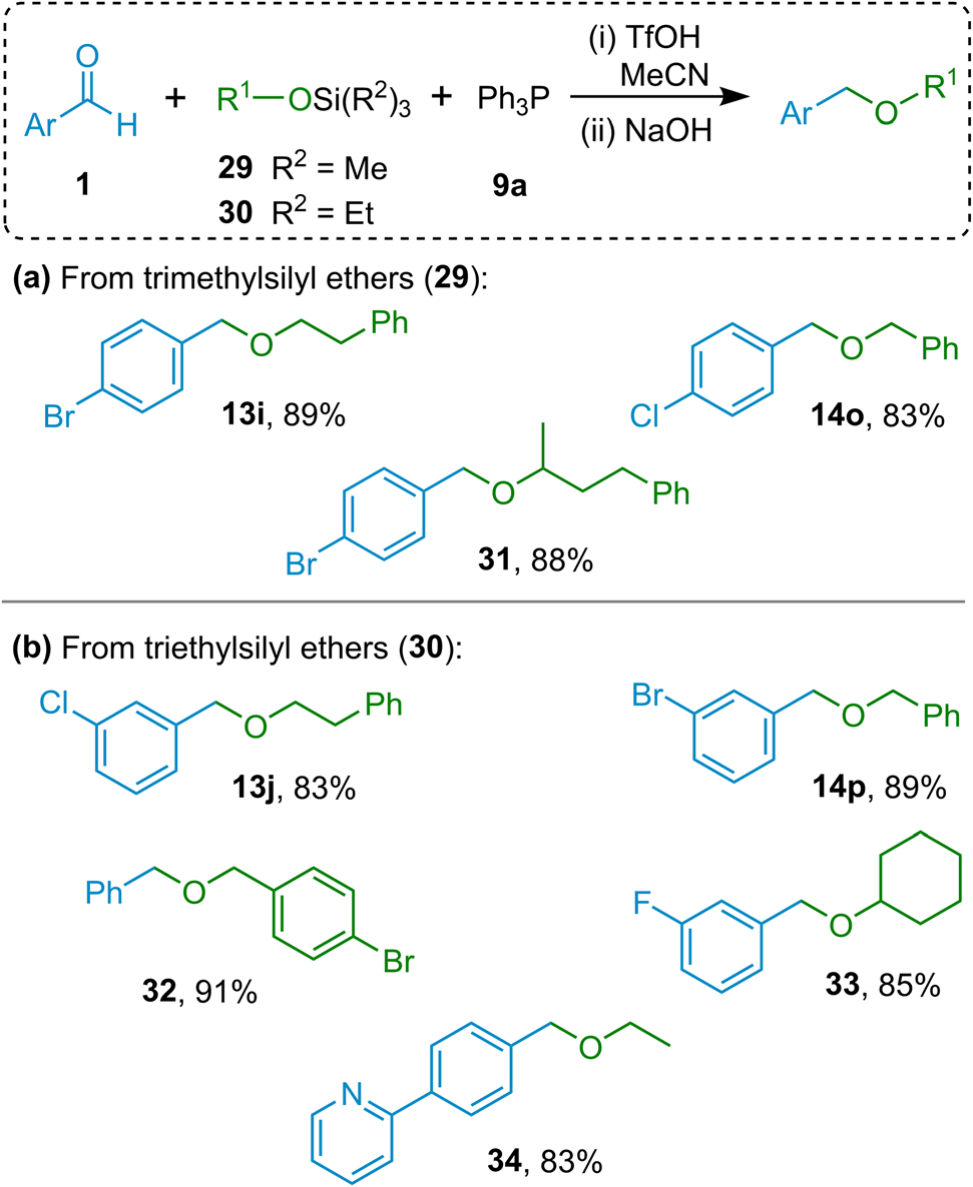
Substrate scope for hydrolytic etherification reactions of aromatic aldehydes with (a) trimethylsilyl ethers, and (b) with triethylsilyl ethers (isolated yields are shown).

Further to the results described above, it was also observed that trimethylsilyl and triethylsilyl ethers (TMS and TES ethers) could fulfil the role played by the alcohol reactant in the above reactions.^34^ Using silyl ethers as alcohol surrogates but otherwise maintaining the same reaction conditions used for the reactions discussed above, the methodology was found to be effective in the formation of benzyl ethers containing as the second oxygen-substituent non-benzylic primary alkyl groups (compounds 13i, 13j, 34 in Fig. 3), secondary alkyl groups (31, 33) and benzyl groups (14o, 14p, 32).

It is envisaged that this transformation could be utilised as a method of choice should a direct and efficient way of replacing a silyl ether group with a benzyl protecting group be sought. If conducting this protecting group swap using classical methodologies, extra steps and purifications would be required to deliver the benzylated product.

### Summary on substrate scope of hydrolytic etherification methodologies

The hydrolytic etherification methodologies developed in the project (starting from both alcohols and silyl ethers) described herein have proved to be applicable to a variety of aromatic aldehydes, enabling formation of ethers derived from benzaldehyde and 2-naphthaldehyde (ethers 13d, 14a, 14g, 25, 32; Fig. 1), from various *para*-halogen substituted benzaldehydes (ethers 13a–b, 13g–i, 14o, 15–18, 20–24, 26, 27 and 31), from *meta*-substituted benzaldehydes (ethers 13e, 13j, 14d–e, 14p, 33), from *ortho*-substituted benzaldehydes (ethers 13f and 14f), from benzaldehydes with mesomeric electron-withdrawing *para*-substituents (ethers 14b, 14c, 14j–m), and from aldehydes containing heteroaromatic groups (ethers 14h–i, 14n, 19, 34). (α-Alkoxyalkyl)phosphonium salts can be formed from aliphatic aldehydes – however, selective cleavage of the alkoxyalkyl group from phosphorus as an ether requires the development of separate methodology. Our research group is currently pursuing several approaches to achieve this.

Across the aldehyde and alcohol substrates used in construction of ethers, it is clear that the new hydrolytic methodology is tolerant of aryl halide functional groups, heteroaryl groups (*i.e.*, benzothiophen-2-yl (19), benzothiophen-3-yl (14n), thiophen-2-yl (14h), fur-2-yl (14i) and pyridyl (33) groups), alkenes (16, 17, 27) and alkynes (15). This set of functional groups includes examples that would be sensitive to catalytic hydrogenation approaches (or other reductive conditions) and hence ethers containing these functional groups would not be accessible using many existing methodologies for ether synthesis – *e.g.*, compounds analogous to 16, 17 and 27 (alkene-containing), 15 (alkyne-containing), 14k (ketone-containing), 14b (nitrile-containing), 14m (amide-containing) and 14j (ester-containing).

The hydrolytic etherification methodology described herein is thus applicable for the synthesis of benzyl ethers (and analogues thereof) containing a wide range of functional groups. We have demonstrated that the new methodology is applicable for formation of benzyl ethers containing functional groups that are sensitive to reduction and hydrolysis, and hence this approach will prove very useful as a means of introducing benzyl protecting groups into compounds containing reductively or hydrolytically unstable functional groups. In addition, the syntheses discussed above were carried out open to the ambient atmosphere, as these reactions do not require inert atmosphere conditions.

### Mechanistic experiments – (α-alkoxyalkyl)phosphonium salt formation

**Scheme 8:**
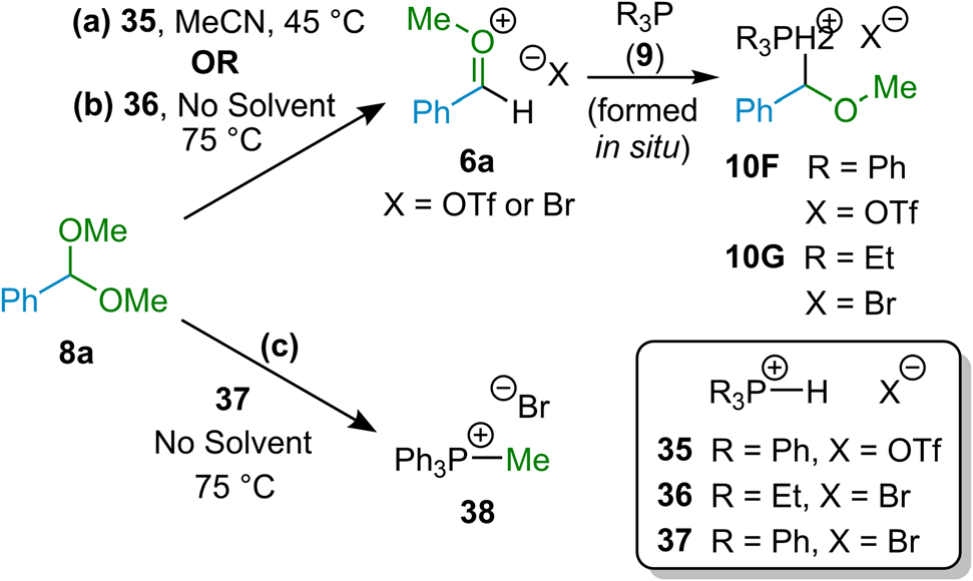
Reactions of acetal 8a with salts [Ph_3_PH]X enable independent generation of *O*-alkoxycarbenium ion 6a, and hence enable testing of whether such species can be intercepted by phosphines to give (α-alkoxyalkyl)phosphonum species 10. (a) Reaction of acetal 8a + [Ph_3_PH]OTf (35) in MeCN to give 10F; (b) solvent-free reaction of 8a + [Et_3_PH]Br (36) to give 10G; (c) solvent-free reaction of 8a + [Ph_3_PH]Br (37) to give [MePh_3_P]Br (38).

We focus first on the mechanism for (α-alkoxyalkyl)phosphonium salt formation; discussion is included below on the mechanism for the hydrolysis of these species (*i.e.*, of the ether forming step). As indicated above, our strategy in development of the hydrolytic etherification methodology described herein revolved around the transient generation of *O*-alkyloxocarbenium ion intermediates (species 6^+^ in Scheme 1, above) from reactions of alcohols with aldehydes in the presence of acid, and interception of these intermediates with phosphines in order to form (α-alkoxyalkyl)phosphonium salts (10) that could then be hydrolysed to form ethers (7). If *O*-alkyloxocarbenium ions (6^+^) are indeed involved as intermediates, then interception by phosphines (such as Ph_3_P (9a)) of independently generated *O*-alkyloxocarbenium ions (formed through alternative means to those used for the reactions discussed above) should be possible. In principle, *O*-alkyloxocarbenium salts 6a can be generated as a transient intermediate by treatment of dimethylacetal 8a with an acid (Scheme 8), and thus we undertook the reaction of 8a with tertiary phosphonium salt 35 ([Ph_3_PH]OTf), which functions as an acid to protonate 8a and also results in generation of Ph_3_P to act as a nucleophile towards the putative *O*-alkyloxocarbenium species (6a, with triflate as the counter-anion in this case).

Under our experimental conditions (with MeCN as solvent), it was observed that the reaction of acetal 8a with 35 ([Ph_3_PH]OTf) did indeed result in the formation of (α-alkoxyalkyl)phosphonium salt 10F (Scheme 8a). A similar experiment (conducted in the absence of solvent) has been reported previously by McNulty and co-workers, who found that (α-alkoxyalkyl)phosphonium salt 10G was formed if tertiary phosphonium salt 36 ([Et_3_PH]Br) was used instead of 35 (Scheme 8b).^12*i*^ Since McNulty and co-workers did also observe that treatment of 8a with tertiary phosphonium salt 37 ([Ph_3_PH]Br) led to formation of quaternary phosphonium salt 38 ([MePh_3_P]Br) as the major product (Scheme 8c),^12*i*^ we were careful to establish conclusively that no formation of phosphonium salt [MePh_3_P]OTf (*i.e.*, 38 with triflate rather than bromide as counter-anion) had occurred in our experiments involving combination of acetal 8a with 35 ([Ph_3_PH]OTf) (see Scheme 8a). A sample of the reaction mixture from this experiment was spiked with [MePh_3_P]OTf, and NMR spectroscopic analysis of this sample showed that new signals characteristic of [MePh_3_P]OTf had appeared in both the ^1^H and ^31^P NMR spectra in addition to the signals of 10F, indicating that no [MePh_3_P]OTf had been present in the reaction mixture prior to spiking.^35^

The result of the experiment described in Scheme 8a demonstrates that if *O*-alkyloxocarbenium ions (*e.g.*, 6a) are formed in the experiments described above, then they can be intercepted by Ph_3_P to form (α-alkoxyalkyl)phosphonium salts 10, *i.e.*, *O*-alkyloxocarbenium ions are plausible intermediates in our reactions.

We also conducted a control experiment involving delayed addition of Ph_3_P (9a) to a mixture of alcohol 5d, aldehyde 1b and TfOH (*i.e.*, a similar experiment to that represented in Scheme 5 above, but with delayed addition of Ph_3_P). While this did result in formation of (α-alkoxyalkyl)phosphonium salt 10D, the amount of this species formed (54% NMR spectral yield) was diminished in comparison to reactions conducted under our standard conditions. We attribute this to the occurrence of side-reactions involving the reactive *O*-alkyloxocarbenium ion intermediate. In order to verify the importance of acid and phosphine to the reaction outcome, separate experiments were undertaken in which phosphine and acid, respectively, were omitted from the reaction mixture. In both cases, no (α-alkoxyalkyl)phosphonium salt (10) was formed and thus, as expected, no ether formation occurred.

### Computational investigations

**Scheme 9:**
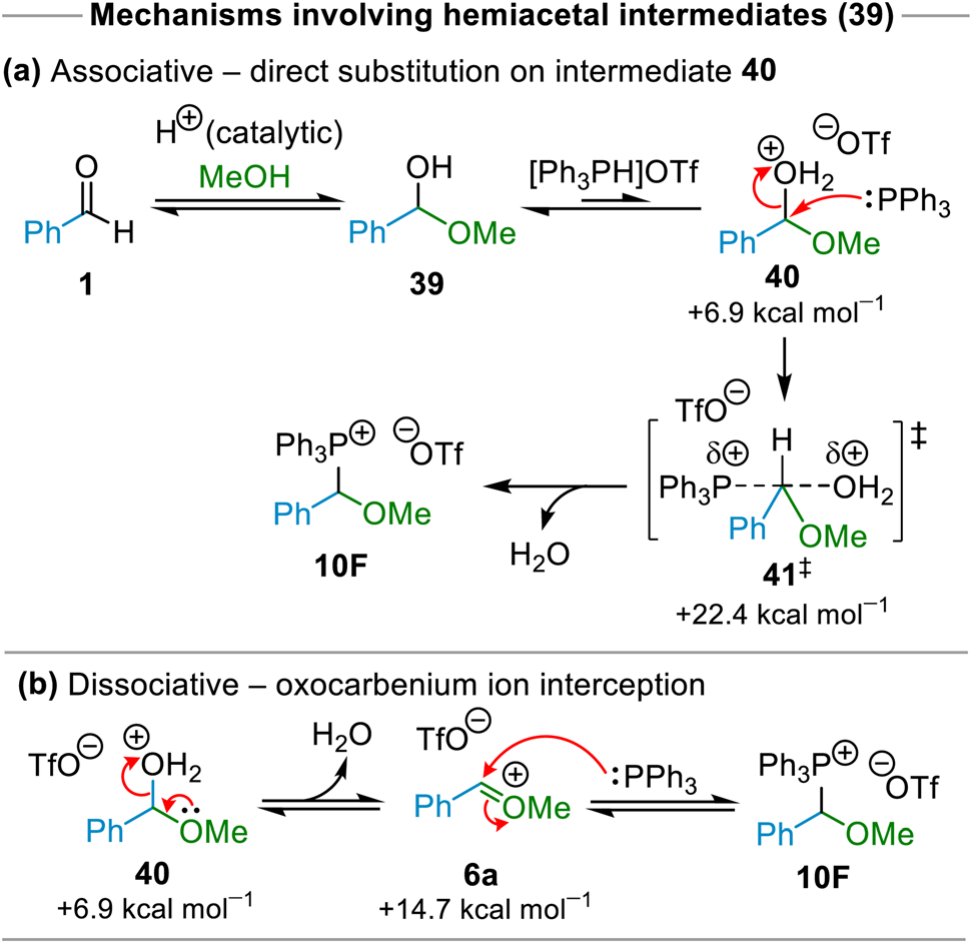
Possible mechanism(s) for formation of (α-alkoxyalkyl)phosphonium salt 10F*via* hemiacetal 39. Gibbs energy values shown (in kcal mol^−1^, at 298.15 K) are relative to the Gibbs energy of the starting materials, and were calculated at the ωb97XD/def2-TZV/PCM(CH3CN)//ωb97XD/6-31G(d,p)/PCM(CH3CN) level of theory.

**Scheme 10:**
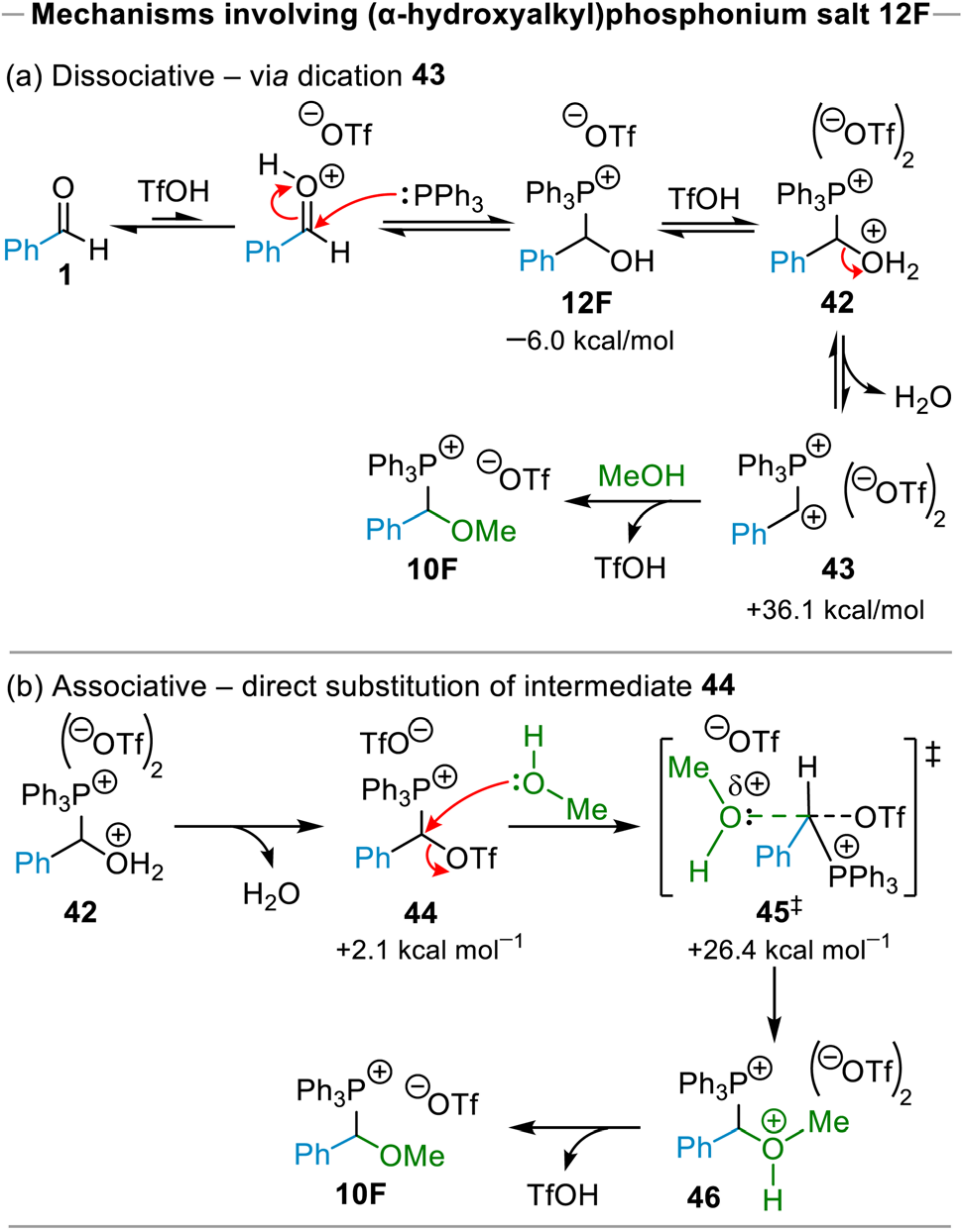
Possible mechanism(s) for formation of (α-alkoxyalkyl)phosphonium salt 10F*via* (α-hydroxyalkyl)phosphonium salt 12F. Gibbs energy values shown (in kcal mol^−1^, at 298.15 K) are relative to the Gibbs energy of the starting materials, and were calculated at the ωb97XD/def2-TZV/PCM(CH3CN)//ωb97XD/6-31G(d,p)/PCM(CH3CN) level of theory.

We also conducted quantum chemical calculations to derive further insight into the mechanism of the reaction, focusing on the formation of (α-alkoxyalkyl)phosphonium salt 10F from benzaldehyde, methanol and [Ph_3_PH]OTf as a representative example. Four mechanistic variants were investigated, each of which involves multiple elementary steps – these are shown in Schemes 9 and 10 below. The Gibbs energies (at 298.15 K) of key species involved in each mechanism (*i.e.*, high energy intermediates and/or transition states) were calculated at the ωb97XD/def2TZV/PCM(CH3CN)//ωb97XD/6-31G(d,p)/PCM(CH3CN) level of theory.

Two of the mechanisms investigated (shown in Scheme 9) involved formation of hemiacetal 39 from aldehyde 1 + MeOH followed by protonation of 39 to form intermediate 40. The mechanistic possibility shown in Scheme 9a involves associative S_N_2-type displacement occurs of H_2_O from monocationic intermediate 40 by Ph_3_P to form phosphonium salt 10F. The Gibbs energy of activation for this associative process was found to be relatively high, with the Gibbs energy of the highest transition state along the reaction pathway (41^‡^) being 22.4 kcal mol^−1^ higher in Gibbs energy than the starting materials (Scheme 9a). This is presumably due to the sterically crowded nature of TS 41^‡^.

In the second of the mechanistic possibilities involving formation of hemiacetal 39 and ionic intermediate 40, dissociation of H_2_O from 40 results in the formation of oxocarbenium ion 6a (14.7 kcal mol^−1^ higher in Gibbs energy than the starting materials), which then undergoes nucleophilic attack by Ph_3_P to form phosphonium salt 10F (Scheme 9b). The only transition state that could be located on the reaction potential energy surface for the combination of Ph_3_P + 6a was identical to TS 41^‡^ from Scheme 9a, 22.4 kcal mol^−1^ higher in Gibbs energy than the starting materials. However, there exists a distinct possibility that combination of Ph_3_P + 6a occurs *via* a lower energy TS in which there is minimal association of the oxocarbenium ion with H_2_O. The combination of Ph_3_P + 6a may even be essentially barrierless. Thus, we contend that the true Δ*G*^‡^ for the dissociative mechanism shown in Scheme 9b lies between 14.7 kcal mol^−1^ (the Gibbs energy of the oxocarbenium ion + Ph_3_P (+ the triflate counter-anion)) and 22.4 kcal mol^−1^ (the Gibbs energy of TS 41^‡^), with the latter barrier representing the upper limit for the Gibbs energy cost of a mechanism occurring *via* hemiacetal intermediate 39.

As was indicated above, (α-hydroxyalkyl)phosphonium salts were observed spectroscopically to be present in our reaction mixtures. In addition, several precedents exist in which phosphonium salts (*e.g.*, (diarylmethyl)phosphonium salts and (α-alkylthioalkyl)phosphonium salts) were proposed to have been formed through mechanisms that involved (α-hydroxyalkyl)phosphonium salts as intermediates.^25,30,37–39^ Certain of these mechanistic proposals were elaborated further, suggesting the possible involvement of dicationic intermediates such as 43 (formed from (α-hydroxyalkyl)phosphonium salts; see Scheme 10a).^25,30^ Prompted by these precedents, we considered further dissociative and associative mechanistic possibilities for (α-alkoxyalkyl)phosphonium salt formation in the presence of excess TfOH involving (α-hydroxyalkyl)phosphonium salts such as 12F as intermediates.

The first of these involved generation of (α-hydroxyalkyl)phosphonium salt 12F, which our calculations indicate should occur spontaneously (see Scheme 10a). This is consistent with our experimental observations (*vide supra*). Protonation of 12F to form dicationic intermediate 42 followed by dissociation of H_2_O to give dication 43, and reaction of 43 with MeOH leads to formation of phosphonium salt 10F (Scheme 10a). Our calculations on this mechanism showed that the Gibbs energy cost associated with formation of dicationic entity 43 was prohibitively high, at +36.1 kcal mol^−1^, indicating that this intermediate is energetically inaccessible at the temperatures employed for the reactions reported above.

The second possible associative mechanism investigated in which salt 12F functions as an intermediate involves S_N_2-type displacement of triflate from intermediate 44 by MeOH. Although the calculated Gibbs energy barrier for this reaction, at 26.4 kcal mol^−1^ (relative to starting materials; see Scheme 10b), is substantially lower than the Gibbs energy required for formation of dication 43, it nonetheless represents an unfeasibly high barrier for (α-alkoxyalkyl)phosphonium salt formation. It can thus be concluded that the two variants of the mechanism involving (α-hydroxyalkyl)phosphonium salt as an intermediate cannot operate at the temperatures at which our reactions were conducted.

### Proposed mechanism for (α-alkoxyalkyl)phosphonium salt formation

In addition to the computational data discussed above, there are other important factors indicating that (α-alkoxyalkyl)phosphonium salt formation through mechanisms involving (α-hydroxyalkyl)phosphonium salts as intermediates (*i.e.*, Mechanism 2 in Scheme 10) are likely not to be feasible. Firstly, in experiments in which aldehyde 1b, alcohol 5d, Ph_3_P (9a) and TfOH are combined (*i.e.*, in which the alcohol is present from the outset), (α-alkoxyalkyl)phosphonium salt 10D is formed in preference to (α-hydroxyalkyl)phosphonium salt 12D even early on in the reactions (see Scheme 5c and associated discussion above).^27^ Secondly, relatively facile reversal of (α-alkoxyalkyl)phosphonium salts (10) to starting materials (aldehyde, alcohol and phosphine) under basic conditions was observed in most solvents (*i.e.*, solvents other than MeCN or alcohols see Scheme 6 and associated discussion), and was also observed for (α-alkoxyalkyl)phosphonium salts with electron-rich α-aryl groups even in MeCN. In accordance with the principle of microscopic reversibility, the reverse reaction should follow the same mechanistic steps as the forward reaction but in reverse order. While dissociative breakdown of 10 to form 6^+^ (Scheme 9a) is conceivable, dissociation of 10 to give a high-energy dictation 43 in the absence of acid is not.

Our mechanistic experiments show that *O*-alkyloxocarbenium ions are plausible intermediates in our (α-alkoxyalkyl)phosphonium salt forming reactions. Our computational investigations show that a mechanism involving interception of an oxocarbenium ion intermediate by Ph_3_P (Scheme 9b) is energetically feasible, with a closely related associative process involving displacement of H_2_O from hemiacetal-derived intermediate 40 (Scheme 9a) being a plausible alternative. Other mechanistic possibilities were shown to require the formation of intermediates and/or transition states with unfeasibly high Gibbs energies.

Based on the accumulated evidence from our mechanistic experiments and computational studies, we propose that in our hydrolytic etherification reaction sequence, formation of (α-alkoxyalkyl)phosphonium salt 10 occurs by a pathway involving, initially, hemiacetal (*e.g.*, 39) and, subsequently, an intermediate such as 40 and then *O*-alkyloxocarbenium ion (*e.g.*, 6a) as transient intermediates, as shown in Scheme 9b for the representative example involving the combination of benzaldehyde, MeOH and Ph_3_P. While direct substitution of 40 by Ph_3_P is plausible (*i.e.*, with no involvement of 6a), we note that the mechanistic experiment detailed in Scheme 8a must involve formation of 6a in order for (α-alkoxyalkyl)phosphonium salt 10F to be produced, whereas the involvement of 40 is not required. Formation of (α-hydroxyalkyl)phosphonium salts such as 12F clearly does occur during these reactions, but this is a reversible process, and these species are not involved as intermediates on the reaction pathway. Analogous mechanisms have been proposed previously for reactions of arenes with aldehydes and phosphines in the presence of acid to form (diarylmethyl)phosphonium salts; these proposals involved protonation of the aldehyde to form oxocarbenium ion, reaction of this with a weakly nucleophilic arene to form diarylcarbenium ion intermediates that then undergo further reactions with phosphines.^25,36^

As an aside, it is possible, and perhaps likely, that several reactions reported in the literature that are proposed to involve (α-hydroxyalkyl)phosphonium salts as intermediates on pathways to other compounds may not occur by the originally proposed mechanisms. Instead, the reactions in these reports may well occur *via* much more energetically accessible oxocarbenium ions derived from aldehydes that (along with phosphine) are in equilibrium with the (α-hydroxyalkyl)phosphonium salt. These oxocarbenium ions may be intercepted by weak nucleophiles such as thiols,^30^ arenes,^25,36,38^ amides, carbamates or imides^37–39^ to generate reactive entities that, subsequently, are trapped by phosphine.

### Mechanism of (α-alkoxyalkyl)phosphonium salt hydrolysis

**Scheme 11:**
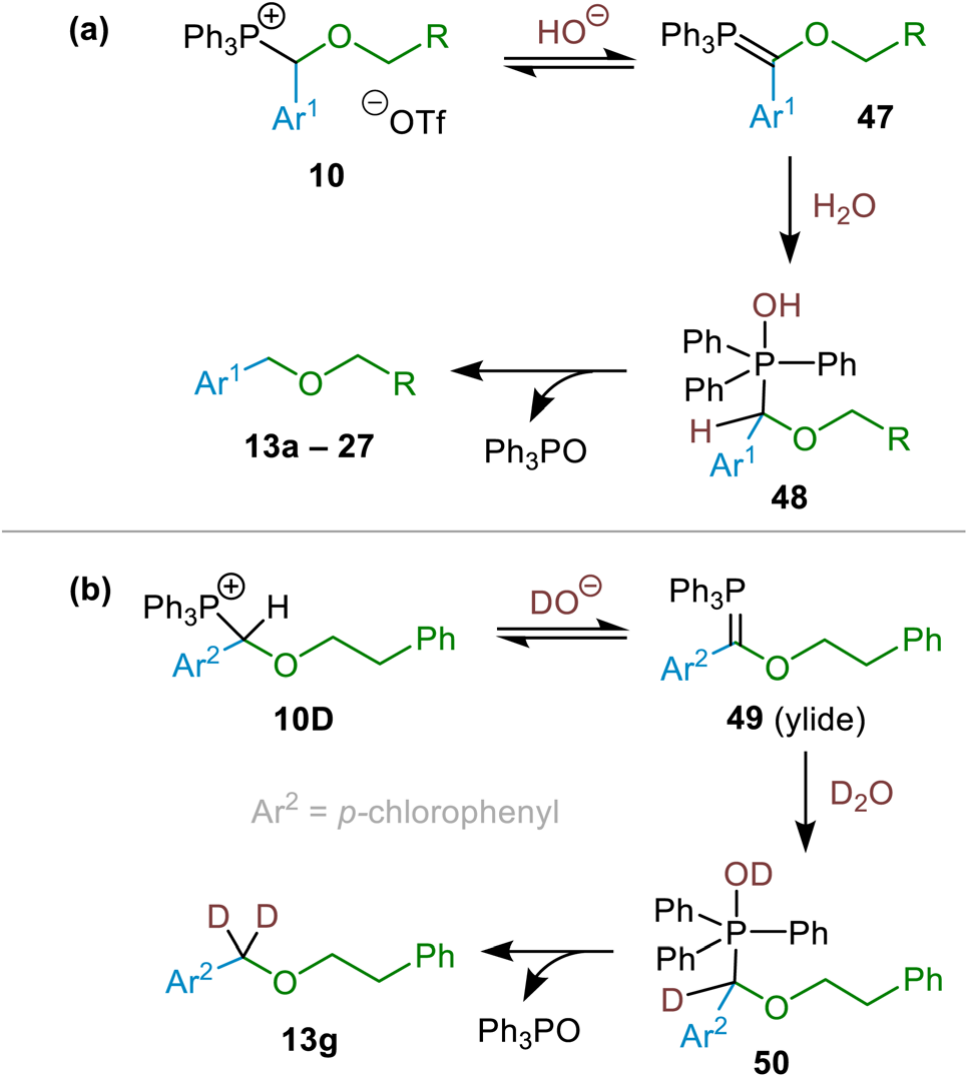
(a) Proposed general mechanism for the hydrolysis of (α-alkoxyalkyl)phosphonium salts (10) *via* ylide formation. (b) Proposed mechanistic pathway for the formation of 13g, with double deuteration of the benzylic position.

Finally, turning to the mechanism of (α-alkoxyalkyl)phosphonium salt hydrolysis, we consider that this process is likely to take place *via* the formation of an ylide (47; see Scheme 11a) followed by addition of water to this ylide. This mechanistic pathway has been established for hydrolysis and alcoholysis of ylides and reported previously.^13*a*,*b*^ It is also consistent with incorporation of two deuterium atoms into the benzylic positions of ethers 13g, 13h and 14n as the major products in the experiments described above (see Fig. 2 above) in which the (α-alkoxyalkyl)phosphonium salt precursors of 13g, 13h and 14n were subjected to deuterolysis using a solution of NaOD in excess D_2_O (see example in Scheme 11b, involving formation of ether 13g from (α-alkoxyalkyl)phosphonium salt 10D). We believe that (α-alkoxyalkyl)phosphonium salt deuterolysis proceeds *via* formation of an ylide (*i.e.*, through deprotonation by DO^−^), which then undergoes an addition of D_2_O to give a phosphorane, and that this, in turn, eliminates the phosphine oxide by-product 11a (Ph_3_PO), leading to incorporation of two deuterium atoms into the structure of the ether product (see Scheme 11b). The presence of HDO in these reactions (arising from deuteroxide-mediated deprotonation of (α-alkoxyalkyl)phosphonium salts) leads to the formation of monodeuterated ethers as minor products in these reactions (see Fig. 2 and associated discussion above).

## Conclusion

We have developed an alternative strategy to access ethers starting from readily available alcohol and aldehyde starting materials that involves “interception” of oxocarbenium ions using phosphines as “sacrificial” nucleophiles (*i.e.*, introducing a reaction that can out-compete acetal formation). This results in the formation of phosphonium salts that can be controllably decomposed to release ether products by hydrolysis. Thus, net reductive etherifications are achieved without hydrogen or hydride donors as reductants. It has never previously been possible to synthesise ethers directly from carbonyl compounds and alcohols without the need for a direct hydride donor. This makes the proposed two-stage one-pot reductive hydrolytic etherification reported herein an entirely new means of accessing these important structures.

The key phosphonium salt intermediates (10) involved in this etherification methodology have been shown by NMR spectroscopy to be produced essentially quantitatively in the reactions of aromatic aldehydes with phosphines and alcohols in the presence of acid. The structures of the phosphonium salt intermediates isolated from representative exemplar reactions have been unequivocally confirmed by X-ray diffractometric analysis. As expected, they comprise of a phosphonium cation in which the former carbon centre of the carbonyl is linked to the oxygen of the alcohol and to the *P*-centre. Under hydrolytic conditions, this species releases the ether moiety and a phosphine oxide, leading to an efficient etherification method that is applicable for many primary and secondary alcohols (43 examples) in high yields using general reaction conditions. The new approach is characterised by mild conditions, high to excellent yields and unexpected selectivity, allowing, for example, selective benzylation of a secondary alcohol even in the presence of a phenol group elsewhere in the structures of the reactant molecules. The results of mechanistic and computational investigations on the hydrolytic etherification reactions are consistent with the occurrence of the oxocarbenium ion interception process envisaged to occur in the design of the synthetic methodology or a closely related associative variant thereof.

## Data availability

The data supporting this article (synthetic details, experimental methods, characterisation data including copies of NMR spectra, and details of computational investigations) have been included as part of the ESI.† Crystallographic data for compounds 10A and 10B are included in the ESI,† but have also been deposited at the CCDC under accession numbers 2373866 and 2373867, respectively, and can be obtained from the CCDC website using the following URLs: compound 10A: https://www.ccdc.cam.ac.uk/structures/Search?access=referee&ccdc=2373866&Author=Dara+T.+Curran; compound 10B: https://www.ccdc.cam.ac.uk/structures/Search?access=referee&ccdc=2373867&Author=Dara+T.+Curran.

## Author contributions

Conceptualisation, P. A. B. and K. N.; methodology, P. A. B. and K. N.; investigation, D. T. C., M. S., K. N. and H. M. B.; formal analysis, D. T. C., M. S., K. N., H. M. B. and P. A. B.; writing – original draft, P. A. B. and D. T. C.; writing – review & editing, P. A. B., K. N., M. S. and D. T. C.; funding acquisition, P. A. B. and D. T. C.; resources, P. A. B.; supervision, P. A. B.

## Conflicts of interest

There are no conflicts to declare.

## Supplementary Material

SC-OLF-D4SC06203E-s001

SC-OLF-D4SC06203E-s002
