# Supplementary material for: Direct synthesis of ethers from alcohols & aldehydes enabled by an oxocarbenium ion interception strategy
Source: Chem Sci. 2025 Mar 4;16(16):6991–7003. doi: 10.1039/d4sc06203e (PMC11932332; doi:10.1039/d4sc06203e)
Supplement: SC-016-D4SC06203E-s001 [file SC-016-D4SC06203E-s001.pdf]

Supplementary Information (SI) for Chemical Science.
This journal is © The Royal Society of Chemistry 2025

Supporting Information

Synthesis of Ethers from Alcohols & Aldehydes Enabled by an Oxocarbenium Ion Interception Strategy

Dara T. Curran,[a] Marcin Szydło,[a] Helge Müller-Bunz,[a] Kirill Nikitin,[a] and Peter A. Byrne* [a,b]

[a] School of Chemistry, University College Dublin, Belfield, Dublin 4, Ireland.

[b] SSPC, the Science Foundation Ireland Research Centre for Pharmaceuticals, University College Dublin, Ireland.

* Corresponding author: peter.byrne@ucd.ie

Table of Contents

1. General Experimental 3

2. Synthesis of Starting Materials 4

3. Synthesis and Characterisation of (α-Alkoxyalkyl)phosphonium Triflates, **10** 8

4. Optimisation Experiments 13

5. Substrate Scope for Hydrolytic Etherification of Aldehydes & Alcohols 19

6. Synthesis of Deuterated Ethers Using NaOD in the Hydrolytic Etherification Protocol 42

7. Hydrolytic Etherification of Aldehydes using Silyl Ethers 47

8. Formation of **28** from **21** 54

9. Formation of (α-Alkoxyalkyl)phosphonium Mesylate **10H** and Ether **13a** Using Methanesulfonic Acid 55

10. Reaction using pre-made [Ph_3PH]OTf 57

11. Incompatible Substrates 58

12. Control Experiments 66

13. Mechanistic Investigations 71

14. Computational Details 78

15. DFT Calculation Data 80

16. NMR Spectra 97

17. NMR Spectra for Mechanistic Investigations 168

18. References 176

1. General Experimental

Reagents were purchased from major commercial suppliers as highest purity grade. Alcohols and aldehydes were used without any further purification with the exception of liquid aldehydes, which were distilled prior to use. Phosphines and trifluoromethanesulfonic acid (TfOH) were purchased from Fluorochem. Aldehydes and alcohols were purchased from Fischer Scientific, Fluorochem or Merck. Solvents for chromatography and work-up procedures, except in the case of ethyl acetate and cyclohexane, were used as obtained from commercial sources. Ethyl acetate and cyclohexane were purified by distillation – this was done due to the purity level of these solvents available to us during this project; with ethyl acetate and cyclohexane of sufficient purity, distillation is not necessary. Organic phases in reaction work-up procedures were dried using anhydrous magnesium sulfate.

NMR spectroscopic analysis: NMR spectra were recorded on Bruker Avance III 500 and Varian VnmrS 500, Bruker Avance III 400, Agilent/Varian VnmrS 400 and JEOL 400, and Bruker Avance III 300 NMR spectrometers. ^{1}H and ^{13}C NMR chemical sifts were referenced to trimethylsilane (TMS). The operating frequencies for ^{13}C NMR were 125 MHz, 100 MHz and 75 MHz. Chemical shifts (δ) are expressed as parts per million (ppm), positive shift being downfield from TMS; coupling constants (*J*) are expressed in Hertz (Hz). Splitting patterns in ^{1}H NMR spectra are designated as: s (singlet), bs (broad singlet), d (doublet), dd (doublet of doublets), ddd (doublet of doublets of doublets), t (triplet), td (triplet of doublets), q (quartet), quin (quintet) and m (multiplet). ^{31}P NMR chemical shifts were referenced to orthophosphoric acid. The operating frequencies for ^{31}P NMR were 162 MHz and 121 MHz. ^{19}F NMR chemical shifts were referenced to fluorotrichloromethane.

Mass spectrometric analysis: High resolution (precise) mass spectra (HRMS) were recorded using either electrospray ionisation (in positive ionization mode (ESI+) or negative ionization mode (ESI−)) or electron ionisation. Electrospray ionisation experiments were performed using a Waters LCT Premier TOF LC MS instrument or an Agilent-6546-QToF instrument, with 50 % acetonitrile/water containing 0.1% formic acid as eluent. Electron ionisation experiments were run using GC-HRMS on a GCT Premier GC-MS system. Samples were made up at a concentration of approximately 1 mg mL–1 or 0.1 mg mL^{-1}.

Melting points: Melting points for solids were measured on an Electrothermal IA9300 instrument and are uncorrected.

Infrared spectroscopic (IR) analysis: IR spectra were recorded on a Bruker ALPHA platinum ATR instrument. IR spectroscopy was used to help characterize novel compounds.

2. Synthesis of Starting Materials

Synthesis of 2-phenyleth-1-yl (trimethylsilyl) ether

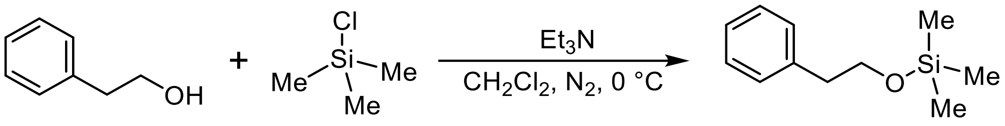

As per literature procedure,[1] 2-phenyl-1-ethanol (1.01 g, 8.00 mmol, 1.00 equivalent) and Et_3N (1.24 g, 12.0 mmol, 1.50 equivalents) were prepared in a Schlenk flask under an atmosphere of nitrogen containing a stirring bar and anhydrous CH_2Cl_2 (16.0 mL). The solution was stirred at 0 °C for 5 minutes. Trimethylsilyl chloride (1.10 g, 9.60 mmol, 1.20 equivalents) was added and the reaction mixture was stirred for 2 hrs at 0 °C. The contents of the reaction flask were transferred to a 100 mL separatory funnel and diluted with H_2O (25 mL). The aqueous layer was extracted with three CH_2Cl_2 portions (25 mL each). The combined organic layers were dried using $MgSO_4$ and filtered. The organic layer was concentrated *in vacuo* to give the product as a colourless oil (1.55 g, 7.92 mmol, 99% yield).

^{1}H NMR (300 MHz, $CDCl_3$) δ 7.43 – 7.31 (m, 5H), 3.91 (t, J = 7.3 Hz, 2H), 2.96 (t, J = 7.3 Hz, 2H), 0.20 (s, 9H).[2]

Synthesis of trimethyl((4-phenylbutan-2-yl)oxy)silane

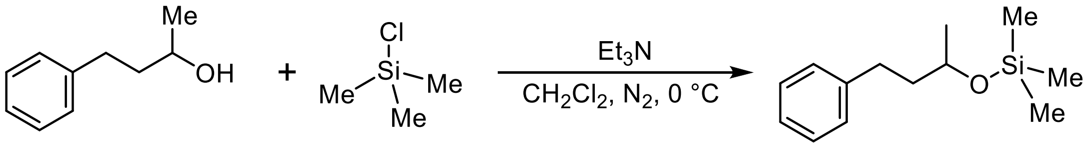

As per literature procedure,[1] 4-phenyl-2-butanol (1.22 g, 8.00 mmol, 1.00 equivalent) and Et_3N (1.23 g, 12.0 mmol, 1.50 equivalents) were prepared in a Schlenk flask under an atmosphere of nitrogen containing a stirring bar and anhydrous CH_2Cl_2 (16.0 mL). The solution was stirred at 0 °C for 5 minutes. Trimethylsilyl chloride (1.10 g, 9.60 mmol, 1.20 equivalents) was added and the reaction mixture was stirred for 2 hrs at 0 °C. The contents of the reaction flask were transferred to a 100 mL separatory funnel and diluted with H_2O (25 mL). The aqueous layer was extracted with three CH_2Cl_2 portions (25 mL each). The combined organic layers were dried using $MgSO_4$ and filtered. The organic layer was concentrated *in vacuo* to give the product as a colourless oil (1.78 g, 7.92 mmol, 99% yield).

^{1}H NMR (300 MHz, $CDCl_3$) δ 7.25 – 6.94 (m, 5H), 3.87 – 3.54 (m, 1H), 2.77 – 2.28 (m, 2H), 1.83 – 1.42 (m, 2H), 1.07 (d, J = 6.1 Hz, 3H), 0.10 (s, 9H).[3]

Synthesis of benzyl triethylsilyl ether

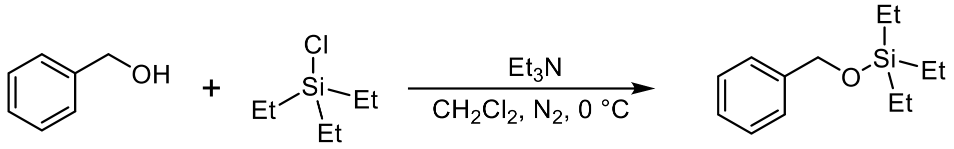

As per literature procedure,[1] 2-phenyl-1-ethanol (1.01 g, 8.00 mmol, 1.00 equivalent) and Et_3N (1.24 g, 12.0 mmol, 1.50 equivalents) were prepared in a Schlenk flask under an atmosphere of nitrogen containing a stirring bar and anhydrous CH_2Cl_2 (16.0 mL). The solution was stirred at 0 °C for 5 minutes. Triethylsilyl chloride (1.10 g, 9.60 mmol, 1.20 equivalents) was added and the reaction mixture was stirred for 2 hrs at 0 °C. The contents of the reaction flask were transferred to a 100 mL separatory funnel and diluted with H_2O (25 mL). The aqueous layer was extracted with three CH_2Cl_2 portions (25 mL each). The combined organic layers were dried using $MgSO_4$ and filtered. The organic layer was concentrated *in vacuo* to give the product as a colourless oil (1.55 g, 7.92 mmol, 99% yield).

^{1}H NMR (500 MHz, $CDCl_3$) δ 7.40 – 7.33 (m, 4H), 7.31 – 7.24 (m, 1H), 4.78 (s, 2H), 1.03 (t, J = 7.9 Hz, 9H), 0.70 (q, J = 7.9 Hz, 6H).[4]

Synthesis of 2-phenyleth-1-yl trimethylsilyl ether

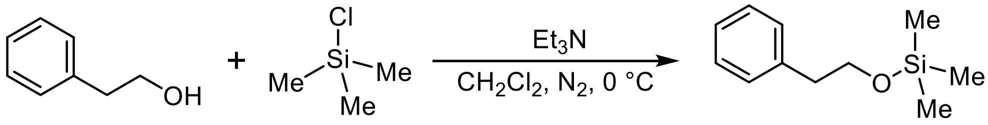

As per literature procedure,[1] 2-phenyl-1-ethanol (0.735 g, 6.02 mmol, 1.00 equivalent) and Et_3N (0.911 g, 9.00 mmol, 1.50 equivalents) were prepared in a Schlenk flask under an atmosphere of nitrogen containing a stirring bar and anhydrous CH_2Cl_2 (12.0 mL). The solution was stirred at 0 °C for 5 minutes. Triethylsilyl chloride (1.10 g, 9.60 mmol, 1.20 equivalents) was added and the reaction mixture was stirred for 2 hrs at 0 °C. The contents of the reaction flask were transferred to a 100 mL separatory funnel and diluted with H_2O (25 mL). The aqueous layer was extracted with three CH_2Cl_2 portions (25 mL each). The combined organic layers were dried using $MgSO_4$ and filtered. The organic layer was concentrated *in vacuo* to give the product as a colourless oil (1.55 g, 7.92 mmol, 99% yield).

^{1}H NMR (500 MHz, $CDCl_3$) δ 7.35 – 7.20 (m, 5.01H), 3.85 (t, J = 7.3 Hz, 1.99H), 2.89 (t, J = 7.3 Hz, 2H), 0.98 (t, J = 8.0 Hz, 9H), 0.62 (q, J = 7.9 Hz, 6H).[5]

Synthesis of trimethyl((4-phenylbutan-2-yl)oxy)silane

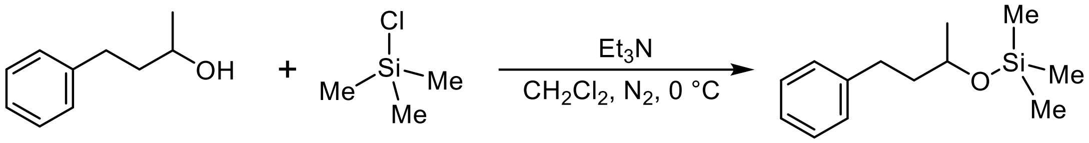

As per literature procedure, 4-phenyl-2-butanol (1.22 g, 8.0 mmol, 1.0 equivalent) and Et_3N (1.23 g, 12.0 mmol, 1.5 equivalents) were prepared in a Schlenk flask under an atmosphere of nitrogen containing a stirring bar and anhydrous CH_2Cl_2 (16.0 mL). The solution was stirred at 0 °C for 5 minutes. Trimethylsilyl chloride (1.10 g, 9.6 mmol, 1.2 equivalent) was added and the reaction mixture was stirred for 2 hrs at 0 °C. The contents of the reaction flask were transferred to a 100 mL separatory funnel and diluted with H_2O (25 mL). The aqueous layer was extracted with 3 x 25 mL portions of CH_2Cl_2. The combined organic layers were dried using $MgSO_4$ and filtered. The organic layer was concentrated *in vacuo* to give the title compound as a colourless oil (1.78 g, 7.92 mmol, 99% yield).

^{1}H NMR (300 MHz, $CDCl_3$) δ 7.25 – 6.94 (m, 5H), 3.87 – 3.54 (m, 1H), 2.77 – 2.28 (m, 2H), 1.83 – 1.42 (m, 2H), 1.07 (d, *J* = 6.1 Hz, 3H), 0.10 (s, 9H).[3]

Synthesis of 4-bromobenzyl triethylsilyl ether

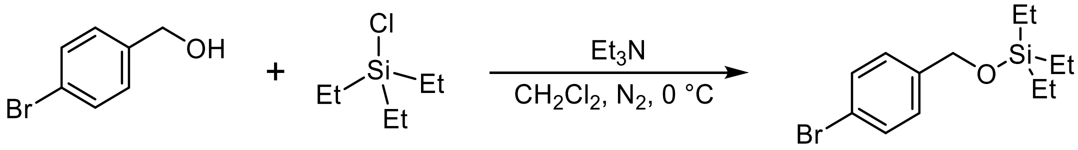

As per literature procedure, 4-bromobenzyl alcohol (0.3662 g, 1.96 mmol, 1.00 equivalent) and Et_3N (0.333 g, 3.29 mmol, 1.68 equivalents) were prepared in a Schlenk flask under an atmosphere of nitrogen containing a stirring bar and anhydrous CH_2Cl_2 (4.0 mL). The solution was stirred at 0 °C for 5 minutes. Triethylsilyl chloride (0.370 g, 2.46 mmol, 1.25 equivalents) was added and the reaction mixture was stirred for 2 hrs at 0 °C. The contents of the reaction flask were transferred to a 100 mL separatory funnel and diluted with H_2O (25 mL). The aqueous layer was extracted with three CH_2Cl_2 portions (25 mL each). The combined organic layers were dried using $MgSO_4$ and filtered. The organic layer was concentrated *in vacuo* to give the product as a colourless oil (0.579 g, 1.92 mmol, 98% yield).

^{1}H NMR (500 MHz, $CDCl_3$) δ 7.48 – 7.42 (m, 2H), 7.24 – 7.18 (m, 2H), 4.68 (s, 2H), 0.98 (t, *J* = 7.9 Hz, 9H), 0.65 (q, *J* = 7.9 Hz, 6H).[4]

Synthesis of cyclohexyl triethylsilyl ether

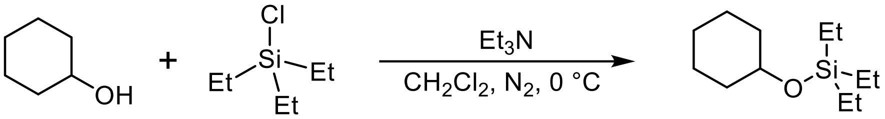

As per literature procedure, cyclohexanol (0.3075 g, 0.307 mmol, 1.00 equivalent) and Et_3N (0.455 g, 4.50 mmol, 1.47 equivalents) were prepared in a Schlenk flask under an atmosphere of nitrogen containing a stirring bar and anhydrous CH_2Cl_2 (6.0 mL). The solution was stirred at 0 °C for 5 minutes. Triethylsilyl chloride (0.543 g, 3.60 mmol, 1.17 equivalents) was added and the reaction mixture was stirred for 2 hrs at 0 °C. The contents of the reaction flask were transferred to a 100 mL separatory funnel and diluted with H_2O (25 mL). The aqueous layer was extracted with three CH_2Cl_2 portions (25 mL each). The combined organic layers were dried using $MgSO_4$ and filtered. The organic layer was concentrated *in vacuo* to give the product as a colourless oil (0.458 g, 0.213 mmol, 70% yield).

^{1}H NMR (500 MHz, $CDCl_3$) δ 3.56 (tt, *J* = 9.4, 3.9 Hz, 1H), 1.82 – 1.66 (m, 4H), 1.51 (m, 1H), 1.35 – 1.19 (m, 5H), 0.95 (t, *J* = 7.9 Hz, 9H), 0.58 (q, *J* = 7.9 Hz, 6H).[4]

Synthesis of triphenylphosphonium triflate

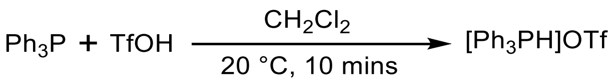

Triphenylphosphine (2.62 g, 10.0 mmol, 1.00 equivalent) and trifluoromethanesulfonic acid (1.50 g, 10.0 mmol, 1.00 equivalent) were added to CH_2Cl_2 (10 mL) added to a round bottom flask equipped with a stir bar. The reaction mixture was stirred at 20 °C (i.e., room temperature) for 10 mins. At the end of specified time, the solvent was removed *in vacuo* to give a viscous colourless oil. Diethyl ether (5 mL) was added to the round bottom flask with a stir bar and the reaction mixture was stirred for 10 mins to give a white solid. The precipitate was washed with diethyl ether (5 mL) and filtered to give the product as a white solid (4.1 g, 0.99 mmol, 99%).

^{1}H NMR (400 MHz, $CDCl_3$) δ 9.65 (d, *J* = 530.9 Hz, 1H), 7.81 – 7.75 (m, 9H), 7.67 – 7.63 (m, 6H).[a, 6]

^{19}F (376 MHz, $CDCl_3$) δ −78.1.[6]

^{31}P NMR (162 MHz, $CDCl_3$**)** δ 3.40.[b, 6]

---

[a] The signal appearing at δ 9.65 ppm in the ^{1}H NMR spectrum is reported in reference 6 as appearing at δ 9.38 ppm.

[b] The signal appearing at δ 3.40 ppm in the ^{31}P NMR spectrum is reported in reference 6 as appearing at δ 3.89 ppm.

3. Synthesis and Characterisation of (α-Alkoxyalkyl)phosphonium Triflates, **10**

(a) Formation of (α-cyclohexyloxybenzyl)triphenylphosphonium triflate, 10A

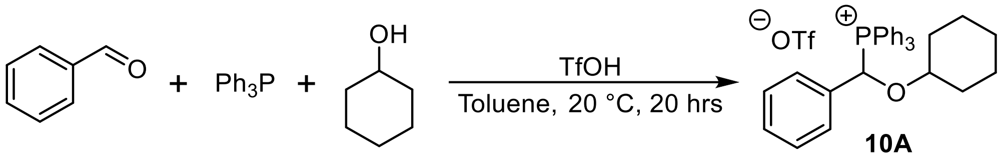

10A

To a solution of triphenylphosphine (131 mg, 0.499 mmol, 1.00 equivalents) in toluene (0.6 mL), cyclohexanol (100 mg, 1.00 mmol, 2.00 equivalents), benzaldehyde (64 mg, 0.6 mmol, 1.2 equivalents) and trifluoromethanesulfonic acid (85 mg, 0.66 mmol, 1.3 equivalents) were added. The reaction mixture was stirred at room temperature (20 °C) for 20 hrs after which time a solid was observed. The reaction mixture was analysed by NMR spectroscopy which indicated formation of salt **10A** (288 mg, 0.46 mmol, 98%). The solvent was removed under reduced pressure and the X-ray diffractometry quality crystals of **10A** were obtained by recrystallisation from chloroform-cyclohexane.

^{1}H NMR (400 MHz, $CDCl_3$) δ 7.78 (t, *J* = 7 Hz, 3H), 7.60 (td, *J* = 7 Hz, 4 Hz, 6H), 7.5 (td, *J* = 11, 4 Hz, 6H), 7.35 (t, *J* = 7 Hz, 1H), 7.17 (t, *J* = 7 Hz, 2H), 7.21 (t, *J* = 8 Hz, 2H), 6.68 (d, *J* = 8 Hz, 1H), 3.61 (tt, *J* = 9, 4Hz, 1H), 1.74 (m, 1H), 1.61 (m, 1H), 1.45 (m, 2H), 1.35 (m, 1H), 1.20 (m, 1H), 1.18 (m, 1H), 1.15 (m, 1H), 1.11 (m, 1H), 1.08 (m, 1H).

^{13}C NMR (75 MHz $CDCl_3$) δ 135.2 (d, *J* = 3 Hz), 134.9 (d, *J* = 9 Hz), 130.3 (d, *J* = 3 Hz), 131.7 (bs), 129.9 (d, *J* = 12 Hz), 129.5 (d, *J* = 6 Hz), 128.7 (d, *J* = 3 Hz), 120.7 (q, *J* = 321 Hz), 116.0 (d, *J* = 83 Hz), 77.4 (d, *J* = 67 Hz), 80.5 (d, *J* = 10 Hz), 32.9, 31.0, 23.2, 23.5, 25.2.

^{31}P NMR (161 MHz, $CDCl_3$) δ 21.7.

^{19}F NMR (376 MHz, $CDCl_3$) δ −78.0.

(b) Formation of (α-isopropoxy)benzyl-tris-(4-chlorophenyl)phosphonium triflate, 10B

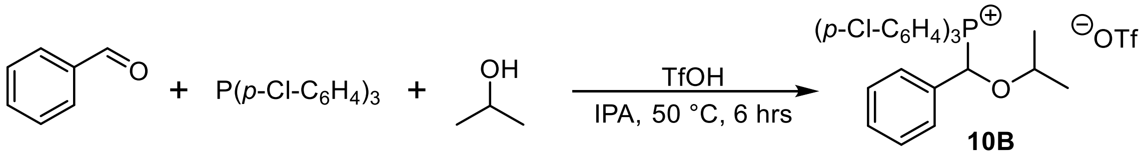

10B

To a suspension of *tris*(4-chlorophenyl)phosphine (183 mg, 0.500 mmol, 1.0 equivalents) in isopropanol (0.8 mL), benzaldehyde (64 mg, 0.60 mmol, 1.2 equivalents) and trifluoromethanesulfonic acid (85 mg, 0.66 mmol, 1.3 equivalents) were added. The reaction mixture was stirred in a heating bath set to 50 °C for 6 hrs after which time the system was allowed to cool to room temperature and formation of a solid was observed. The reaction mixture was analysed by NMR spectroscopy which indicated

formation of salt **10b** (321 mg, 0.480 mmol, 99%). The X-ray diffractometry quality crystals of **10B** were obtained from the reaction mixture.

^{1}H NMR (400 MHz, $CDCl_3$) δ 7.63 (dd, *J* = 9, 3 Hz, 6H), 7.52 (dd, *J* = 11, 9 Hz, 6H), 7.40 (t, *J* = 7 Hz, 1H), 7.30 (d, *J* = 8 Hz, 2H), 7.27 (t, *J* = 8 Hz, 2H), 6.97 (d, *J* = 8 Hz, 1H), 3.97 (m, 1H), 1.16 (t, *J* = 6 Hz, 3H), 1.08 (t, *J* = 6 Hz, 3H).

^{13}C NMR (101 MHz, $CDCl_3$) δ 142.9 (d, *J* = 4 Hz), 136.2 (d, *J* = 9 Hz), 131.0 (bs), 130.6 (d, *J* = 13 Hz), 129.0 (d, *J* = 3 Hz), 129.4 (d, *J* = 6 Hz), 130.5, 120.2 (q, *J* = 321 Hz), 113.9 (d, *J* = 85 Hz), 76.8 (d, *J* = 67 Hz), 75.6 (d, *J* = 11 Hz), 23.0, 21.3.

^{31}P NMR (161 MHz, $CDCl_3$) δ 21.5.

^{19}F NMR (376 MHz, $CDCl_3$) δ −78.0.

Table S1: Crystal data and structure refinement for **10A**•OTf and **10B**•OTf. CCDC 2373866 and 2373867 contain the X-ray crystallographic data for this paper.

	**10A**•OTf	**10B**•OTf
Empirical formula	$C_{32}H_{32}F_3O_4PS$	$C_{29}H_{25}Cl_3F_3O_4PS$
Molecular formula	$[C_{31}H_{32}OP]^+[CF_3SO_3]^-$	$[C_{28}H_{25}OP]^+[CF_3SO_3]^-$
Formula weight	600.61	663.87
Temperature	100(2) K	100(2) K
Wavelength	0. 71073 Å	1.54184 Å
Crystal system	orthorhombic	monoclinic
Space group	$Pca2_1$	$P2_1/n$
Unit cell dimensions	a = 17.0140(2) Å α = 90°	a = 9.49648(7) Å α = 90°
	b = 9.10342(11) Å β = 90 °	b = 16.70850(12) Å β = 9598046°
	c = 18.8380(3) Å γ = 90°	c = 18.79840(13) Å γ = 90°
Volume	2917.74(6) $Å^3$	2966.54(4) $Å^3$
Z	4	4
Density (calculated)	1.367 mg/m^3	1.486 mg/m^3
Absorption coefficient	0.221 mm^{-1}	4.43 mm^{-1}
F(000)	1256	1360
Crystal size	0.2179 × 0.1229 × 0.1153 mm^3	0.0989 × 0.0748 × 0.0555 mm^3
Theta range for data collection	3.44 to 29.22°	3.52 to 76.39°
Index ranges	–22<=h<=22, –12<=k<=11, –23<=l<=23	–11<=h<=11, –20<=k<=20, –23<=l<=23
Reflections collected	29792	61025
Independent reflections	7057<br>R_{int} = 0.0331	6205<br>R_{int} = 0.0295
Completeness to theta = 76.38°	99.5 %	99.6 %
Absorption correction	Analytical	Analytical
Max. and min. transmission	0.875 and 0.663	0.825 and 0.720
Refinement method	Full–matrix least–squares on F^2	Full–matrix least–squares on F^2
Data / restraints / parameters	7057 / 1 / 370	6025/0/372
Goodness–of–fit on F^2	1.062	1.045
R indices (all data)	R1 = 0.0341, wR2 = 0.0701	R1 = 0.0418, wR2 = 0.1059

(c) Formation of (α-ethoxybenzyl)triphenylphosphonium triflate, 10C

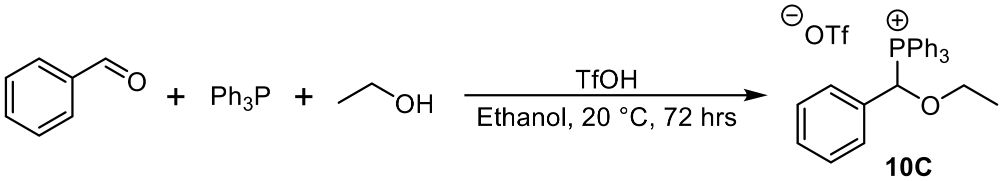

To a suspension of triphenylphosphine (131 mg, 0.500 mmol, 1.00 equivalent) in ethanol (0.8 mL), benzaldehyde (64 mg, 0.60 mmol, 1.2 equivalents) and trifluoromethanesulfonic acid (85 mg, 0.66 mmol, 1.3 equivalents) were added. The reaction mixture was stirred at room temperature (20 °C) for 72 hrs. The reaction mixture was analysed by NMR spectroscopy which indicated formation of salt **10C** (241 mg, 0.440 mmol, 97%). No crystalline material was obtained upon removal of the solvent under reduced pressure.

^{1}H NMR (400 MHz, CDCl3) δ 7.76 (t, *J* 8Hz, 3H), 7.58 (m, 6H), 7.55 (m, 6H), 7.33 (d, *J* = 8 Hz, 1H), 7.17 (t, *J* = 8 Hz, 1H), 7.12 (t, *J* = 8 Hz, 2H), 6.70 (d, *J* = 8 Hz, 1H), 3.77 (dq, *J* = 10, 7 Hz, 1H), 3.67 (dq, *J* = 10, 7 Hz, 1H), 1.12 (t, *J* = 7 Hz, 3H).

^{13}C NMR (75 MHz $CDCl_3$) δ 135.2 (d, *J* = 3 Hz), 134.8 (d, *J* = 9 Hz), 130.3 (d, *J* = 3 Hz), 130.6 (bs), 130.0 (d, *J* = 12 Hz), 129.5 (d, *J* = 5 Hz), 128.8 (d, *J* = 2 Hz), 120.4 (q, *J* = 324 Hz), 116.1 (d, *J* = 83 Hz), 78.0 (d, *J* = 68 Hz), 68.0 (d, *J* = 12 Hz), 15.1.

.**^{31}P NMR** (161 MHz, $CDCl_3$) δ 21.6.

^{19}F NMR (376 MHz, $CDCl_3$) δ −78.0.

(d) Formation of (α-(2-phenyl-1-ethoxy)-4-chlorobenzyl)triphenylphosphonium triflate, 10D

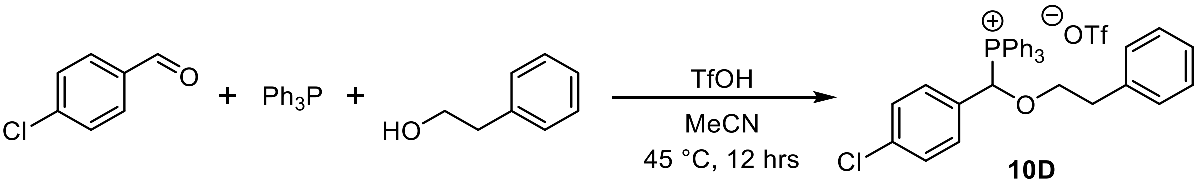

To a solution of 4-chlorobenzaldehyde (141 mg, 1.00 mmol, 1.00 equivalent) in acetonitrile (2.0 mL), was added triphenylphosphine (262 mg, 1.00 mmol, 1.00 equivalent) and trifluoromethanesulfonic acid (166 mg, 1.1 mmol, 1.1 equivalents). This was followed by 2-phenyl-1-ethanol (244 mg, 2.00 mmol, 2.00 equivalents) and the reaction mixture was stirred in a heating bath set to 45 °C. The reaction mixture was allowed to rest for a period of time after which **10D** precipitated from the reaction mixture. The precipitate was washed with diethyl ether (5 mL) and filtered to give the product as a white solid (499 mg, 0.760 mmol, 76%).

Melting point: 165 – 168 °C.

IR $\tilde{\nu}_{max}$ (ATR): 2960 (C—H stretch), 2678 (O—H stretch), 1695 (C=O stretch), 1652 (C=C stretch), 1437 (P^+—Phenyl stretch), 1108 (P^+—Ph stretch) cm^{-1}.

HRMS (ESI+): Calculated for $[M]^+$ = $[C_{33}H_{29}ClOP]^+$ *m/z* = 507.1644; found *m/z* = 507.1638.

^{1}H NMR (400 MHz, $CDCl_3$) δ 7.86 – 7.67 (m, 3H), 7.65 – 7.39 (m, 12H), 7.18 (m, 3H), 7.10 (d, *J* = 7.9 Hz, 2H), 7.03 – 6.95 (dd, *J* = 7.9, 3.9 Hz, 2H), 6.94 – 6.84 (m, 2H contains 6.90 (d, *J* = 8.4 Hz, 2H) 6.89 (app d, *J* = 8.5 Hz, 1H), 4.11 (dt, *J* = 9.3, 3.8 Hz, 1H), 3.82 (dt, *J* = 9.3, 3.8 Hz, 1H), 2.77 (t, *J* = 6.5 Hz, 2H).

^{13}C NMR (101 MHz, $CDCl_3$) δ 138.3, 136.3 (d, *J* = 4 Hz), 135.1 (d, *J* = 3 Hz), 134.7 (d, *J* = 9 Hz), 130.9 (d, *J* = 5 Hz), 130.0 (d, *J* = 13 Hz), 128.9, 128.8, 128.8, 128.2, 126.3, 115.8 (d, *J* = 83 Hz), 76.6, 72.2 (d, *J* = 12 Hz), 36.0.

^{31}P NMR (162 MHz, $CDCl_3$) δ 21.9.

^{19}F NMR (376 MHz, $CDCl_3$) δ −78.2.

4. Optimisation Experiments

4.1. Formation of (α-Alkoxyalkyl)phosphonium Triflate, 10D

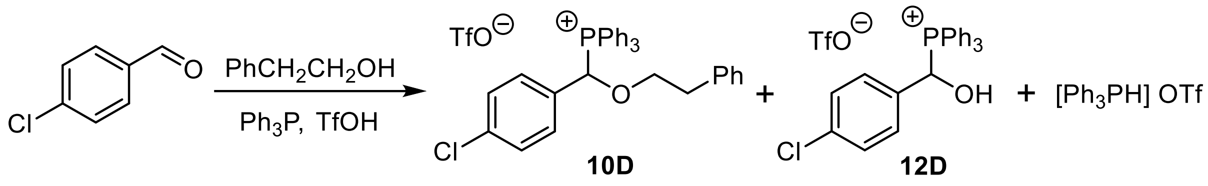

General Procedure A: Solvent (2.0 mL; see Table S3 for details of the solvents employed) was added to a round bottom flask equipped with a stir bar. This was followed by triphenylphosphine (288 – 294 mg, 1.10 – 1.20 mmol, 1.10 – 1.20 equivalents), trifluoromethanesulfonic acid (180 – 194 mg, 1.20 – 1.30 mmol, 1.20 – 1.30 equivalents) and 4-chlorobenzaldehyde (140 – 145 mg, 1.00 – 1.04 mmol, 1.00 – 1.04 equivalent). The reaction mixture was stirred at room temperature for 5 minutes, followed by addition of 2-phenyl-1-ethanol (219 – 246 mg, 1.80 – 2.00 mmol, 1.80 – 2.00 equivalents) and the flask was then closed by addition of a plastic lid. The reaction mixture was then heated in an oil bath at the specified temperature (see Table S2 below for details of the reaction temperatures investigated) for 10 – 24 hrs. Upon completion of the reaction, an aliquot was removed from the reaction mixture and added to an NMR tube. This was then diluted by addition of $CDCl_3$ (0.6 mL). An approximate ^{31}P NMR spectral yield was then determined using the method proposed by Montchamp and co-workers.[7] The ^{31}P NMR spectral yield of a given phosphorus-containing compound was obtained by expressing the integration of the signal of that compound in the ^{31}P NMR spectrum of the reaction mixture as a percentage of the sum of the integrations of all of the signals present in the ^{31}P NMR spectrum.

The signals of the species formed in these reactions appeared at the following approximate chemical shifts in ^{31}P NMR spectra taken on samples of the reaction mixtures diluted in $CDCl_3$:

Assigned to (α-alkoxyalkyl)phosphonium salt **10D**: δ_P *ca.* 22

Assigned to (α-hydroxyalkyl)phosphonium salt **12D**: δ_P *ca.* 21

Assigned to $[Ph_3PH]$ OTf : δ_P 2 – 4 ppm

The precise chemical shifts of the ^{31}P NMR spectral signals of **10D** and **12D** did vary over a range of *ca.* 0.5 ppm between different experiments due to differences in sample composition.

(a) Experiments to determine the optimal reaction temperature

The reactions in Table S2 were conducted according to General Procedure A. An estimated yield for each reaction was determined using the ^{31}P NMR spectroscopic method described above in General Procedure A. Optimisation for the formation of (α-alkoxyalkyl)phosphonium triflate **10D** began by varying the temperature of the reaction mixture. A reaction conducted using MeCN solvent at 45 °C (heating bath temperature) led to an 80% ^{31}P NMR spectral yield of **10D** (i.e., 80% of the phosphorus-containing material in the reaction mixture was observed to be **10D** by ^{31}P NMR spectroscopic analysis – see Table S2, Entry 1). In further experiments, the temperature of the reaction mixture was increased (Table S2, Entries 2 – 5) but the yield of product decreased in each case at higher temperatures.

Table S2: Temperature optimisation for formation of triflate, **10D**

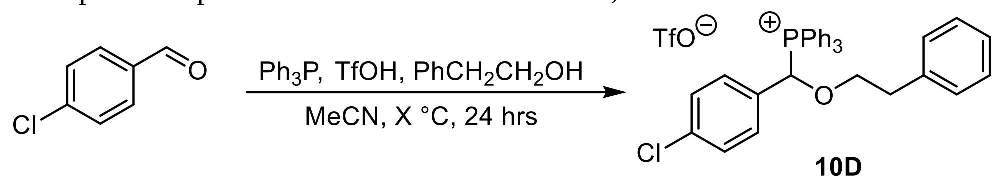

10D

Entry	Heating Bath Temp. (°C)	^{31}P NMR Spectral Yields [a]	
		**10D** (%)	Side-Product (%)
1	45	80 [b]	0
2	55	75	17
3	60	70	12
4	70	72	13
5	75	55	23
6	20	87	0
**7**	**45**	**95**	**0**

[a] The quantity of product formed was determined from relative integrations in ^{31}P NMR spectra of the reaction mixtures. The mass balance was accounted for in each case by (α-hydroxyalkyl)phosphonium salt **12D** and $[Ph_3PH]OTf$;

[b] 1.2 equivalents of $PhCH_2CH_2OH$ was used in this reaction.

(b) Experiments to determine the optimal reaction solvent

The reactions in Table S3 were conducted according to General Procedure A. An estimated yield for each reaction was determined using the ^{31}P NMR spectroscopic method described above in General Procedure A. A variety of solvents were chosen for the solvent screen in order to observe the quantity of **10D** formed. Efforts were directed at increasing the reaction temperature in toluene with the purpose of decreasing the reaction time. Thus, the reaction time was successfully decreased from 24 hrs to 8 hrs in entries 2 – 6 while maintaining the quantity of **10D** formed. A range of polar and non-polar solvents were screened which all enabled formation of **10D** in good to excellent conversions as quantified by ^{31}P

NMR spectral analysis. Alcohols were not investigated in this solvent screen as competitive formation of solvent-derived (α-alkoxyalkyl)phosphonium triflate in such experiments would occur. However, see the substrate scope section, in which alcohols could be used effectively as solvents in instances in which formation of solvent-derived (α-alkoxyalkyl)phosphonium triflate was the objective. A 2:1 mixture of toluene/$CHCl_3$ gave a ^{31}P NMR spectral yield of 95% in 6 (Table S3, Entry 5). However, as one overarching aim of this work is to minimise the use of halogenated reagents, it was decided not to pursue approaches based around utilisation of chlorinated solvents. The reaction was repeated using MeCN at shorter reaction times (10 hrs) and a yield of 95% of **10D** was observed (Table S3, Entry 1). The reaction was also attempted neat, i.e., in the absence of any solvent. However, the (α-alkoxyalkyl)phosphonium triflate salt was formed in a yield of only 41% (Table S3, Entry 10). Finally, it is hypothesised that water is formed in these reactions, meaning that water does not appear to adversely affect the formation of (α-alkoxyalkyl)phosphonium salts. To determine whether addition of water might enhance the process, an experiment was conducted using MeCN with 10% v/v H_2O as the solvent, but this gave a low yield (Table S3, Entry 11).

Table S3: Solvent screen for formation of (α-alkoxyalkyl)phosphonium triflate, **10D**

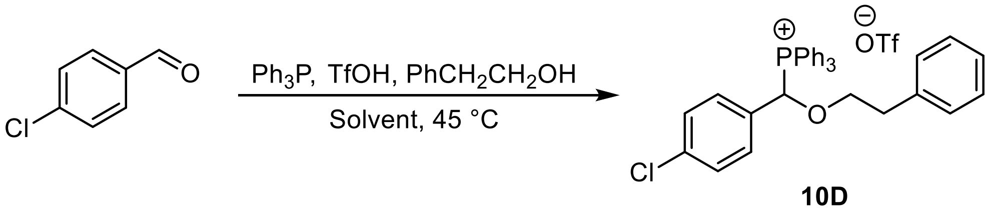

10D

Entry	Solvent (Concentration)	Heating Bath Temp. (°C)	^{31}P NMR Spectral Yield of **10D** (%) [a]	Time (hrs)
**1**	**MeCN (0.5 mol L^{-1})**	**45**	**95**	**10**
2	Toluene (0.5 mol L^{-1})	100	89	6
3	MeCN/Toluene (2:1) (0.5 mol L^{-1})	60	82	12
4	$CHCl_3$ (0.5 mol L^{-1})	65	86	6
5	Toluene/$CHCl_3$ (2:1) (0.5 mol L^{-1})	65	95	6
6	CH_2Cl_2 (0.5 mol L^{-1})	45	86	6
7	EtOAc (0.5 mol L^{-1})	45	75	10
8	Acetone (0.5 mol L^{-1})	45	44	10
9	THF (0.5 mol L^{-1})	45	91	10
10	Neat	100	41	6
11	MeCN/H_2O (9:1) (0.5 mol L^{-1})	48	45	10

[a] The quantity of product formed was determined from relative integrations in ^{31}P NMR spectra of the reaction mixtures. The mass balance was accounted for in each case by (α-hydroxyalkyl)phosphonium salt **12D** and $[Ph_3PH]OTf$;

(c) Experiments to determine the optimal relative amounts to use of each reactant

The reactions in Table S4 were conducted according to General Procedure A. An estimated yield for each reaction was determined using the ^{31}P NMR spectroscopic method described in General Procedure A. To determine the optimal equivalents of reagent in the transformation, a series of experiments were carried out (Table S4). Adding an equimolar amount of each reagent (Table S4, Entry 1) resulted in only a moderate yield of **10D** (Table S4, Entry 1). However, by increasing the amount of alcohol used to 1.9 equivalents, the yield increased significantly to 87% (Table S4, Entry 2). Increasing the amounts of Ph_3P and TfOH added (Table S4, Entry 5 and 6) resulted in diminished yields of 74% and 77%, respectively. Stepwise increases in the alcohol concentration of the reaction showed that there was an increase in the yield of **10D** (Table S4, Entries 7 – 11). Finally, by significantly increasing the amount of aldehyde to 1.5 equivalents and using 1.0 equivlanet of alcohol, a yield of 75% was obtained (Table S4, Entry 12). The purpose of this was to show the alcohol can be the limiting reagents and the reaction can still proceed efficiently, albeit at a slightly lower conversion.

Table S4: Investigation into optimal reactant quantities to use in the formation of (α-alkoxyalkyl)phosphonium triflate, **10D**

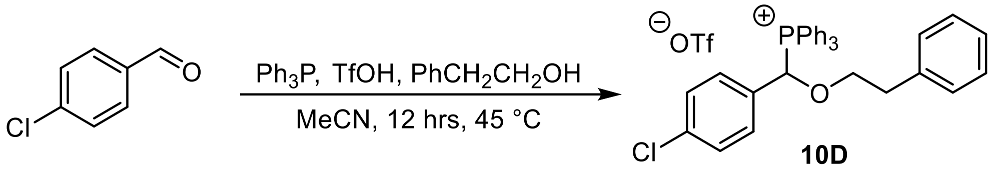

10D

Entry	Ph_3P (eq)	TfOH (eq)	Aldehyde (eq)	$PhCH_2CH_2OH$ (eq)	Time (hrs)	% Formation of **10D** [a]
1	1.0	1.0	1.0	1.0	12	67
2	1.0	1.0	1.0	1.9	12	87
3	1.1	1.1	1.0	1.9	12	79
4	1.2	1.2	1.0	1.9	12	88 [b]
5	1.4	1.5	1.0	1.9	24	75
6	1.6	1.7	1.0	1.9	24	74
7	1.1	1.2	1.0	1.2	24	77
8	1.1	1.2	1.0	1.4	24	74
9	1.1	1.2	1.0	1.5	24	71
10	1.1	1.2	1.0	1.7	36	82
11	1.1	1.2	1.0	2.0	12	92
12	1.1	1.2	1.5	1.0	12	75
**13**	**1.1**	**1.2**	**1.0**	**1.9**	**10**	**95**

[a] The quantity of product formed was determined from relative integrations in ^{31}P NMR spectra of the reaction mixtures. The mass balance was accounted for in each case by (α-hydroxyalkyl)phosphonium salt **12D** and $[Ph_3PH]OTf$;

[b] Experimental details for this reaction can be found in Section 10.

4.2. Hydrolysis of (α-Alkoxyalkyl)phosphonium Triflate, 10D

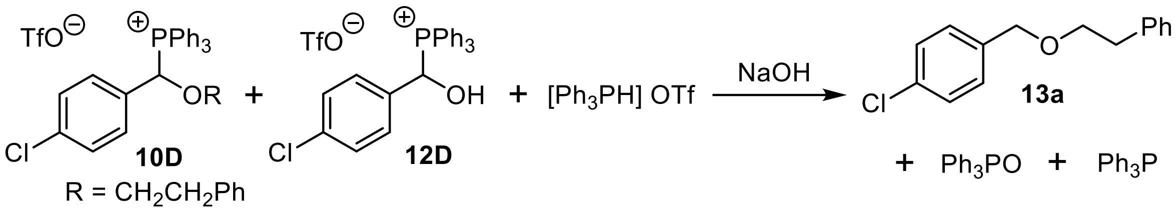

General Procedure B: To a reaction mixture generated as outlined in General Procedure A (with no aliquot removed for NMR spectroscopic analysis), NaOH solution was added (see Table S5 for details on the concentration and volume of NaOH solution employed per experiment). The reaction mixture was stirred at room temperature for 1.5 hrs at a high stirring rate to ensure mixing of the biphasic reaction mixture. The reaction mixture was transferred to a 50 mL separatory funnel. A saturated brine solution (10 mL) was added, and the mixture was washed with three ethyl acetate portions (10 mL each). The combined organic layers were dried over anhydrous magnesium sulfate, filtered through cotton wool to remove the drying agent and the organic layer was concentrated *in vacuo*. Upon completion of the reaction, an aliquot was removed from the reaction mixture and added to an NMR tube along with $CDCl_3$ (0.6 mL). The mixture was analysed by ^{31}P NMR spectroscopy and an approximate ^{31}P NMR spectral yield was determined using the method proposed by Montchamp and co-workers.[7] The ^{31}P NMR spectral yield of a given phosphorus-containing compound was obtained by expressing the integration of the signal of that compound in the ^{31}P NMR spectrum of the reaction mixture as a percentage of the sum of the integrations of all of the signals present in the ^{31}P NMR spectrum.

Since Ph_3PO is formed during the hydrolytic process that releases the ether from (α-alkoxyalkyl)phosphonium salt **10D**, the amount of Ph_3PO generated was used as an indicator of the amount of ether formed. The signal of Ph_3PO from the ^{31}P NMR spectra of the reaction mixtures used for ^{31}P NMR spectral yield determination appeared at δ_P *ca.* 29 ppm,[8] while the signal of Ph_3P used appeared at δ_P *ca.* −5 ppm[9] (in $CDCl_3$ solvent containing MeCN solvent from the reaction mixture). In each case, the precise chemical shifts of these signals did vary from experiment to experiment due to differences in the sample composition.

The reactions in Table S5 were conducted according to General Procedure B. An estimated yield for each reaction was determined using the ^{31}P NMR spectroscopic method described above in General Procedure B. The first experiment aimed at hydrolysis of (α-alkoxyalkyl)phosphonium salt **10D** involved addition of 2 mol L^{-1} of NaOH to the reaction mixture (Table S5, Entry 1). This produced a significant reformation of Ph_3P, which can be rationalised as arising from C-attack by hydroxide rather than the desired P-attack which leads to Ph_3PO formation and thus benzyl ether formation. The hydrolysis was repeated at varying temperatures in MeCN (Table S5, Entries 3 – 5). These experiments showed that lower temperatures favour P-attack over C-attack. With this observation in mind, hydrolysis was also attempted in the solvents that facilitated the formation of **10D**. However, in these solvents, phosphine oxide was the minor product. Instead, the preferred reaction was C-attack by hydroxide, which resulted in regeneration of starting materials (Table S5, Entries 8 – 12). The concentration of NaOH could be significantly reduced from 6 moles to 2.8 moles to achieve complete consumption of **10D** in 1.5 hrs at 20 °C (Table S5, Entry 7).

Table S5: Hydrolysis optimisation for formation of benzyl ether and triphenylphosphine oxide

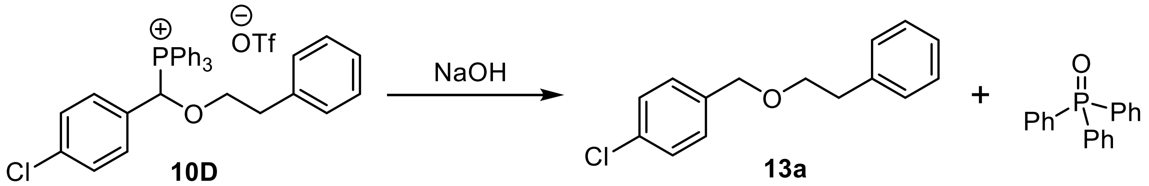

Entry	Solvent	Temp. (°C)	Time (hrs)	NaOH		% Formed [a]		
				Conc. (mol L^{-1})	Volume (mL)	Ph_3PO	**10D**	Ph_3P
1	MeCN	45 [b]	24	3	2	69	0	31
2	Toluene	100 [b]	24	3	2	60	0	40
3	MeCN/Toluene (2:1)	60 [b]	24	3	2	7	0	93
4	MeCN	50 [b]	4	3	2	58	0	42
5	MeCN	20	1.5	3	2	85	0	14
6	MeCN	20	1.5	1	2	91	0	9
**7**	**MeCN**	**20**	**1.5**	**2**	**1.4**	**95**	**0**	**5**
8	$CHCl_3$	20	1.5	2	1.4	12	60	25
9	CH_2Cl_2	20	1.5	2	1.4	7	68	24
10	EtOAc	20	1.5	2	1.4	9	7	83
11	Acetone	20	1.5	2	1.4	44	0	55
12	THF	20	1.5	2	1.4	19	79	2

[a] The quantity of product formed was determined from relative integrations in ^{31}P NMR spectra of the reaction mixtures.
[b] Heating bath temperature.

5. Substrate Scope for Hydrolytic Etherification of Aldehydes & Alcohols

General Procedure C: Acetonitrile (2.0 mL) was added to a round bottom flask that was equipped with a stirring bar. To this, the following were added sequentially: triphenylphosphine (288 mg, 1.10 mmol, 1.10 equivalents), trifluoromethanesulfonic acid (180 mg, 1.20 mmol, 1.20 equivalents) and aldehyde substrate (1.0 mmol, 1.0 equivalent) and the contents of the flask were stirred at room temperature. The alcohol substrate (1.9 mmol, 1.9 equivalents) was then added and the flask was sealed with a plastic lid. The flask was placed in an oil bath set at 45 °C for 10 hours. After this time, the flask was removed from the oil bath, and the reaction mixture was allowed to cool to room temperature. A 2 mol L^{-1} solution of NaOH (1.4 mL, 2.8 mmol, 2.8 equivalents) was added to the flask and it was stirred at room temperature for 2 hours. Upon completion of the reaction, the mixture was diluted in ethyl acetate (10 mL) and extracted with a saturated brine solution (10 mL). The aqueous layer was extracted twice more with 10 mL portions of ethyl acetate (2 × 10 mL). The organic phases were combined and dried over anhydrous magnesium sulfate, filtered and concentrated *in vacuo*.

The solution was worked up as per the process outlined in *Org. Process Res. Dev*. **2017**, 21, 1394−1403.[10] The residue was taken up in methanol (5 mL) and added to a separatory funnel along with a saturated sodium bisulfite solution (3 mL). The separatory funnel was vigorously shaken for approximately 30 s and H_2O (25 mL) was added. The mixture was extracted with 90:10 cyclohexane/ethyl acetate (25 mL). The aqueous layer was extracted twice more with 90:10 cyclohexane/ethyl acetate (2 × 25 mL). The combined organic layers were dried over anhydrous magnesium sulfate, filtered and concentrated *in vacuo*. Unless otherwise stated, the residual material remaining after solvent removal was purified by flash column chromatography on silica (SiO_2) to give the ether product.

(i) Synthesis of compound 13a

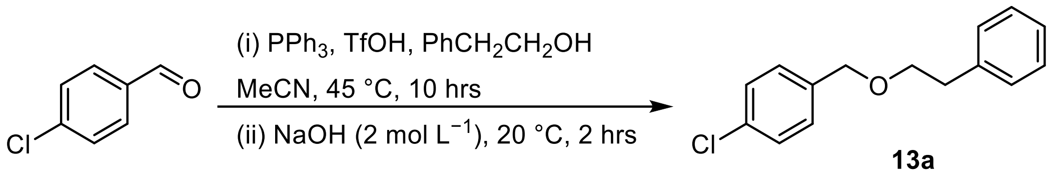

13a

According to General Procedure C, using 4-chlorobenzaldehyde (141 mg, 1.00 mmol, 1.00 equivalent), triphenylphosphine (291 mg, 1.11 mmol, 1.11 equivalents) and trifluoromethanesulfonic acid (187 mg, 1.20 mmol, 1.20 equivalents) in acetonitrile (2.0 mL), followed by addition of 2-phenyl-1-ethanol (244 mg, 2.00 mmol, 2.00 equivalents). The reaction mixture was stirred at 45 °C for 10 hrs. After the flask was cooled to room temperature, a 2 mol L^{-1} NaOH solution (1.4 mL, 2.8 mmol, 2.8 equivalents) was added and the reaction mixture was stirred for 2 hrs. After work-up, the compound was purified by flash column chromatography (95:5 cyclohexane/EtOAc) to give the product as a colourless oil (224 mg, 0.91 mmol, 91%).

$\mathbf{R}_f$ = 0.4.

^{1}H NMR (300 MHz, $CDCl_3$) δ 7.35 – 7.25 (m, 4H), 7.25 – 7.15 (m, 5H), 4.47 (s, 2H), 3.67 (t, *J* = 7.2 Hz, 2H), 2.92 (t, *J* = 7.2 Hz, 2H).[11]

(ii) Synthesis of compound 13b

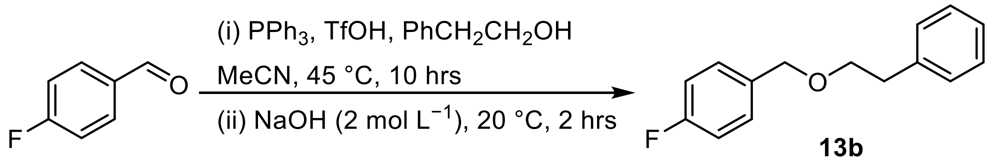

13b

According to General Procedure C, using 4-fluorobenzaldehyde (1.05 g, 8.46 mmol, 1.00 equivalent) was added to acetonitrile (16 mL), triphenylphosphine (2.31 g, 8.81 mmol, 1.04 equivalents) and trifluoromethanesulfonic acid (1.48 g, 9.86 mmol, 1.16 equivalents), followed by addition of 2-phenyl-1-ethanol (1.89 g, 15.2 mmol, 1.82 equivalents). The reaction mixture was stirred at 45 °C for 10 hrs. After the flask was cooled to room temperature, a 2 mol L^{-1} NaOH solution (11 mL, 22 mmol, 2.8 equivalents) was added and the reaction mixture was stirred for 2 hrs. After work-up, the compound was purified by flash column chromatography (96:4 cyclohexane/EtOAc) to give the product as a colourless oil (1.80 g, 7.80 mmol, 92%).

$\mathbf{R}_f$ = 0.33.

IR $\tilde{\nu}_{max}$ (ATR): 2858 (C—H stretch), 1451 (C—H stretch), 1137 (C—O stretch), 1101 (C—F stretch), 1652 (C=C stretch) cm^{-1}.

GC-HRMS (Electron Ionisation): Calculated for $[M]^+ = [C_{15}H_{15}OF]^+$ *m/z* = 230.1106; found *m/z* = 231.1107.

^{1}H NMR (400 MHz, $CDCl_3$) δ 7.38 – 7.15 (m, 6H), 7.11 – 6.89 (m, 3H), 4.51 (s, 2H), 3.70 (t, *J* = 7.1 Hz, 2H), 2.94 (t, *J* = 7.1 Hz, 2H).

^{19}F NMR (376 MHz, $CDCl_3$) δ −113.2.

^{13}C NMR (101 MHz, $CDCl_3$) δ 163.1 (d, ^1J = 246 Hz), 141.3 (d, ^4J = 7 Hz), 138.9, 129.9 (d, ^3J = 8 Hz), 129.1, 128.5, 126.4, 114.5 (d, ^2J = 21 Hz), 72.3 (d, ^5J = 2 Hz), 71.5, 36.5.

(iii) Synthesis of compound 13c

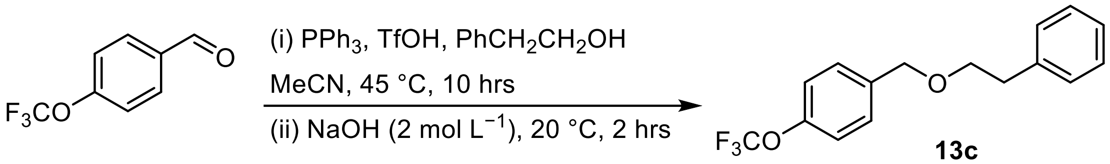

According to General Procedure C, using 4-trifluoromethoxybenzaldehyde (206 mg, 1.00 mmol, 1.00 equivalent), triphenylphosphine (289 mg, 1.10 mmol, 1.10 equivalents) and trifluoromethanesulfonic acid (200 mg, 1.20 mmol, 1.20 equivalents) in acetonitrile (2.0 mL), followed by addition of 2-phenyl-1-ethanol (232 mg, 1.90 mmol, 1.90 equivalents). The reaction mixture was stirred at 45 °C for 10 hrs. After the flask was cooled to room temperature, a 2 mol L^{-1} NaOH solution (1.4 mL, 2.8 mmol, 2.8 equivalents) was added and the reaction mixture was stirred for 2 hrs. The compound was purified by flash column chromatography (95:5 cyclohexane/EtOAc) to give the product as a colourless oil (264 mg, 0.890 mmol, 89%).

R$_f$ = 0.33

IR $\tilde{\nu}_{max}$ (ATR): 2858 (C—H stretch), 1454 (C—H stretch), 1155 (C—O stretch), 1101 (C—F stretch) cm^{-1}.

GC-HRMS (Electron Ionisation): Calculated for $[M]^+ = [C_{16}H_{15}F_3O_2]^+$ *m/z* = 296.1024; found *m/z* = 296.1022.

^{1}H NMR (400 MHz $CDCl_3$) δ 7.37 – 7.27 (m, 4H), 7.24 (d, *J* = 7.7 Hz, 3H), 7.18 (d, *J* = 7.7 Hz, 2H), 4.52 (s, 2H), 3.72 (t, *J* = 7.1 Hz, 2H), 2.95 (t, *J* = 7.1 Hz, 2H).

^{19}F NMR (376 MHz, $CDCl_3$) δ −57.7.

^{13}C NMR (101 MHz, $CDCl_3$) δ 148.6 (q, *J* = 2 Hz), 138.9, 137.3, 129.0, 128.9, 128.4, 126.3, 121.0, 120.5 (q, *J* = 256 Hz), 72.1, 71.5, 36.4.

(iv) Synthesis of compound 13d

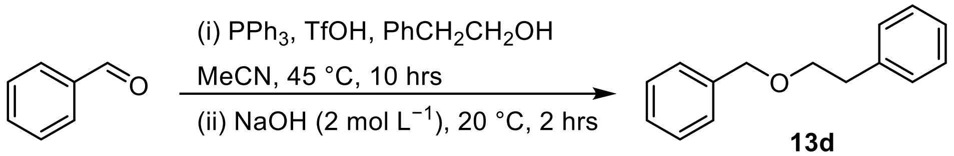

13d

According to General Procedure C, using benzaldehyde (112 mg, 1.00 mmol, 1.00 equivalent), triphenylphosphine (288 mg, 1.1 mmol, 1.1 equivalents) and trifluoromethanesulfonic acid (187 mg, 1.20 mmol, 1.20 equivalents) in acetonitrile (2.0 mL), followed by addition of 2-phenyl-1-ethanol (255 mg, 1.90 mmol, 1.90 equivalents). The reaction mixture was stirred at 45 °C for 10 hrs. After the flask was cooled to room temperature, a 2 mol L^{-1} NaOH solution (1.4 mL, 2.8 mmol, 2.8 equivalents) was added and the reaction mixture was stirred for 2 hrs. After work-up, the compound was purified by flash column chromatography (97:3 cyclohexane/EtOAc) to give the product as a colourless oil (186 mg, 0.880 mmol, 88%).

R*f* = 0.25.

^{1}H NMR (400 MHz, $CDCl_3$) δ 7.39 – 7.20 (m, 10H), 4.55 (s, 2H), 3.72 (t, *J* = 7.2 Hz, 2H), 2.96 (t, *J* = 7.2 Hz, 2H).[12]

(v) Synthesis of compound 13e

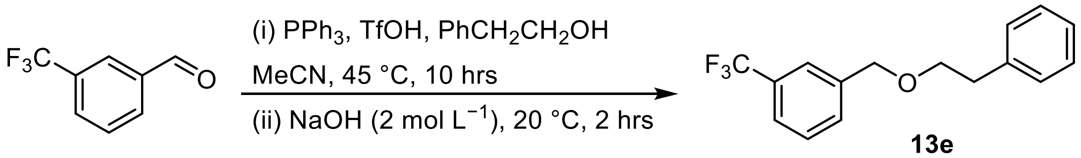

13e

According to General Procedure C, using 3-(trifluoromethyl)benzaldehyde (186 mg, 1.00 mmol, 1.00 equivalent), triphenylphosphine (289 mg, 1.10 mmol, 1.10 equivalents) and trifluoromethanesulfonic acid (202 mg, 1.20 mmol, 1.20 equivalents) in acetonitrile (2.0 mL), followed by addition of 2-phenyl-1-ethanol (263 mg, 1.90 mmol, 1.90 equivalents). The reaction mixture was stirred at 45 °C for 10 hrs. After the flask was cooled to room temperature, a 2 mol L^{-1} NaOH solution (1.4 mL, 2.8 mmol, 2.8 equivalents) was added and the reaction mixture was stirred for 2 hrs. After work-up, the compound was purified by flash column chromatography (95:5 cyclohexane/EtOAc) to give the product as a colourless oil (211 mg, 0.752 mmol, 75%).

R*f* = 0.33

^{1}H NMR (300 MHz, $CDCl_3$) δ 7.61 – 7.49 (m, 2H), 7.49 – 7.37 (m, 2H), 7.36 – 7.14 (m, 5H), 4.55 (s, 2H), 3.72 (t, *J* = 7.0 Hz, 2H), 2.94 (t, *J* = 7.0 Hz, 2H).[c,13]

[c] The signal at δ 7.61 – 7.49 ppm (m, 2H) is reported as δ 7.56 ppm (s, 1H) and δ 7.52 ppm (d, *J* = 7.0 Hz, 1H) in reference 11. However, in our spectrum of this compound, the signals are not baseline-separated.

(vi) Synthesis of compound 13f

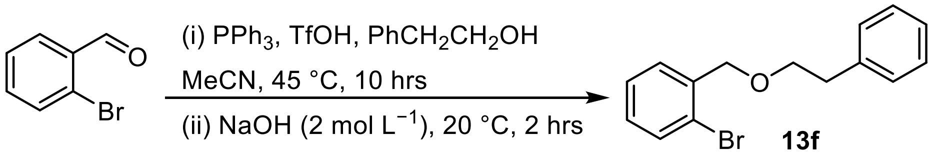

According to General Procedure C, using 2-bromobenzaldehyde (199 mg, 1.07 mmol, 1.07 equivalent), triphenylphosphine (290 mg, 1.10 mmol, 1.10 equivalents) and trifluoromethanesulfonic acid (196 mg, 1.30 mmol, 1.30 equivalents) in acetonitrile (2.0 mL), followed by addition of 2-phenyl-1-ethanol (254 mg, 2.07 mmol, 2.07 equivalents). The reaction mixture was stirred at 45 °C for 10 hrs. After the flask was cooled to room temperature, a 2 mol L^{-1} NaOH solution (1.4 mL, 2.8 mmol, 2.8 equivalents) was added and the reaction mixture was stirred for 2 hrs. After work-up, the compound was purified by flash column chromatography (95:5 cyclohexane/EtOAc) to give the product as a colourless oil (247 mg, 0.85 mmol, 85%).

$\mathbf{R}_f$ = 0.38

^{1}**H NMR** (300 MHz, $CDCl_3$) δ 7.51 (dd, *J* = 8.0, 1.3 Hz, 1H), 7.44 – 7.36 (m, 1H), 7.35 – 7.17 (m, 6H), 7.11 (td, *J* = 8.0, 1.8 Hz 1H), 4.58 (s, 2H), 3.76 (t, *J* = 7.1 Hz, 2H), 2.97 (t, *J* = 7.1 Hz, 2H).[d,14]

(vii) Synthesis of compound 14a

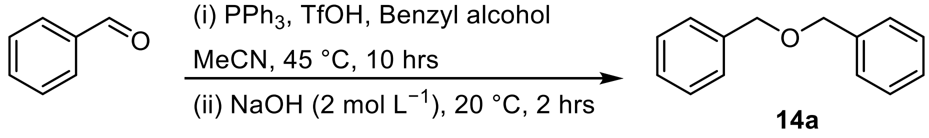

According to General Procedure C, using benzaldehyde (104 mg, 0.980 mmol, 0.980 equivalent), triphenylphosphine (292 mg, 1.11 mmol, 1.11 equivalents) and trifluoromethanesulfonic acid (192 mg, 1.28 mmol, 1.28 equivalents) in acetonitrile (2.0 mL), followed by addition of benzyl alcohol (218 mg, 2.01 mmol, 2.01 equivalents). The reaction mixture was stirred at 45 °C for 10 hrs. After the flask was cooled to room temperature, a 2 mol L^{-1} NaOH solution (1.4 mL, 2.8 mmol, 2.8 equivalents) was added and the reaction mixture was stirred for 2 hrs. After work-up, the compound was purified by flash column chromatography (98:2 cyclohexane/EtOAc) to give the product as a colourless oil (172 mg, 0.870 mmol, 87%).

[d] The signal at δ 7.44 – 7.36 ppm (m, 1H) is reported as δ 7.42 ppm (ddt, *J* = 7.7, 1.8, 0.9 Hz, 1H) and the signal at δ 7.11 ppm (td, *J* = 8.0, 1.8 Hz, 1H) is reported as δ 7.14 ppm (dddt, *J* = 8.0, 7.4, 1.8, 0.6 Hz, 1H) in reference 12. However, in the spectrum we obtained for this compound such multiplicities are not observed.

R*f* = 0.63

¹H NMR (400 MHz, $CDCl_3$) δ 7.55 – 7.15 (m, 10H), 4.59 (s, 4H).[e, 15]

(viii) Synthesis of compound 14b

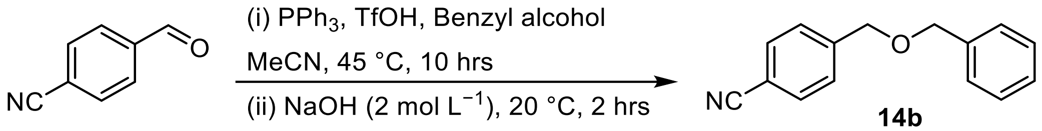

According to General Procedure C, using 4-cyanobenzaldehyde (128 mg, 0.977 mmol, 0.977 equivalent), triphenylphosphine (291 mg, 1.11 mmol, 1.11 equivalents) and trifluoromethanesulfonic acid (191 mg, 1.27 mmol, 1.27 equivalents) in acetonitrile (2.0 mL), followed by addition of benzyl alcohol (229 mg, 2.0 mmol, 2.0 equivalents). The reaction mixture was stirred at 45 °C for 10 hrs. After the flask was cooled to room temperature, a 2 mol L^{-1} NaOH solution (1.4 mL, 2.8 mmol, 2.8 equivalents) was added and the reaction mixture was stirred for 2 hrs. After work-up, the compound was purified by flash column chromatography (97:3 cyclohexane/EtOAc) to give the product as a colourless oil (170 mg, 0.762 mmol, 76%).

R*f* = 0.22

¹H NMR (300 MHz, $CDCl_3$) δ 7.69 – 7.59 (m, 2H), 7.50 – 7.43 (m, 2H), 7.42 – 7.25 (m, 5H), 4.60 (s, 4H).[f, 16]

(ix) Synthesis of compound 14c

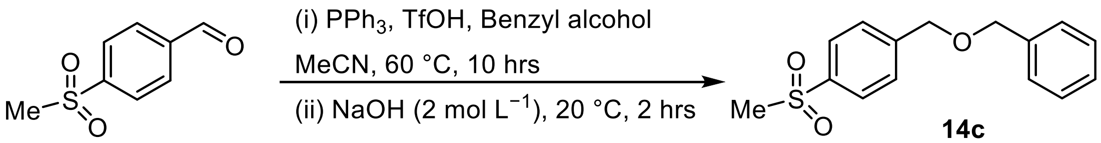

According to General Procedure C, using 4-(methylsulfonyl)benzaldehyde (188 mg, 1.02 mmol, 1.02 equivalent), triphenylphosphine (289 mg, 1.10 mmol, 1.10 equivalents) and trifluoromethanesulfonic acid (190 mg, 1.26 mmol, 1.26 equivalents) in acetonitrile (2.0 mL), followed by addition of benzyl alcohol (221 mg, 2.04 mmol, 2.04 equivalents). The reaction mixture was stirred at 60 °C for 10 hrs. After the flask was cooled to room temperature, a 2 mol L^{-1} NaOH solution (1.4 mL, 2.8 mmol, 2.8

[e] The signal at δ 7.55 – 7.15 ppm (m, 10H) is reported as δ 7.36 – 7.31 (m, 8H) and δ 7.28 – 7.24 (m, 2H) in reference 13. However, in the spectrum we obtained for this compound the signals are not baseline separated.

[f] The signals at δ 7.69 – 7.59 (m, 2H) and δ 7.50 – 7.43 (m, 2H) are reported as δ 7.65 (d, J = 8 Hz, 2H) and δ 7.48 (d, J = 8 Hz, 2H) in reference 14. However, in the spectrum we obtained for this compound the signals have finer multiplicity.

equivalents) was added and the reaction mixture was stirred for 2 hrs. After work-up, the compound was purified by flash column chromatography (80:20 cyclohexane/EtOAc) to give the product as a colourless oil (173 mg, 0.630 mmol, 63%).

R*f* = 0.18

IR $\tilde{\nu}_{max}$ (ATR): 2925 (C—H stretch), 1299 (S=O stretch), 1145 (S=O stretch), 1086 (C—O stretch) cm^{-1}.

HRMS (ESI+): Calculated for $[M + H]^+ = [C_{15}H_{17}O_3S]^+$ *m/z* = 277.0892; found *m/z* = 277.0899

^{1}H NMR (500 MHz, $CDCl_3$) δ 7.94 – 7.90 (m, 2H), 7.58 – 7.54 (m, 2H), 7.43 – 7.27 (m, 5H), 4.63 (s, 2H), 4.61 (s, 2H), 3.04 (s, 3H).

^{13}C NMR (126 MHz, CDCl3) δ 144.9, 139.7, 137.7, 128.6, 128.1, 128.0, 127.8, 127.5, 72.8, 71.0, 44.6.

(x) Synthesis of compound 14d

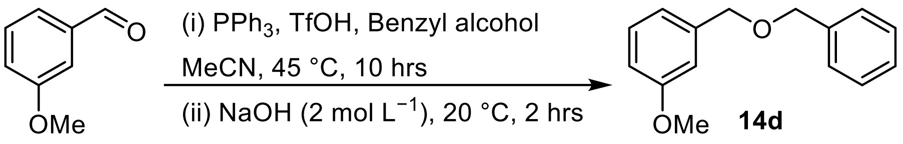

According to General Procedure C, using 3-methoxybenzaldehyde (135 mg, 0.992 mmol, 0.992 equivalent), triphenylphosphine (291 mg, 1.11 mmol, 1.11 equivalents) and trifluoromethanesulfonic acid (198 mg, 1.32 mmol, 1.32 equivalents) in acetonitrile (2.0 ml), followed by addition of benzyl alcohol (213 mg, 1.97 mmol, 1.97 equivalents). The reaction mixture was stirred at 45 °C for 10 hrs. After the flask was cooled to room temperature, a 2 mol L^{-1} NaOH solution (1.4 mL, 2.8 mmol, 2.8 equivalents) was added and the flask reaction mixture for 2 hrs. After work-up, the compound was purified by flash column chromatography (96:4 cyclohexane/EtOAc) to give the product as a colourless oil (164 mg, 0.720 mmol, 72%).

R*f* = 0.3

^{1}H NMR (300 MHz, $CDCl_3$) δ 7.48 – 7.16 (m, 6H), 6.97 – 6.90 (m, 2H), 6.89 – 6.76 (m, 1H), 4.55 (s, 2H), 4.53 (s, 2H), 3.79 (s, 3H).[g, 17]

[g] The signal at 7.48 – 7.16 (m, 6H) is reported as 7.40 – 7.33 (m, 4H) and 7.33 – 7.27 (m, 2H) in reference 15. However, in the spectrum we obtained for this compound, the signals are not baseline separated.

(xi) Synthesis of compound 14e

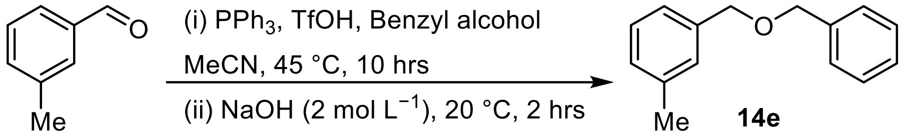

14e

According to General Procedure C, using 3-methylbenzaldehyde (121 mg, 1.00 mmol, 1.00 equivalent), triphenylphosphine (289 mg, 1.1 mmol, 1.1 equivalents) and trifluoromethanesulfonic acid (198 mg, 1.32 mmol, 1.32 equivalents) in acetonitrile (2.0 mL), followed by addition of benzyl alcohol (211 mg, 1.95 mmol, 1.95 equivalents). The reaction mixture was stirred at 45 °C for 10 hrs. After the flask was cooled to room temperature, a 2 mol L^{-1} NaOH solution (1.4 mL, 2.8 mmol, 2.8 equivalents) was added and the flask reaction mixture for 2 hrs. After work-up, the compound was purified by flash column chromatography (96:4 cyclohexane/EtOAc) to give the product as a colourless oil (139 mg, 0.655 mmol, 65%).

$\mathbf{R}_f$ = 0.33

^{1}H NMR (300 MHz, $CDCl_3$) δ 7.49 – 7.00 (m, 9H), 4.58 (s, 2H), 4.55 (s, 2H), 2.38 (s, 3H).[18]

(xii) Synthesis of compound 14f

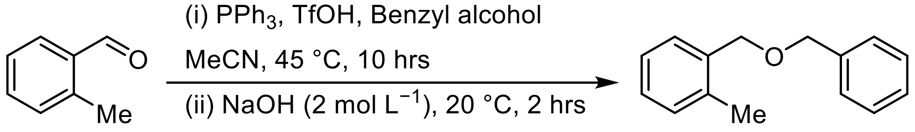

14f

According to General Procedure C, using 2-methylbenzaldehyde (124 mg, 1.03 mmol, 1.03 equivalent), triphenylphosphine (288 mg, 1.09 mmol, 1.09 equivalents) and trifluoromethanesulfonic acid (185 mg, 1.23 mmol, 1.23 equivalents) in acetonitrile (2.0 mL), followed by addition of benzyl alcohol (229 mg, 2.12 mmol, 12.12 equivalents). The reaction mixture was stirred at 45 °C for 10 hrs. After the flask was cooled to room temperature, a 2 mol L^{-1} NaOH solution (1.4 mL, 2.8 mmol, 2.8 equivalents) was added and the reaction mixture was stirred for 2 hrs. After work-up, the compound was purified by flash column chromatography (96:4 cyclohexane/EtOAc) to give the product as a colourless oil (146 mg, 0.690 mmol, 69%).

$\mathbf{R}_f$ = 0.25

^{1}H NMR (300 MHz, $CDCl_3$) δ 7.42 – 7.24 (m, 6H), 7.22 – 7.12 (m, 3H), 4.56 (s, 2H), 4.54 (s, 2H), 2.32 (s, 3H).[h, 19]

---

[h] The signal at δ 7.42 – 7.24 ppm (m, 6H) is reported as δ 7.41 – 7.37 ppm (m, 5H) and δ 7.34 – 7.32 ppm (m, 1H) in reference 17. However, in our spectrum for this compound, the signals are not baseline separated.

(xiii) Synthesis of compound 14g

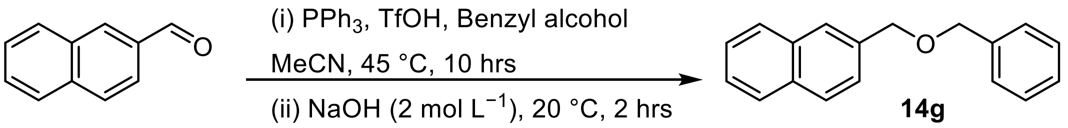

According to General Procedure C, using 2-naphthaldehyde (163 mg, 1.04 mmol, 1.04 equivalents), Ph_3P (294 mg, 1.21 mmol, 1.21 equivalents) and trifluoromethanesulfonic acid (202 mg, 1.34 mmol, 1.34 equivalents) in acetonitrile (2.0 mL), followed by addition of benzyl alcohol (224 mg, 2.07 mmol, 2.07 equivalents). The reaction mixture was stirred at 45 °C for 10 hrs. After the flask was cooled to room temperature, a 2 mol L^{-1} NaOH solution (1.4 mL, 2.8 mmol, 2.8 equivalents) was added and the reaction mixture was stirred for 2 hrs. After work-up, the compound was purified by flash column chromatography (96:4 cyclohexane/EtOAc) to give the product as a colourless oil (207 mg, 0.834 mmol, 83%).

$\mathbf{R}_f$ = 0.3

^{1}H NMR (300 MHz, $CDCl_3$) δ 7.96 – 7.72 (m, 4H), 7.55 – 7.42 (m, 3H), 7.42 – 7.25 (m, 5H), 4.72 (s, 2H), 4.60 (s, 2H).[i, 20]

(xiv) Synthesis of compound 14h

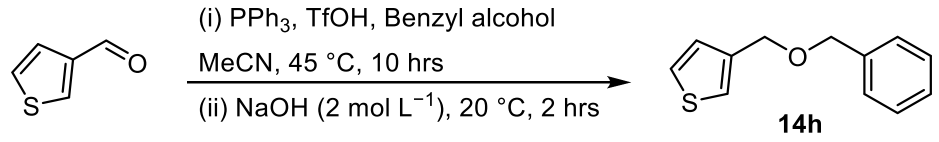

According to General Procedure C, using 3-thiophene carboxaldehyde (123 mg, 1.09 mmol, 1.09 equivalent), triphenylphosphine (293, 1.11 mmol, 1.11 equivalents) and trifluoromethanesulfonic acid (203 mg, 1.35 mmol, 1.35 equivalents) in acetonitrile (2.0 mL), followed by addition of benzyl alcohol (211 mg, 1.96 mmol, 1.96 equivalents). The reaction mixture was stirred at 45 °C for 10 hrs. After the flask was cooled to room temperature, a 2 mol L^{-1} NaOH solution (1.4 mL, 2.8 mmol, 2.8 equivalents) was added and the reaction mixture was stirred for 2 hrs. After work-up, the compound was purified by flash column chromatography (95:5 cyclohexane/EtOAc) to give the product as a colourless oil (141 mg, 0.691 mmol, 69%).

$\mathbf{R}_f$ = 0.25

[i] The signal at δ 7.55 – 7.42 ppm (m, 3H) is reported as δ 7.59 – 7.55 ppm (m, 1H) and δ 7.55 – 7.32 ppm (m, 1H) in reference 18. However, in our spectrum for this compound, the signals are not baseline separated.

^{1}**H NMR** (300 MHz, $CDCl_3$) δ 7.44 – 7.20 (m, 6H), 7.04 – 6.92 (m, 2H), 4.70 (d, *J* = 0.7 Hz ,2H), 4.55 (s, 2H).[j, 21]

(xv) Synthesis of compound 14i

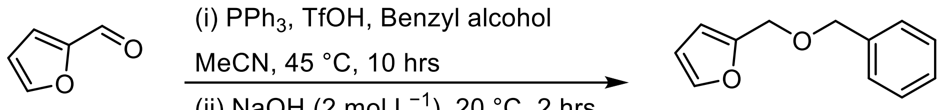

14i

According to General Procedure C, using 2-furancarboxaldehyde (96 mg, 1.00 mmol, 1.0 equivalent), triphenylphosphine (291, 1.11 mmol, 1.11 equivalents) and trifluoromethanesulfonic acid (192 mg, 1.28 mmol, 1.28 equivalents) in acetonitrile (2.0 mL), followed by addition of benzyl alcohol (217 mg, 2.00 mmol, 2.00 equivalents). The reaction mixture was stirred at 45 °C for 10 hrs. After the flask was cooled to room temperature, a 2 mol L^{-1} NaOH solution (1.4 mL, 2.8 mmol, 2.8 equivalents) was added and the reaction mixture was stirred for 2 hrs. After work-up, the compound was purified by flash column chromatography (96:4 cyclohexane/EtOAc) to give the product as a colourless oil (133 mg, 0.712 mmol, 71%).

$\mathbf{R}_f$ = 0.2

^{1}**H NMR** (300 MHz, $CDCl_3$) δ 7.41 (dd, *J* = 1.8, 0.9 Hz, 1H), 7.39 – 7.24 (m, 5H), 6.33 – 6.29 (m, 2H), 4.55 (s, 2H), 4.48 (s, 2H).[22]

---

[j] The signals at δ 7.44 – 7.20 ppm (m, 6H) and δ 7.04 – 6.92 ppm (m, 2H) is reported as δ 7.40 – 7.15 ppm (m, 7H) and δ 7.10 – 6.95 ppm (m, 1H) in reference 19. However, in the spectrum we obtained for this compound, the signals are baseline separated.

(xvi) Synthesis of compound 14j

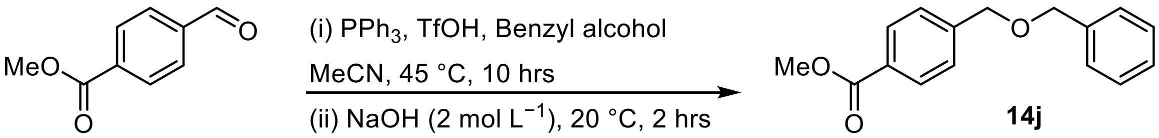

According to General Procedure C, using methyl 4-formylbenzoate (166 mg, 1.01 mmol, 1.01 equivalent), triphenylphosphine (290, 1.10 mmol, 1.10 equivalents) and trifluoromethanesulfonic acid (189 mg, 1.26 mmol, 1.26 equivalents) in acetonitrile (2.0 mL), followed by addition of benzyl alcohol (214 mg, 1.98 mmol, 1.98 equivalents). The reaction mixture was stirred at 45 °C for 10 hrs. After the flask was cooled to room temperature, a 2 mol L^{-1} NaOH solution (0.7 mL, 1.4 mmol, 1.4 equivalents) was added and the reaction mixture was stirred for 2 hrs. After work-up, the compound was purified by flash column chromatography (96:4 cyclohexane/EtOAc) to give the product as a colourless oil (193 mg, 0.753 mmol, 75%).

$\mathbf{R}_f$ = 0.22

^{1}H NMR (300 MHz, $CDCl_3$) δ 8.05 – 8.02 (m, 2H), 7.46 – 7.43 (m, 2H), 7.38 – 7.32 (m, 5H), 4.60 (s, 2H), 4.58 (s, 2H), 3.91 (s, 3H).[k, 18]

(xvii) Synthesis of compound 14k

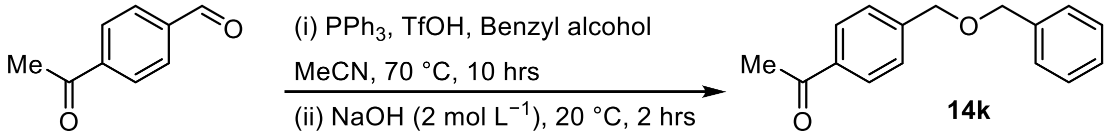

According to General Procedure C, using 4-acetylbenzaldehyde (151 mg, 1.02 mmol, 1.02 equivalent) was added to acetonitrile (1.5 mL), triphenylphosphine (289 mg, 1.10 mmol, 1.10 equivalents) and trifluoromethanesulfonic acid (193 mg, 1.28 mmol, 1.28 equivalents) in acetonitrile (2.0 mL), followed by addition of benzyl alcohol (204 mg, 1.88 mmol, 1.88 equivalents). The reaction mixture was stirred at 70 °C for 10 hrs. After the flask was cooled to room temperature, a 2 mol L^{-1} NaOH solution (1.4 mL, 2.8 mmol, 2.8 equivalents) was added and the reaction mixture was stirred for 2 hrs. After work-up, the compound was purified by flash column chromatography (85:15 cyclohexane/EtOAc) to give the product as a colourless oil (187 mg, 0.779 mmol, 77%).

$\mathbf{R}_f$ = 0.33

---

[k] The signals at δ 8.05 – 8.02 ppm (m, 2H) and δ 7.46 – 7.43 ppm (m, 2H) are reported as δ 8.05 ppm (d, J = 8.2 Hz, 2H), δ 7.45 ppm (d, J = 8.0 Hz, 2H) in reference 18. In our spectrum, the signals exhibit more complex splitting. The signals at δ 7.38 – 7.32 ppm (m, 5H) is reported as δ 7.41 – 7.35 (m, 4H) and δ 7.35 – 7.29 (m, 1H) in reference 16. However, in our spectrum for this compound, the signals are not baseline separated.

^{1}H NMR (300 MHz, $CDCl_3$) δ 7.97 – 7.94 (m, 2H), 7.48 – 7.45 (m, 2H), 7.42 – 7.27 (m, 5H), 4.61 (s, 2H), 4.59 (s, 2H), 2.60 (s, 3H).[l, 17]

(xviii) Synthesis of compound 14l

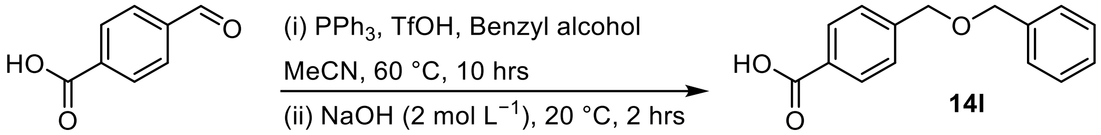

Acetonitrile (2.0 mL) was added to a round bottom flask that was equipped with a stirring bar. To this, the following were added sequentially: triphenylphosphine (288 mg, 1.09 mmol, 1.09 equivalent), trifluoromethanesulfonic acid (182 mg, 1.21 mmol, 1.21 equivalent) and 4-formyl benzoic acid (151 mg, 1.00 mmol, 1.00 equivalent), and the solution was stirred at room temperature. Benzyl alcohol (215 mg, 1.99 mmol, 1.99 equivalents) was then added and the flask was sealed with a plastic lid. The flask was placed in an oil bath set at 60 °C for 10 hours. After this time, the flask was removed from the oil bath, and the reaction mixture was allowed to cool to room temperature. A 2 mol L^{-1} solution of NaOH (2.4 mL, 4.8 mmol, 4.8 equivalent) was added to the flask and it was stirred at room temperature for 2 hours. The reaction mixture was transferred to a separatory funnel. H_2O (10 mL) was added and the reaction mixture was washed with 10mL of EtOAc. The aqueous layer was washed once more with 10 mL of EtOAc and was then neutralised with 1 mol L^{-1} HCl. The aqueous layer was washed three times with 10 mL portions of EtOAc. The separated organic phases were combined and then dried using anhydrous magnesium sulfate and filtered through cotton wool. The organic layer was concentrated *in vacuo* to give a white solid. The solid was dissolved in MeOH (5mL) and saturated sodium bisulfite (3 mL) was added. The solution was worked up as per the process outlined in General Procedure C (taken from *Org. Process Res. Dev.* **2017**, *21*, 1394–1403)[8] and concentrated *in vacuo* to give the product as a white solid (175 mg, 0.723 mmol, 72%).

R_f = 0.30

IR $\tilde{\nu}_{max}$ (ATR): 3064 (O—H stretch), 2852 (C—H stretch), 1681 (C=O stretch), 1425 (C—H stretch), 1182 (C—O stretch) cm^{-1}.

HRMS (ESI–): Calculated for $[M – H]^-$ = $[C_{15}H_{14}O_3]^-$ *m/z* = 242.0942; found *m/z* = 241.0869.

---

[l] The signals at δ 7.97 – 7.94 ppm (m, 2H) and δ 7.48 – 7.45 ppm (m, 2H) are reported as δ 7.96 ppm (d, *J* = 8.4 Hz, 2H) and δ 7.47 ppm (d, *J* = 8.4 Hz, 2H) in reference 17. In our spectrum, the signals exhibit more complex splitting. The signal at δ 7.42 – 7.27 ppm (m, 5H) is reported as δ 7.40 – 7.35 ppm (m, 4H) and δ 7.33-7.30 ppm (m, 1H) in reference 15. However, in the spectrum we obtained for this compound, the signals are not baseline separated.

Melting Point: 147 – 149 °C.

^{1}H NMR (400 MHz, DMSO-d_6) δ 7.90 (d, J = 8.3 Hz, 1H), 7.43 (d, J = 8.3 Hz, 1H), 7.30 (m, 5H), 4.57 (s, 2H), 4.52 (s, 2H).

^{13}C NMR (101 MHz, DMSO-d_6) δ 167.7, 144.1, 138.7, 130.4, 129.9, 128.9, 128.1, 128.1, 127.7, 72.2, 71.4.

(xix) Synthesis of compound 14m

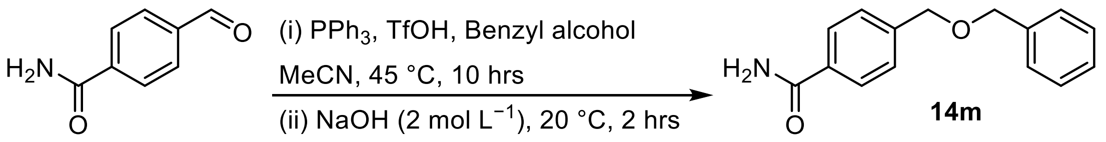

According to General Procedure C, using 4-formylbenzamide (154 mg, 1.03 mmol, 1.03 equivalent) was added to acetonitrile (1.5 mL), triphenylphosphine (291 mg, 1.11 mmol, 1.11 equivalents) and trifluoromethanesulfonic acid (201 mg, 1.34 mmol, 1.34 equivalents) in acetonitrile (2.0 mL), followed by addition of benzyl alcohol (212 mg, 1.96 mmol, 1.96 equivalents) was then added and the reaction mixture was stirred at 45 °C for 10 hrs. After the flask was cooled to room temperature, a 2 mol L^{-1} NaOH solution (1.4 mL, 2.8 mmol, 2.8 equivalents) was added and the reaction mixture was stirred for 2 hrs. After work-up, the compound was purified by flash column chromatography (85:15 cyclohexane/EtOAc) to give the product as a white solid (152 mg, 0.630 mmol, 63%).

R$_f$ = 0.3

IR $\tilde{\nu}_{max}$ (ATR): 3366 (N—H stretch), 2949 (C—H stretch), 1649 (C=O stretch), 1067 (C—O stretch) cm^{-1}.

HRMS (ESI+): Calculated for $[M + H]^+ = [C_{15}H_{15}NO_2]^+$ m/z = 242.1103; found m/z = 242.1175

Melting Point: 134 – 136 °C.

^{1}H NMR (400 MHz, $CDCl_3$) δ 7.79 (d, J = 8.3 Hz, 2H), 7.43 (d, J = 8.3 Hz, 2H), 7.39 – 7.20 (m, 5H), 6.09 (s, 2H), 4.59 (s, 2H), 4.57 (s, 2H).

^{13}C NMR (101 MHz $CDCl_3$) δ 169.3, 142.7, 137.9, 132.6, 128.5, 127.9, 127.8, 127.6, 127.6, 72.5, 71.4.

(xx) Synthesis of compound 15

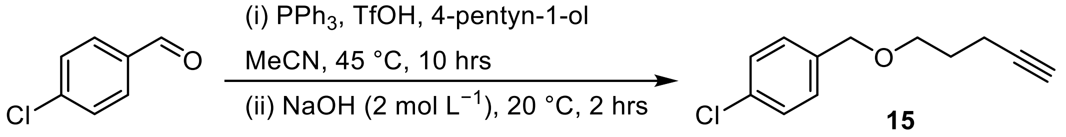

According to General Procedure C, using 4-chlorobenzaldehyde (143 mg, 1.02 mmol, 1.02 equivalent), triphenylphosphine (291, mg 1.11 mmol, 1.11 equivalents) and trifluoromethanesulfonic acid (199 mg, 1.32 mmol, 1.32 equivalents) in acetonitrile (2.0 mL), followed by addition of 4-pentyl-1-ol (163 mg, 1.94 mmol, 1.94 equivalents). The reaction mixture was stirred at 45 °C for 10 hrs. After the flask was cooled to room temperature, a 2 mol L^{-1} NaOH solution (1.4 mL, 2.8 mmol, 2.8 equivalents) was added and the reaction mixture was stirred for 2 hrs. The compound was purified by flash column chromatography (95:5 cyclohexane/EtOAc) to give the product as a colourless oil (153 mg, 0.735 mmol, 73%).

R*f* = 0.38

^{1}H NMR (300 MHz, $CDCl_3$) δ 7.36 – 7.21 (m, 4H), 4.47 (s, 2H), 3.56 (t, *J* = 6.2 Hz, 2H), 2.31 (td, *J* = 7.1, 2.7 Hz, 2H), 1.93 (t, *J* = 2.7 Hz, 1H), 1.89 – 1.75 (m, 2H).[m, 23]

(xxi) Synthesis of compound 16

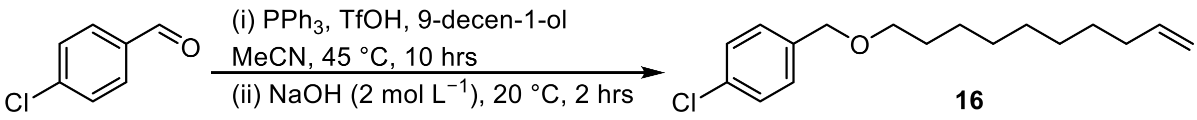

According to General Procedure C, using 4-chlorobenzaldehyde (144 mg, 1.02 mmol, 1.02 equivalent), triphenylphosphine (292 mg, 1.1 mmol, 1.1 equivalents) and trifluoromethanesulfonic acid (195 mg, 1.30 mmol, 1.30 equivalents) in acetonitrile (2.0 mL), followed by addition of 9-decen-1-ol (299 mg, 1.91 mmol, 1.91 equivalents). The reaction mixture was stirred at 45 °C for 10 hrs. After the flask was cooled to room temperature, a 2 mol L^{-1} NaOH solution (1.4 mL, 2.8 mmol, 2.8 equivalents) was added and the reaction mixture was stirred for 2 hrs. The compound was purified by flash column chromatography (95:5 cyclohexane/EtOAc) to give the product as a colourless oil (216 mg, 0.771 mmol, 77%).

R*f* = 0.4

[m] The signals at δ 1.89 – 1.75 ppm (m, 2H) is reported as δ 1.81 (quin, *J* = 6.0 Hz, 2H) in reference 21. However, in the spectrum we obtained for this compound, the signals have finer multiplicity.

IR $\tilde{\nu}_{max}$ (ATR): 2924 (C—H stretch), 2865 (C—H stretch), 1461 (C—H stretch), 1088 (C—O stretch) cm^{-1}.

HRMS (ESI+): Calculated for $[M + Na]^+$ = $[C_{17}H_{25}ClONa]^+$ *m/z* = 303.1486; found *m/z* = 303.1479.

^{1}H NMR (300 MHz, $CDCl_3$) δ 7.37 – 7.17 (m, 4H), 5.81 (ddt, *J* = 16.9, 10.2, 6.8 Hz, 1H), 5.11 – 4.82 (m, 2H), 4.45 (s, 2H), 3.45 (t, *J* = 6.6 Hz, 2H), 2.10 – 1.911 (m, 2H), 1.68 – 1.50 (m, 2H), 1.48 – 1.06 (m, 10H).

^{13}C NMR (126 MHz, $CDCl_3$) δ 139.2, 137.2, 133.2, 128.9, 128.5, 114.1, 72.0, 70.6, 33.8, 29.7, 29.4, 29.4, 29.0, 28.9, 26.2.

(xxii) Synthesis of compound 17

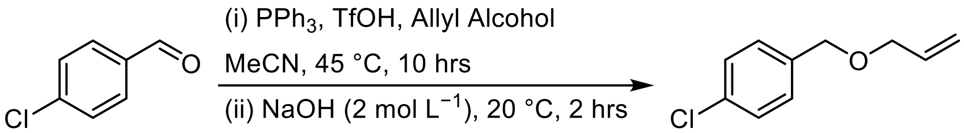

According to General Procedure C, using 4-chlorobenzaldehyde (143 mg, 1.02 mmol, 1.02 equivalent), triphenylphosphine (292 mg, 1.11 mmol, 1.11 equivalents) and trifluoromethanesulfonic acid (194 mg, 1.29 mmol, 1.29 equivalents) in acetonitrile (2.0 mL), followed by addition of allyl alcohol (115 mg, 1.98 mmol, 1.98 equivalents). The reaction mixture was stirred at 45 °C for 10 hrs. After the flask was cooled to room temperature, a 2 mol L^{-1} NaOH solution (1.4 mL, 2.8 mmol, 2.8 equivalents) was added and the reaction mixture was stirred for 2 hrs. The compound was purified by flash column chromatography (95:5 cyclohexane/EtOAc) to give the product as a colourless oil (154 mg, 0.846 mmol, 84%).

R_f = 0.40

^{1}H NMR (300 MHz, $CDCl_3$) δ 7.40 – 7.28 (m, 4H), 6.11 – 5.77 (m, 1H), 5.31 (m (appears as distorted dq, with app ^3J = 17.2 Hz),[n] 1H), 5.21 (m (appears as distorted dq, with ^3J = 10.3 Hz),[n] 1H), 4.49 (s, 2H), 4.02 (dt, *J* = 5.7, 1.5 Hz, 2H).[o, 24]

---

[n] The signal resolution is not sufficient to allow determination of ^2J and ^4J.

[o] The signals at δ 5.39 – 5.14 ppm (m, 2H) are reported as 5.31 (dq, *J* = 17.2, 1.6 Hz, 1H), 5.22 (dm, *J* = 10.6 Hz, 1H) reference 22. However, in the spectrum we obtained for this compound, more defined splitting is observed.

(xxiii) Synthesis of compound 18

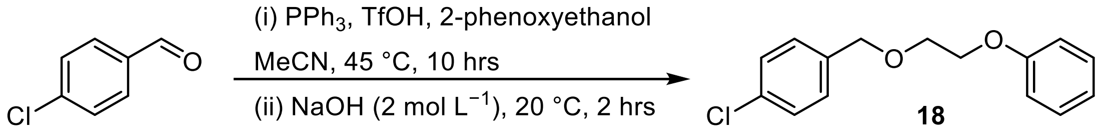

According to General Procedure C, using 4-chlorobenzaldehyde (142 mg, 1.01 mmol, 1.01 equivalent), triphenylphosphine (293, 1.1 mmol, 1.1 equivalents) and trifluoromethanesulfonic acid (205 mg, 1.36 mmol, 1.36 equivalents) in acetonitrile (2.0 mL), followed by addition of 2-phenoxy-1-ethanol (280 mg, 2.05 mmol, 2.05 equivalents). The reaction mixture was stirred at 45 °C for 10 hrs. After the flask was cooled to room temperature, a 2 mol L^{-1} NaOH solution (1.4 mL, 2.8 mmol, 2.8 equivalents) was added and the reaction mixture was stirred for 2 hrs. After work-up, the compound was purified by flash column chromatography (95:5 cyclohexane/EtOAc) to give the product as a colourless oil (179 mg, 0.683 mmol, 68%).

R$_f$ = 0.25

IR $\tilde{\nu}_{max}$ (ATR): 2925 (C—H stretch), 1491 (C—H stretch), 1243 (C—O stretch), 1085 (C—O stretch) cm^{-1}.

HRMS (ESI+): Calculated for $[M + H]^+$ = $[C_{15}H_{15}ClO_2H]^+$ *m/z* = 263.0833; found *m/z* = 263.0827.

^{1}H NMR (300 MHz, $CDCl_3$) δ 7.38 – 7.20 (m, 6H), 7.02 – 6.88 (m, 3H), 4.59 (s, 2H), 4.19 – 4.10 (m, 2H), 3.88 – 3.77 (m, 2H).

^{13}C NMR (126 MHz, $CDCl_3$) δ 158.7, 136.6, 133.4, 129.4, 129.0, 128.5, 120.9, 114.6, 72.5, 68.7, 67.3.

(xxiv) Synthesis of compound 19

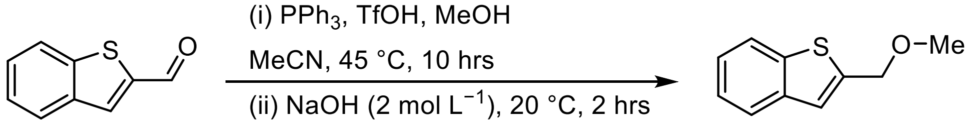

19

According to General Procedure C, using benzo[b]thiophene-2-carbaldehyde (164 mg, 1.01 mmol, 1.01 equivalent), triphenylphosphine (291, 1.1 mmol, 1.1 equivalent) and trifluoromethanesulfonic acid (198 mg, 1.32 mmol, 1.32 equivalent) in acetonitrile (2.0 mL), followed by addition of methanol (75 mg, 2.34 mmol, 2.34 equivalents). The reaction mixture was stirred at 45 °C for 10 hrs. After the flask was cooled to room temperature, a 2 mol L^{-1} NaOH solution (1.4 mL, 2.8 mmol, 2.8 equivalents) was added

and the reaction mixture was stirred for 2 hrs. After work-up, the compound was purified by flash column chromatography (97:3 cyclohexane/EtOAc) to give the product as a colourless oil (158 mg, 0.887 mmol, 88%).

R$_f$ = 0.22

^{1}H NMR (400 MHz, $CDCl_3$) δ 7.87 – 7.80 (m, 1H), 7.79 – 7.71 (m, 1H), 7.41 – 7.28 (m, 2H), 7.22 (s, 1H), 4.72 (s, 2H), 3.44 (s, 3H).[25]

(xxv) Synthesis of compound 20

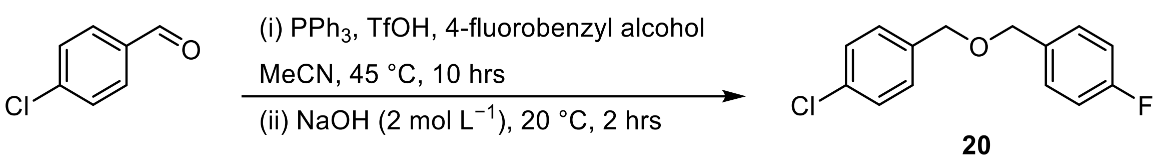

20

According to General Procedure C, using 4-chlorobenzaldehyde (145 mg, 1.03 mmol, 1.03 equivalent) was added to MeCN (2.0 mL), triphenylphosphine (292, 1.11 mmol, 1.11 equivalents) and trifluoromethanesulfonic acid (193 mg, 1.28 mmol, 1.28 equivalents) in acetonitrile (2.0 ml), followed by addition of 4-fluorobenzyl alcohol (253 mg, 2.00 mmol, 2.00 equivalents). The reaction mixture was stirred at 45 °C for 10 hrs. After the flask was cooled to room temperature, a 2 mol L^{-1} NaOH solution (1.4 mL, 2.8 mmol, 2.8 equivalents) was added and the reaction mixture was stirred for 2 hrs. After work-up, the compound was purified by flash column chromatography (95:5 cyclohexane/EtOAc) to give the product as a colourless oil (193 mg, 0.772 mmol, 77%).

R$_f$ = 0.4

IR $\tilde{\nu}_{max}$ (ATR): 2857 (C—H stretch), 1491 (C—H stretch), 1155 (C—O stretch), 1084 (C—F stretch), 820 (C—Cl) cm^{-1}.

GC-HRMS (Electron Ionisation): Calculated for $[M]^+ = [C_{14}H_{12}OClF]^+$ *m/z* = 250.0560; found *m/z* = 250.0561.

^{1}H NMR (400 MHz, $CDCl_3$) δ 7.40 – 7.22 (m, 6H), 7.04 (t, *J* = 8.7 Hz, 2H), 4.51 (s, 4H).

^{13}C NMR (101 MHz, $CDCl_3$) δ 162.5 (d, ^1J = 245.8 Hz), 136.7, 133.8 (d, ^4J = 3.2 Hz), 133.5, 129.6 (d, ^3J = 8.1 Hz), 129.1, 128.7, 115.4 (d, ^2J = 21.4 Hz), 71.6 (d, ^5J = 0.7 Hz), 71.4.

(xxvi) Synthesis of compound 21

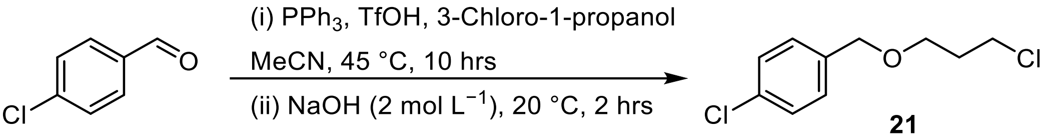

21

According to General Procedure C, using 4-chlorobenzaldehyde (142 mg, 1.01 mmol, 1.01 equivalent), triphenylphosphine (293, 1.11 mmol, 1.1 equivalents) and trifluoromethanesulfonic acid (189 mg, 1.26 mmol, 1.26 equivalents) in acetonitrile (2.0 mL), followed by addition of 3-chloro-1-propanol (180 mg, 1.90 mmol, 1.90 equivalents) was then added and the reaction mixture was stirred at 45 °C for 10 hrs. After the flask was cooled to room temperature, a 2 mol L^{-1} NaOH solution (1.0 mL, 2.0 mmol, 2.0 equivalents) was added and the reaction mixture was stirred for 2 hrs. After work-up, the compound was purified by flash column chromatography (96:4 cyclohexane/EtOAc) to give the product as a colourless oil (189 mg, 0.863 mmol, 86%).

$\mathbf{R}_f$ = 0.38

IR $\tilde{\nu}_{max}$ (ATR): 2863 (C—H stretch), 1490 (C—H stretch), 1085 (C—O stretch), 805 (C—Cl) cm^{-1}.

GC-HRMS (Electron Ionisation): Calculated for $[M]^+ = [C_{10}H_{12}Cl_2O]^+$ *m/z* = 218.0265; found *m/z* = 218.0075.

^{1}H NMR (400 MHz, $CDCl_3$) δ 7.43 – 7.14 (m, 4H), 4.47 (s, 2H), 3.66 (t, *J* = 6.2 Hz, 2H), 3.60 (t, *J* = 5.9 Hz, 2H), 2.04 (quint, *J* = 6.2 Hz, 2H).

^{13}C NMR (101 MHz, $CDCl_3$) δ 136.8, 133.4, 128.9, 128.6, 72.4, 66.8, 41.9, 32.8.

(xxvii) Synthesis of compound 22

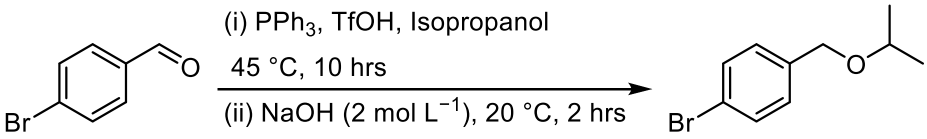

22

According to General Procedure C, using 4-bromobenzaldehyde (191 mg, 1.03 mmol, 1.03 equivalent), triphenylphosphine (292 mg, 1.1 mmol, 1.1 equivalents) and trifluoromethanesulfonic acid (196 mg, 1.30 mmol, 1.30 equivalents) in isopropanol (2.0 mL). The reaction mixture was stirred at 45 °C for 10 hrs. After the flask was cooled to room temperature, a 2 mol L^{-1} NaOH solution (1.4 mL, 2.8 mmol, 2.8 equivalents) was added and the reaction mixture was stirred for 2 hrs. The compound was purified by flash column chromatography (96:4 cyclohexane/EtOAc) to give the product as a colourless oil (200 mg, 0.877 mmol, 87%).

R*f* = 0.48

^{1}H NMR (400 MHz, $CDCl_3$) δ 7.48 – 7.40 (m, 2H), 7.24 – 7.16 (m, 2H), 4.44 (s, 2H), 3.66 (sept, *J* = 6.1 Hz, 1H), 1.20 (d, *J* = 6.1 Hz, 6H).[p, 26]

(xxviii) Synthesis of compound 23

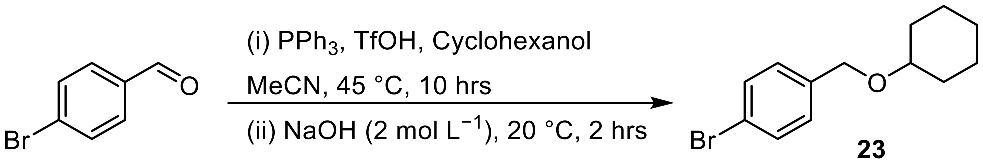

According to General Procedure C, using 4-bromobenzaldehyde (189 mg, 1.02 mmol, 1.02 equivalent), triphenylphosphine (291, 1.1 mmol, 1.1 equivalents) and trifluoromethanesulfonic acid (186 mg, 1.24 mmol, 1.24 equivalents) in acetonitrile (2.0 mL), followed by addition of cyclohexanol (192 mg, 1.92 mmol, 1.92 equivalents). The reaction mixture was stirred at 45 °C for 10 hrs. After the flask was cooled to room temperature, a 2 mol L^{-1} NaOH solution (1.4 mL, 2.8 mmol, 2.8 equivalents) was added and the reaction mixture was stirred for 2 hrs. After work-up, the compound was purified by flash column chromatography (97:3 cyclohexane/EtOAc) to give the product as a yellow oil (210 mg, 0.781 mmol, 78%).

R*f* = 0.42

^{1}H NMR (300 MHz, $CDCl_3$) δ 7.50 – 7.41 (m, 2H), 7.25 – 7.18 (m, 2H), 4.49 (s, 2H), 3.33 (tt, *J* = 9.1, 3.7 Hz, 1H), 2.02 – 1.87 (m, 2H), 1.83 – 1.65 (m, 2H), 1.63 – 1.47 (m, 1H), 1.46 – 1.10 (m, 5H).[q, 27]

(xxix) Synthesis of compound 24

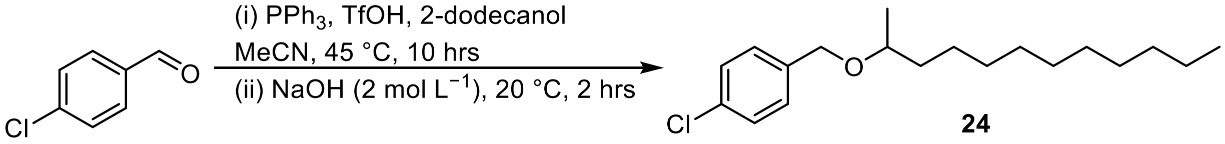

According to General Procedure C, using 4-chlorobenzaldehyde (141 mg, 1.00 mmol, 1.00 equivalent), triphenylphosphine (291 mg, 1.1 mmol, 1.1 equivalents) and trifluoromethanesulfonic acid (189 mg,

---

p The signals at δ 7.48 – 7.40 ppm (m, 2H) and δ 7.24 – 7.16 ppm (m, 2H) are reported as δ 7.25 ppm (d, *J* = 8.4 Hz, 1H) and δ 7.02 ppm (d, *J* = 8.4 Hz, 1H) in reference 24. However, in the spectrum we obtained for this compound, finer splitting is observed. The signal at δ 3.66 ppm (sept, J = 6.1 Hz, 1H) is reported as δ 3.46 ppm (m, 1H) in reference 22. However, in the spectrum provided below, more defined splitting is observed.

q The signals at δ 7.50 – 7.41 ppm (m, 2H) and δ 7.25 – 7.18 ppm (m, 2H) are reported as δ 7.45 ppm (d, J = 8.0 Hz, 1H) and δ 7.22 ppm (d, *J* = 8.0 Hz, 1H) in reference 25. However, in the spectrum we obtained for this compound, finer splitting is observed.

1.26 mmol, 1.26 equivalents) in acetonitrile (2.0 mL), followed by addition of 2-dodecanol (381 mg, 2.08 mmol, 2.08 equivalents). The reaction mixture was stirred at 45 °C for 10 hrs. After the flask was cooled to room temperature, a 2 mol L^{-1} NaOH solution (1.4 mL, 2.8 mmol, 2.8 equivalents) was added and the reaction mixture was stirred for 2 hrs. After work-up, the compound was purified by flash column chromatography (100% cyclohexane) to give the product as a colourless oil (242 mg, 0.780 mmol, 78%).

R_f = 0.36

IR $\tilde{\nu}_{max}$ (ATR): 2922 (C—H stretch), 1464 (C—H stretch), 1087 (C—O stretch), 806 (C—Cl) cm^{-1}.

HRMS (ESI+): Calculated for $[M + Na]^+ = [C_{19}H_{31}ClONa]^+$ *m/z* = 333.1955; found *m/z* = 333.1960

^{1}H NMR (300 MHz, $CDCl_3$) δ 7.32 – 7.22 (m, 4H), 4.51 (d, *J* = 12.0 Hz, 1H), 4.40 (d, *J* = 12.0 Hz, 1H), 3.47 (dt, *J* = 7.6, 3.9 Hz, 1H), 1.71 – 1.20 (m, 18H), 1.17 (d, *J* = 6.1 Hz, 3H), 0.88 (t, *J* = 6.7 Hz, 3H).

^{13}C NMR (126 MHz, $CDCl_3$) δ 137.8, 133.1, 128.9, 128.4, 75.2, 69.8, 69.5, 36.7, 31.9, 29.8, 29.7, 29.6, 29.3, 25.6, 22.7, 19.6, 14.1.

(xxx) Synthesis of compound 25

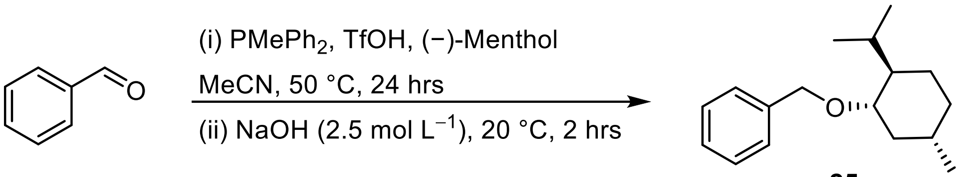

According to General Procedure C, using benzaldehyde (107 mg, 1.00 mmol, 1.00 equivalent), MeCN (0.5 mL), methyldiphenylphosphine (186 mg, 0.930 mmol, 0.930 equivalent) and (−)-menthol (330 mg, 2.11 mmol, 2.11 equivalent) were treated with trifluoromethanesulfonic acid (195 mg, 1.31 mmol, 1.31 equivalents) and the reaction mixture was stirred at 50 °C for 24 hrs after which time the solvent was removed *in vacuo* at 50 °C. NMR spectral analysis of the reaction mixture confirmed the formation of phosphonium salt **10** in 91% NMR spectral yield and the presence of unreacted methyldiphenylphosphine in 9% NMR spectral yield After the flask was cooled to room temperature, MeCN (5 mL) and a 2.5 mol L^{-1} NaOH solution (1.5 mL, 5.0 mmol, 5.0 equivalents) was added and the reaction mixture was stirred for 24 hrs. After work-up, the compound was purified by flash column chromatography (100% cyclohexane) (98:2 cyclohexane/EtOAc) to give the product as a colourless oil (220 mg, 0.894 mmol, 89 %).

HRMS (ESI+): calculated for $[M + H]^+ = [C_{17}H_{27}O]^+$ *m/z* = 247.2056; found *m/z* = 247.2055.

^{1}H NMR (400 MHz, $CDCl_3$) δ 7.35 (m, 4H), 7.33 (d, *J* = 8.4 Hz, 1H), 7.27 (dd, *J* = 8.4, 2.8 Hz, 1H), 4.68 (d, *J* = 11.4 Hz, 1H), 4.42 (d, *J* = 11.4 Hz, 1H), 3.19 (td, *J* = 10.6, 4.1 Hz, 1H), 2.33 (hd, *J* = 7, 3 Hz, 1H), 2.21 (m, 1H), 1.67 (m, 1H), 1.64 (m, 1H), 1.36 (m, 1H), 1.31 (m, 1H), 0.99 (m, 1H), 0.96 (d, *J* = 6.5 Hz, 3H), 0.92 (d, *J* = 7 Hz, 3H), 0.88 (m, 1H) (s, 3H), 0.73 (d, *J* = 7 Hz, 3H).

^{13}C NMR (101 MHz, $CDCl_3$) δ 139.1, 128.3, 127.8, 127.4, 78.8, 70.4, 48.4, 40.3, 34.5, 31.6, 25.5, 23.2, 22.5, 21.0, 16.1.

(xxxi) Synthesis of compound 26

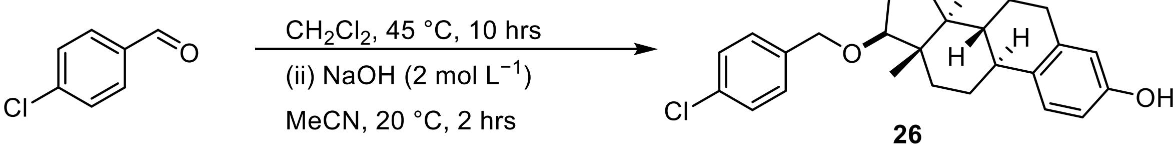

According to General Procedure C, using 4-chlorobenzaldehyde (141 mg, 1.00 mmol, 1.00 equivalent), triphenylphosphine (291, 1.11 mmol, 1.11 equivalents) and trifluoromethanesulfonic acid (198 mg, 1.32 mmol, 1.32 equivalents) in CH_2Cl_2 (2.0 mL), followed by addition of estradiol (558 mg, 2.05 mmol,

2.05 equivalent). The reaction mixture was stirred at 45 °C for 10 hrs. After the flask was cooled to room temperature, the CH_2Cl_2 solvent was removed *in vacuo* to give a viscous colourless oil. MeCN (5.0 mL) and 2 mol L^{-1} NaOH solution (1.4 mL, 2.8 mmol, 2.8 equivalents) were added to the flask and the contents were stirred for 2 hrs. After work-up, the compound was purified by flash column chromatography (85:15 cyclohexane/EtOAc) to give the product as a colourless oil (266 mg, 0.672 mmol, 67%).

NOTE: CH_2Cl_2 used instead of MeCN for formation of (α-alkoxyalkyl)phosphonium triflate due to insolubility of estradiol in MeCN.

$\mathbf{R}_f$ = 0.2

IR $\tilde{\nu}_{max}$ (ATR): 3355 (O—H stretch), 2851 (C—H stretch), 1491 (C—H stretch), 1220 (C—O stretch) cm^{-1}.

HRMS [ESI] was calculated for $[C_{25}H_{29}O_2Cl]^-$ $[M - H]^-$: 396.1856 *m/z*, found = 395.1779 *m/z*.

^{1}H NMR (400 MHz, $CDCl_3$) δ 7.31 (m, 4H), 7.15 (d, *J* = 8.4 Hz, 1H), 6.63 (dd, *J* = 8.4, 2.8 Hz, 1H), 6.55 (d, *J* = 2.8 Hz, 1H), 5.15 (s, 1H), 4.55 (s, 2H), 3.50 (t, *J* = 8.3 Hz, 1H), 2.95 – 2.70 (m, 2H), 2.37 – 2.22 (m, 1H), 2.23 – 2.12 (m, 1H), 2.12 – 1.96 (m, 1H), 1.92 – 1.79 (m, 1H), 1.77 – 1.10 (m, 8H), 0.88 (s, 3H).

^{13}C NMR (101 MHz, $CDCl_3$) δ 153.4, 138.3, 137.8, 133.1, 132.7, 128.8, 128.5, 126.6, 115.4, 112.8, 88.6, 71.0, 50.3, 44.0, 43.5, 38.7, 38.0, 29.7, 28.1, 27.3, 26.5, 23.2, 11.9.

(xxxii) Synthesis of compound 27

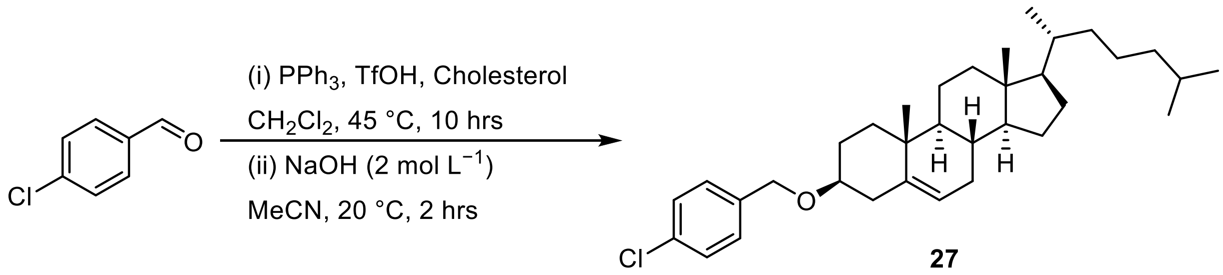

According to General Procedure C, using 4-chlorobenzaldehyde (141 mg, 1.00 mmol, 1.00 equivalent), triphenylphosphine (291, 1.11 mmol, 1.11 equivalents) and trifluoromethanesulfonic acid (197 mg, 1.31 mmol, 1.31 equivalents) in CH_2Cl_2 (2.0 mL), followed by addition of cholesterol (759 mg, 1.96 mmol, 1.96 equivalent). The reaction mixture was stirred at 45 °C for 10 hrs. After the flask was cooled to room temperature, the CH_2Cl_2 solvent was removed *in vacuo* to give a viscous colourless oil. MeCN (5.0 mL) and 2 mol L^{-1} NaOH solution (1.4 mL, 2.8 mmol, 2.8 equivalents) were added to the flask and the contents were stirred for 2 hrs. After work-up, the compound was purified by flash column

chromatography (98:2 cyclohexane/EtOAc) to give the product as a colourless oil (425 mg, 0.831 mmol, 83%).

NOTE: CH_2Cl_2 used instead of MeCN for formation of (α-alkoxyalkyl)phosphonium triflate due to insolubility of cholesterol in MeCN.

R*f* = 0.38

HRMS (ESI+): Calculated for $[M + H]^+ = [C_{34}H_{51}ClOH]^+$ *m/z* = 511.3701; found *m/z* = 511.3679

^{1}H NMR (400 MHz, $CDCl_3$) δ 7.33 – 7.21 (m, 4H), 5.33 (d, *J* = 5.4 Hz, 1H), 4.51 (s, 2H), 3.24 (tt, *J* = 11.2, 4.6 Hz, 1H), 2.39 (ddd, *J* = 13.2, 4.8, 2.3 Hz, 1H), 2.31 – 2.25 (m, 1H), 2.08 – 1.73 (m, 6H), 1.68 – 0.94 (m, 24H), 0.90 (d, J = 6.5 Hz, 3H), 0.85 (dd, *J* = 6.6, 1.8 Hz, 6H), 0.67 (s, 3H).

^{13}C NMR (101 MHz, $CDCl_3$) δ 140.9, 137.7, 133.2, 128.9, 128.5, 121.8, 78.8, 69.2, 56.8, 56.5, 50.8, 42.1, 39.7, 39.2, 39.1, 37.0, 36.8, 36.3, 35.8, 32.0, 31.9, 28.5, 28.3, 28.1, 24.3, 23.9, 22.9, 22.6, 21.2, 19.4, 18.8, 11.9.

6. Synthesis of Deuterated Ethers Using NaOD in the Hydrolytic Etherification Protocol

General Procedure D

Acetonitrile (2.0 mL) was added to a round bottom flask that was equipped with a stirring bar. To this, the following were added sequentially: triphenylphosphine (288 mg, 1.10 mmol, 1.10 equivalents), trifluoromethanesulfonic acid (180 mg, 1.20 mmol, 1.20 equivalents) and aldehyde substrate (1.0 mmol, 1.0 equivalent) and the contents of the flask were stirred at room temperature. The alcohol substrate (1.9 mmol, 1.9 equivalents) was then added and the flask was sealed with a plastic lid. The flask was placed in an oil bath set at 45 °C for 10 hours. After this time, the flask was removed from the oil bath, and the reaction mixture was allowed to cool to room temperature. A 30 – 40 wt% solution of NaOD in D_2O (2.8 mmol, 2.8 equivalents) was added to the flask and it was stirred at room temperature for 2 hours. Upon completion of the reaction, the mixture was diluted in ethyl acetate (10 mL) and extracted with a saturated brine solution (10 mL). The aqueous layer was extracted twice more with 10 mL portions of ethyl acetate (2 × 10 mL). The organic phases were combined and dried over anhydrous magnesium sulfate, filtered and concentrated *in vacuo*.

The solution was worked up as per the process outlined in *Org. Process Res. Dev*. **2017**, *21*, 1394−1403.[8] The residue was taken up in methanol (5 mL) and added to a separatory funnel along with a saturated sodium bisulfite solution (3 mL). The separatory funnel was vigorously shaken for approximately 30 s and H_2O (25 mL) was added. The mixture was extracted with 90:10 cyclohexane/ethyl acetate (25 mL). The aqueous layer was extracted twice more with 90:10 cyclohexane/ethyl acetate (2 × 25 mL). The combined organic layers were dried over anhydrous magnesium sulfate, filtered and concentrated *in vacuo*. Unless otherwise stated, the residual material remaining after solvent removal was purified by flash column chromatography on silica (SiO_2) to give the deuterated ether product.

The isolated products were analysed using ^{1}H and ^{13}C NMR spectroscopy in $CDCl_3$. For ^{2}H NMR spectroscopy, the probe was first locked and shimmed manually on a blank sample of C*D*Cl_3. Subsequently a second sample containing the product dissolved in C*H*Cl_3 was input into the spectrometer and analysed at 77 MHz to obtain a ^{2}H NMR spectrum of the deuterated ether.

(i) Synthesis of compound 13g

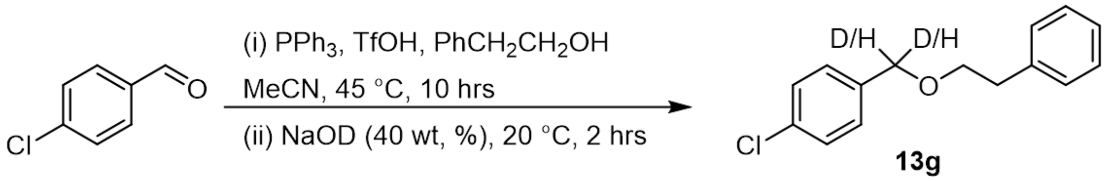

13g

74% dideuterated
23% monodeuterated
3% non-deuterated

According to General Procedure C, using 4-chlorobenzaldehyde (141 mg, 1.00 mmol, 1.00 equivalent), triphenylphosphine (291 mg, 1.11 mmol, 1.11 equivalents) and trifluoromethanesulfonic acid (187 mg, 1.24 mmol, 1.24 equivalents) in acetonitrile (2.0 mL), followed by addition of 2-phenyl-1-ethanol (244 mg, 2.00 mmol, 2.00 equivalents). The reaction mixture was stirred at 45 °C for 10 hrs. After the flask was cooled to room temperature, a 14.3 mol L^{-1} NaOD (40 wt%) solution in D_2O (0.20 mL, 2.86 mmol, 2.86 equivalents) was added and the reaction mixture was stirred for 2 hrs. After work-up, the compound was purified by flash column chromatography (95:5 cyclohexane/EtOAc) to give the product as a colourless oil (226 mg, 0.913 mmol, 91%).

R*f* = 0.40

GC-HRMS (Electron Ionisation): Calculated for $[M]^+ = [C_{15}H_{13}D_2ClO]^+$ *m/z* = 248.0937; found *m/z* = 248.0926. Calculated for $[M]^+ = [C_{15}H_{14}DClO]^+$ *m/z* = 247.0874; found *m/z* = 247.0843.

^{1}H NMR (400 MHz, $CDCl_3$) δ 7.35 – 7.25 (m, 4H), 7.25 – 7.15 (m, 5H), 4.49 (s, approximate integration = 0.05H (2H of non-deuterated product)), δ 4.47 (t, *J* = 1.7 Hz, approximate integration = 0.23H (1H of monodeuterated product)), 3.73 – 3.66 (contains 2 × t (overlapping) each with *J* = 7.1 Hz, 2H, overlapping signals of dideuterated, monodeuterated and non-deuterated species), 2.92 (t, *J* = 7.1 Hz, 2H).

^{2}H NMR (77 MHz, $CHCl_3$) δ 4.44.

^{13}C NMR (101 MHz, $CDCl_3$) Overlapping signals of the di-, mono- and non-deuterated product:
δ_C 138.9, 129.03, 128.6, 128.4, 126.3, 72.3 – 71.1 (m),[28,r] 36.5.[s]

Signals assigned to the dideuterated product: δ_C 136.9, 133.37, 129.01.[s]
Signals assigned to the monodeuterated product: δ_C 137.0, 133.35, 128.98.[s]
Signals assigned to the non-deuterated product: δ_C 137.1, 133.3, 128.95.[s]

---

[r] Multiplet appears to be composed of 72.2 (s, C-*C*H_2-O-), 71.9 (t, *J* = 22.5 Hz, C-*C*HD-O-), 71.5 (quin, *J* = 22.5 Hz, C-*C*D_2-O-, weak signal due to deuterium isotope effect on ^{13}C relaxation delay time, see reference 26 for more detail), 71.44 (s, -CH_2-O-*C*H_2-CH_2-), 71.39 (-CDH-O-*C*H_2-), 71.3 (s, -CD_2-O-*C*H_2-).

[s] Note: peaks in this spectrum with very similar chemical shifts are listed with two decimal places indicated.

(ii) Synthesis of compound 13h

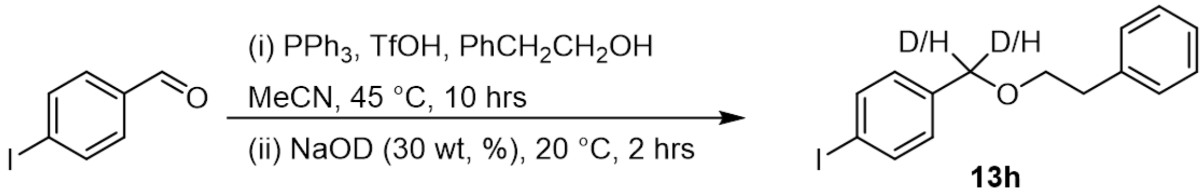

13h

64% dideuterated
32% monodeuterated
4% non-deuterated

According to General Procedure D, using 4-iodobenzaldehyde (236 mg, 1.02 mmol, 1.00 equivalent), triphenylphosphine (301 mg, 1.15 mmol, 1.13 equivalents) and trifluoromethanesulfonic acid (232 mg, 1.55 mmol, 1.52 equivalents) in acetonitrile (2.0 mL), followed by addition of 2-phenyleth-1-anol (236 mg, 1.93 mmol, 1.89 equivalents). The reaction mixture was stirred at 45 °C for 10 hrs. After the flask was cooled to room temperature, a 30 wt% solution of NaOD in D_2O (421 mg, 3.08 mmol, 3.02 equivalents) was added and the reaction mixture was stirred for 2 hrs. After work-up, the compound was purified by flash column chromatography (95:5 cyclohexane/EtOAc) to give the title compound as a colourless oil (300 mg, 0.883 mmol, 87%).

R$_f$ = 0.44

IR $\tilde{\nu}_{max}$ (ATR): 3026 (C—H stretch), 2854 (C—H stretch), 2091 (C—D stretch), 1590 (C=C stretch), 1483 (C—H stretch), 1435 (C=C stretch), 1100 (C—O stretch) cm^{-1}.

GC-HRMS (Electron Ionisation): Calculated for $[M + Na]^+ = [C_{15}H_{13}D_2IONa]^+$ *m/z* = 363.0191; found *m/z* = 363.0182; calculated for $[M + Na]^+ = [C_{15}H_{14}DIONa]^+$ *m/z* = 363.0190; found *m/z* = 363.0182.

^{1}H NMR (400 MHz, $CDCl_3$) – Integrations are set such that 1H = the combined integration of 1H each of the di-, mono- and non-deuterated product.

δ 7.65 – 7.61 (m, 2H), 7.34 – 7.17 (contains C*H*Cl_3 signal & 2H of product), 7.23 – 7.18 (m, 3H), 7.03 – 6.97 (m, 2H), 4.44 (approximate integration = 0.05H (2H of non-deuterated product)), 4.42 (t, *J* = 1.8 Hz, approximate integration = 0.35H (1H of monodeuterated product)), 3.70 – 3.61 (contains 2 × t (overlapping) each with *J* = 7.1 Hz, 2H, overlapping signals of dideuterated, monodeuterated and non-deuterated species), 2.91 (t, *J* = 7.1 Hz, 2H).

^{2}H NMR (77 MHz, $CHCl_3$) δ 4.45.

^{13}C NMR (101 MHz, $CDCl_3$) Overlapping signals of the di-, mono- and non-deuterated product: δ_C 139.0, 137.5, 129.0, 128.5, 126.4, 72.4 – 71.5 (m),[26,t] 36.5.[u]

Signals assigned to the dideuterated product: δ_C 138.1, 129.6, 93.07.[u]

Signals assigned to the monodeuterated product: δ_C 138.2, 129.54, 93.05.[u]

Signals assigned to the non-deuterated product: δ_C 138.3, 129.51, 93.0.[u]

(iii) Synthesis of compound 14n

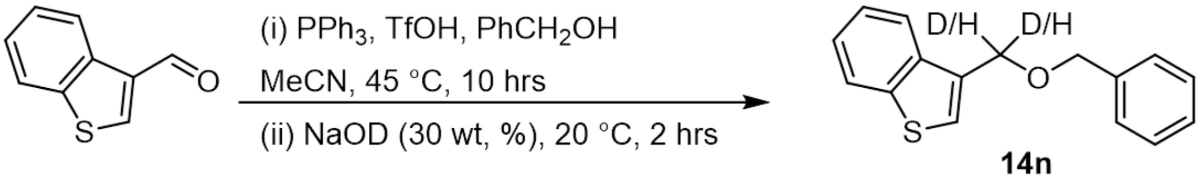

14n

67% dideuterated
30% monodeuterated
3% non-deuterated

According to General Procedure D, using 1-benzothiophene-3-carbaldehyde (166 mg, 1.02 mmol, 1.00 equivalent), triphenylphosphine (320 mg, 1.22 mmol, 1.20 equivalents) and trifluoromethanesulfonic acid (313 mg, 2.09 mmol, 2.05 equivalents) in acetonitrile (2.0 mL), followed by addition of benzyl alcohol (278 mg, 2.55 mmol, 2.50 equivalents). The reaction mixture was stirred at 45 °C for 10 hrs. After the flask was cooled to room temperature, a 30 wt. % solution of NaOD in D_2O (415 mg, 3.04 mmol, 2.99 equivalents) was added and the reaction mixture was stirred for 2 hrs. After work-up, the compound was purified by flash column chromatography (95:5 cyclohexane/EtOAc) to give the title compound as a pale-yellow solid (209 mg, 0.817 mmol, 80%).

R$_f$ = 0.32

IR $\tilde{\nu}_{max}$ (ATR): 3090 (C—H stretch), 2866 (C—H stretch), 2175 (C—D stretch), 1537 (C=C stretch), 1497 (C—H stretch), 1393 (C=C stretch), 1047 (C—O stretch).

GC-HRMS (Electron Ionisation): Calculated for $[M + Na]^+ = [C_{16}H_{12}D_2OSNa]^+$ *m/z* = 279.0789; found *m/z* = 279.0781; calculated for $[M + Na]^+ = [C_{16}H_{13}DOSNa]^+$ *m/z* = 278.0726; found *m/z* = 278.0720; calculated for $[M + Na]^+ = [C_{16}H_{14}OSNa]^+$ *m/z* = 277.0663; found *m/z* = 277.0657.

Melting Point: 52 – 54 °C.

---

[t] Multiplet appears to be composed of δ 72.3 (s, C-CH_2-O), 72.0 (t, *J* = 21.7 Hz, C-*C*HD-O), 71.6 (quin, *J* = 21.42 Hz, C-*C*D_2-O, very weak signal due to deuterium isotope effect on ^{13}C relaxation delay time, see reference 26 for more details), 71.5 (s, CH_2-O-*C*H_2-CH_2), 71.41 (s, CDH-O-*C*H_2), 71.35 (s, CD_2-O-*C*H_2).

[u] Note: peaks in this spectrum with very similar chemical shifts are listed with two decimal places indicated.

^{1}H NMR (400 MHz, $CDCl_3$) (400 MHz, $CDCl_3$) – Integrations are set such that 1H is equal to the combined integration of 1H each of the di-, mono- and non-deuterated product.

δ 7.86 – 7.82 (m, 1H), 7.77 – 7.74 (m, 1H), 7.41 – 7.29 (m, 7H), 7.24 (s, contains C*H*Cl_3 signal & 1H of product), 4.83 (d, J = 1.0 Hz, approximate integration 0.12H (2H of non-deuterated product)), 4.81 (s, approximate integration 0.25H (1H of monodeuterated product)), 4.62 – 4.59 (contains 3 × s (overlapping), 2H, overlapping signals of dideuterated, monodeuterated and non-deuterated species).

^{2}H NMR (77 MHz, $CHCl_3$) δ 4.80

^{13}C NMR (101 MHz, $CDCl_3$) Overlapping signals of the di-, mono- and non-deuterated product: δ_C 140.4, 139.6, 137.93, 128.6, 128.0, 124.39, 124.36, 123.6, 67.4 – 66.1 (m),[26,v] 122.6.[w]

Signals assigned to the dideuterated product: δ_C 142.2, 127.93, 122.80, 71.9.[w]

Signals assigned to the monodeuterated product: δ_C 142.3, 127.91, 122.77, 72.0.[w]

Signals assigned to the non-deuterated product: δ_C 142.4, 138.4, 128.5, 127.8, 122.7, 72.2.[w]

v Multiplet appears to be composed of 67.3 (s, C-*C*H_2-O-), 67.0 (t, J = 22.2 Hz, C-*C*HD-O-), 66.9 (quin, J = 21.9 Hz, C-*C*D_2-O-, weak signal due to deuterium isotope effect on ^{13}C relaxation delay time, see reference 26 for more details).

w Note: peaks in this spectrum with very similar chemical shifts are listed with two decimal places indicated.

7. Hydrolytic Etherification of Aldehydes using Silyl Ethers

General Procedure E: Acetonitrile (2.0 mL) was added to a round bottom flask that was equipped with a stirring bar. To this, the following were added sequentially: triphenylphosphine (288 mg, 1.11 mmol, 1.11 equivalents), trifluoromethanesulfonic acid (180 mg, 1.20 mmol, 1.20 equivalents) and aldehyde substrate (1.0 mmol, 1.0 equivalent) and the contents of the flask were stirred at room temperature. The trimethylsilyl or triethylsilyl ether substrate (1.9 mmol, 1.9 equivalents) was then added and the flask was sealed with a plastic lid. The flask was placed in an oil bath set at 45 °C for 10 hours. After this time, the flask was removed from the oil bath, and the reaction mixture was allowed to cool to room temperature. A 2 mol L^{-1} solution of NaOH (1.4 mL, 2.8 mmol, 2.8 equivalents) was added to the flask and it was stirred at room temperature for 2 hours. Upon completion of the reaction, the mixture was diluted in ethyl acetate (10 mL) and extracted with a saturated brine solution (10 mL). The aqueous layer was extracted twice more with 10 mL portions of ethyl acetate (2 × 10 mL). The organic phases were combined and dried over anhydrous magnesium sulfate, filtered and concentrated *in vacuo*. The solution was worked up as per the process outlined in *Org. Process Res. Dev*. **2017**, *21*, 1394−1403.[8] The residue was taken up in methanol (5 mL) and added to a separatory funnel along with a saturated sodium bisulfite solution (3 mL). The separatory funnel was vigorously shaken for approximately 30 s and H_2O (25 mL) was added. The mixture was extracted with 90:10 cyclohexane/ethyl acetate (25 mL). The aqueous layer was extracted twice more with 90:10 cyclohexane/ethyl acetate (2 × 25 mL). The combined organic layers were dried over anhydrous magnesium sulfate, filtered and concentrated *in vacuo*. Unless otherwise stated, the residual material remaining after solvent removal was purified by flash column chromatography on silica (SiO_2) to give the ether product.

(i) Synthesis of compound 13i

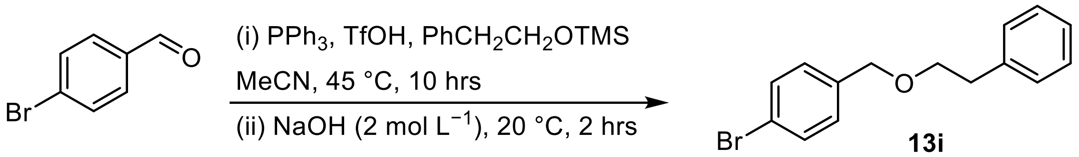

According to General Procedure E, using 4-bromobenzaldehyde (187 mg, 1.01 mmol, 1.01 equivalent), triphenylphosphine (289, 1.10 mmol, 1.10 equivalents) and trifluoromethanesulfonic acid (202 mg, 1.34 mmol, 1.34 equivalents) in acetonitrile (2.0 mL), followed by addition of 2-phenyleth-1-yl(trimethylsilyl) ether (365 mg, 1.88 mmol, 1.88 equivalents). The reaction mixture was stirred at 45 °C for 10 hrs. After the flask was cooled to room temperature, a 2 mol L^{-1} NaOH solution (1.4 mL, 2.8 mmol, 2.8 equivalent) was added and the reaction mixture was stirred for 2 hrs. After work-up, the compound was purified by flash column chromatography (96:4 cyclohexane/EtOAc) to give the product as a colourless oil (260 mg, 0.893 mmol, 89%).

R_f = 0.25

^{1}H NMR (300 MHz, $CDCl_3$) δ 7.48 – 7.39 (m, 2H), 7.33 – 7.17 (m, 5H), 7.16 – 7.10 (m, 2H), 4.45 (s, 2H), 3.67 (t, J = 7.1 Hz, 2H), 2.92 (t, J = 7.1 Hz, 2H).[x, 29]

(ii) Synthesis of compound 14o

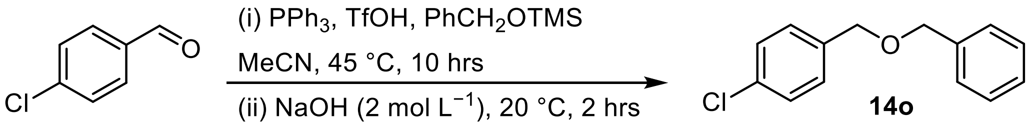

According to General Procedure E, using 4-chlorobenzaldehyde (143 mg, 1.02 mmol, 1.02 equivalent), triphenylphosphine (290 mg, 1.10 mmol, 1.10 equivalents) and trifluoromethanesulfonic acid (193 mg, 1.28 mmol, 1.28 equivalents) in acetonitrile (2.0 mL), followed by benzyloxytrimethylsilane ($PhCH_2OTMS$) (357 mg, 1.98 mmol, 1.98 equivalents) and the flask stirred at 45 °C for 10 hrs. After the flask was cooled to room temperature, a 2 mol L^{-1} NaOH solution (1.4 mL, 2.8 mmol, 2.8 equivalents) was added and the flask stirred for 2 hrs. The compound was purified by flash column chromatography (98:2 cyclohexane/EtOAc) to give the product as a colourless oil (193 mg, 0.831 mmol, 83%).

R_f = 0.30

^{1}H NMR (300 MHz, $CDCl_3$) δ 7.47 – 7.27 (m, 9H), 4.58 (s, 2H), 4.54 (s, 2H).[27]

---

[x] The signals at δ 7.33 – 7.17 ppm (m, 5H) and 7.16 – 7.10 (m, 2H) are reported in reference 27 as 7.28 – 7.13 ppm (m, 7H). However, in our spectrum for this compound, the signals are baseline separated.

(iii) Synthesis of compound 31

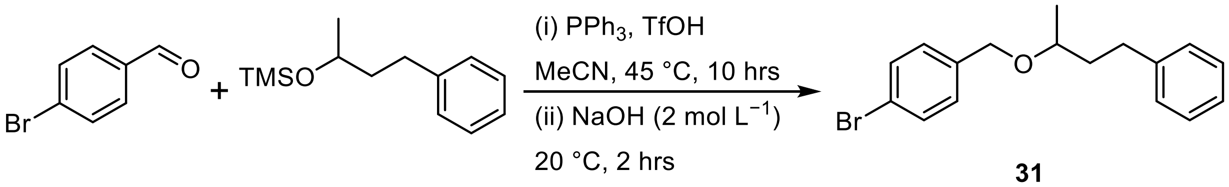

31

According to General Procedure E, using 4-bromobenzaldehyde (189 mg, 1.02 mmol, 1.02 equivalent) was added to MeCN (2.0 mL), triphenylphosphine (289 mg, 1.10 mmol, 1.10 equivalents) and trifluoromethanesulfonic acid (202 mg, 1.34 mmol, 1.34 equivalents) in acetonitrile (2.0 mL), followed by addition of 4-phenylbutan-2-yl (trimethylsilyl) ether (421 mg, 1.91 mmol, 1.91 equivalents). The reaction mixture was stirred at 45 °C for 10 hrs. After the flask was cooled to room temperature, a 2 mol L^{-1} NaOH solution (1.4 mL, 2.8 mmol, 2.8 equivalent) was added and the reaction mixture was stirred for 2 hrs. After work-up, the compound was purified by flash column chromatography (95:5 cyclohexane/EtOAc) to give the product as a colourless oil (282 mg, 0.884 mmol, 88%).

$\mathbf{R}_f$ = 0.43

^{1}H NMR (300 MHz, $CDCl_3$) δ 7.50 – 7.39 (m, 2H), 7.35 – 7.09 (m, 7H), 4.51 (d, J = 12.0 Hz, 1H), 4.37 (d, J = 12.0 Hz, 1H), 3.59 – 3.42 (m, 1H), 2.70 (m, 2H), 1.98 – 1.91 (m, 1H), 1.78 – 1.71 (m, 1H), 1.21 (d, J = 6.1 Hz, 3H).[y, 30]

(iv) Synthesis of compound 13j

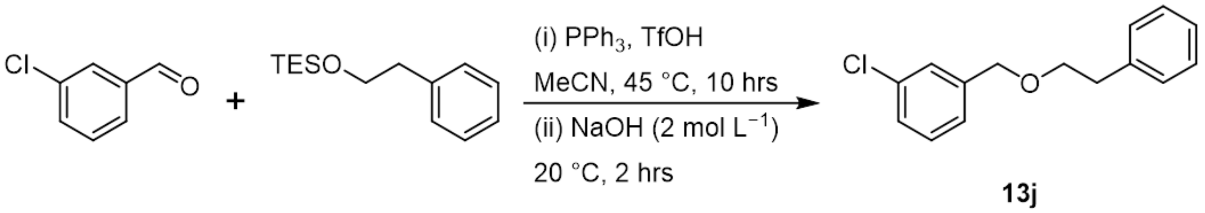

13j

According to General Procedure E, using 3-chlorobenzaldehyde (142 mg, 1.01 mmol, 1.00 equivalent), triphenylphosphine (319, 1.22 mmol, 1.20 equivalents) and trifluoromethanesulfonic acid (0.270 mg, 1.80 mmol, 1.78 equivalents) in acetonitrile (2.0 mL), followed by addition of 2-phenyleth-1-yl(trimethylsilyl) ether (516 mg, 2.18 mmol, 2.16 equivalents). The reaction mixture was stirred at 45 °C for 10 hrs. After the flask was cooled to room temperature, a 2 mol L^{-1} NaOH solution (1.6 mL, 3.2 mmol, 3.2 equivalent) was added and the reaction mixture was stirred for 2 hrs. After work-up, the

[y] The signal at δ 7.50 – 7.39 ppm (m, 2H) is reported as δ 7.46 ppm (d, J = 8.4 Hz, 1H) in reference 28. However, in the spectrum we obtained for this compound, the signals exhibit more complex splitting patterns.

compound was purified by flash column chromatography (96:4 cyclohexane/EtOAc) to give the product as a colourless oil (206 mg, 0.835 mmol, 83%).

R$_f$ = 0.45

IR $\tilde{\nu}_{max}$ (ATR): 3027 (C—H stretch), 2919 (C—H stretch), 2858 (C—H stretch), 1600 (C=C stretch), 1491 (C—H stretch), 1243 (C—O stretch), 1082 (C—O stretch) cm^{-1}

GC-HRMS (Electron Ionisation): Calculated for $[M + Na]^+$ = $[C_{15}H_{15}ClONa]^+$ *m/z* = 269.0709; found *m/z* = 269.0704.

^{1}H NMR (500 MHz, $CDCl_3$) δ 7.33 – 7.18 (m, contains C*H*Cl_3 signal and 8H of product), 7.19 – 7.12 (m, 1H), 4.48 (s, 2H), 3.69 (t, *J* = 7.1 Hz, 2H), 2.94 (t, *J* = 7.1 Hz, 2H).

^{13}C NMR (101 MHz, $CDCl_3$) δ 140.6, 138.8, 134.3, 129.6, 128.9, 128.4, 127.6, 127.5, 126.3, 125.5, 72.2, 71.5, 36.4.

(v) Synthesis of compound 14p

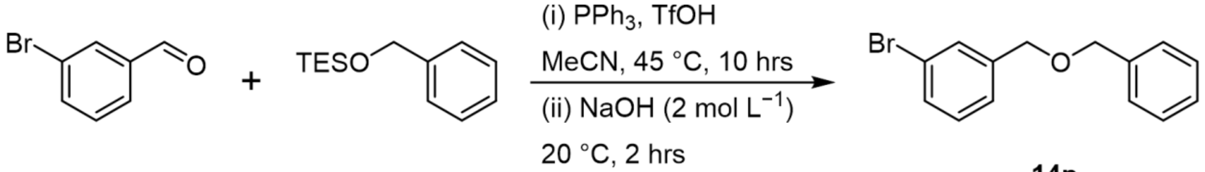

14p

According to General Procedure E, using 3-bromobenzaldehyde (208 mg, 1.13 mmol, 1.00 equivalent), triphenylphosphine (320, 1.22 mmol, 1.08 equivalents) and trifluoromethanesulfonic acid (309 mg, 2.06 mmol, 1.82 equivalents) in acetonitrile (2.0 mL), followed by addition of benzyl(trimethylsilyl) ether (420 mg, 1.89 mmol, 1.67 equivalents). The reaction mixture was stirred at 45 °C for 10 hrs. After the flask was cooled to room temperature, a 2 mol L^{-1} NaOH solution (1.6 mL, 3.2 mmol, 2.8 equivalent) was added and the reaction mixture was stirred for 2 hrs. After work-up, the compound was purified by flash column chromatography (96:4 cyclohexane/EtOAc) to give the product as a colourless oil (260 mg, 0.893 mmol, 89%).

R$_f$ = 0.40

^{1}H NMR (400 MHz, $CDCl_3$) δ 7.54 (app. t, app. *J* = 1.9 Hz, 1H), 7.48 – 7.07 (m, contains C*H*Cl_3 signal and 9H of product), 4.60 – 4.50 (contains 2 × s (overlapping), 4H).[31]

(vi) Synthesis of compound 32

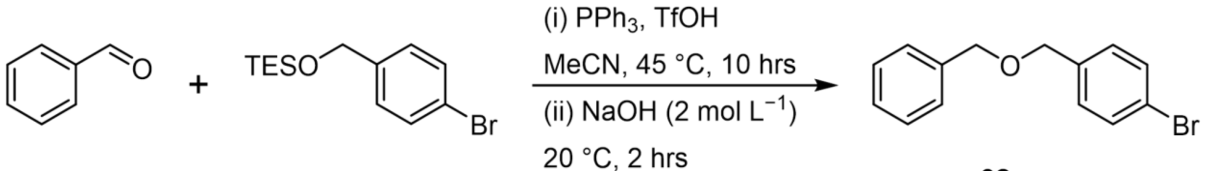

32

According to General Procedure E, using benzaldehyde (112 mg, 1.01 mmol, 1.00 equivalent), triphenylphosphine (289 mg, 1.10 mmol, 1.10 equivalents) and trifluoromethanesulfonic acid (245 mg, 1.63 mmol, 1.61 equivalents) in acetonitrile (2.0 mL), followed by addition of (4-bromobenzyloxy)triethylsilyl ether (579 mg, 1.88 mmol, 1.88 equivalents). The reaction mixture was stirred at 45 °C for 10 hrs. After the flask was cooled to room temperature, a 2 mol L^{-1} NaOH solution (1.6 mL, 3.2 mmol, 3.2 equivalent) was added and the reaction mixture was stirred for 2 hrs. After work-up, the compound was purified by flash column chromatography (96:4 cyclohexane/EtOAc) to give the product as a colourless oil (277 mg, 0.998 mmol, 91%).

$\mathbf{R}_{\boldsymbol{f}}$ = 0.42

^{1}H NMR 1H NMR (400 MHz, $CDCl_3$) δ 7.45 – 7.33 (m, 2H), 7.33 – 7.05 (m, contains C*H*Cl_3 signal and 7H of product), 4.50 – 4.39 (contains 2 × s (overlapping), 4H).[32]

(vii) Synthesis of compound 33

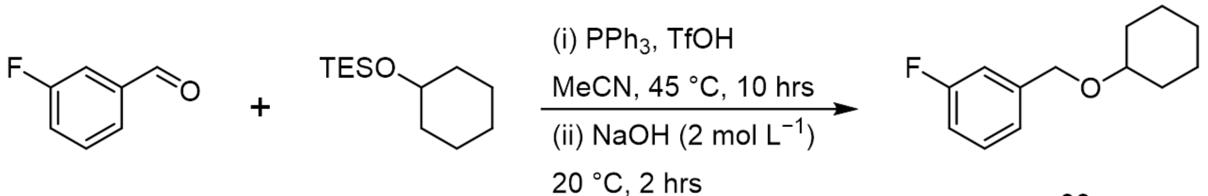

33

According to General Procedure E, using 3-fluorobenzaldehyde (141 mg, 1.14 mmol, 1.00 equivalent), triphenylphosphine (309, 1.18 mmol, 1.03 equivalents) and trifluoromethanesulfonic acid (248 mg, 1.65 mmol, 1.45 equivalents) in acetonitrile (2.0 mL), followed by addition of cyclohexyl(trimethylsilyl) ether (458 mg, 2.14 mmol, 1.87 equivalents). The reaction mixture was stirred at 45 °C for 10 hrs. After the flask was cooled to room temperature, a 2 mol L^{-1} NaOH solution (1.6 mL, 3.2 mmol, 2.7 equivalent) was added and the reaction mixture was stirred for 2 hrs. After work-up, the compound was purified by flash column chromatography (96:4 cyclohexane/EtOAc) to give the product as a colourless oil (216 mg, 1.04 mmol, 85%).

$\mathbf{R}_{f}$ = 0.52

^{1}H NMR (500 MHz, $CDCl_3$) δ 7.46 – 7.20 (m, contains $CHCl_3$ signal and 1H of product), 7.13 – 7.05 (m, 2H), 6.95 (td, *J* = 8.9, 2.8 Hz, 1H), 4.54 (s, 2H), 3.35 (tt, *J* = 9.4, 3.8 Hz, 1H), 2.00 – 1.89 (m, 2H), 1.80 – 1.71 (m, 2H), 1.61 – 1.49 (m, contains H_2O signal and 1H of product), 1.44 – 1.12 (m, 5H).[33]

(viii) Synthesis of compound 34

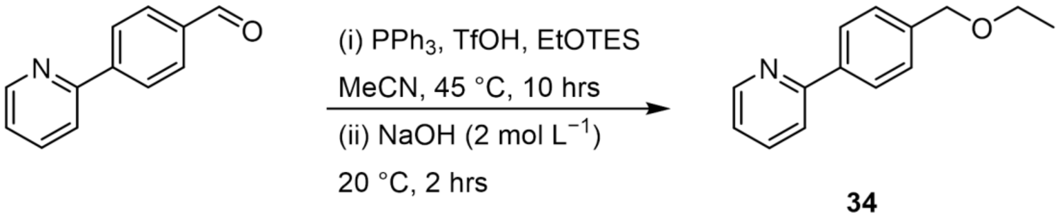

34

According to General Procedure E, using 4-(2-pyridyl)benzaldehyde (189 mg, 1.03 mmol, 1.00 equivalent), triphenylphosphine (343 mg, 1.31 mmol, 1.27 equivalents) and trifluoromethanesulfonic acid (339 mg, 2.26 mmol, 2.19 equivalents) in acetonitrile (2.0 mL), followed by addition of ethyl(triethylsilyl) ether (316 mg, 1.90 mmol, 1.90 equivalents). The reaction mixture was stirred at 45 °C for 10 hrs. After the flask was cooled to room temperature, a 2 mol L^{-1} NaOH solution (1.6 mL, 3.2 mmol, 3.1 equivalent) was added and the reaction mixture was stirred for 2 hrs. After work-up, the compound was purified by flash column chromatography (90:10 cyclohexane/EtOAc) to give the product as a colourless oil (183 mg, 0.856 mmol, 83%).

$\mathbf{R}_f$ = 0.20

IR $\tilde{\nu}_{max}$ (ATR) = 2973 (C—H stretch), 2861 (C—H stretch), 1586 (C=C stretch), 1466 (C—H stretch), 1435 (C=C stretch), 1093 (C—O stretch) cm^{-1}

HRMS (ESI+): Calculated for $[M + H]^+ = [C_{14}H_{15}NO]^+$ *m/z* = 214.1232; found *m/z* = 214.1228

^{1}H NMR (500 MHz, $CDCl_3$) δ 8.68 (dt, *J* = 4.8, 1.3 Hz, 1H), 8.01 – 7.95 (m, 2H), 7.76 – 7.68 (m, 2H), 7.49 – 7.41 (m, 2H), 7.21 (ddd, *J* = 5.9, 4.8, 2.5 Hz, contains C*H*Cl_3 signal and 1H of product), 4.56 (s, 2H), 3.56 (q, *J* = 7.0 Hz, 2H), 1.26 (t, *J* = 7.0 Hz, 3H).

^{13}C NMR (126 MHz, $CDCl_3$) δ 157.2, 149.7, 139.5, 138.6, 136.7, 128.0, 126.9, 122.0, 120.5, 72.4, 65.8, 15.3.

8. Formation of **28** from **21**

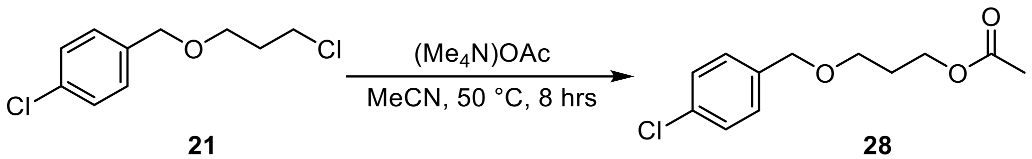

21 **28**

Compound **21** (50 mg, 0.2 mmol, 1.0 equivalent) was added to a round bottom flask equipped with a stir bar. Acetonitrile (1.0 mL) and $(Me_4N)OAc$ (40 mg, 0.3 mmol, 1.5 equivalents) were added and the flask was then closed by addition of a plastic lid. The reaction mixture was then heated in an oil bath at 50 °C for 8 hrs. Upon completion of the reaction, the reaction mixture was added to a 50 mL separatory funnel. A saturated brine solution (5mL) and deionised water (5mL) were added and the mixture was washed with three ethyl acetate portions (10 mL each). The combined organic layers were dried over anhydrous magnesium sulfate and filtered through cotton wool to remove the drying agent and the filtrate was concentrated *in vacuo* to give a colourless, viscous oil.

The ^{1}H NMR spectral conversion to **28** was determined as follows: the residue was dissolved in $CDCl_3$ and aliquot was added to an NMR tube which was then diluted by the addition of $CDCl_3$ (0.6 mL). A ^{1}H NMR spectrum was obtained of the sample. The ^{1}H NMR spectral conversion to **28** was obtained by expressing the integration of one of the signals of **28** in the ^{1}H NMR spectrum of the reaction mixture as a percentage of the sum of the integrations of the signals of interest (i.e., the signal of the protons at position F in the structures shown below for each of **28** and **21**) in the ^{1}H NMR spectrum. An overall 70% ^{1}H NMR spectral conversion to product **28** was obtained using this approach.

HRMS (ESI+): Calculated for $[M + Na]^+ = [C_{12}H_{15}ClO_3Na]^+$ *m/z* = 265.0607; found *m/z* = 265.0603

Signals assigned to **28** in NMR spectra:

^{1}H NMR (400 MHz, $CDCl_3$) δ 7.28 – 7.22 (m, 4H – 2 H_A & 2 H_B), 4.45 (s, 2H, ArCH_2O,), 4.17 (t, J = 6.5 Hz, 2H, CH_2OAc), 3.52 (t, J = 6.2 Hz, 2H, OCH_2CH_2), 2.02 (s, 3H, COCH_3), 1.92 (quint, J = 6.3 Hz, 2H, CH_2C$H_2$$CH_2$).

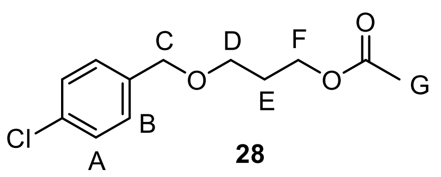

^{13}C NMR (126 MHz, $CDCl_3$) δ 171.2, 136.9, 133.5, 129.0, 128.6, 72.3, 66.9, 61.7, 29.1, 21.1.

Signals assigned to residual **21** in ^{1}H NMR spectrum:

^{1}H NMR (400 MHz, $CDCl_3$) δ 7.35 – 7.22 (m, 4H, 2 H_A & 2 H_B), 4.47 (s, 2H, ArCH_2O), 3.66 (t, J = 6.2 Hz, 2H, OCH_2CH_2), 3.60 (t, J = 5.9 Hz, 2H, CH_2CH_2Cl), 2.04 (quint, J = 6.2 Hz, 2H, CH_2C$H_2$$CH_2$).

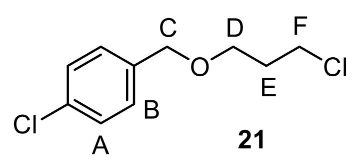

9. Formation of (α-Alkoxyalkyl)phosphonium Mesylate **10H** and Ether **13a** Using Methanesulfonic Acid**(DC8**

Synthesis of (α-alkoxyalkyl)phosphonium mesylate salt 10H

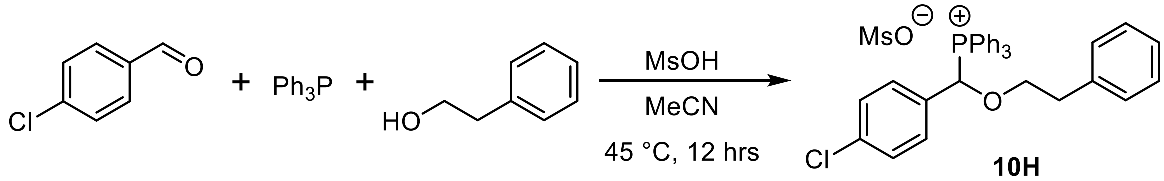

10H

Acetonitrile (2.0 mL) was added to a round bottom flask equipped with a stir bar, followed by triphenylphosphine (290 mg, 1.10 mmol, 1.10 equivalents), **methanesulfonic acid** (214 mg, 2.22 mmol, 2.22 equivalents) and 4-chlorobenzaldehyde (140, 1.00 mmol, 1.00 equivalent). The reaction mixture was stirred at room temperature for **15 minutes**, followed by addition of 2-phenyl-1-ethanol (234 mg, 1.91 mmol, 1.91 equivalents) and the flask was then closed by addition of a plastic lid. The reaction mixture was stirred in a heating bath set to 45 °C for 12 hrs. After the specified time, the reaction mixture was concentrated *in vacuo* and $CDCl_3$ was added to the residue. Upon completion of the reaction, an aliquot was removed from the reaction mixture and added to an NMR tube along with $CDCl_3$ (0.6 mL) for analysis by ^{31}P NMR and ^{1}H NMR spectroscopy. The mixture was analysed and approximate ^{31}P NMR spectral yields were determined using the method reported by Montchamp and co-workers.[7] The ^{31}P NMR spectral yield of **10H** was obtained by expressing the integration of the signal of **10H** in the ^{31}P NMR spectrum of the reaction mixture as a percentage of the sum of the integrations of all of the signals present in the ^{31}P NMR spectrum. The ^{31}P NMR spectral yield for **10H** was thus determined to be 93%.

Signals assigned to **10H** from reaction residue:

^{31}P NMR (162 MHz, $CDCl_3$) spectrum of reaction residue: δ 22.4.

^{1}H NMR (400 MHz, $CDCl_3$) δ 7.82 – 7.75 (m, 3H, *p*-H P—Ph), 7.65 – 7.55 (m, 6H, *o*-H P—Ph), 7.52 – 7.40 (m, 6H, *m*-H P—Ph), 7.06 – 6.94 (m, 2H, *p*-chlorophenyl H), 6.91-6.85 (m, 2H, *p*-chlorophenyl H), 6.65 (d, *J* = 7.9 Hz, 1H, PC*H*), 4.16 – 3.96 (m, 1H, OC*H*),[z] 2.79 (t, *J* = 6.0 Hz, 2H, OCH_2CH_2).

[z] Only one half of the methylene signal (integrating for 1H) is visible in the spectrum. The other half of the signal of the methylene unit is obscured by a signal of residual 2-phenyl-1-ethanol. Thus, unambiguous assignment is not possible.

Hydrolysis of (α-alkoxyalkyl)phosphonium mesylate salt 10H to form ether 13a

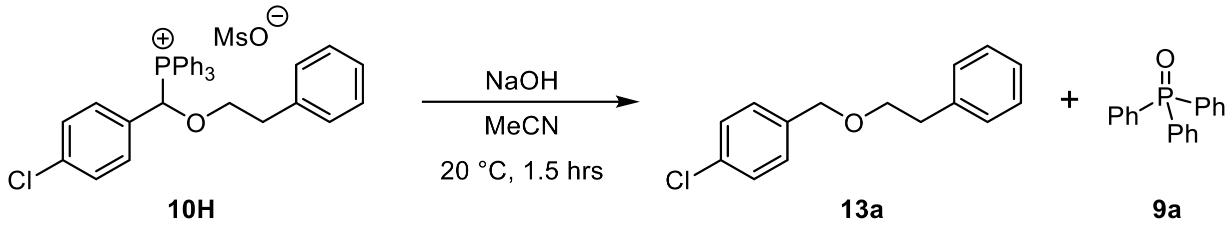

The sample in the NMR tube (including the $CDCl_3$) was added back to the reaction mixture. Acetonitrile (2.0 mL) was added to the residue, which gave a homogeneous solution. A 2 mol L^{-1} solution of NaOH (1.4 mL, 2.8 mmol, 2.8 equivalents) was added to the flask and it was stirred at room temperature for 2 hours. After work-up, the compound was purified by flash column chromatography (95:5 cyclohexane/EtOAc) to give the product as a colourless oil (220 mg, 0.894 mmol, 89%).

R*f* = 0.4.

^{1}H NMR (300 MHz, $CDCl_3$) δ 7.35 – 7.25 (m, 4H), 7.25 – 7.15 (m, 5H), 4.47 (s, 2H), 3.68 (t, *J* = 7.1 Hz, 2H), 2.92 (t, *J* = 7.1 Hz, 2H).[9]

10. Reaction using pre-made $[Ph_3PH]$ OTf

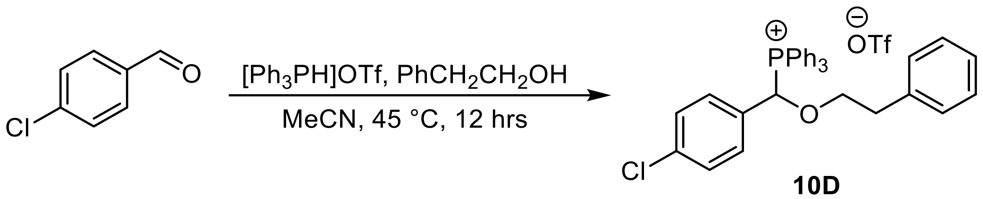

10D

Acetonitrile (2.0 mL) was added to a round bottom flask that was equipped with a stirring bar. To this, the following were added sequentially: $[Ph_3PH]OTf$ (499 mg, 1.21 mmol, 1.21 equivalents) and 4-chlorobenaldehyde (141 mg, 1.00 mmol, 1.00 equivalent). After addition, the contents of the flask were stirred at room temperature. 2-Phenyl-1-ethanol (246 mg, 2.01 mmol, 2.01 equivalents) was then added and the flask was sealed with a plastic lid. The flask was placed in an oil bath set at 45 °C for 12 hours. After the specified time, the reaction mixture was allowed to cool to room temperature and concentrated *in vacuo*, giving a colourless viscous residue. $CDCl_3$ was added to the residue. Upon completion of the reaction, an aliquot was removed from the reaction mixture and added to an NMR tube along with $CDCl_3$ (0.6 mL) for analysis by ^{31}P NMR spectroscopy. An approximate ^{31}P NMR spectral yield of **10D** was determined using the method previously reported by Montchamp and co-workers.[7] The ^{31}P NMR spectral yield of **10D** was obtained by expressing the integration of the signal of **10D** in the ^{31}P NMR spectrum of the reaction mixture as a percentage of the sum of the integrations of all of the signals present in the ^{31}P NMR spectrum. The ^{31}P NMR spectral yield for **10D** was thus determined to be 88%.

^{31}P NMR (162 MHz, $CDCl_3$) δ 22.2 (**10D**), 21.5 (**12D**), 2.5 ($[Ph_3PH]OTf$).

11. Incompatible Substrates

(i) Tertiary alcohols

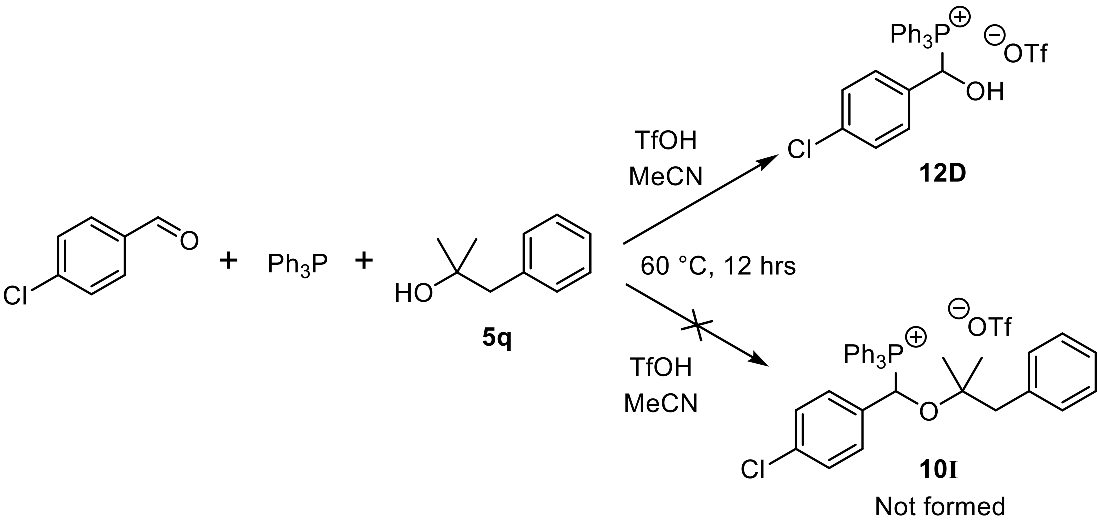

According to General Procedure B, using 4-chlorobenzaldehyde (141 mg, 1.00 mmol, 1.00 equivalent), triphenylphosphine (288, 1.09 mmol, 1.09 equivalents) and trifluoromethanesulfonic acid (203 mg, 1.35 mmol, 1.35 equivalents) in acetonitrile (2.0 mL), followed by addition of 2-methyl-1-phenyl-2-propanol (301 mg, 2.00 mmol, 2.00 equivalents) was then added and the reaction mixture was stirred at 60 °C for 10 hrs. Upon completion of the reaction, an aliquot was removed from the reaction mixture and added to an NMR tube along with $CDCl_3$ (0.6 mL) for analysis by ^{31}P NMR spectroscopy. The mixture was analysed and approximate ^{31}P NMR spectral yields were determined using the method reported by Montchamp and co-workers.[7]

^{31}P NMR (162 MHz, $CDCl_3$) δ 24.1 (relative integration 7%) 21.2 (**12D**, relative integration 67%), −1.8 (Ph_3P + $[Ph_3PH]OTf$, relative integration 26%).[aa]

The predominant compound present in the reaction mixture was determined to be **12D**. No signals that could be assigned to (α-alkoxyalkyl)phosphonium triflate, **10I**, were present. The ^{31}P NMR spectral yield of **12D** was obtained by expressing the integration of the signal of **12D** in the ^{31}P NMR spectrum of the reaction mixture as a percentage of the sum of the integrations of all of the signals present in the ^{31}P NMR spectrum. The ^{31}P NMR spectral yield for **12D** was thus determined to be 67%.

[aa] The compound giving rise to the signal at δ_P 24.1 ppm is a minor side-product of unknown structure, and does not belong to (α-alkoxyalkyl)phosphonium triflate **10I**.

(ii) Phenols

(a) 2,4-Di-tert-butylphenol

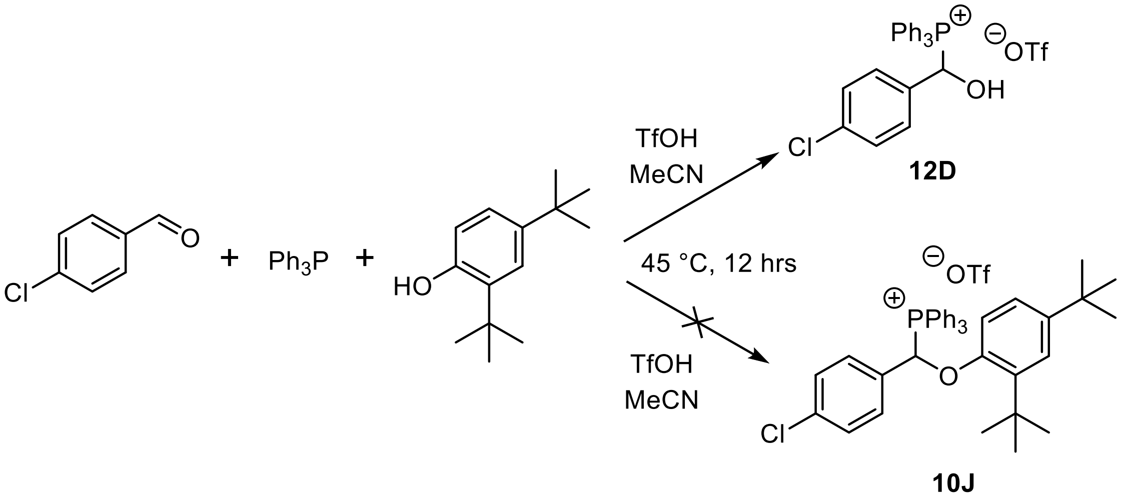

According to General Procedure B, using 4-chlorobenzaldehyde (142 mg, 1.01 mmol, 1.01 equivalent), triphenylphosphine (293, 1.11 mmol, 1.11 equivalents) and trifluoromethanesulfonic acid (200 mg, 1.33 mmol, 1.33 equivalents) in acetonitrile (2.0 mL), followed by addition of 2,4-di-*tert*-butylphenol (406 mg, 1.97 mmol, 1.97 equivalents) was then added and the reaction mixture was stirred at 45 °C for 10 hrs. Upon completion of the reaction, an aliquot was removed from the reaction mixture and added to an NMR tube along with $CDCl_3$ (0.6 mL) for analysis by ^{31}P NMR spectroscopy. The mixture was analysed and approximate ^{31}P NMR spectral yields were determined using the method reported by Montchamp and co-workers.[7] The ^{31}P NMR spectral yield of **12D** was obtained by expressing the integration of the signal of **12D** in the ^{31}P NMR spectrum of the reaction mixture as a percentage of the sum of the integrations of all of the signals present in the ^{31}P NMR spectrum. The ^{31}P NMR spectral yield for **12D** was thus determined to be 70%.

^{31}P NMR (162 MHz, $CDCl_3$) δ 21.5 (**12D**, relative integration 70%), 4.1 ([Ph_3PH]OTf, relative integration 30%).

Unequivocal assignment of the ^{31}P NMR peak at 21.5 ppm to (α-hydroxyalkyl)phosphonium triflate, **12D**, or (α-alkoxyalkyl)phosphonium triflate, **10J**, was not possible. Thus, to verify the species present in the reaction mixture, it was subject to standard hydrolysis conditions as per General Procedure C. A 2 mol L^{-1} solution of NaOH (1.4 mL, 2.8 mmol, 2.8 equivalent) was added to the flask and it was stirred at room temperature for 2 hours. Upon completion of the reaction, the mixture was added to a separatory funnel, ethyl acetate (10 mL) was added and extracted with a saturated brine solution (10 mL). The aqueous layer was extracted twice more with 10 mL portions of ethyl acetate (2 × 10 mL). The combined organic layer was dried over anhydrous magnesium sulfate, filtered and concentrated *in*

vacuo. An aliquot was removed for analysis ^{31}P NMR analysis which showed triphenylphosphine as the major product. This outcome confirms that the signal at δ_P 21.5 ppm in the ^{31}P NMR spectrum of the reaction mixture prior to hydrolysis belongs to (α-hydroxyalkyl)phosphonium triflate, **12D**, as this compound is base-sensitive, and consequently reverts to starting materials under basic conditions.

^{31}P NMR (162 MHz, $CDCl_3$ δ 31.1 (Ph_3PO, relative integration 8%), −4.7 (Ph_3P, relative integration 92%).

(b) 4-Chlorophenol

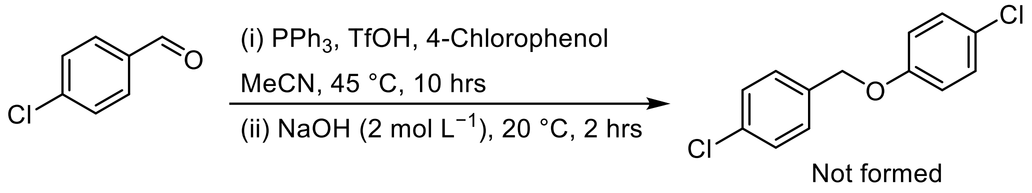

According to General Procedure B, using 4-chlorobenzaldehyde (104 mg, 0.742 mmol, 1.01 equivalent), triphenylphosphine (210, 0.801 mmol, 1.07 equivalents) and trifluoromethanesulfonic acid (161 mg, 1.07 mmol, 1.44 equivalents) in acetonitrile (2.0 mL), followed by addition of 4-chlorophenol (184 mg, 1.43 mmol, 1.92 equivalents) was then added and the reaction mixture was stirred at 45 °C for 10 hrs. After the flask was cooled to room temperature, a 2 mol L−1 NaOH solution (1.4 mL, 2.8 mmol, 2.8 equivalents) was added and the reaction mixture was stirred for 2 h. Upon completion of the reaction, the mixture was diluted in ethyl acetate (10 mL) and extracted with a saturated brine solution (10 mL). The aqueous layer was extracted twice more with 10 mL portions of ethyl acetate. The combined organic layer was dried over anhydrous magnesium sulfate, filtered and concentrated in vacuo. $CDCl_3$ was added to the residue and an aliquot placed in an NMR tube. The ^{31}P NMR spectral yield of Ph_3P was obtained by expressing the integration of the signal of Ph_3P in the ^{31}P NMR spectrum of the reaction mixture as a percentage of the sum of the integrations of all of the signals present in the ^{31}P NMR spectrum, as per the method proposed by Montchamp and co-workers.[7]. The ^{31}P NMR spectral yield for Ph_3P was thus determined to be 88%.

^{31}P NMR (162 MHz, $CDCl_3$) δ 31.9 (Ph_3PO, relative integration 12%), −5.3 (Ph_3P, relative integration 88%).

Conclusion: The signals present in the ^{31}P NMR spectrum were consistent with the formation of Ph_3P (88%) and Ph_3PO (12%), indicating that the targeted ether product was formed in tiny amounts, at most. This is likely to be due to a failure to form the required (α-alkoxyalkyl)phosphonium salt, but may also be explicable by reversal of the (α-alkoxyalkyl)phosphonium salt to starting materials (including Ph_3P) upon exposure to NaOH.

(ii) Aldehydes with *para*-electron donating substituents

(a) Reaction with *para*-anisaldehyde

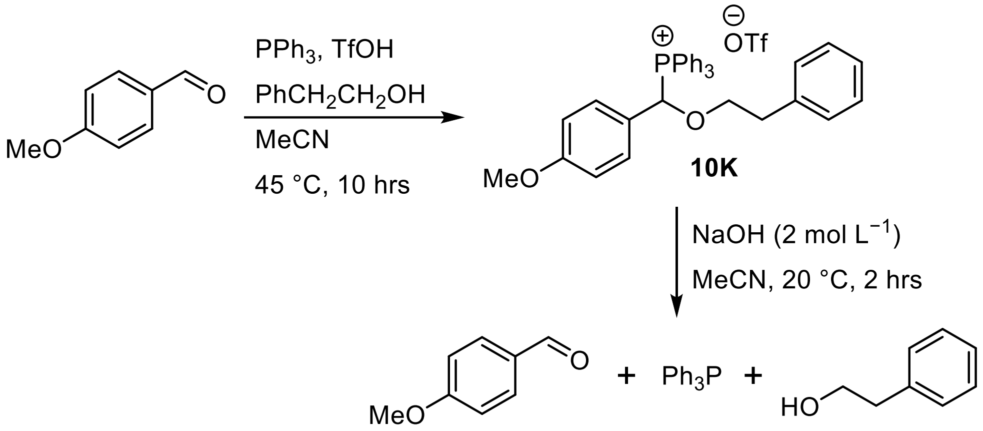

According to General Procedure C, using *para*-anisaldehyde (137 mg, 1.00 mmol, 1.00 equivalent) triphenylphosphine (294 mg, 1.12 mmol, 1.12 equivalent) and trifluoromethanesulfonic acid (197 mg, 1.31 mmol, 1.31 equivalents) in MeCN (2.0 mL). 2-Phenyl-1-ethanol (234 mg, 1.91 mmol, 1.91 equivalents) was then added and the reaction mixture was stirred at 45 °C for 10 hrs. After the flask was cooled to room temperature, a 2 mol L^{-1} NaOH solution (1.4 mL, 2.8 mmol, 2.8 equivalents) was added and the reaction mixture was stirred for 2 hrs. Upon completion of the reaction, the mixture was diluted in ethyl acetate (10 mL) and extracted with a saturated brine solution (10 mL). The aqueous layer was extracted twice more with 10 mL portions of ethyl acetate. The combined organic layer was dried over anhydrous magnesium sulfate, filtered and concentrated *in vacuo*. $CDCl_3$ was added to the residue and an aliquot placed in an NMR tube. The ^{1}H NMR spectrum had no signals that could be assigned to the desired ether product, instead the signals were assigned to the aldehyde and alcohol starting materials. This was further confirmed by ^{31}P NMR spectrum as the predominant peak belonged to triphenylphosphine. This can only reform *via* decomposition of (α-alkoxyalkyl)phosphonium triflate, **10K**, to starting materials.

A ^{31}P NMR spectrum of a sample of the reaction mixture (diluted with $CDCl_3$) recorded after addition of the aldehyde but *prior* to addition of the alcohol showed that (α-hydroxyalkyl)phosphonium triflate **12K** (δ_P 20.7) was the major phosphorus-containing species present in the reaction mixture at this point (along with significant [Ph_3PH]OTf; δ_P 3.9) – see the spectral data given below for details. A ^{31}P NMR spectrum of a sample of the reaction mixture (diluted with $CDCl_3$) recorded *after* addition of the alcohol showed that (α-alkoxyalkyl)phosphonium triflate **10K** (δ_P 21.3) had been formed in 77% conversion (see below for spectral details). Thus, (α-alkoxyalkyl)phosphonium triflate **10K** is clearly formed in this reaction, but exposure of it to hydrolytic conditions leads to regeneration of the parent phosphine, alcohol and aldehyde rather than formation of an ether.

The mixture was analysed and approximate ^{31}P NMR spectral yields were determined using the method reported by Montchamp and co-workers.[7] The ^{31}P NMR spectral yield of a given phosphorus-containing compound was obtained by expressing the integration of the signal of that compound in the ^{31}P NMR spectrum of the reaction mixture as a percentage of the sum of the integrations of all of the signals present in the ^{31}P NMR spectrum. The ^{31}P NMR spectral yields were thus determined to be 57% for **12K** and 77% for **10K**, respectively.

^{31}P NMR (162 MHz, $CDCl_3$) after *aldehyde* addition: δ 20.7 (**10K**, relative integration 57%), 3.9 ([Ph_3PH]OTf, relative integration 43%).

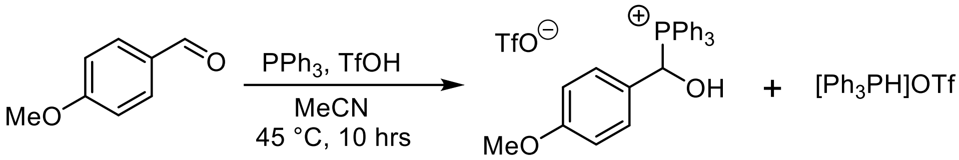

12K, 57% 43%

^{31}P NMR (162 MHz, $CDCl_3$) after *alcohol* addition: δ 21.3 (**10K**, relative integration 77%), 20.7 (**12K**, relative integration 12%), 3.8 ([Ph_3PH]OTf, relative integration 21%).

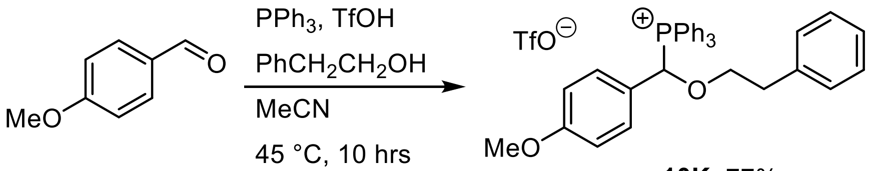

10K, 77%

(b) Reaction with 4-(diphenylamino)benzaldehyde

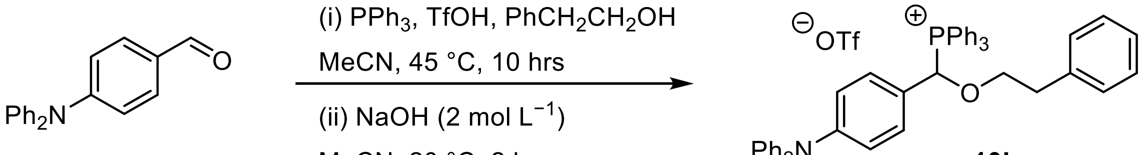

10L

According to General Procedure C, using 4-(diphenylamino)benzaldehyde (274 mg, 1.00 mmol, 1.00 equivalent), triphenylphosphine (295 mg, 1.12 mmol, 1.12 equivalents) and trifluoromethanesulfonic acid (199 mg, 1.32 mmol, 1.32 equivalents) in MeCN (2.0 mL). 2-Phenyl-1-ethanol (300 mg, 2.45 mmol, 2.45 equivalents) was then added and the reaction mixture was stirred at 45 °C for 10 hrs. After the flask was cooled to room temperature, a 2 mol L^{-1} NaOH solution (1.4 mL, 2.8 mmol, 2.8 equivalent) was added and the reaction mixture was stirred for 2 hrs. Upon completion of the reaction, the mixture was diluted in ethyl acetate (10 mL) and extracted with a saturated brine solution (10 mL). The aqueous layer was extracted twice more with 10 mL portions of ethyl acetate. The organic phases were combined, dried over anhydrous magnesium sulfate, filtered and concentrated *in vacuo*. $CDCl_3$

was added to the residue and an aliquot placed in an NMR tube. The ^{1}H NMR spectrum had no peaks that could be assigned to the desired ether product. This was further confirmed by the ^{31}P NMR spectrum as the predominant peak was triphenylphosphine. This can only reform *via* decomposition of (α-alkoxyalkyl)phosphonium triflate), **10L**, to starting materials.

A ^{31}P NMR spectrum of a sample of the reaction mixture (diluted with $CDCl_3$) recorded after addition of the aldehyde but prior to addition of the alcohol showed that (α-hydroxyalkyl)phosphonium triflate **12L** (δ_P 19.8) was the major phosphorus-containing species present in the reaction mixture at this point (along with significant [Ph_3PH]OTf; δ_P 3.6) – see the spectral data given below for details. A ^{31}P NMR spectrum of a sample of the reaction mixture (diluted with $CDCl_3$) recorded after addition of the alcohol showed that (α-alkoxyalkyl)phosphonium triflate **10L** (δ_P 20.8) had been formed in 76% conversion (see below for spectral details). Thus, (α-alkoxyalkyl)phosphonium triflate **10L** is clearly formed in this reaction, but exposure of it to hydrolytic conditions leads to regeneration of the parent phosphine, alcohol and aldehyde rather than formation of an ether.

The mixture was analysed and approximate ^{31}P NMR spectral yields were determined using the method reported by Montchamp and co-workers.[7] The ^{31}P NMR spectral yield of a given phosphorus-containing compound was obtained by expressing the integration of the signal of that compound in the ^{31}P NMR spectrum of the reaction mixture as a percentage of the sum of the integrations of all of the signals present in the ^{31}P NMR spectrum. The ^{31}P NMR spectral yields were thus determined to be 47% for **12L** and 76% for **10-L**, respectively.

^{31}P NMR (162 MHz, $CDCl_3$) after *aldehyde* addition: δ 19.8 (**12L**, relative integration 47%), 3.6 ([Ph_3PH]OTf, relative integration 61%).

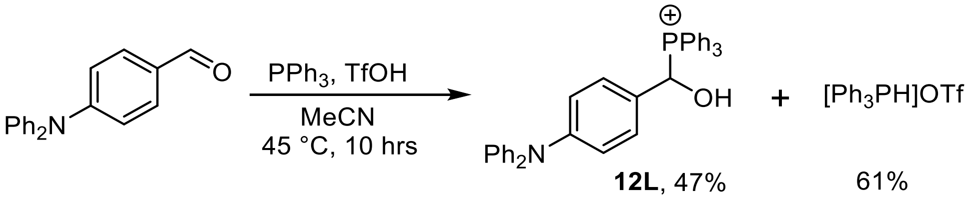

^{31}P NMR (162 MHz, $CDCl_3$) after *alcohol* addition: δ 20.8 (**10L**, relative integration 76%), 20.1 (**18-XII**, relative integration 7%), 3.6 ([Ph_3PH]OTf, 16%) .

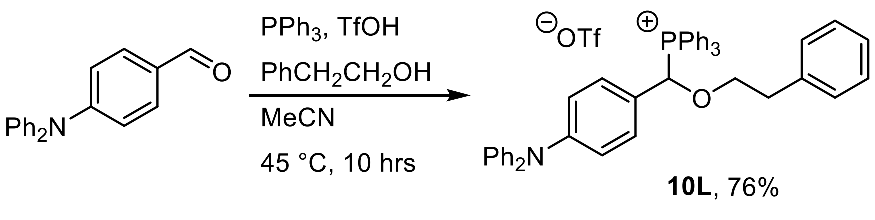

To confirm (α-alkoxyalkyl)phosphonium triflate, **10L**, decomposes to starting materials (i.e., triphenylphosphine, aldehyde and alcohol), 1,3,5-trimethoxybenzene (29 mg, 0.17 mmol, 0.17 equivalents) was added to the residue as an internal standard and a ^{1}H NMR spectral yield for residual alcohol and aldehyde was obtained.

Method for Determining ^{1}H NMR spectral yield

The reaction solvent was not removed subsequent to analysis. 1,3,5-Trimethoxybenzene (10–15 mol%) was weighed into a vial and added to the reaction flask. $CDCl_3$ (*ca*. 0.3 mL) was then added to the vial to dissolve any remaining internal standard, and the resulting solution was also transferred to the reaction flask to ensure quantitative transfer. The mixture was agitated vigorously to ensure that the internal standard was evenly distributed throughout the solution. A small aliquot was then removed (*ca*. 10 drops) which was added to a vial containing $CDCl_3$ (*ca*. 0.6 mL). The contents of the vial were again agitated and then transferred into an NMR tube. The concentration of product in the reaction mixture relative to the known concentration of the internal standard could then be established by determining the relative integrations of the NMR signals of the two species, and hence a ^{1}H NMR spectral yield could be calculated in the manner shown in the example below.

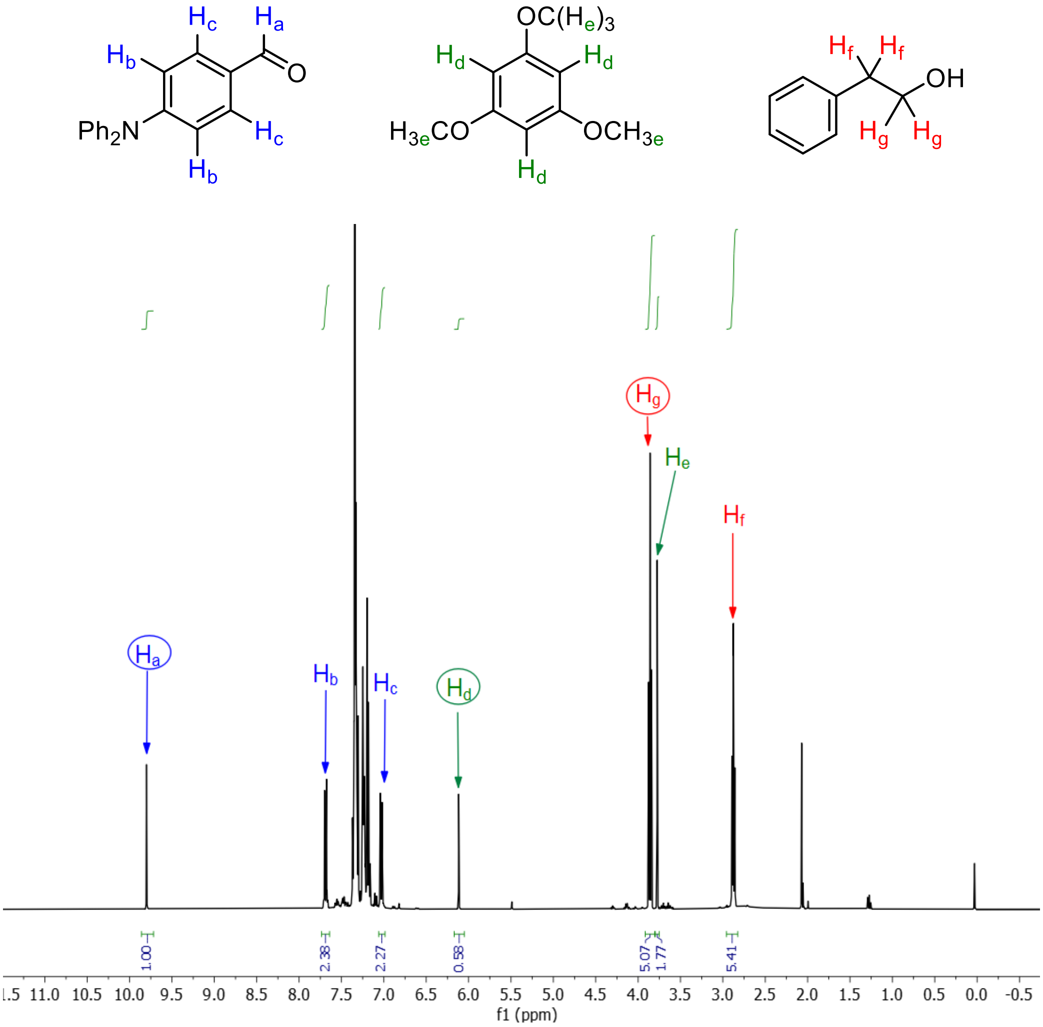

The ^{1}H NMR spectral yield (in mmol) of each compound of interest (aldehyde or alcohol starting material) was determined using the following equation:

$$\frac{\left(\frac{\text{Integration of signal of compound}}{\text{No. of protons contributing to signal}}\right)}{\left(\frac{\text{Integration of signal of I.S.}}{\text{No. of protons contributing to signal}}\right)} \times \left(\frac{\text{Mass of I.S. added (g)}}{M_W \text{ of I.S. (g mol}^{-1}\text{)}}\right) = \text{mmol of compound}$$

I.S. = Internal standard.

Internal standard calculation to quantify yield of aldehyde recovery:

The integration of the aldehyde signal at δ_H 9.80 (corresponding to H_a) was set equal to 1.00, and the integrations of the signals of other compounds present in the sample were expressed relative to this value.

Relative integration of 3H of 1,3,5-trimethoxybenzene = 0.58 (from δ 6.12 ppm (s, $(H_e)_3$). Therefore 1H of 1,3,5-trimethoxybenzene = 0.58/3.

$$= \frac{\left(\frac{1.00}{1}\right)}{\left(\frac{0.58}{3}\right)} \times \left(\frac{29 \text{ mg}}{168.19 \text{ mg mmol}^{-1}}\right) = 0.90 \text{ mmol of aldehyde}$$

0.90 mmol of 4-(diphenylamino)benzaldehyde recovered from 1.0 of mmol 4-(diphenylamino)benzaldehyde added at the start of the reaction = 90% aldehyde regenerated.

Internal standard calculation to quantify yield of alcohol recovery:

Note: The relative integrations for 2-phenyl-1-ethanol are specified relative to 1H of the aldehyde (see above).

Relative integration of 2H of 2-phenyl-1-ethanol (**5a**) = 5.07 (from δ 3.86 ppm (t, *J* = 6.7 Hz, 2H of **5a**, H_g)). Therefore, 1H of alcohol = 5.07/2.

Relative integration of 3H of 1,3,5-trimethoxybenzene = 0.58 (from δ 6.12 ppm (s, $(H_e)_3$). Therefore 1H of 1,3,5-trimethoxybenzene = 0.58/3.

$$\frac{\left(\frac{5.07}{2}\right)}{\left(\frac{0.58}{3}\right)} \times \left(\frac{29 \text{ mg}}{168.19 \text{ mg mmol}^{-1}}\right) = 2.3 \text{ mmol of alcohol}$$

2.3 mol of 2-phenyl-1-ethanol recovered from 2.5 mmol of 2-phenyl-1-ethanol added at the start of the reaction = 92% alcohol regenerated.

12. Control Experiments

Experiment 1: Requirement for acid (TfOH) for formation of 10D

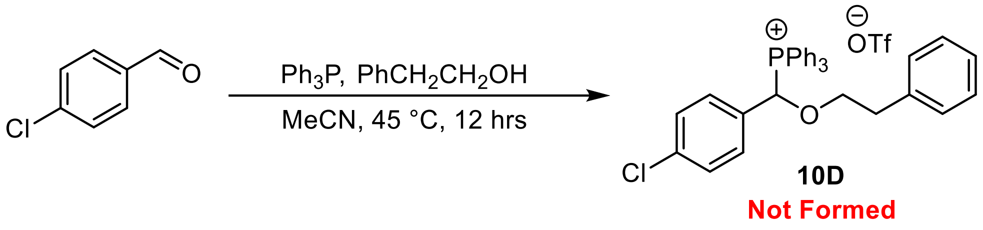

Triphenylphosphine (291 mg, 1.11 mmol, 1.1 equivalents), and 4-chlorobenzaldehyde (139 mg, 0.995 mmol, 0.995 equivalent) were dissolved in MeCN (2 mL). The reaction mixture was stirred at room temperature for 5 minutes, followed by addition of 2-phenyl-1-ethanol (234 mg, 1.91 mmol, 1.91 equivalents) and the flask was then closed by addition of a plastic lid. The reaction mixture was stirred in a heating bath set to 45 °C for 12 hrs. Upon completion of the reaction, an aliquot was removed from the reaction mixture and added to an NMR tube along with $CDCl_3$ (0.6 mL) for analysis by ^{31}P NMR spectroscopy. The mixture was analysed and approximate ^{31}P NMR spectral yields were determined using the method proposed by Montchamp and co-workers.[7] The ^{31}P NMR spectral yield of Ph_3P was obtained by expressing the integration of the signal of Ph_3P in the ^{31}P NMR spectrum of the reaction mixture as a percentage of the sum of the integrations of all of the signals present in the ^{31}P NMR spectrum. The ^{31}P NMR spectral yield for Ph_3P was thus determined to be 95%.

^{31}P NMR (121 MHz, $CDCl_3$) of reaction mixture: δ 28.8 (Ph_3PO,[bb] relative integration 5%), −5.5 (Ph_3P, relative integration, 95%).

Conclusion: In the absence of acid, the reaction does not proceed.

Experiment 2: Reaction of alcohol + 4-chlorobenzaldehyde in the absence of Ph_3P

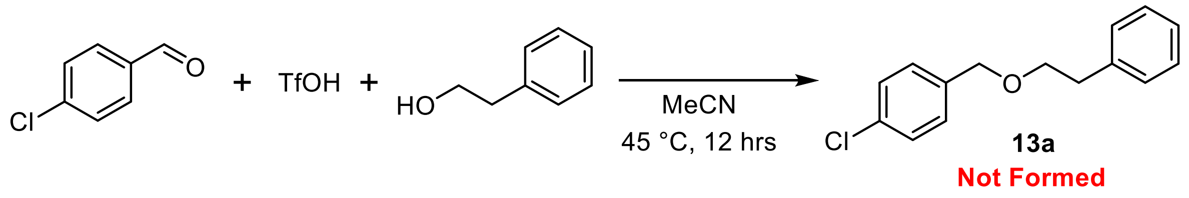

4-Chlorobenzaldehyde (145 mg, 1.03 mmol, 1.03 equivalent) and trifluoromethanesulfonic acid (190 mg, 1.26 mmol, 1.26 equivalents) were dissolved in MeCN (2 mL). The reaction mixture was stirred at room temperature for 5 minutes, followed by addition of 2-phenyl-1-ethanol (244 mg, 2.00 mmol, 2.00 equivalents) and the flask was then closed by addition of a plastic lid. The reaction mixture was stirred

[bb] The presence of Ph_3PO (*ca*. 5%) can be rationalised by the tendency of triphenylphosphine to oxidise in the presence of O_2.

in a heating bath set to 45 °C for 12 hrs. Upon completion of the reaction, an aliquot was removed for analysis by ^{1}H NMR spectroscopy and $CDCl_3$ added to the aliquot.

^{1}H NMR (300 MHz, $CDCl_3$) δ 9.98 (s, 1H), 7.90 – 7.76 (m, 2H), 7.58 – 7.46 (m, 2H), 4.58 (t, *J* = 6.4 Hz, 3H), 3.84 (t, *J* = 6.8 Hz, 3H), 3.12 (t, *J* = 6.4 Hz, 3H), 2.86 (t, *J* = 6.8 Hz, 3H).

Signals assigned to 4-chlorobenzaldehyde: 9.98 (s, 1H, C*H*O), 7.90 – 7.76 (m, 2H, ArC*H*), 7.58 – 7.46 (m, 2H, ArC*H*).

Signals assigned to 2-phenyl-1-ethanol: 3.84 (t, *J* = 6.8 Hz, 3H, OC$H_2$$H_2$), 2.86 (t, *J* = 6.8 Hz, 3H, OC$H_2$$H_2$).

No signals could be assigned to ether **13a**. The following list of signals belong to ether **13a** which were not found in the ^{1}H NMR of the reaction residue: δ 7.35 – 7.25 (m, 4H), 7.25 – 7.15 (m, 5H), 4.47 (s, 2H), 3.67 (t, *J* = 7.2 Hz, 2H), 2.92 (t, *J* = 7.2 Hz, 2H).

The signals at 4.58 (t, *J* = 6.4 Hz) and 3.12 (t, *J* = 6.4 Hz) have equal integration values, and thus are most likely part of the same molecule, however, it is not possible to unambiguously assign these signals.

Conclusion: In the absence of Ph_3P, the reaction does not proceed. In theory, many products could potentially form under the reaction conditions employed. Acid-catalysed alcohol dehydration, acid-mediated alcohol elimination or acetal formation could all occur but none of the signals present in the spectrum could be attributed to these compounds.

Experiment 3: Interception of oxocarbenium ion 6d using Ph_3P

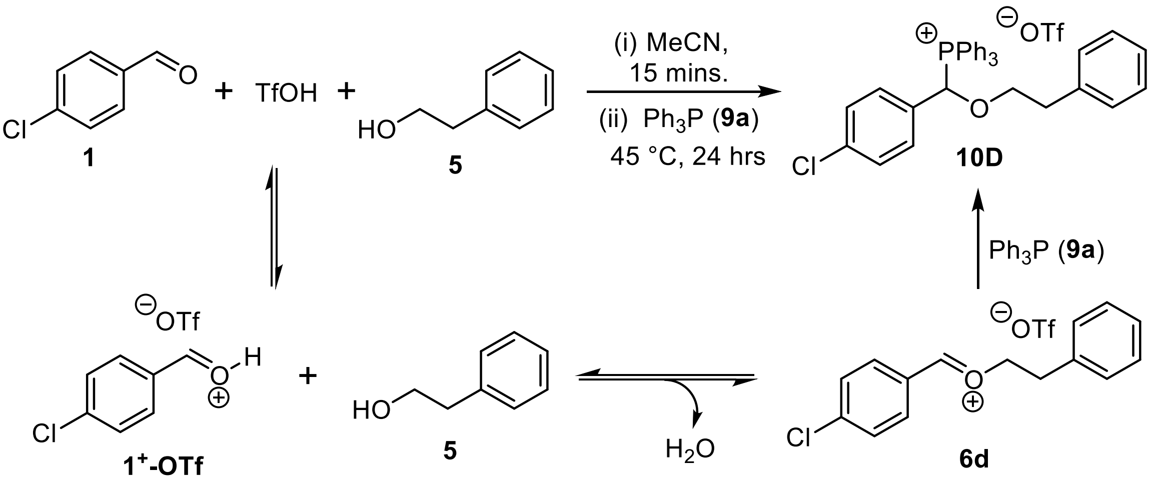

MeCN (2.0 mL) was added to a round bottom flask equipped with a stir bar. This was followed by 4-chlorobenzaldehyde (140, 1.00 mmol, 1.00 equivalent), trifluoromethanesulfonic acid (204 mg, 1.36 mmol, 1.36 equivalents) and 2-phenyl-1-ethanol (229 mg, 1.87 mmol, 1.87 equivalents). The reaction mixture was stirred at room temperature for **15 minutes**, followed by addition of triphenylphosphine

(291 mg, 1.11 mmol, 1.11 equivalents) and the flask was then closed by addition of a plastic lid. The reaction mixture was stirred in a heating bath set to 45 °C for 12 hrs. Upon completion of the reaction, an aliquot was removed from the reaction mixture and added to an NMR tube along with $CDCl_3$ (0.6 mL) for analysis by ^{31}P NMR spectroscopy. The mixture was analysed and approximate ^{31}P NMR spectral yields were determined using the method proposed by Montchamp and co-workers.[7] The ^{31}P NMR spectral yield of **10D** was obtained by expressing the integration of the signal of **10D** in the ^{31}P NMR spectrum of the reaction mixture as a percentage of the sum of the integrations of all of the signals present in the ^{31}P NMR spectrum. The ^{31}P NMR spectral yield for **10D** was thus determined to be 54%.

^{31}P NMR (121 MHz, $CDCl_3$) spectrum of reaction mixture: δ 21.4 (**10D**, relative integration 54%), 20.8 ((α-hydroxyalkyl)phosphonium salt **12D**, relative integration 5%), −5.4 (Ph_3P, relative integration 45%).

Conclusion: The order of addition is important to obtain the maximum yield of (α-alkoxyalkyl)phosphonium triflate **10D**. Competitive acetal formation is likely to have prevented the formation of **10D** from occurring to the extent that is typically observed under standard reaction conditions (see Section 4 above). The intention of this experiment was to purposefully form an oxocarbenium ion which would subsequently be trapped by Ph_3P to furnish the desired **10D**. However, since oxocarbenium ions are only ever formed transiently, delaying the addition of Ph_3P does not enable augmentation of the degree to which oxocarbenium ion is formed, and instead of aiding the trapping of oxocarbenium ion by Ph_3P, the conditions employed for this reaction enable the occurrence of side reactions that would not otherwise take place were Ph_3P present from the outset.

Experiment 4: Importance of Order of Addition for Formation of 10D

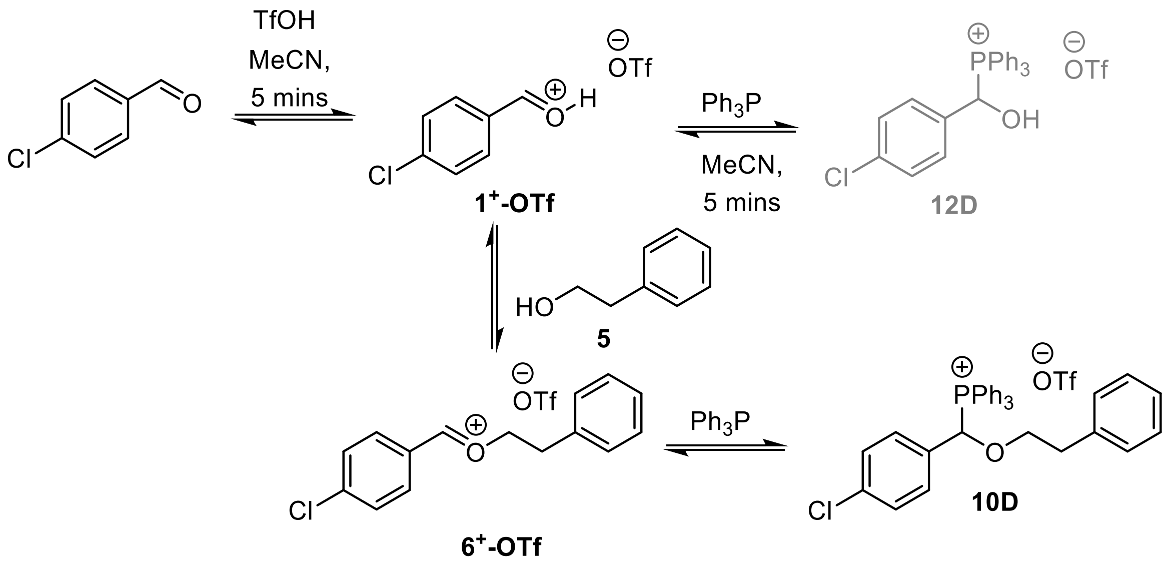

MeCN (2.0 mL) was added to a round bottom flask equipped with a stir bar. This was followed by 4-chlorobenzaldehyde (103, 0.735 mmol, 1.00 equivalent) and trifluoromethanesulfonic acid (133 mg, 0.886 mmol, 1.20 equivalents). The reaction mixture was stirred for 5 minutes. Triphenylphosphine (209 mg, 0.797 mmol, 1.08 equivalents) was added and the reaction was stirred for a further 5 minutes. This was followed by the addition of 2-phenyl-1-ethanol (169 mg, 1.38 mmol, 1.87 equivalents) and the flask was then closed by addition of a plastic lid. The reaction mixture was stirred in a heating bath set to 45 °C for 12 hrs. After the specified time, the reaction mixture was allowed to cool to room temperature and isopropyltripenylphosphonium iodide ([^{i}PrPh$_3$P]I) (95 mg, 0.22 mmol, 0.29 equivalents) was added to the reaction mixture.[cc] An aliquot was removed and $CDCl_3$ added to the aliquot, allowing for ^{31}P NMR and ^{1}H NMR spectral analysis to be carried out. The ^{31}P NMR and ^{1}H NMR spectral yields for **10D** were determined using the method described below.

Method for Determining ^{31}P NMR and ^{1}H NMR spectral yield

The reaction solvent was not removed subsequent to analysis. ([^{i}PrPh$_3$P]I) (20–25 mol%) was weighed into a vial and added to the reaction flask. $CDCl_3$ (*ca*. 0.3 mL) was then added to the vial to dissolve any remaining internal standard, and the resulting solution was also transferred to the reaction flask to ensure quantitative transfer. The mixture was agitated vigorously to ensure that the internal standard was evenly distributed throughout the solution. A small aliquot was then removed (*ca.* 10 drops) which was added to a vial containing $CDCl_3$ (*ca*. 0.6 mL). The contents of the vial were again agitated and then transferred into an NMR tube. The concentration of product in the reaction mixture relative to the known concentration of the internal standard could then be established by determining the relative integrations of the NMR signals of the two species, and hence a ^{31}P NMR and ^{1}H NMR spectral yield could be calculated in the manner shown in the example below.

^{31}P NMR (121 MHz, $CDCl_3$) spectrum of reaction mixture: δ 30.7 ([^{i}PrPh$_3$P]I), 21.6 (**10D**), 20.8 (**12D**).

^{1}H NMR (300 MHz, $CDCl_3$) δ 6.70 (d, *J* = 7.9 Hz, PC*H* of **10D**), 1.37 (dd, *J* = 18.7, 7.0 Hz, 2 × C*H*$_3$ of [^{i}PrPh$_3$P]I).

The ^{1}H and ^{31}P NMR spectral yield (in mmol) of (α-alkoxyalkyl)phosphonium triflate, **10D** was determined using the following equation:

$$\frac{\left(\frac{\text{Integration of signal of compound}}{\text{No. of protons contributing to signal}}\right)}{\left(\frac{\text{Integration of signal of I.S.}}{\text{No. of protons contributing to signal}}\right)} \times \left(\frac{\text{Mass of I.S. added (g)}}{M_W \text{ of I.S. (g mol}^{-1})}\right) = \text{mmol of compound}$$

I.S. = Internal standard.

[cc] The purpose of isopropyltripenylphosphonium iodide ([^{i}PrPh$_3$P]I) was to act as a dual internal standard to quantify the NMR spectroscopic yield by ^{31}P and ^{1}H NMR spectroscopy.

Internal standard calculation to quantify yield of 10D by ^{1}H NMR spectrum:

The integration of the signal of internal standard [^{i}PrPh$_3$P]I at δ_H 1.37 (corresponding to the two sets of methyl group protons) was set equal to 6.00, and the integrations of the signals of other compounds present in the sample were expressed relative to this value.

Relative integration of 1H of **10D** = 2.75 (from δ 6.70 (d, *J* = 7.9 Hz, 1H, PC*H*)).

The ^{1}H NMR spectral yield of **10D** was determined as follows:

$$\frac{\left(\frac{2.75}{1}\right)}{\left(\frac{6}{6}\right)} \times \left(\frac{95 \text{ mg}}{432.28 \text{ mg mmol}^{-1}}\right) = 0.61 \text{ mmol of } \mathbf{10D}$$

$$\left(\frac{0.61 \text{ mmol}}{0.73 \text{ mmol}}\right) = 83\% \text{ yield of } \mathbf{10D}$$

Internal standard calculation to quantify yield of 10D by ^{31}P NMR spectrum:

The integration of the signal of internal standard [^{i}PrPh$_3$P]I at δ_P 30.7 was set equal to 1.00, and the integrations of the signals of other compounds present in the sample were expressed relative to this value.

Relative integration of 1P of **10D** = 2.66 (from δ 21.6).

The ^{31}P NMR spectral yield of **10D** was determined as follows:

$$= \frac{\left(\frac{2.66}{1}\right)}{\left(\frac{1}{1}\right)} \times \left(\frac{95 \text{ mg}}{432.28 \text{ mg mmol}^{-1}}\right) = 0.59 \text{ mmol of } \mathbf{10D}$$

$$\left(\frac{0.59 \text{ mmol}}{0.73 \text{ mmol}}\right) = 81\% \text{ yield of } \mathbf{10D}$$

Conclusion: In this experiment, the aldehyde and acid were mixed together first and then a small delay was left prior to addition of Ph$_3$P, and then a further small delay was left prior to adding the alcohol. While formation of **10D** occurred is comparatively high yield, the amount formed was lower than was observed to occur for the same reaction using the optimised conditions. Hence, the order of addition is important to obtain the maximum yield of **10D**.

13. Mechanistic Investigations

Experiment 1: Formation of (α-hydroxyalkyl)phosphonium salt **12D** in the absence of alcohol, followed by addition of alcohol to form (α-hydroxyalkyl)phosphonium salt **10D**

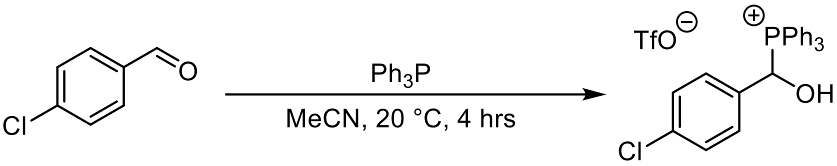

12D

Triphenylphosphine (210 mg, 0.80 mmol, 1.11 equivalents) and trifluoromethanesulfonic acid (131 mg, 0.87 mmol, 1.12 equivalents) were dissolved in MeCN (2 mL). 4-Chlorobenzaldehyde (103 mg, 0.73 mmol, 1.00 equivalents) was then added. The reaction mixture was stirred at room temperature for 4 hours. After this time, an aliquot was removed from the reaction mixture and added to an NMR tube along with $CDCl_3$ (0.6 mL) for analysis by ^{31}P NMR spectroscopy. An approximate ^{31}P NMR spectral yield of 84% was calculated for (α-hydroxyalkyl)phosphonium salt **12D** using the relative integrations of the signals in the ^{31}P NMR spectrum of the reaction mixture, as per the method proposed by Montchamp and co-workers.[7]

^{31}P NMR (121 MHz, $CDCl_3$) spectrum of reaction mixture: δ 20.9 (**12D**, relative integration 84%), 3.8 ([Ph_3PH]OTf, relative integration 16%).

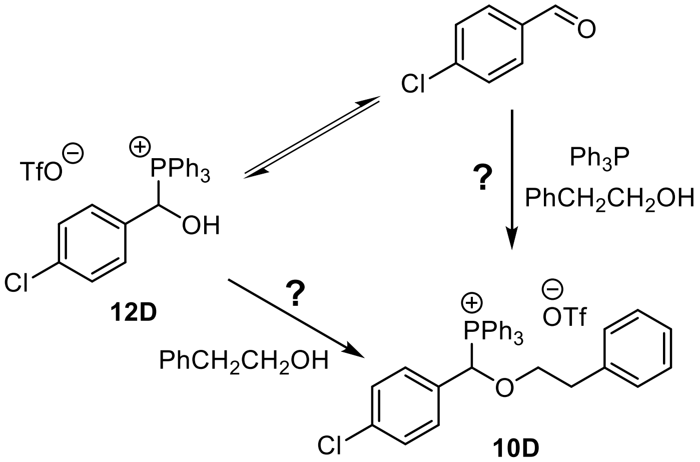

The contents of the NMR tube were added back to the reaction mixture along with 2-phenyl-1-ethanol (156 mg, 1.28 mmol, 1.75 equivalents) and the reaction mixture was stirred in a heating bath set to 45 °C for 24 hrs. After this time, an aliquot was removed from the reaction mixture and added to an NMR tube along with $CDCl_3$ (0.6 mL) for analysis by ^{31}P NMR spectroscopy. An approximate ^{31}P NMR spectral yield of 90% was calculated for (α-alkoxyalkyl)phosphonium salt **10D** using the relative integrations of the signals in the ^{31}P NMR spectrum of the reaction mixture, as per the method proposed by Montchamp and co-workers.[7]

^{31}P NMR (121 MHz, $CDCl_3$) spectrum of reaction mixture: δ 21.4 (**10D**, relative integration 90%), 20.9 (**12D**, relative integration 10%).

Experiment 2: Determination of the relative favourability of (α-alkoxyalkyl)phosphonium salt and (α-hydroxyalkyl)phosphonium salt formation at an early time-point of a reaction in toluene

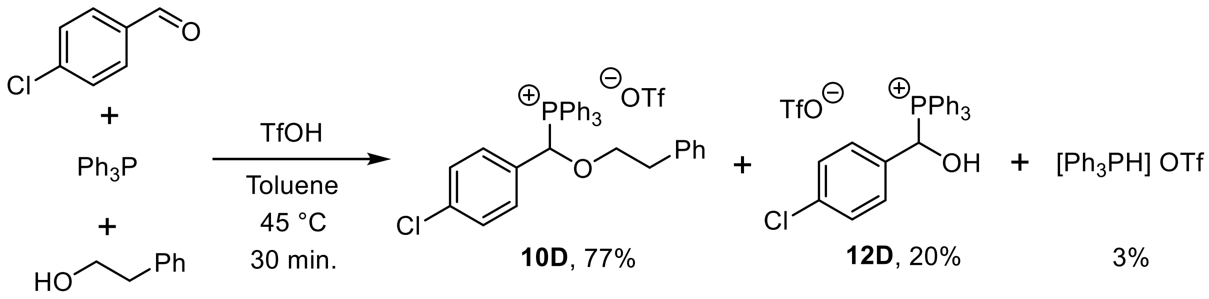

Toluene (2.0 mL) was added to a round bottom flask that was equipped with a stirring bar. To this, the following were added sequentially: triphenylphosphine (212 mg, 0.809 mmol, 1.10 equivalents), trifluoromethanesulfonic acid (158 mg, 1.05 mmol, 1.32 equivalents) and 4-chlorobenzaldehyde (111 mg, 0.792 mmol, 1.00 equivalent) and the contents of the flask were stirred at room temperature. 2-Phenyl-1-ethanol (178.1 mg, 1.46 mmol, 1.84 equivalents) was then added and the flask was sealed with a plastic lid. The flask was placed in an oil bath set at 100 °C for 30 minutes. Upon completion of the reaction, an aliquot was removed from the reaction mixture and added to an NMR tube along with $CDCl_3$ (0.6 mL) for analysis by ^{31}P NMR spectroscopy. Approximate ^{31}P NMR spectral yields were determined using the method proposed by Montchamp and co-workers,[7] i.e., by expressing the integration of the signal of a given compound in the ^{31}P NMR spectrum of the reaction mixture as a percentage of the sum of the integrations of all of the signals present in the ^{31}P NMR spectrum. The ^{31}P NMR spectral yield for **10D** was thus determined to be 77% after 30 minutes of the reaction.

^{31}P NMR (121 MHz, $CDCl_3$ δ 22.0 (**10D**, relative integration 77%), 21.0 (**12D**, relative integration 20%), 3.7 ([Ph_3PH]OTf, relative integration 3%).

Conclusion: While formation of (α-hydroxyalkyl)phosphonium salt **12D** does occur, even at an early time-point in the reaction, (α-alkoxyalkyl)phosphonium salt **10D** is the predominant species formed.

Experiment 3: Determination of the relative favourability of (α-alkoxyalkyl)phosphonium salt and (α-hydroxyalkyl)phosphonium salt formation at an early time-point of a reaction in MeCN

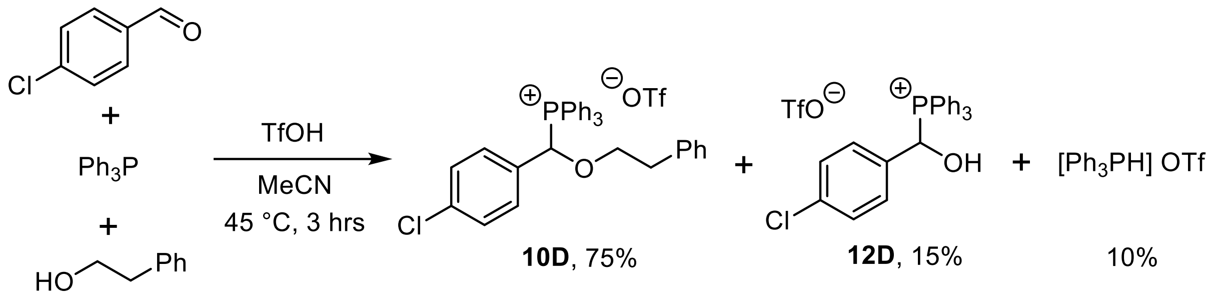

MeCN (2.0 mL) was added to a round bottom flask that was equipped with a stirring bar. To this, the following were added sequentially: triphenylphosphine (214 mg, 0.810 mmol, 1.10 equivalents), trifluoromethanesulfonic acid (146 mg, 0.973 mmol, 1.20 equivalents) and 4-chlorobenzaldehyde (104 mg, 0.741 mmol, 1.0 equivalent) and the contents of the flask were stirred at room temperature. 2-Phenyl-1-ethanol (148.1 mg, 1.21 mmol, 1.63 equivalents) was then added and the flask was sealed with a plastic lid. The flask was placed in an oil bath set at 45 °C for 3 hours. Upon completion of the reaction, an aliquot was removed from the reaction mixture and added to an NMR tube along with $CDCl_3$ (0.6 mL) for analysis by ^{31}P NMR spectroscopy. Approximate ^{31}P NMR spectral yields were determined using the method proposed by Montchamp and co-workers,[7] i.e., by expressing the integration of the signal of a given compound in the ^{31}P NMR spectrum of the reaction mixture as a percentage of the sum of the integrations of all of the signals present in the ^{31}P NMR spectrum. The ^{31}P NMR spectral yield for **10D** was thus determined to be 75% after 3 hours of the reaction.

^{31}P NMR (121 MHz, $CDCl_3$ δ 21.4 (**10D**, relative integration 75%), 20.9 (**12D**, relative integration 16%), 3.7 ([Ph_3PH]OTf, relative integration 10%).

Conclusion: While formation of (α-hydroxyalkyl)phosphonium salt **12D** does occur, even at a relatively early time-point in the reaction, (α-alkoxyalkyl)phosphonium salt **10D** is the predominant species formed.

Experiment 4: Formation of **10D** in the presence of a radical trap

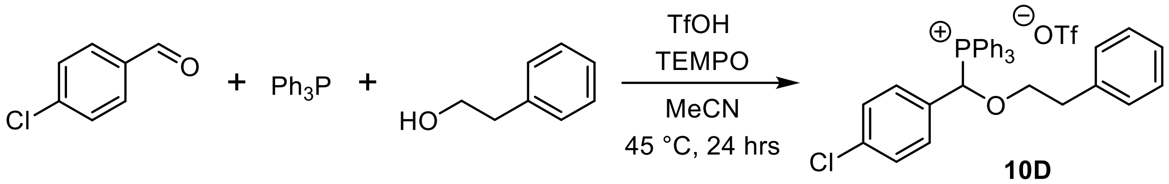

10D

MeCN (2.0 mL) was added to a round bottom flask that was equipped with a stirring bar. To this, the following were added sequentially: triphenylphosphine (212 mg, 0.809 mmol, 1.10 equivalents), trifluoromethanesulfonic acid (170 mg, 1.13 mmol, 1.54 equivalents) and 4-chlorobenzaldehyde (108, 0.771 mmol, 1.04 equivalent) and the contents of the flask were stirred at room temperature. 2-Phenyl-1-ethanol (128 mg, 1.05 mmol, 1.43 equivalents) and 2,2,6,6-tetramethyl-1-piperidinyloxy (TEMPO) (121 mg, 0.775 mmol, 1.00 equivalent) were then added and the flask was sealed with a plastic lid. The flask was placed in an oil bath set at 45 °C for 12 hours. Upon completion of the reaction, an aliquot was removed from the reaction mixture and added to an NMR tube along with $CDCl_3$ (0.6 mL) for analysis by ^{31}P NMR spectroscopy. The mixture was analysed and approximate ^{31}P NMR spectral yields were determined using the method proposed by Montchamp and co-workers.[7] The ^{31}P NMR spectral yield of **10D** was obtained by expressing the integration of the signal of **10D** in the ^{31}P NMR spectrum of the reaction mixture as a percentage of the sum of the integrations of all of the signals present in the ^{31}P NMR spectrum. The ^{31}P NMR spectral yield for **10D** was thus determined to be 63%.

^{31}P NMR (121 MHz, $CDCl_3$ δ 30.9 (Ph_3PO, relative integration 32%), 21.5 (**10D**, relative integration 63%), 20.8 (**12D**, relative integration 5%).

Conclusion: Conversion to **10D** still occurs in the presence of a radical trap. If the reaction were to be a radical-mediated process, the presence of a radical trap should quench the reaction and prevent the formation the **10D**.

Experiment 5: Independent generation of O-alkyloxocarbenium ion from an acetal, and capture of the O-alkyloxocarbenium ion to generate (α-alkoxyalkyl)phosphonium salt.

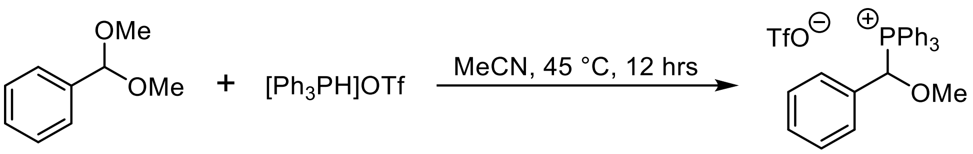

10F

MeCN (2.0 mL) was added to a round bottom flask equipped with a stir bar. This was followed by $[Ph_3PH]OTf$ (500 mg, 1.21 mmol, 1.21 equivalents) and benzaldehyde dimethyl acetal (153 mg, 1.00 mmol, 1.00 equivalent). The flask was then closed by addition of a plastic lid. The reaction mixture was stirred in a heating bath set to 45 °C for 12 hrs. Upon completion of the reaction, solvent was removed *in vacuo* and a viscous colourless oil remained. A small quantity (approximately 0.5 mg) of this was removed for HRMS analysis. Diethyl ether (3 mL) was added to the residue and mixture was stirred for 30 mins. A white solid precipitated which was filtered and washed with two portions of cold diethyl ether (5 mL). Upon completion of the reaction, an aliquot was removed from the reaction mixture and added to an NMR tube along with $CDCl_3$ (0.6 mL) for analysis by ^{31}P NMR and ^{1}H NMR spectroscopy. The mixture was analysed and approximate ^{31}P NMR spectral yields were determined using the method proposed by Montchamp and co-workers.[7] The ^{31}P NMR spectral yield of **10F** was obtained by expressing the integration of the signal of **10F** in the ^{31}P NMR spectrum of the reaction mixture as a percentage of the sum of the integrations of all of the signals present in the ^{31}P NMR spectrum. The ^{31}P NMR spectral yield for **10F** was thus determined to be 93% with the remaining phosphorus-containing material determined to be 7% for **12F**.

The following signals were assigned to (α-alkoxyalkyl)phosphonium triflate **10F** in the residue remaining after solvent removal:

^{31}P NMR (162 MHz, $CDCl_3$) spectrum of reaction mixture: δ 22.0 (**10F**, relative integration 93%), 21.3 (**12F**, relative integration 7%).

^{1}H NMR (400 MHz, $CDCl_3$) spectrum of reaction mixture:

Assigned to **10F**: δ 6.65 (d, J = 7.8 Hz, 1H, PC*H*), 3.51 (s, 3H, OCH_3).

Assigned to benzaldehyde: δ 10.01 (s, 0.16H, C*H*O (1H of benzaldehyde)).

HRMS (ESI+): Calculated for $[M]^+ = [C_{26}H_{24}OP]^+$ m/z = 383.1564; found m/z = 383.1559.

Conclusion: The formation of compound **10F** was confirmed (see further details below). To verify the formation of (α-alkoxyalkyl)phosphonium salt **10F** and to establish that methyltriphenylphosphonium triflate, **38-OTf**, had not occurred, a series of verification experiments were undertaken (see below).

Experiment 5a: Spike experiment involving addition of [$MePh_3P$]OTf, **38-OTf**

[$MePh_3P$]OTf (25 mg, 0.050 mmol) was added to the NMR sample that was prepared in the experiment described just above (Experiment 2). The sample was analysed by ^{31}P and ^{1}H NMR spectroscopy.

In addition to the signals at δ_P 22.0 (**10F**) and δ_P 21.3 (**12F**) that were present prior to spiking with **38-OTf**, after spiking, a third signal appeared (at δ_P 20.6) in the ^{31}P NMR spectrum of the sample which can be assigned to [$MePh_3P$]OTf (**38-OTf**). A new signal characteristic of the *P*-methyl group protons of **38-OTf** also appeared in the ^{1}H NMR spectrum of this sample.

^{31}P NMR (162 MHz, $CDCl_3$) δ 20.6.[34]

^{1}H NMR (400 MHz, $CDCl_3$) δ 2.85 (d, *J* = 13.3 Hz, 3H).[34]

Conclusion: The appearance of a new doublet at δ_H 2.85 in the ^{1}H NMR spectrum and a new signal at δ_P 20.6 in the ^{31}P NMR spectrum arose due to the addition of [$MePh_3P$]OTf to the sample. Since these signals are distinct to those assigned to **10F**, this proves that [$MePh_3P$]OTf was not formed in the experiment described above.

Experiment 5b: Formation of **10F** from PhCHO + MeOH + Ph_3P + TfOH (standard conditions)

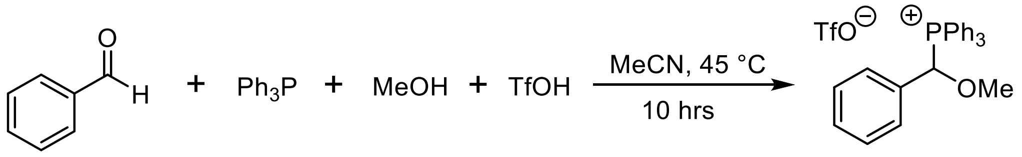

10F

This reaction was carried out according to General Procedure A, using benzaldehyde (112 mg, 1.05 mmol, 1.05 equivalent), triphenylphosphine (288 mg, 1.09 mmol, 1.09 equivalents) and trifluoromethanesulfonic acid (187 mg, 1.24 mmol, 1.42 equivalents). MeOH (61 mg, 1.90 mmol, 1.90 equivalents) was then added and the reaction mixture was stirred at 45 °C for 10 hrs. The reaction mixture was concentrated *in vacuo* to give a viscous colourless oil. A small quantity (approximately 0.5 mg) of this was removed for HRMS analysis. $CDCl_3$ was then added to the residue. An aliquot was removed from the reaction residue and added to an NMR tube along with further $CDCl_3$ (0.6 mL) for analysis by ^{31}P NMR spectroscopy. Approximate ^{31}P NMR spectral yields of the phosphorus-containing compounds present were determined using the method proposed by Montchamp and co-workers.[7] The ^{31}P NMR spectral yield of **10F** was obtained by expressing the integration of the signal of **10F** in the

^{31}P NMR spectrum of the reaction mixture as a percentage of the sum of the integrations of all of the signals present in the ^{31}P NMR spectrum. The ^{31}P NMR spectral yield for **10F** was thus determined to be 91%.

^{31}P NMR (162 MHz, $CDCl_3$) spectrum of reaction mixture: δ 22.0 (**10F**, relative integration 91%), 21.4 ((α-hydroxyalkyl)phosphonium salt **12F**, relative integration 9%).

A ^{1}H NMR spectrum of the reaction mixture was also obtained, and the following signals consistent with the presence of (α-alkoxyalkyl)phosphonium salt **10F** as the major product in the reaction mixture were observed:

^{1}H NMR (400 MHz, $CDCl_3$) spectrum of residue (integrations are expressed relative to 1H of **10F**):
Assigned to **10F**: δ 6.65 (d, *J* = 7.8 Hz, 1H, PC*H*), 3.50 (s, 3H, OCH_3).
Assigned to benzaldehyde: δ 10.01 (s, 0.34H, C*H*O (1H of benzaldehyde)).

A high resolution mass spectrum of the residue remaining after solvent removal also indicated the presence of **10F**.

HRMS (ESI+): Calculated for $[M]^+ = [C_{26}H_{24}OP]^+$ *m/z* = 383.1564; found *m/z* = 383.1559.

Conclusion: The formation of (α-methoxyalkyl)phosphonium salt **10F** was achieved using the General Procedure employed in this methodology. ^{31}P and ^{1}H NMR spectroscopic analyses of the crude product gave analytical data characteristic of compound **10F**. The fact that both this reaction and the reaction of benzaldehyde dimethyl acetal with $[Ph_3PH]OTf$ (experiment 2 above) result in the formation of (α-methoxyalkyl)phosphonium salt **10F** strongly supports that hypothesis that both reactions occur *via* a common O-alkyloxocarbenium ion intermediate.

14. Computational Details

Density functional theory (DFT) calculations were performed using Gaussian 016, Revision B.01.[35] DFT geometry optimizations were carried out in unconstrained *C*1 symmetry and were refined to meet standard convergence criteria. Transition state geometries were optimized using the standard Gaussian algorithm (TS keyword). The curvature of all stationary points was checked by a normal mode frequency calculation. The number of imaginary frequencies was verified to be exactly 0 for local minima and exactly 1 for transition states. The energy reported refer to the most stable conformation (the global minimum) of each intermediate and transition state. DFT calculations of the initial guess ion-pair ensemble structures were performed using the dispersion-corrected ωb97XD hybrid functional,[36] combined with the 6-31G(d,p) basis set. The single-point energy was then calculated for all optimised geometries using the def2-TZV basis set.[37] The thermal corrections were combined with the single point energies calculated at the ωb97XD/def2-TZV level of theory to yield Gibbs free energies at 298.15 K. All energies are reported in kcal mol^{-1}. The energy profiles were constructed using the most stable conformation (the global minimum) of each intermediate and transition state. Geometry optimizations, frequency and thermochemistry calculations, as well as single-point calculations were performed using the polarizable continuum model (PCM) with standard parameterization.

Table S6. Energies (in Hartree) of optimised structures.

Code	Ensemble	Electronic energy *E* (ωB 97XD/def2TZV)	Free energy *G* (*E* + ωB97XD/6-31G(d,p) corrections)	Imaginary Freq. ωB97XD/6-31G(d,p)
**Starting materials**				
none	TfOH	−961.701233	−961.695007	none
none	$[Ph_3PH]OTf$	−1997.785818	−1997.529403	none
none	PhCHO	−345.467235	−345.386527	none
none	MeOH	−115.694738	−115.665495	none

Table S6 continued:

**Hemiacetal mechanism**				
**40**	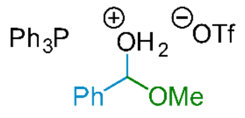	−2458.982889	−2458.570459	none
**6a**	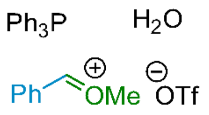	−2458.963201	−2458.557994	none
**41**	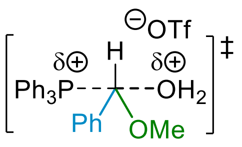	−2458.96112	−2458.545686	−71.51
**α-Hydroxyphosphonium salt mechanism**				
**12F**	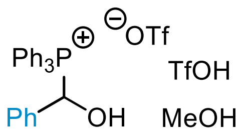	−3420.730582	−3420.286039	none
**43**	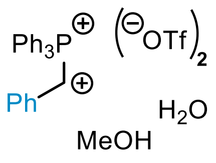	−3420.657872	−3420.218912	none
**44**	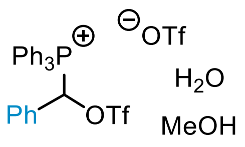	−3420.714939	−3420.273109	none
**45**	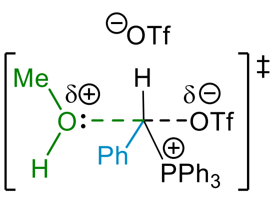	−3420.67821	−3420.234253	−276.09

15. DFT Calculation Data

Atom coordinates:

TfOH

C	−1.00209	0.012454	−0.00194
S	0.845841	−0.13622	0.061277
O	1.242515	−0.00263	1.444838
O	1.208391	−1.27763	−0.74071
O	1.264106	1.162083	−0.7641

[Ph_3PH]OTf

C	0.504639	−0.80642	1.344893
C	−0.40481	−2.09747	3.621607
C	1.174794	-0.61981	2.557686
C	−0.62457	−1.62662	1.267359
C	−1.07663	−2.26992	2.413453
C	0.717257	−1.27434	3.695554
H	2.039414	0.034503	2.616135
H	−1.15606	−1.73911	0.328281
H	−1.95872	−2.89837	2.363606
H	1.231568	−1.13558	4.639902
H	−0.76236	−2.60081	4.513646
C	2.358617	−1.01792	−0.95374
C	4.350823	−2.47728	−2.22051
C	3.090554	−1.96299	−0.23027
C	2.619091	−0.80329	−2.31231
C	3.61689	−1.53686	−2.94158
C	4.089137	−2.69033	−0.86985
H	2.882141	−2.1352	0.820622
H	2.047957	−0.07261	−2.87671
H	3.819304	−1.37685	−3.99465
H	4.658753	−3.42536	−0.31237
H	5.128422	−3.04878	−2.71593

P	1.095633	−0.01668	−0.15308
C	1.741648	1.623965	0.200659
C	2.700055	4.145979	0.837163
C	3.120501	1.840363	0.273202
C	0.836379	2.664288	0.443448
C	1.325669	3.926014	0.759449
C	3.595471	3.107345	0.595929
H	3.817872	1.03243	0.076462
H	−0.23318	2.482124	0.381131
H	0.632791	4.739562	0.943961
H	4.664001	3.281997	0.654296
H	3.074761	5.133517	1.084955
C	−4.13084	−0.32851	−0.42093
S	−2.73028	0.665228	−1.09774
O	−3.38266	1.621318	−1.9973
O	−2.10992	1.25153	0.103134
O	−1.88934	−0.34764	−1.76857
F	−3.689	−1.26935	0.419819
F	−4.98278	0.455305	0.242832
F	−4.79533	−0.93248	−1.40816
H	−0.00531	0.074915	−1.019

PhCHO

C	2.209872	−0.2476	0.000012
C	1.728757	1.059777	0.000004
C	0.356809	1.289265	−1.2E-05
C	−0.53157	0.211202	−2.2E-05
C	−0.04388	−1.10031	−1.3E-05
C	1.324852	−1.3278	0.000004
H	3.279993	−0.42823	0.000025
H	2.420797	1.894971	0.00001
H	−0.02893	2.305331	−1.9E-05
H	−0.75058	−1.9237	−0.00002
H	1.708108	−2.34267	0.00001
C	−1.98593	0.469689	−4.3E-05

O	−2.83908	−0.39652	0.000047
H	−2.27019	1.541161	0.000049

MeOH

C	0.660281	−0.01896	0
H	1.086797	0.986731	−1E-06
H	1.028511	−0.54572	0.890345
H	1.028511	−0.54572	−0.89035
O	−0.74734	0.122391	0

Ensemble of 40

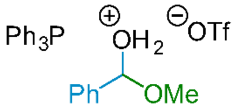

C	−1.29165	2.629795	−0.38086
C	−1.27335	5.409536	−0.67196
C	−1.87515	3.446476	0.594601
C	−0.69463	3.219827	−1.49838
C	−0.68882	4.604946	−1.64566
C	−1.86502	4.82935	0.449419
H	−2.33572	2.998376	1.470147
H	−0.22481	2.594196	−2.25165
H	−0.22234	5.05386	−2.51642
H	−2.31821	5.455612	1.211061
H	−1.26498	6.489079	−0.78294
C	−3.02678	0.337232	−0.64414
C	−5.60468	−0.53214	−1.31135
C	−3.96689	1.237074	−1.15205
C	−3.39086	−1.0061	−0.48101
C	−4.67183	−1.43594	−0.80513
C	−5.24844	0.801792	−1.48521
H	−3.70402	2.281097	−1.28823

H	−2.67419	−1.71787	−0.08412
H	−4.93727	−2.47917	−0.66551
H	−5.96971	1.511014	−1.87856
H	−6.60407	−0.86722	−1.56965
P	−1.29352	0.803219	−0.26763
C	−1.13166	0.48865	1.533079
C	−0.66911	−0.08422	4.233436
C	−2.20018	0.210373	2.388095
C	0.170607	0.485604	2.047197
C	0.401639	0.203402	3.388649
C	−1.96779	−0.07646	3.731685
H	−3.2159	0.203351	2.006162
H	1.008772	0.692291	1.388296
H	1.417999	0.197487	3.769261
H	−2.80507	−0.29645	4.386232
H	−0.49125	−0.31458	5.278912
C	4.13336	0.172735	0.730556
S	3.590026	1.096218	-0.77868
O	3.365452	−0.06607	−1.787
O	2.334349	1.731115	−0.42004
O	4.721577	1.884498	−1.21233
F	4.306461	1.033542	1.723004
F	3.193373	−0.70699	1.076152
F	5.265289	−0.47026	0.491628
C	2.577549	−3.50378	−1.13692
H	3.442819	−3.96906	−1.60855
H	2.916741	−2.69344	−0.48551
H	2.046993	−4.24929	−0.53564
O	1.765669	−3.01811	−2.19953
C	0.657158	−2.26833	−1.82798
H	0.108834	−2.09598	−2.76009
C	−0.25077	−2.93712	−0.81192
C	−0.12886	−2.68002	0.553479
C	−1.22015	−3.83596	−1.25836
C	−0.98185	−3.29774	1.463742
H	0.619298	−1.97771	0.902292

C	−2.06626	−4.46345	−0.34894
H	−1.32174	−4.0353	−2.32156
C	−1.95223	−4.18982	1.013702
H	−0.89354	−3.06803	2.520722
H	−2.82353	−5.1548	−0.70367
H	−2.62275	−4.66706	1.720876
O	1.127829	−1.01922	−1.32327
H	2.407702	−0.47498	−1.68068
H	0.377056	−0.40179	−1.13439

Ensemble of 6a

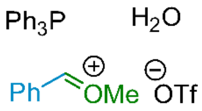

C	4.078932	−1.72211	1.798082
H	4.086876	−1.83326	2.878445
H	4.837205	−2.3473	1.327642
H	4.171409	−0.67704	1.50557
O	2.780544	−2.20415	1.366164
C	2.485318	−2.06545	0.135649
H	3.169038	−1.54331	−0.52842
C	1.224827	−2.52732	−0.35285
C	0.909248	−2.25665	−1.69427
C	0.314863	−3.2086	0.476643
C	−0.31207	−2.67062	−2.20395
H	1.613511	−1.70029	−2.30459
C	−0.89854	−3.6167	−0.04432
H	0.568475	−3.40072	1.513042
C	−1.20978	−3.34608	−1.38014
H	−0.57515	−2.45457	−3.23279
H	−1.61653	−4.13099	0.583701
H	−2.17734	−3.64374	−1.7716
C	4.286457	2.10287	−0.3965
S	2.709941	1.24745	−0.82857

O	3.114677	0.291148	−1.87469
O	2.325432	0.607572	0.445357
O	1.829784	2.332755	−1.2722
F	4.082978	3.012656	0.557227
F	5.187684	1.220605	0.052237
F	4.799992	2.716941	−1.46339
O	4.069458	−3.86909	−0.59512
H	3.596264	−4.68841	−0.41925
H	4.154859	−3.84211	−1.55326
H	−1.40883	5.32449	−0.76664
C	−2.08643	4.485486	−0.64283
C	−3.81787	2.325807	−0.34202
C	−1.5757	3.195409	−0.52336
C	−3.46275	4.697571	−0.61678
C	−4.32745	3.615081	−0.47049
C	−2.43657	2.102812	−0.36073
H	−0.50108	3.032317	−0.56787
H	−3.86121	5.702232	−0.71711
H	−5.40114	3.774332	−0.45535
H	−4.49961	1.487307	−0.23363
C	−3.02942	−0.68479	−0.61061
C	−4.96328	−2.51242	−1.50868
C	−3.41196	−0.72126	−1.95888
C	−3.62942	−1.58035	0.277465
C	−4.58668	−2.4901	−0.16946
C	−4.37504	−1.61927	−2.40383
H	−2.94757	−0.04248	−2.66988
H	−3.3424	−1.58194	1.323593
H	−5.03797	−3.18223	0.534552
H	−4.66058	−1.62915	−3.45094
H	−5.70876	−3.22086	−1.85525
H	−1.1754	−0.17341	5.505892
C	−1.27907	−0.04281	4.433317
C	−1.55348	0.292434	1.665458
C	−2.45571	0.490207	3.906463
C	−0.23679	−0.39629	3.582406

C	−0.37279	−0.22527	2.205825
C	−2.59057	0.658569	2.532691
H	−3.26767	0.775338	4.567974
H	0.686691	−0.79926	3.986469
H	0.45336	−0.46789	1.546413
H	−3.50927	1.076322	2.131004
P	−1.64874	0.448574	−0.16649

Structure 41‡

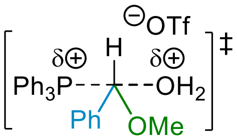

C	−0.9194	2.400912	−0.15124
C	−0.36915	5.133023	−0.35539
C	−1.95963	3.337428	−0.09346
C	0.393013	2.845198	−0.33307
C	0.667053	4.207794	−0.42868
C	−1.68393	4.695999	−0.19431
H	−2.98803	3.006113	0.012432
H	1.200353	2.126357	−0.4203
H	1.690376	4.541231	−0.56673
H	−2.49516	5.415276	−0.14911
H	−0.15711	6.194548	−0.43275
C	−2.75081	0.28929	−0.85675
C	−4.93463	−0.4666	−2.44322
C	−2.91778	0.823798	−2.1418
C	−3.69603	−0.62508	−0.3762
C	−4.77923	−1.00124	−1.16669
C	−4.00524	0.45292	−2.92538
H	−2.19339	1.531551	−2.53364
H	−3.58786	−1.04823	0.61756
H	−5.50294	−1.7118	−0.78124
H	−4.1222	0.877055	−3.91712

H	−5.77807	−0.76196	−3.05831
P	−1.18345	0.59919	0.028847
C	−1.59564	0.334157	1.793182
C	−2.13171	−0.14907	4.495096
C	−2.70061	0.930884	2.414189
C	−0.76606	−0.50579	2.541068
C	−1.03344	−0.74727	3.88682
C	−2.96531	0.691454	3.757176
H	−3.36308	1.576548	1.846456
H	0.091534	−0.97748	2.074318
H	−0.38344	−1.40494	4.454494
H	−3.82369	1.158214	4.229025
H	−2.34244	−0.33694	5.542932
C	3.477345	0.752778	0.893695
S	3.896388	0.239378	−0.82898
O	4.059235	−1.2269	−0.70743
O	2.701724	0.635878	−1.59716
O	5.121727	0.974624	−1.14047
F	3.385779	2.081332	0.97492
F	2.297224	0.232605	1.257449
F	4.402419	0.337638	1.755807
C	−1.36124	−2.17232	−2.96855
H	−1.36457	−2.0171	−4.04418
H	−1.05774	−3.19034	−2.72631
H	−2.34129	−1.93216	−2.55661
O	−0.38227	−1.23359	−2.47033
C	0.236307	−1.35539	−1.35221
H	1.080209	−0.67448	−1.27593
C	0.078593	−2.39955	−0.34852
C	−1.13546	−3.03351	−0.04022
C	1.228608	−2.69642	0.401097
C	−1.19183	−3.94453	1.004464
H	−2.04567	−2.78674	−0.57078
C	1.166438	−3.6269	1.427985
H	2.16539	−2.19895	0.172265
C	−0.04387	−4.24602	1.734714

H	−2.13593	−4.41584	1.25263
H	2.059001	−3.86014	1.997405
H	−0.09497	−4.96066	2.549172
O	2.365469	−2.46055	−2.64445
H	2.327906	−1.77316	−3.31629
H	3.038304	−2.12948	−2.02611

Ensemble of 12F

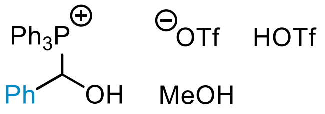

O	−0.93053	−1.9341	0.242127
C	−0.82699	−0.76452	−0.53255
H	0.203872	−0.39439	−0.56443
C	−1.32486	−0.94814	−1.94224
C	−2.49348	−1.66769	−2.20156
C	−0.61633	−0.36983	−2.99741
C	−2.95394	−1.79606	−3.50762
H	−3.03767	−2.13036	−1.38478
C	−1.08188	−0.49782	−4.30242
H	0.289977	0.190687	−2.78893
C	−2.25219	−1.20769	−4.55836
H	−3.86214	−2.35562	−3.70546
H	−0.52904	−0.04423	−5.11825
H	−2.61548	−1.30733	−5.57597
C	3.059926	3.049673	0.3947
S	3.03268	1.395277	−0.42181
O	1.65256	1.229443	−0.88556
O	4.067623	1.440884	−1.44763
O	3.368549	0.508972	0.745518
F	4.276414	3.31212	0.86272
F	2.726781	3.986737	−0.48947
F	2.194191	3.084474	1.406747
C	−1.48803	2.159788	−0.49636

C	−0.96506	4.541904	−1.83247
C	−0.77712	3.177659	0.144706
C	−1.94239	2.337903	−1.81069
C	−1.67953	3.530794	−2.47177
C	−0.51614	4.365835	−0.52723
H	−0.41661	3.042334	1.157225
H	−2.49521	1.555992	−2.31971
H	−2.03101	3.667634	−3.48826
H	0.044844	5.148888	−0.03043
H	−0.75812	5.469669	−2.35518
C	−3.5154	0.211496	0.447626
C	−6.21558	−0.4498	0.592045
C	−4.47462	1.069916	−0.09736
C	−3.91302	−0.97193	1.086097
C	−5.26113	−1.29843	1.149696
C	−5.82271	0.733994	−0.02395
H	−4.18194	1.999464	−0.57091
H	−3.1872	−1.64106	1.534454
H	−5.56422	−2.21674	1.640246
H	−6.56361	1.402436	−0.44799
H	−7.26755	−0.70913	0.645225
P	−1.75634	0.598331	0.358732
C	−1.08689	0.669725	2.027516
C	−0.03585	0.748906	4.598448
C	−1.9312	0.912901	3.11611
C	0.284752	0.478353	2.225391
C	0.804761	0.516912	3.512589
C	−1.3998	0.952133	4.400267
H	−2.99531	1.060429	2.966826
H	0.955929	0.301632	1.393501
H	1.867818	0.363638	3.659808
H	−2.05313	1.136285	5.245672
H	0.372668	0.773068	5.603058
O	−2.11864	−3.53658	2.279083
H	−1.85931	−3.19929	1.411845
C	−1.24007	−2.95528	3.227542

H	−1.47265	−3.39371	4.200421
H	−0.18689	−3.16254	2.998998
H	−1.36857	−1.86744	3.302366
H	−0.05198	−2.11397	0.62045
C	2.337425	−2.89322	−1.32838
S	2.799618	−2.81606	0.463342
O	1.649434	−2.20854	1.131544
O	3.238078	−4.13634	0.84886
O	3.994435	−1.84151	0.443801
F	3.28886	−3.49667	−2.01814
F	1.199025	−3.56291	−1.44464
F	2.16659	−1.65767	−1.78894
H	3.715915	−0.81624	0.554163

Ensemble of 43

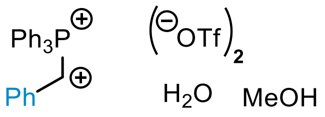

C	−1.46116	1.167713	0.629888
H	−2.04289	0.446572	1.21052
C	−2.10391	2.293182	0.198802
C	−1.52471	3.291987	−0.67199
C	−3.48592	2.430677	0.600117
C	−2.29559	4.327667	−1.11736
H	−0.49207	3.21262	−0.98478
C	−4.23553	3.484014	0.141154
H	−3.93208	1.693897	1.267933
C	−3.64349	4.423613	−0.71098
H	−1.88026	5.076042	−1.78003
H	−5.2766	3.574236	0.421374
H	−4.23882	5.255154	−1.07402
C	6.502554	−0.82356	−0.36821
S	4.72444	−0.347	−0.23598
O	4.364553	0.010913	-1.61554

O	4.085502	−1.57452	0.269114
O	4.745273	0.772682	0.718655
F	6.986374	−1.19138	0.822183
F	6.657324	−1.84692	−1.21397
F	7.235719	0.200355	−0.81654
C	0.204852	−0.41042	−1.12737
C	0.195204	−2.05127	−3.35444
C	1.187713	−1.39884	−1.25507
C	−0.78137	−0.23798	−2.10414
C	−0.78254	−1.0692	−3.2164
C	1.179673	−2.2108	−2.38195
H	1.961895	−1.52796	−0.50323
H	−1.56061	0.506698	−1.99574
H	−1.55273	−0.95286	−3.96996
H	1.942438	−2.97286	−2.49442
H	0.187711	−2.69867	−4.22477
C	1.34418	2.03612	0.13393
C	2.95018	4.266486	−0.19488
C	2.077555	2.186578	−1.0445
C	1.422062	2.989209	1.160675
C	2.228031	4.103703	0.988646
C	2.879827	3.312155	−1.20321
H	2.03976	1.432587	−1.82154
H	0.861711	2.864837	2.082193
H	2.29335	4.846609	1.775191
H	3.455069	3.433412	−2.11359
H	3.57639	5.142517	−0.32482
P	0.261348	0.623598	0.336804
C	0.721222	−0.31308	1.798209
C	1.494856	−1.82368	3.988893
C	1.994581	−0.12955	2.346414
C	−0.16321	−1.26355	2.329334
C	0.23131	−2.01175	3.429135
C	2.373451	−0.89235	3.445889
H	2.701185	0.569309	1.910951
H	−1.14576	−1.43147	1.899734

H	−0.44945	−2.7439	3.848421
H	3.362253	−0.75886	3.869629
H	1.795658	−2.41304	4.848539
C	−2.89062	−2.98329	−0.50213
S	−3.94304	−1.59662	0.118059
O	−3.73594	−0.50595	−0.85909
O	−5.2935	−2.15753	0.143306
O	−3.37305	−1.29072	1.451859
F	−3.17943	−4.0992	0.164277
F	−3.11511	−3.18609	−1.79805
F	−1.59584	−2.71518	−0.32945
O	−5.42201	0.635282	2.354732
H	−4.85311	−0.14448	2.298469
H	−5.83069	0.689128	1.470981
C	−7.28674	0.333868	−0.85934
H	−8.15377	0.756567	−0.34808
H	−7.25514	−0.74516	−0.67754
H	−7.39565	0.514047	−1.93538
O	−6.1317	0.978901	−0.34171
H	−5.34955	0.546099	−0.72282

Ensemble of 44

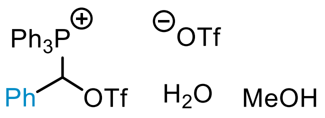

C	1.173185	0.347064	−0.33289
H	0.14175	0.352436	−0.70811
C	2.132938	0.515099	−1.47414
C	3.503572	0.693825	−1.26318
C	1.622871	0.476426	−2.77371
C	4.358306	0.812016	−2.35265
H	3.901579	0.753733	−0.25599
C	2.484848	0.587678	−3.8595
H	0.553097	0.361088	−2.91849

C	3.851676	0.749936	−3.64983
H	5.421113	0.952491	−2.18881
H	2.087574	0.553255	−4.86808
H	4.523562	0.837262	−4.49716
C	−4.92569	−1.01856	−0.09689
S	−4.6563	0.789683	−0.34413
O	−3.95369	0.862273	−1.6427
O	−6.00176	1.362952	−0.34588
O	−3.8132	1.147409	0.813498
F	−5.52731	−1.24241	1.072819
F	−5.6857	−1.52031	−1.06911
F	−3.75919	−1.67329	−0.09565
C	0.984709	−2.57163	−0.58977
C	0.399678	−4.58701	−2.41232
C	−0.10314	−3.42105	−0.36368
C	1.787146	−2.73646	−1.72875
C	1.487682	−3.74462	−2.63569
C	−0.39065	−4.4277	−1.27844
H	−0.72784	−3.29749	0.513435
H	2.640096	−2.09156	−1.90936
H	2.106195	−3.87161	−3.51681
H	−1.23599	−5.0834	−1.10384
H	0.170311	−5.37138	−3.12543
C	2.989611	−1.41028	1.274666
C	5.556377	−1.57361	2.327159
C	3.81832	−2.47863	0.92218
C	3.440505	−0.42906	2.171358
C	4.724848	−0.51471	2.690043
C	5.102775	−2.55432	1.451392
H	3.471346	−3.25002	0.244579
H	2.797539	0.395034	2.45996
H	5.07592	0.244545	3.379533
H	5.745987	−3.38252	1.176645
H	6.559634	−1.63535	2.73464
P	1.333238	−1.2596	0.58939
C	0.117232	−1.24344	1.910331

C	−1.78005	−1.27221	3.933824
C	0.470697	−1.69778	3.18571
C	−1.18844	−0.81117	1.642167
C	−2.13215	−0.82668	2.66184
C	−0.48448	−1.70983	4.195258
H	1.479082	−2.03815	3.393076
H	−1.48167	−0.48788	0.646945
H	−3.13671	−0.48133	2.450212
H	−0.2135	−2.05976	5.184991
H	−2.52124	−1.2799	4.725991
O	−1.44902	−0.25917	−1.76174
H	−2.27537	0.251515	−1.67781
C	−1.7059	−1.34866	−2.62792
H	−0.75228	−1.83705	−2.84284
H	−2.37726	−2.0829	−2.16999
H	−2.14306	−1.01326	−3.57592
C	1.879224	3.856065	−0.0731
S	0.536237	2.741748	0.579091
O	1.34587	1.356045	0.697957
O	0.245815	3.166404	1.923825
O	−0.47979	2.627878	−0.44545
F	1.484724	5.104304	0.107124
F	2.995494	3.628603	0.596682
F	2.063649	3.619778	−1.35882
O	−3.07467	3.871075	0.374869
H	−3.33735	2.996985	0.700781
H	−2.30572	3.663163	−0.16568

Structure 45‡

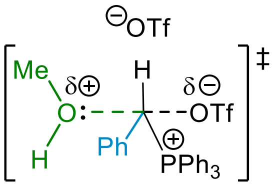

C	−0.45406	0.097835	−0.68198
H	0.437145	−0.24359	−0.17244
C	−0.52346	−0.07919	−2.09795

C	−1.67515	0.184144	−2.86656
C	0.638387	−0.57113	−2.72925
C	−1.65258	−0.01707	−4.23467
H	−2.59496	0.511314	−2.39748
C	0.650713	−0.76671	−4.09973
H	1.524217	−0.77227	−2.13359
C	−0.49037	−0.48693	−4.85053
H	−2.53957	0.182731	−4.82404
H	1.54638	−1.13655	−4.58491
H	−0.47911	−0.64172	−5.92411
C	5.10677	1.431125	−0.16232
S	3.852688	0.117297	0.15601
O	2.794797	0.853409	0.894716
O	3.452864	−0.32433	−1.18333
O	4.563511	−0.8705	0.976853
F	6.134859	0.935039	−0.84837
F	4.559584	2.421323	−0.87054
F	5.559791	1.931041	0.985774
C	−1.72523	2.672652	0.187359
C	−1.79223	5.43998	−0.08961
C	−1.66417	3.486329	1.319924
C	−1.81717	3.246062	−1.08656
C	−1.85112	4.626407	−1.22037
C	−1.6995	4.870519	1.174948
H	−1.58263	3.05392	2.310448
H	−1.84975	2.629791	−1.9781
H	−1.91856	5.066025	−2.20899
H	−1.64964	5.499839	2.056298
H	−1.81618	6.518783	−0.19817
C	−3.42676	0.298297	0.084077
C	−6.05436	−0.53091	−0.33213
C	−4.33865	1.145984	−0.55826
C	−3.84753	−0.95724	0.546354
C	−5.15809	−1.36289	0.334777
C	−5.64499	0.722899	−0.77405
H	−4.04675	2.136476	−0.88591

H	−3.15796	−1.60929	1.064564
H	−5.47859	−2.33386	0.695116
H	−6.34224	1.382391	−1.27791
H	−7.07586	−0.85639	−0.49684
P	−1.74039	0.878521	0.413831
C	−1.31306	0.473379	2.113391
C	−0.74658	−0.0724	4.779475
C	−2.34303	0.490034	3.064416
C	0.001752	0.201223	2.502993
C	0.275574	−0.08632	3.835485
C	−2.05218	0.225193	4.396403
H	−3.36527	0.705864	2.773082
H	0.829092	0.192439	1.802735
H	1.291621	−0.35443	4.103621
H	−2.85007	0.23902	5.130219
H	−0.52736	−0.29831	5.817605
O	0.857395	1.933451	−0.56628
H	1.615221	1.616827	−0.02225
C	1.368995	2.443843	−1.78784
H	2.079941	1.749052	−2.24521
H	0.530382	2.600822	−2.46856
H	1.862321	3.404375	−1.61079
C	−0.10707	−4.09917	−0.73228
S	−0.26527	−2.79057	0.559266
O	−1.2099	−1.82449	−0.09585
O	−0.82845	−3.45473	1.727443
O	1.091688	−2.24082	0.691909
F	0.718109	−5.05094	−0.30561
F	−1.29649	−4.63731	−0.98594
F	0.378817	−3.57604	−1.85506
O	2.802725	−1.87615	3.036817
H	3.482677	−1.48892	2.466679
H	2.104408	−2.09032	2.403416

16. NMR Spectra

(i) Starting materials

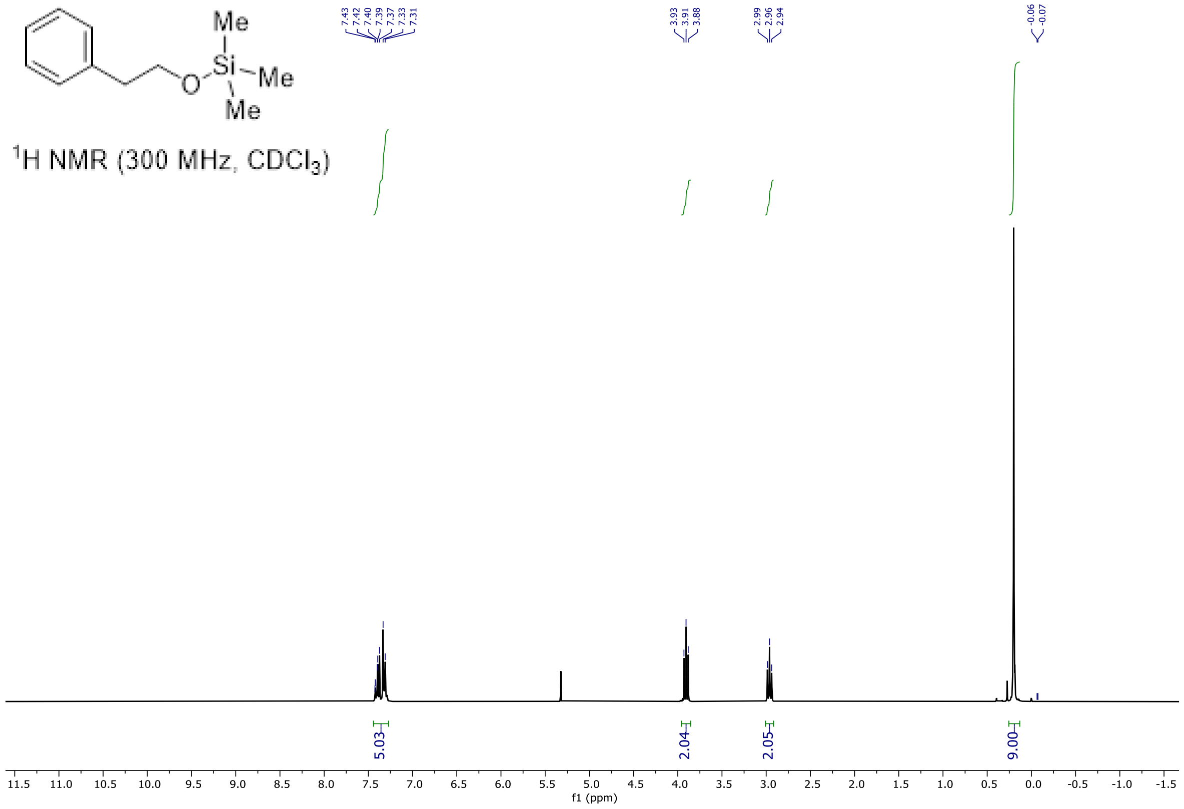

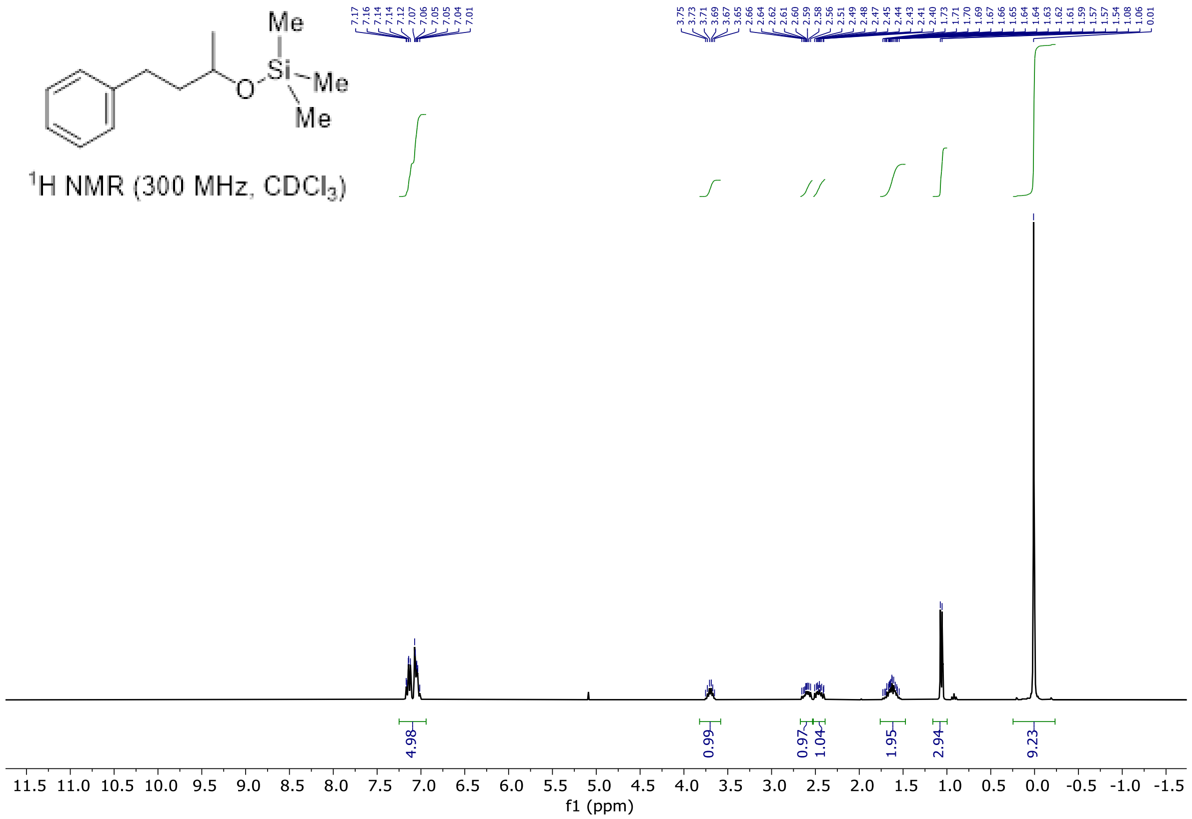

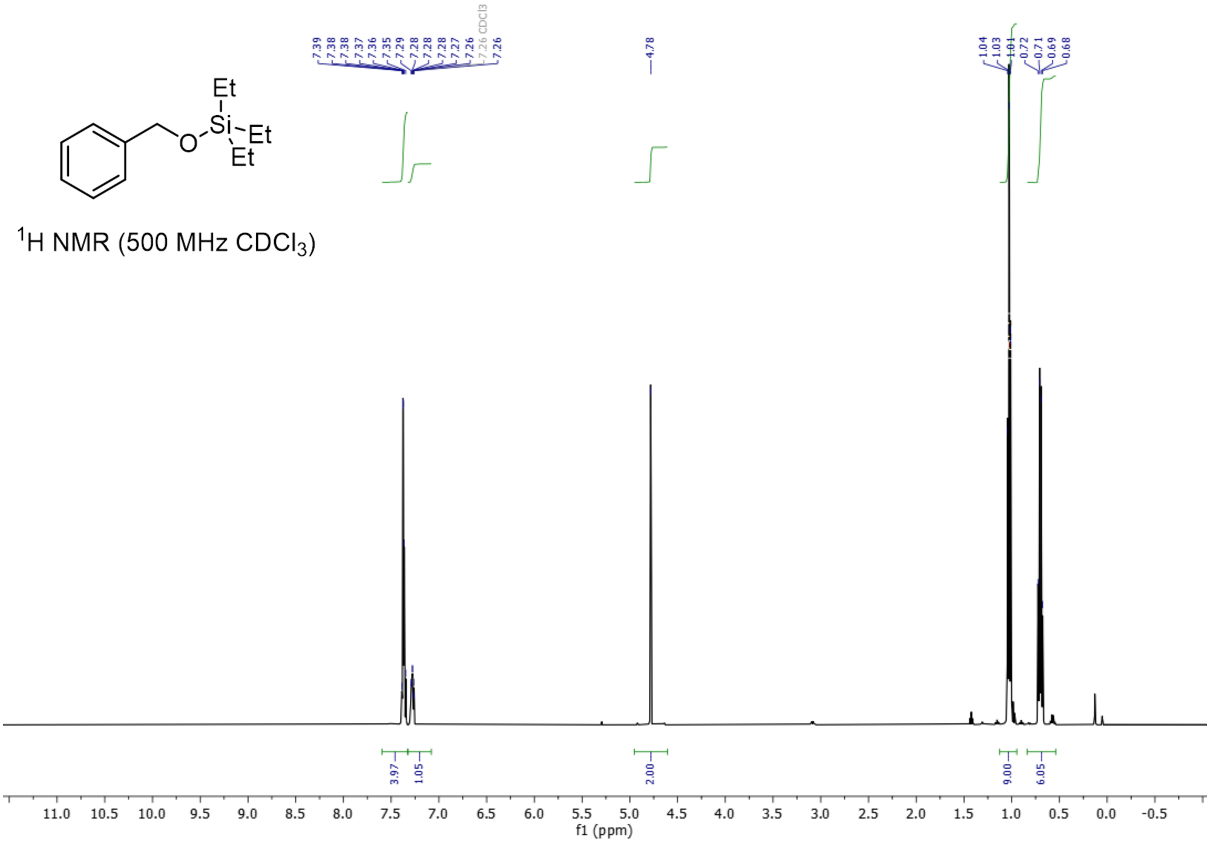
Et
Si
Et
Et
O
^{1}H NMR (500 MHz CDCl$_3$)
f1 (ppm)

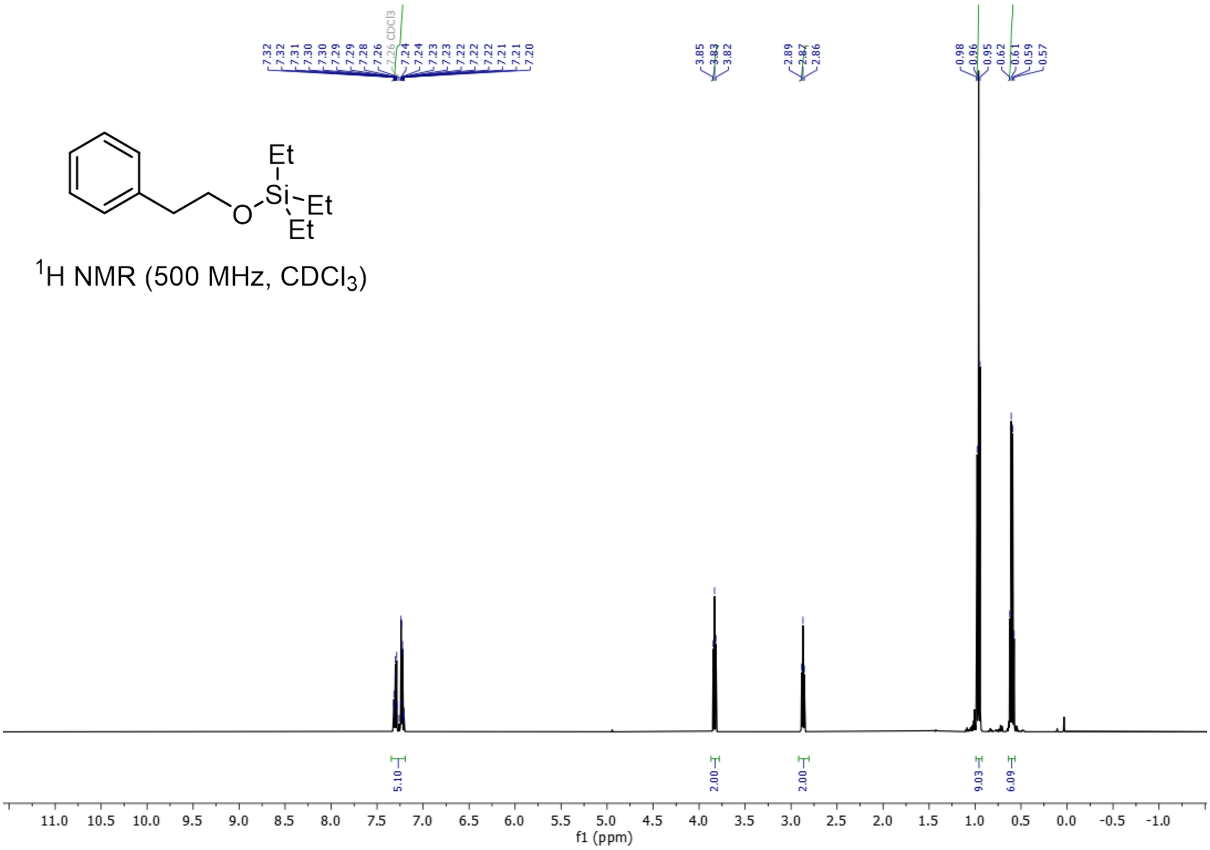
Et
Si
Et
Et
O
^{1}H NMR (500 MHz, CDCl$_3$)
f1 (ppm)

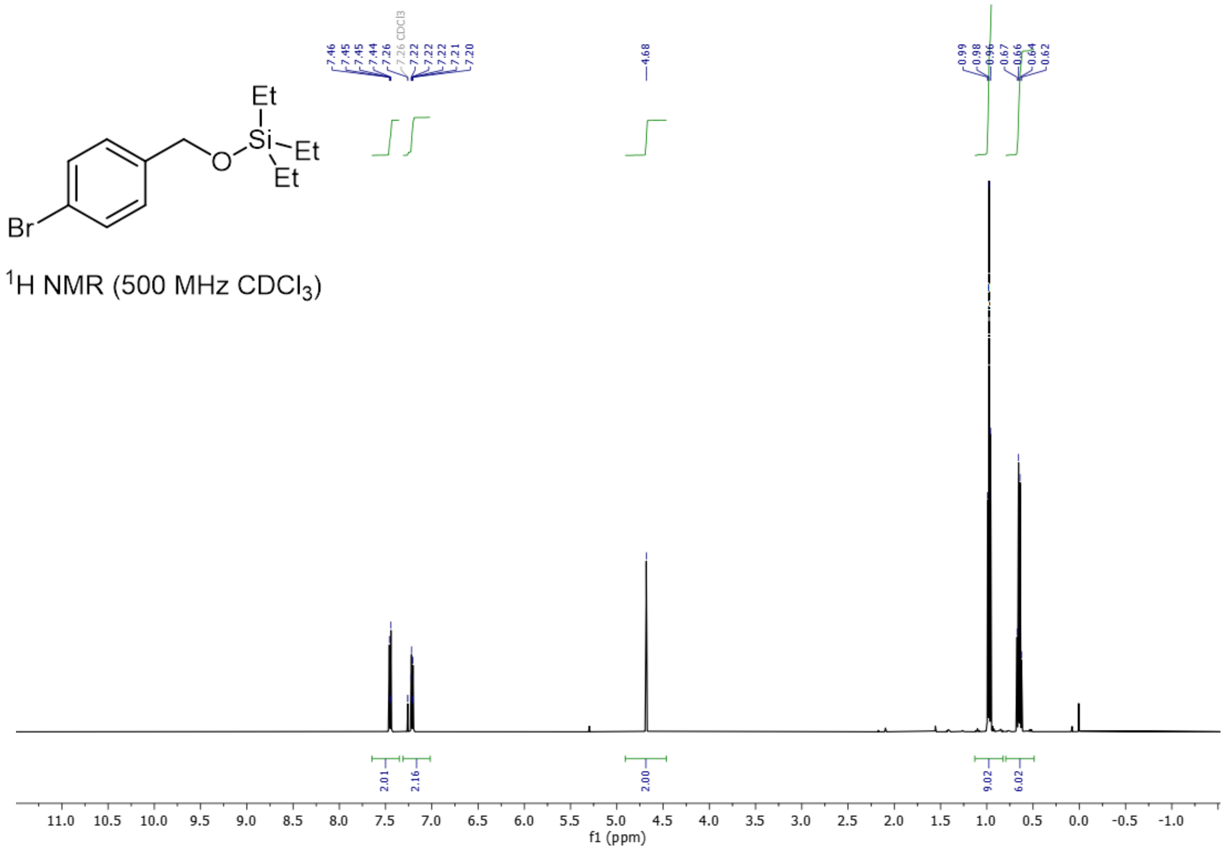
Et
Si
Et
Et
O
Br
^{1}H NMR (500 MHz CDCl$_3$)
f1 (ppm)

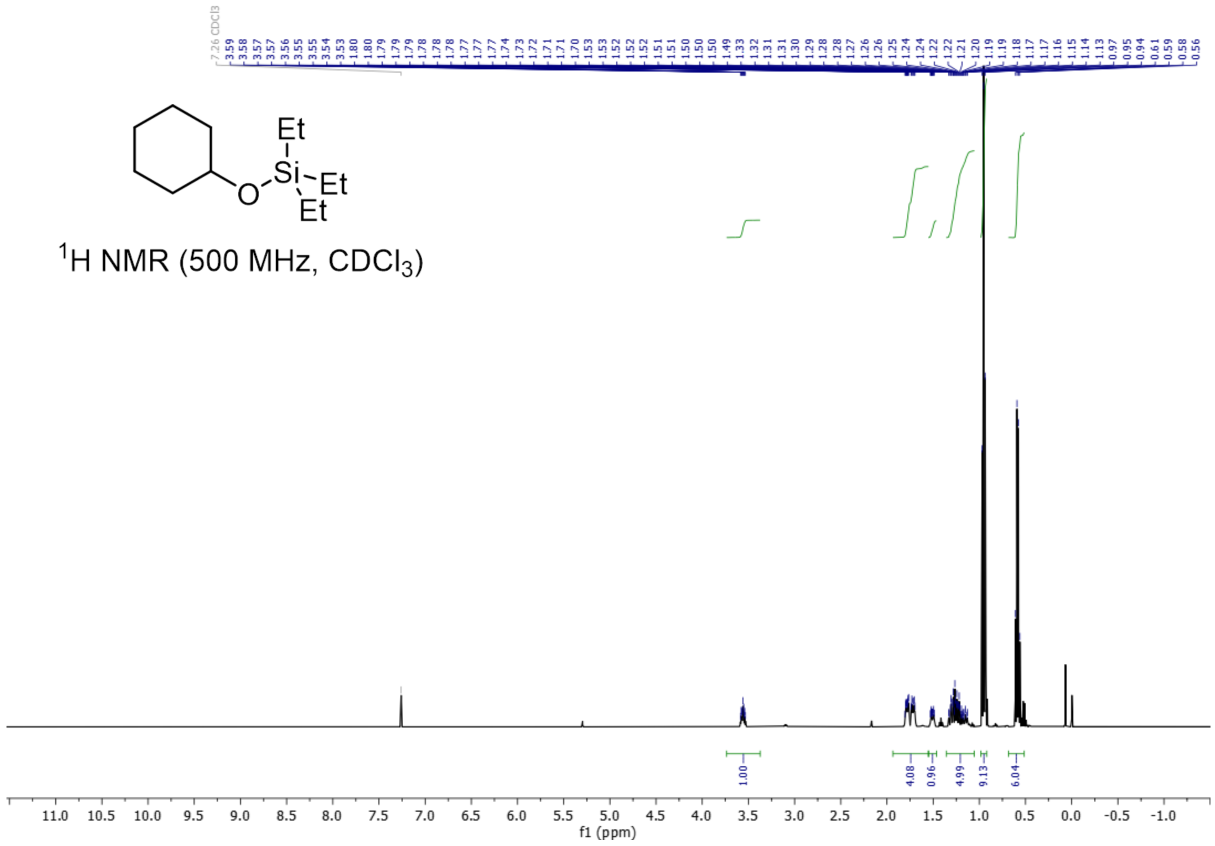
Et
Si
Et
Et
O
^{1}H NMR (500 MHz, CDCl$_3$)
f1 (ppm)

[Ph_3PH]OTf

^1H NMR (400 MHz, $CDCl_3$)

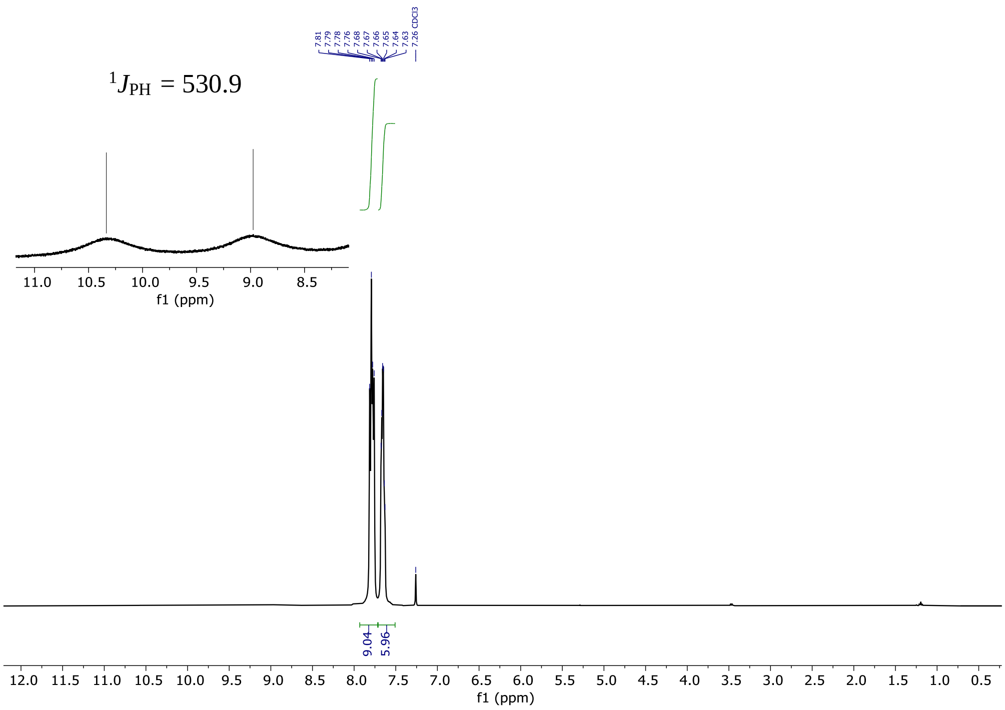

^{31}P {^1H} NMR (162 MHz, $CDCl_3$)

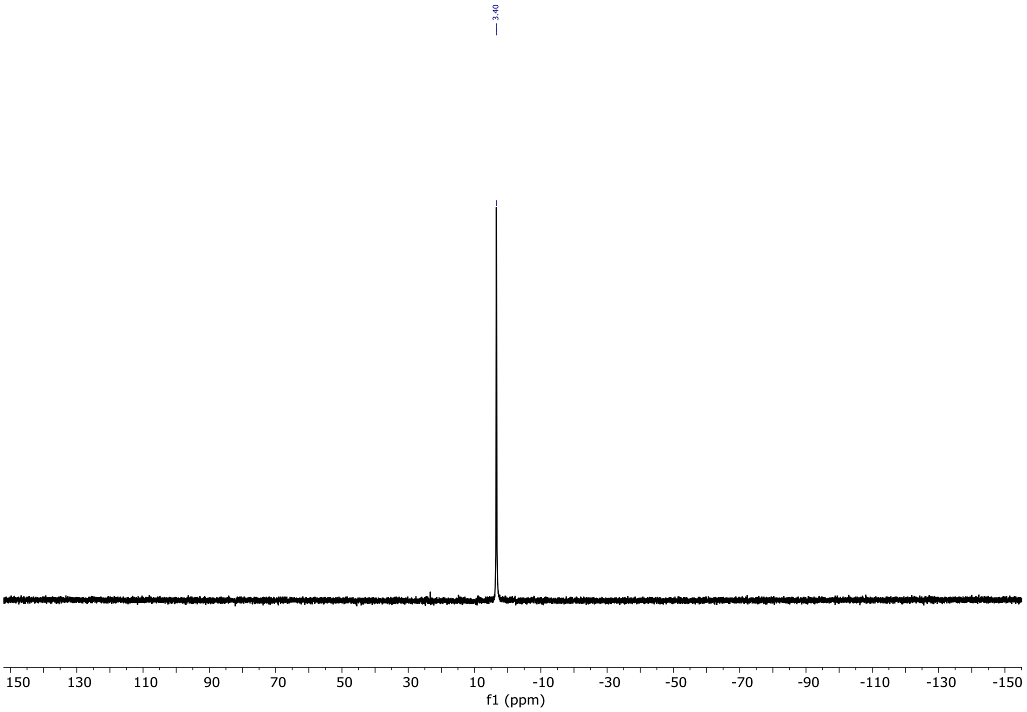

^{19}F NMR (376 MHz, $CDCl_3$)

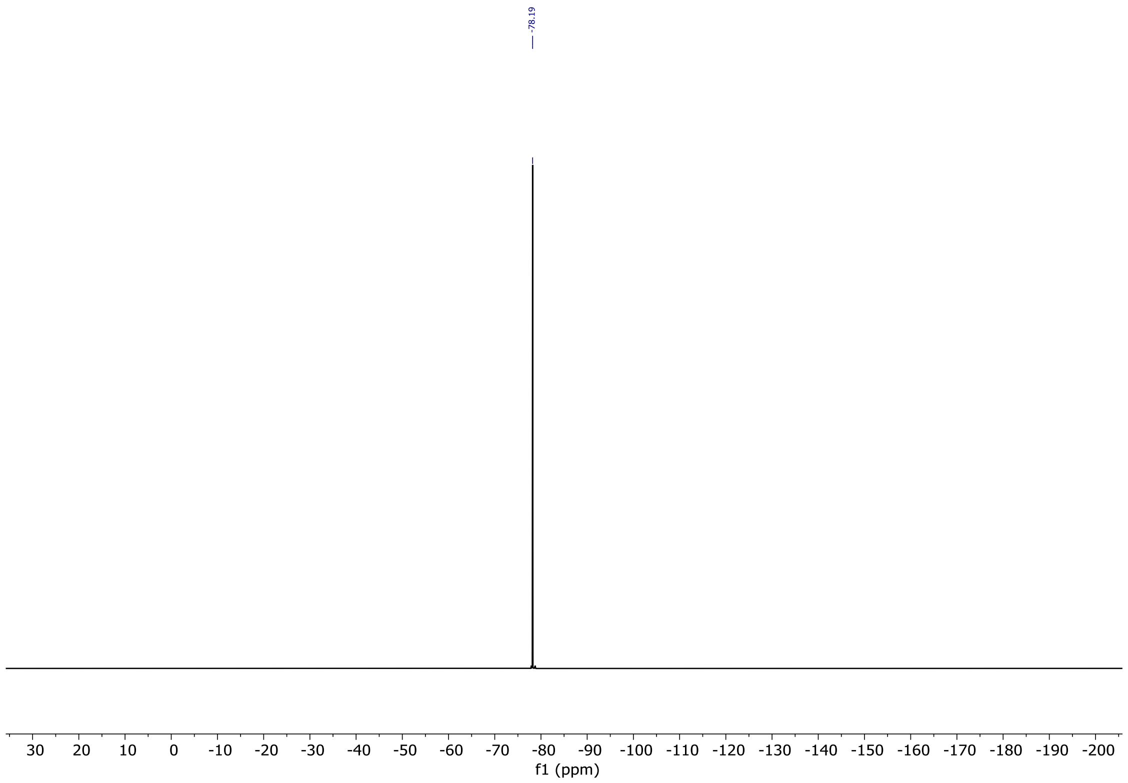

(ii) NMR spectra of (α-alkoxyalkyl)phosphonium triflates (**10**)

NMR spectra of compound **10A**

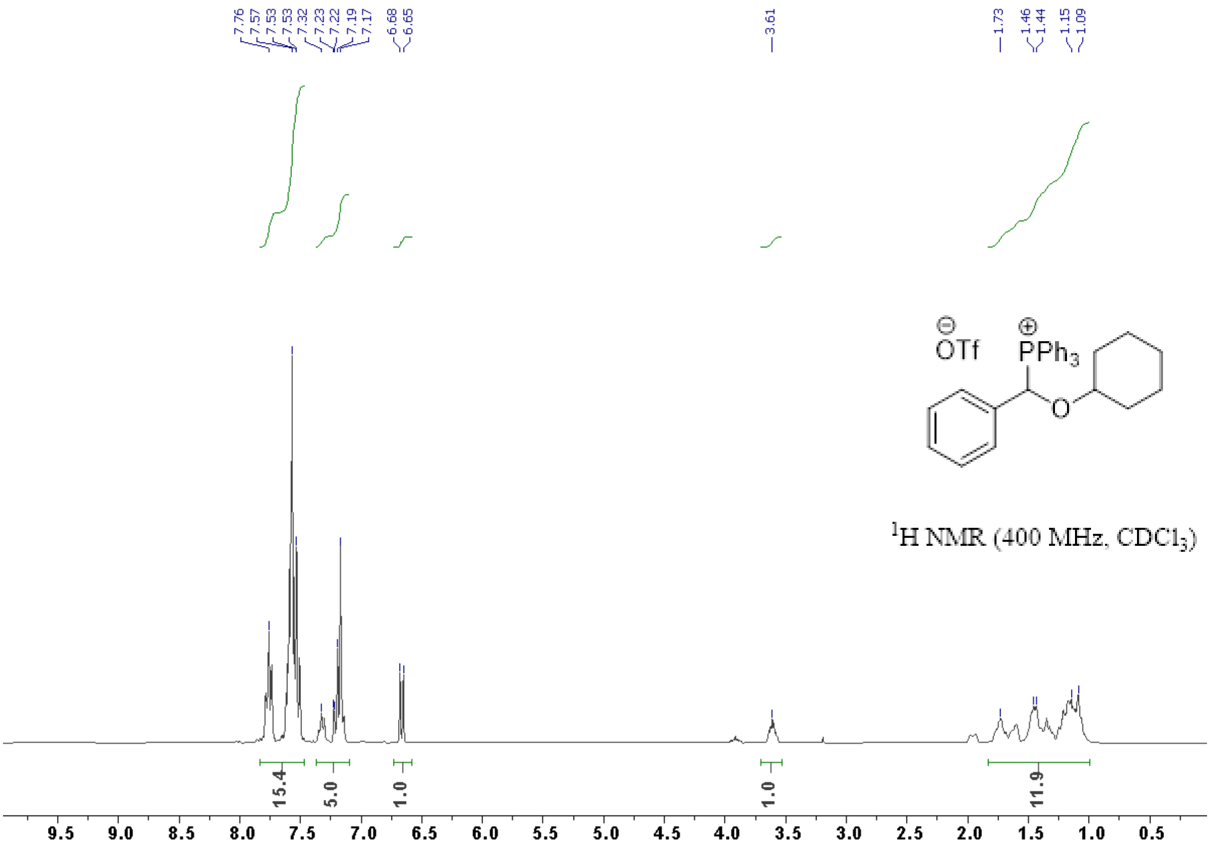

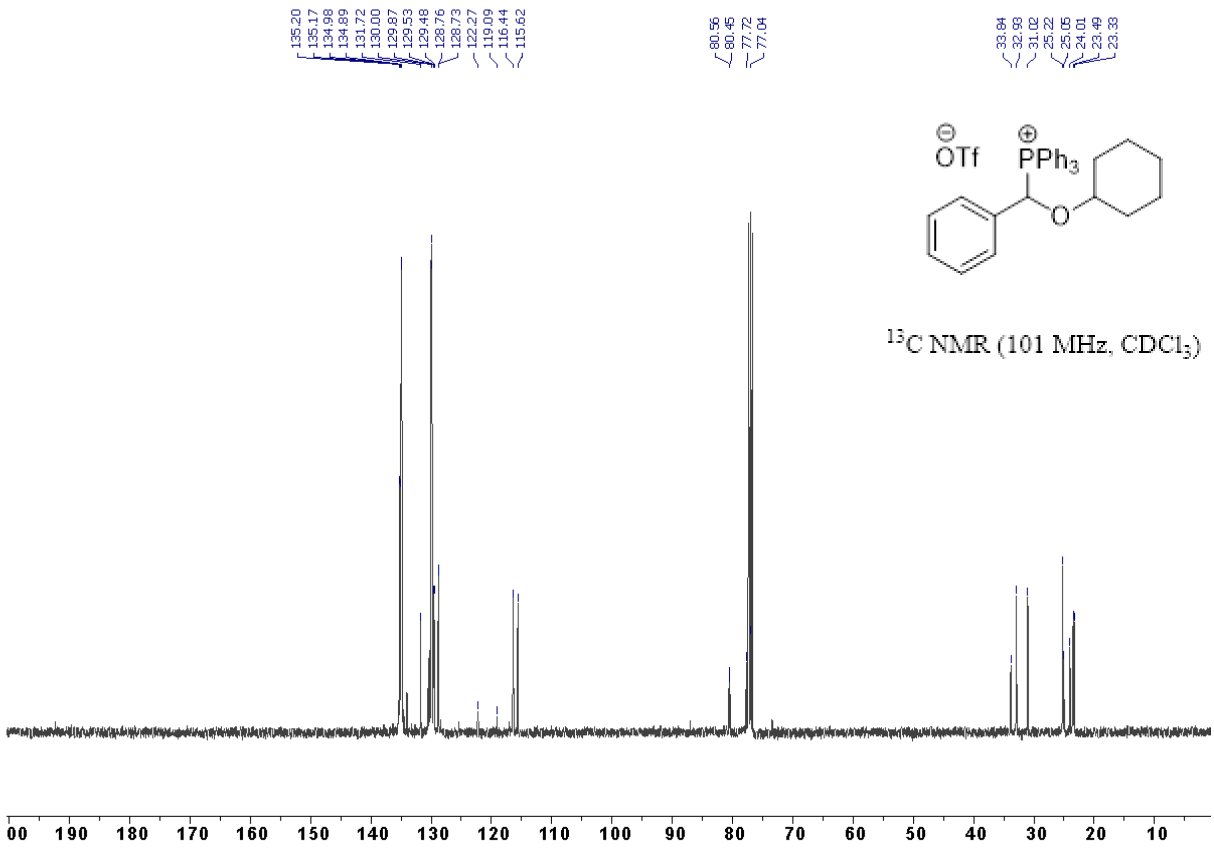
135.20
135.17
134.98
134.89
131.72
130.00
129.87
129.53
129.48
128.76
128.73
122.27
119.09
116.44
115.62
80.56
80.45
77.72
77.04
33.84
32.93
31.02
25.22
25.05
24.01
23.49
23.33
OTf
PPh3
O
13C NMR (101 MHz, CDCl3)

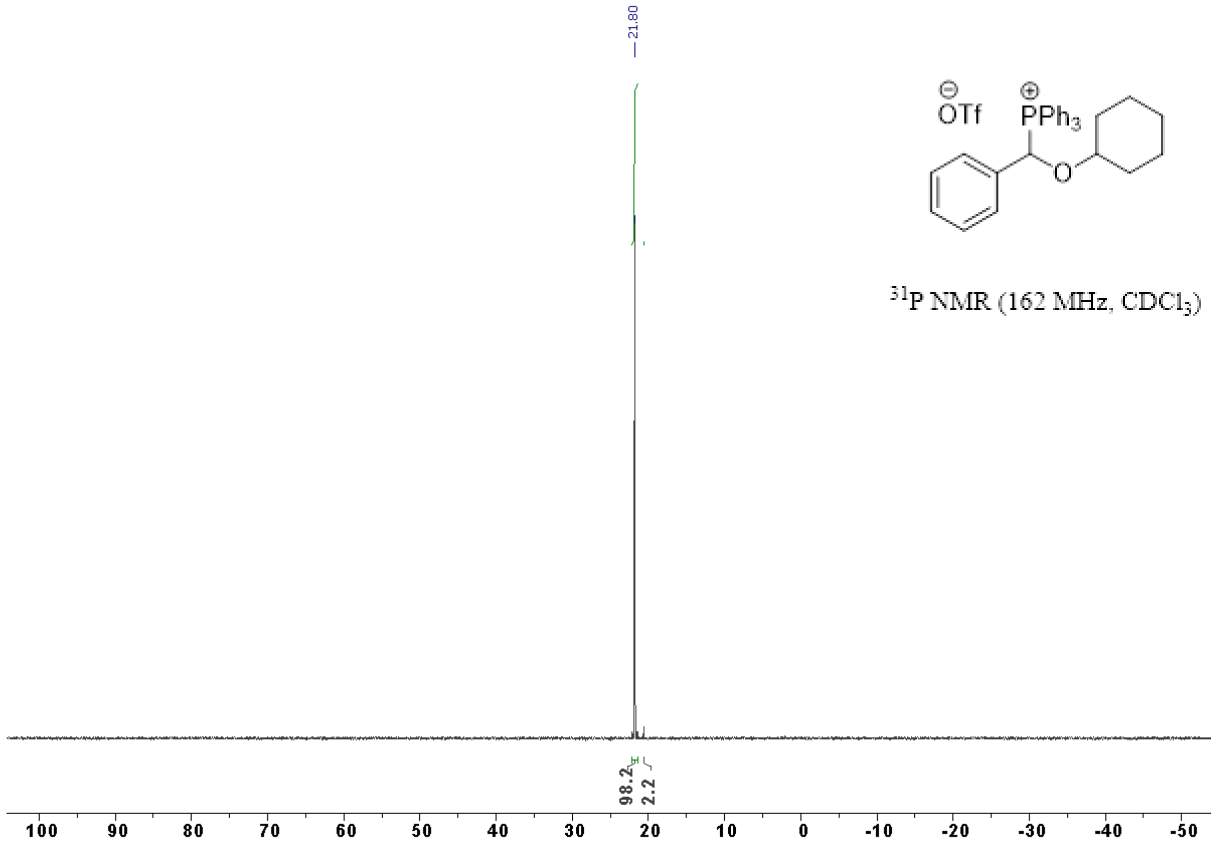
21.80
OTf
PPh3
O
31P NMR (162 MHz, CDCl3)
98.2
2.2

NMR spectra of compound **10B**

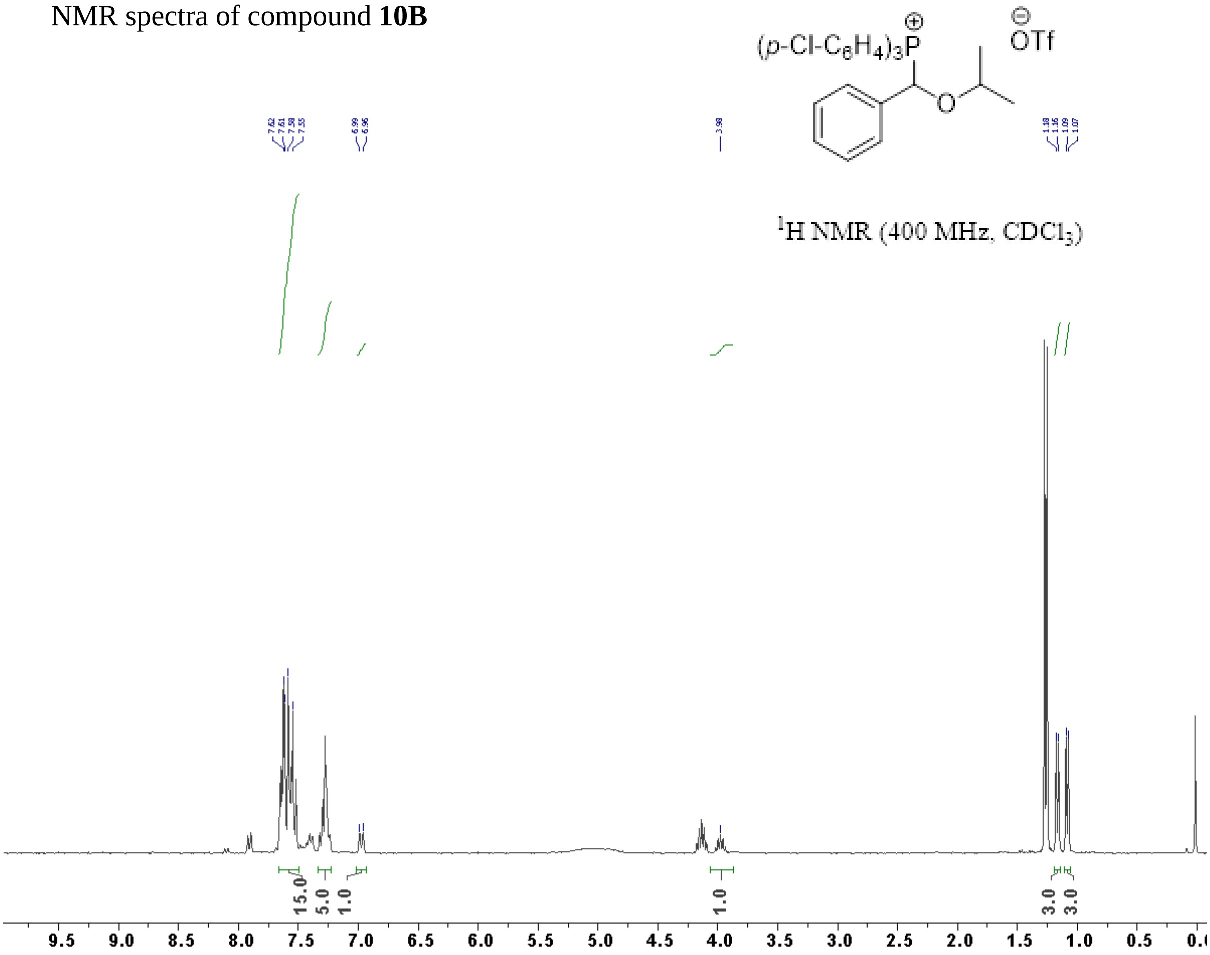

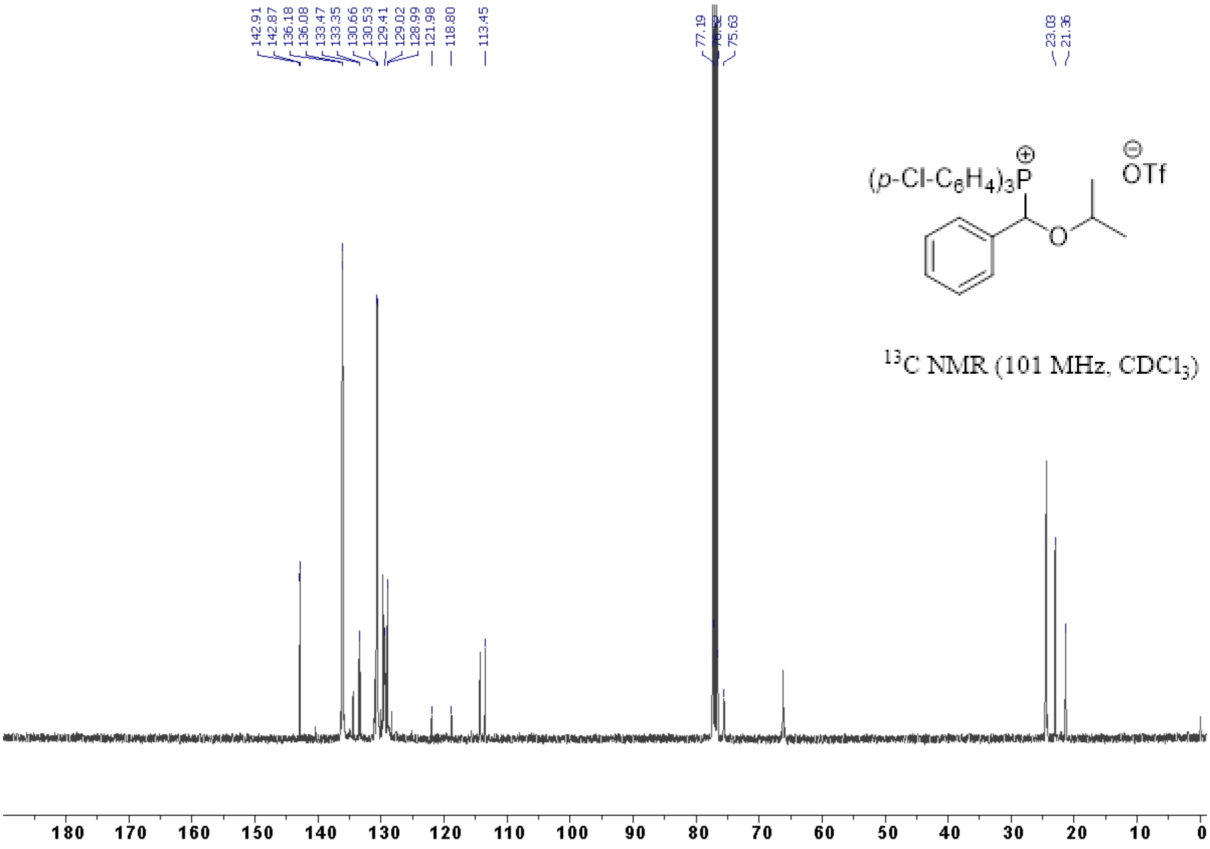

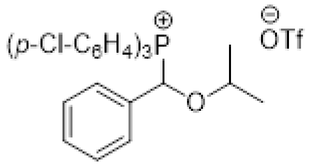

^{31}P NMR (162 MHz, $CDCl_3$)

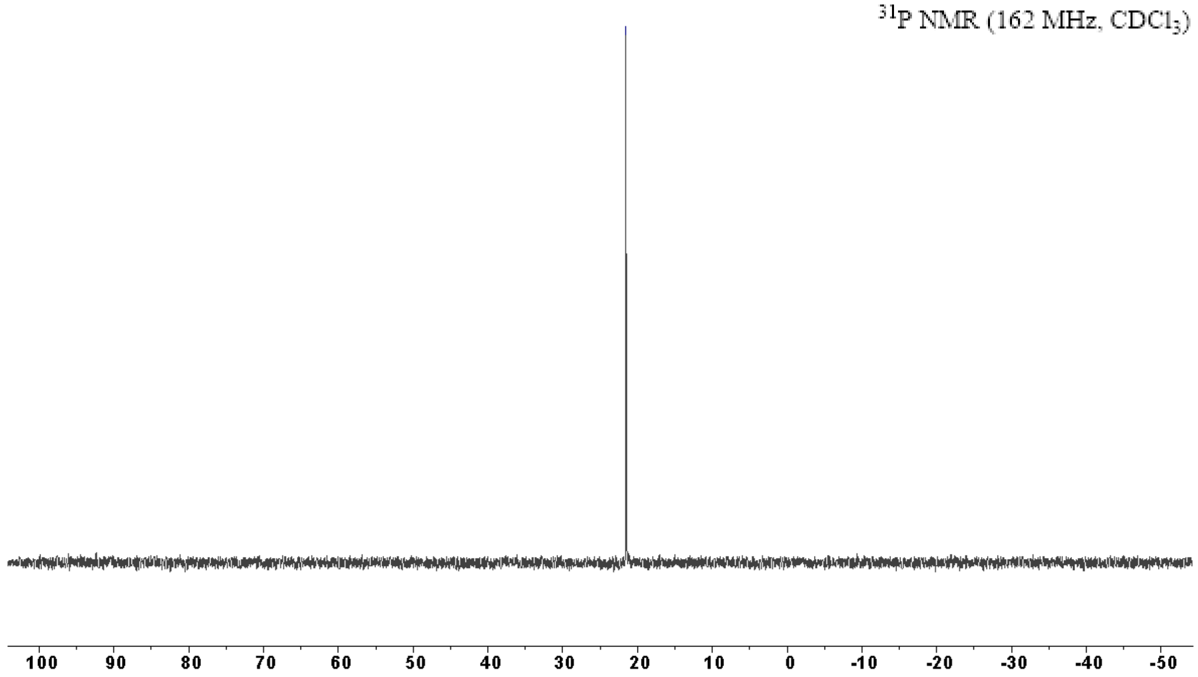

NMR spectra of compound **10C**

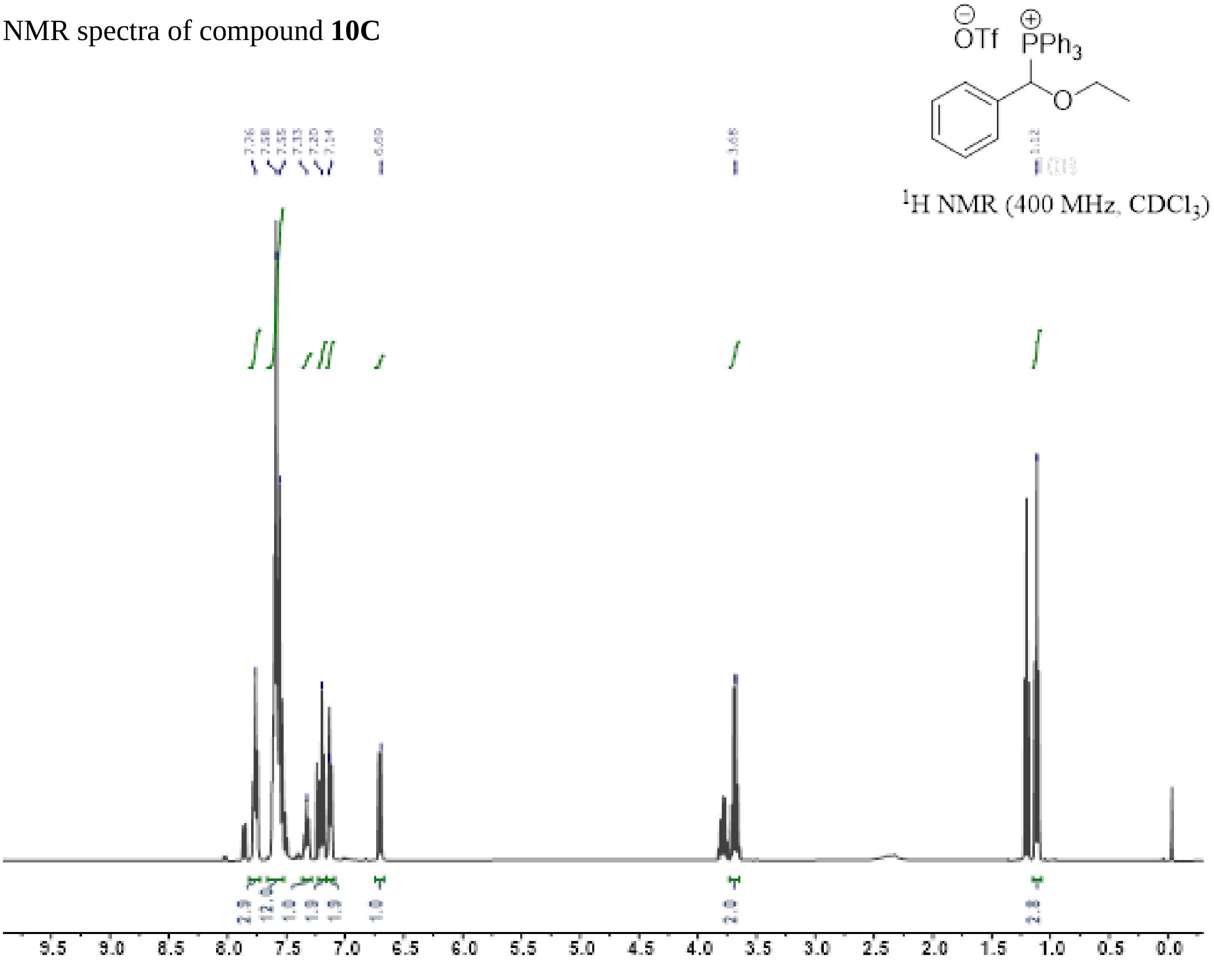

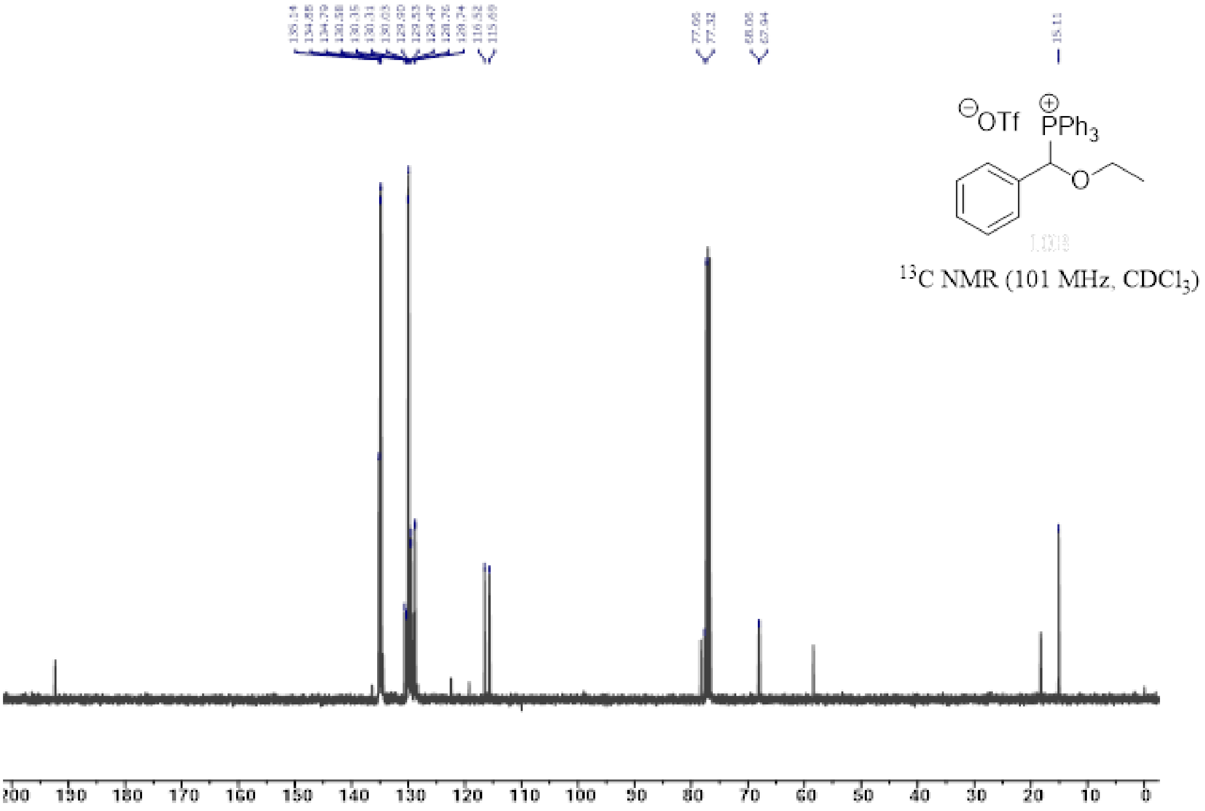

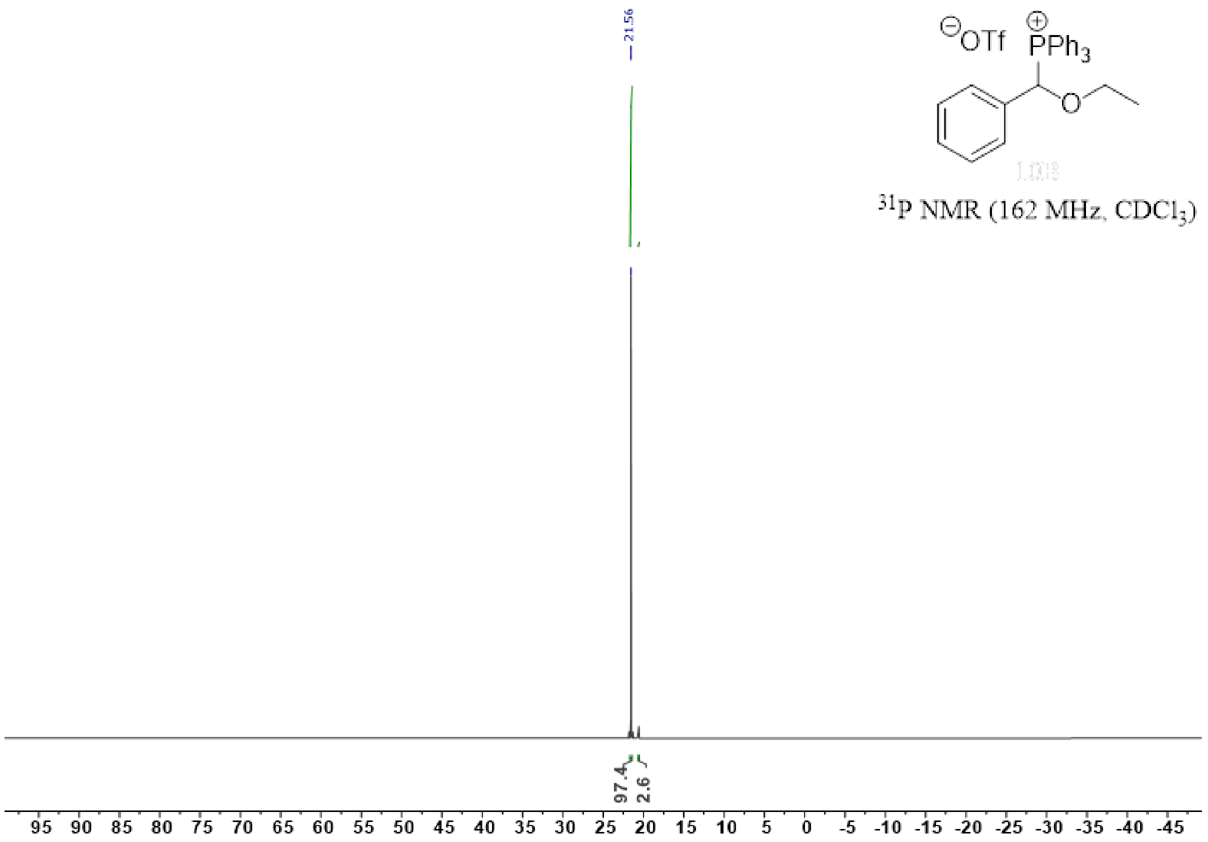

21.56
OTf
PPh3
O
31P NMR (162 MHz, CDCl3)
97.4
2.6
95 90 85 80 75 70 65 60 55 50 45 40 35 30 25 20 15 10 5 0 -5 -10 -15 -20 -25 -30 -35 -40 -45

NMR spectra of compound **10D**

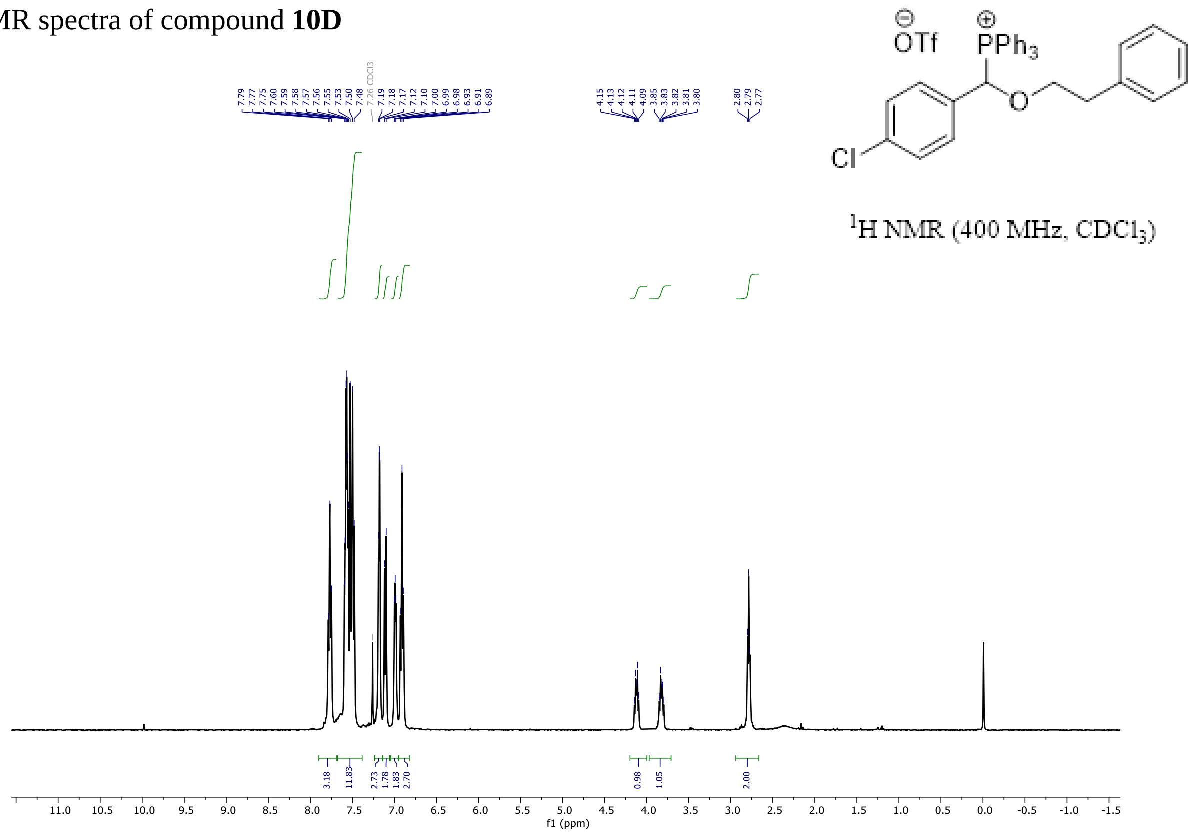

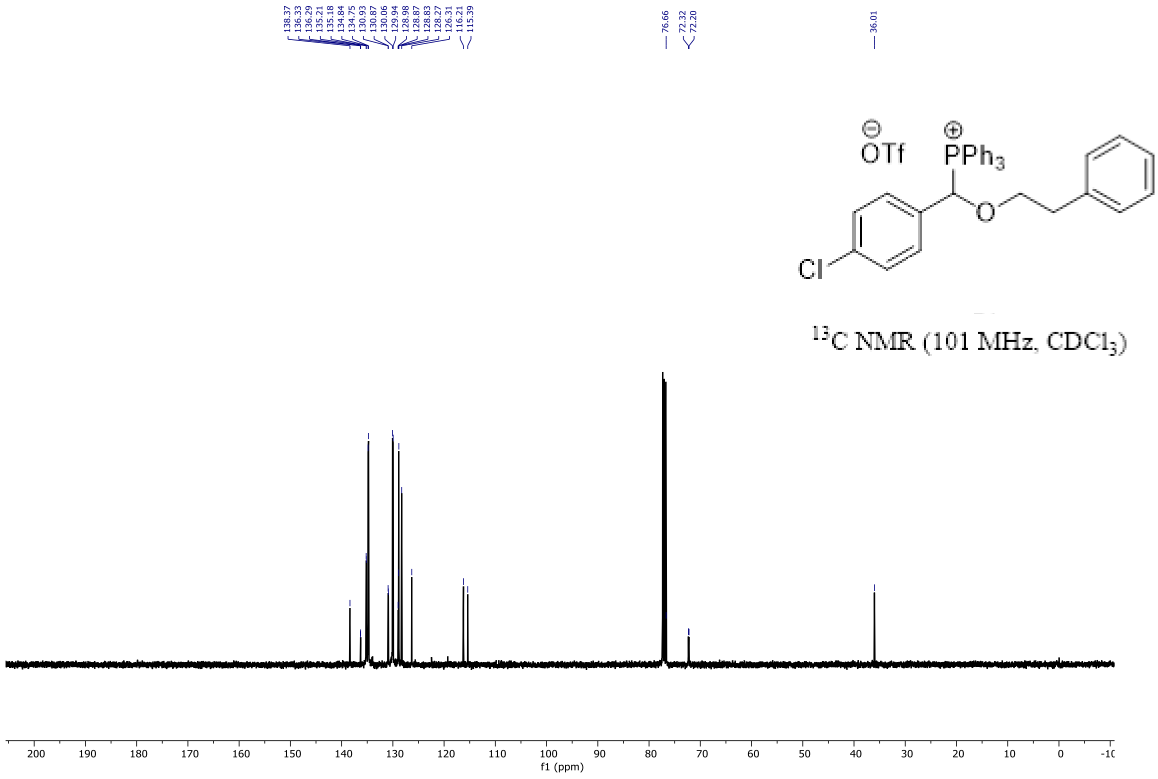

NMR spectra of compound **10D**

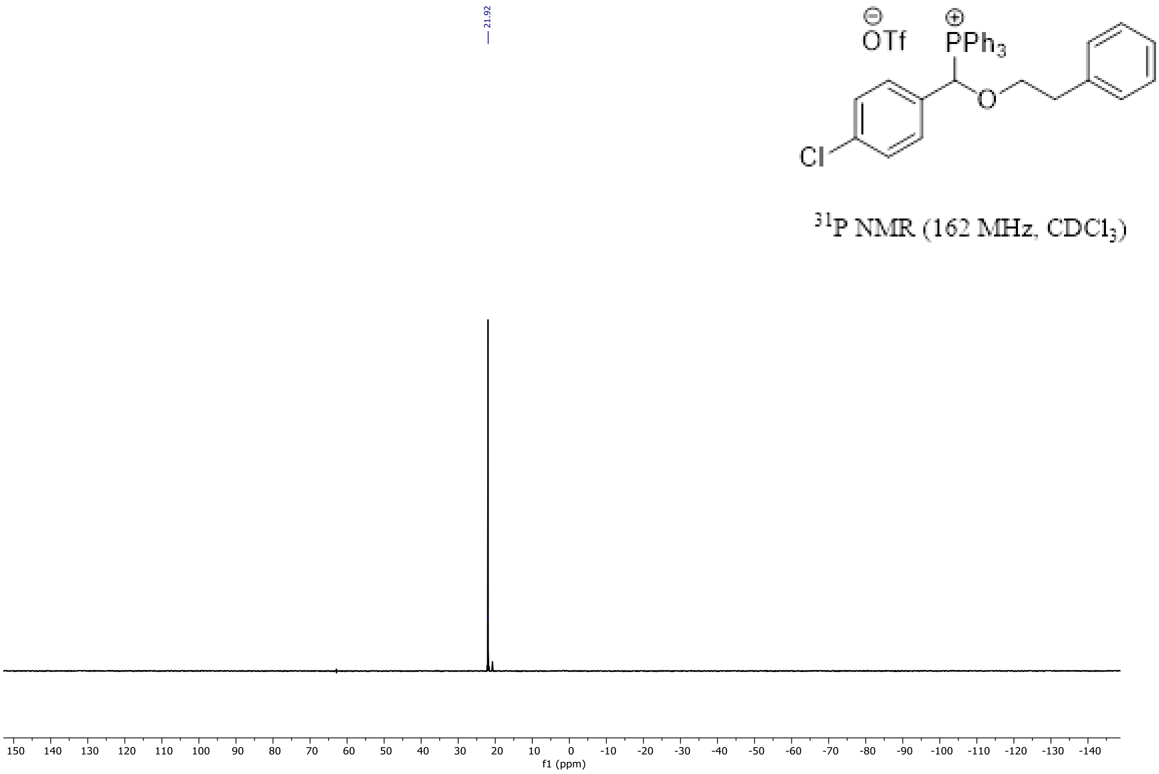

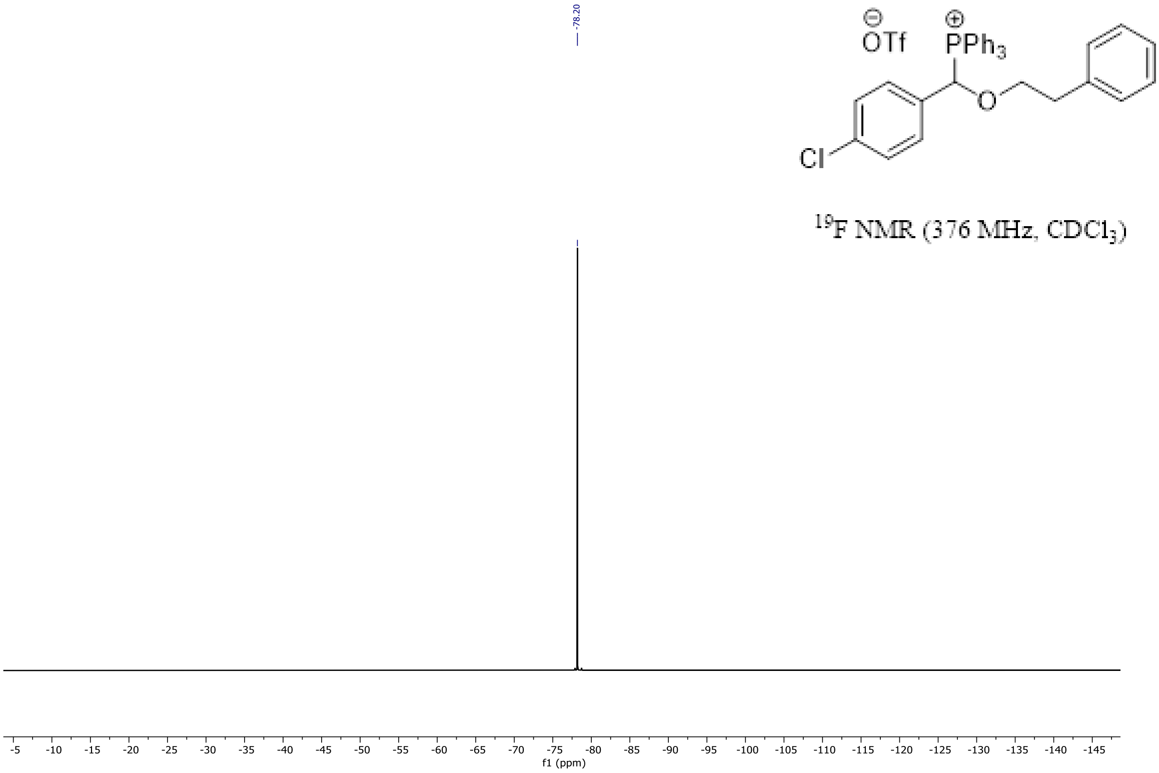

(iii) NMR spectra for ether products (**13a** – **28**)

^{1}H NMR spectrum of compound **13a**

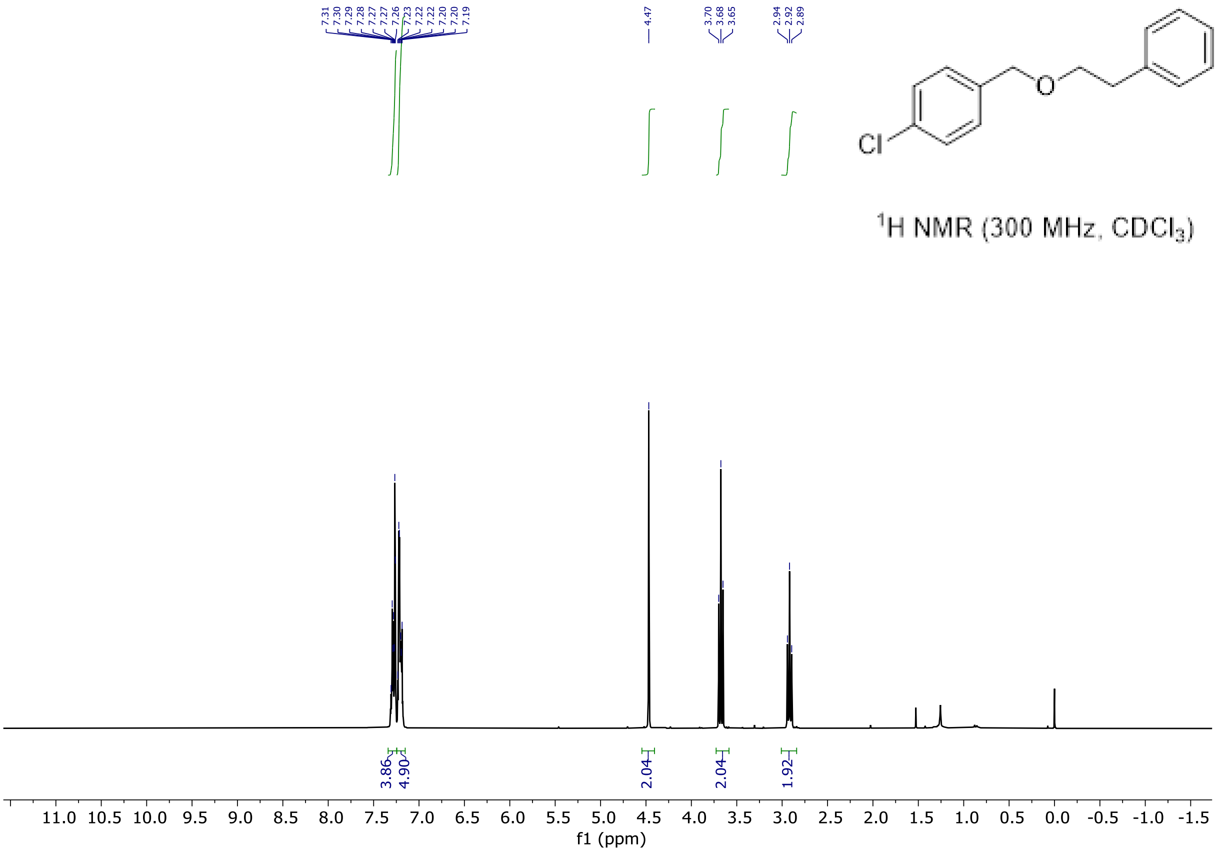

NMR spectra of compound **13b**

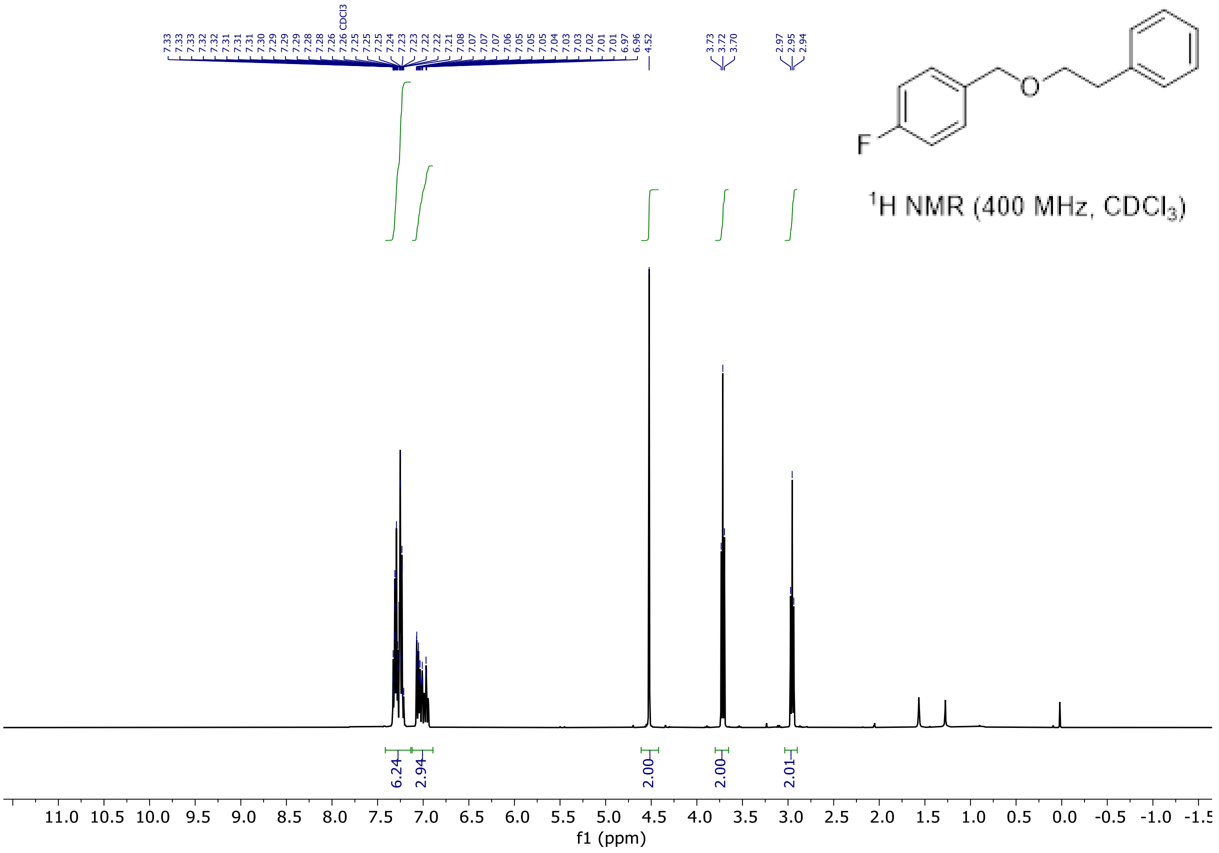

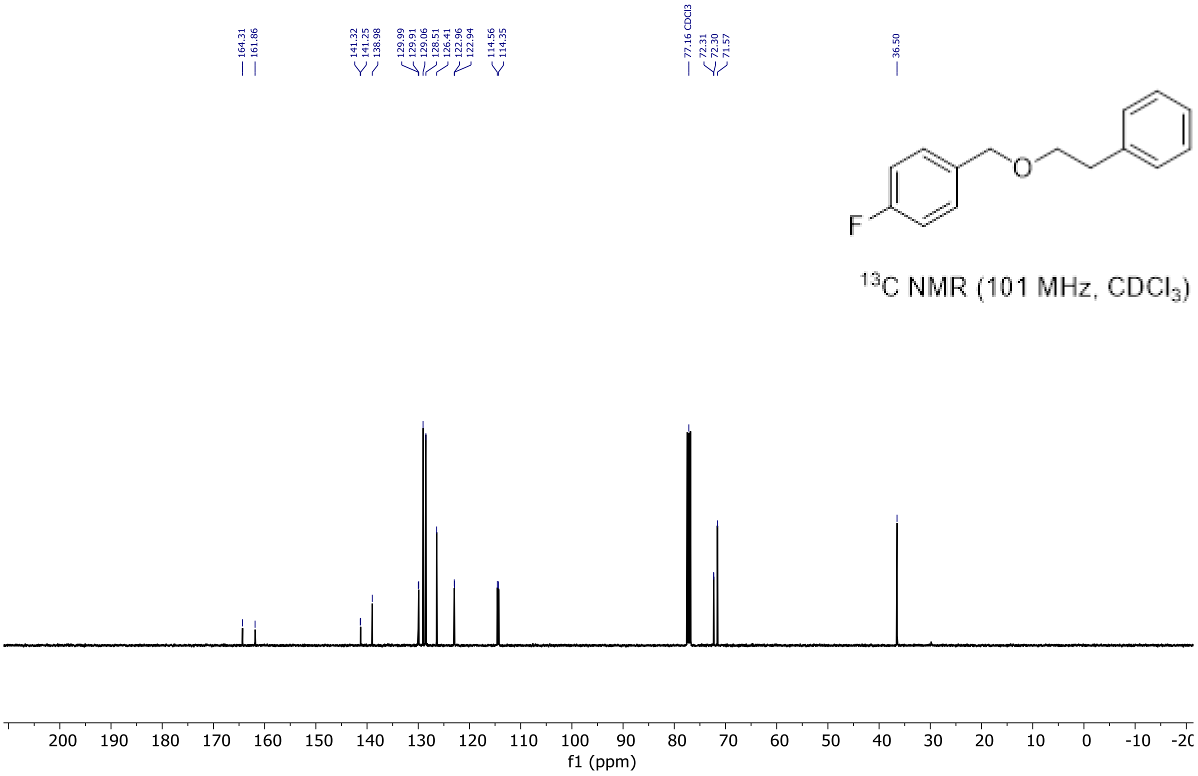

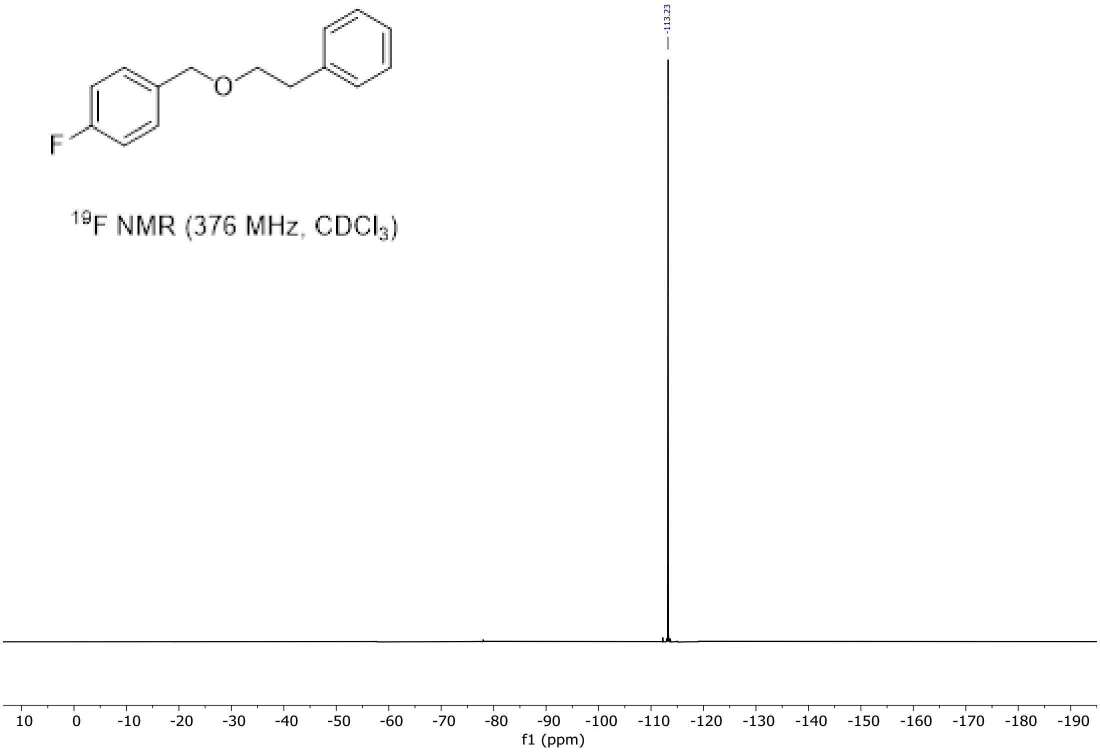
F
O
19F NMR (376 MHz, CDCl3)
-113.23
10 0 -10 -20 -30 -40 -50 -60 -70 -80 -90 -100 -110 -120 -130 -140 -150 -160 -170 -180 -190
f1 (ppm)

NMR spectra of compound **13c**

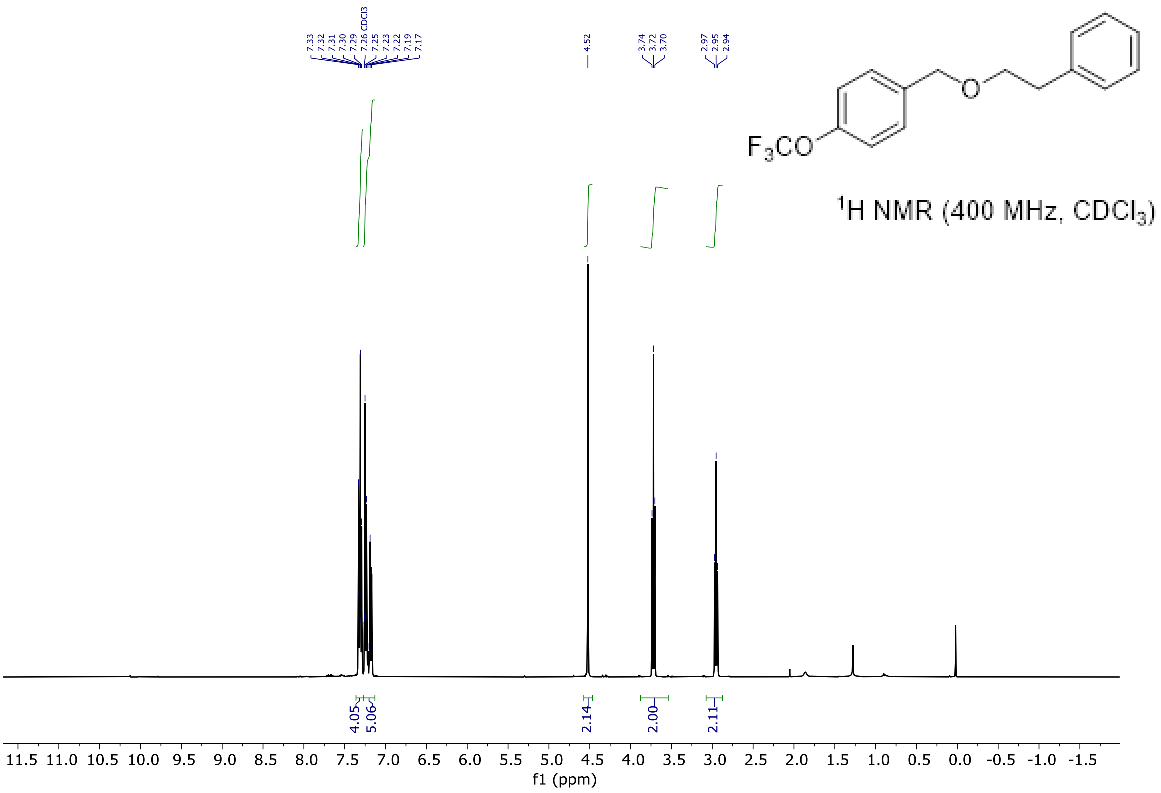

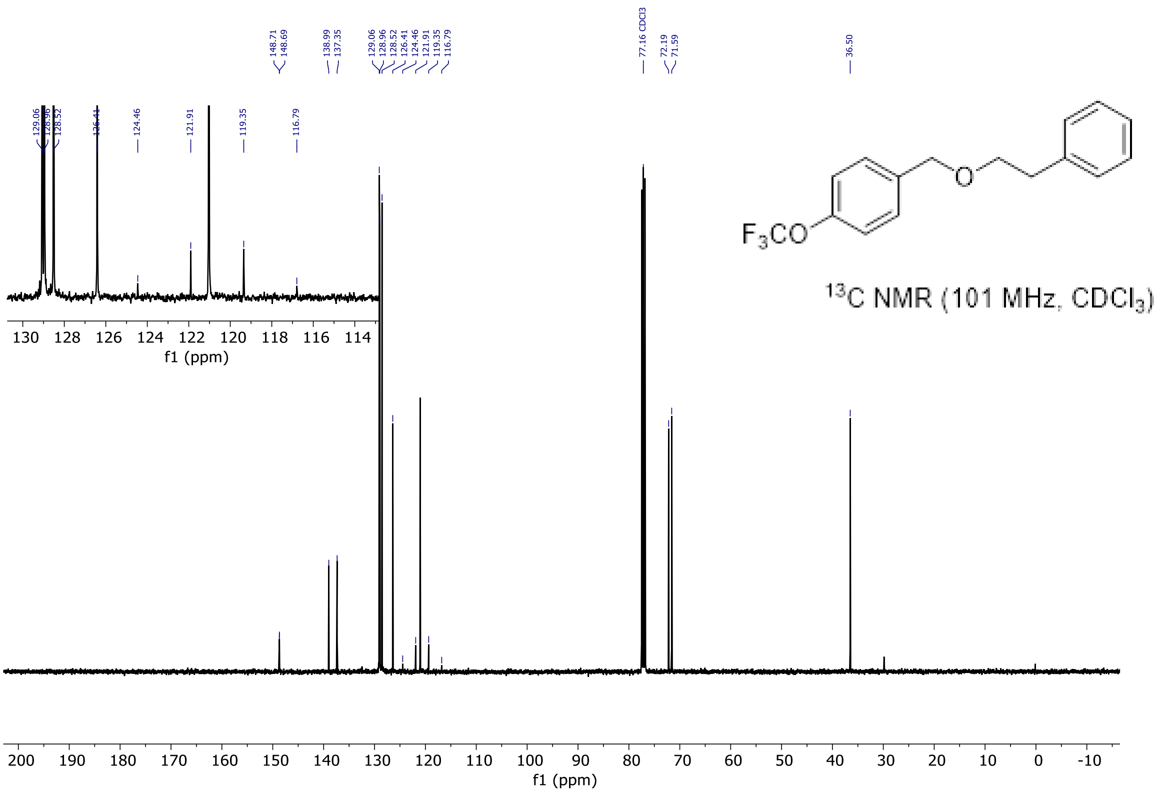

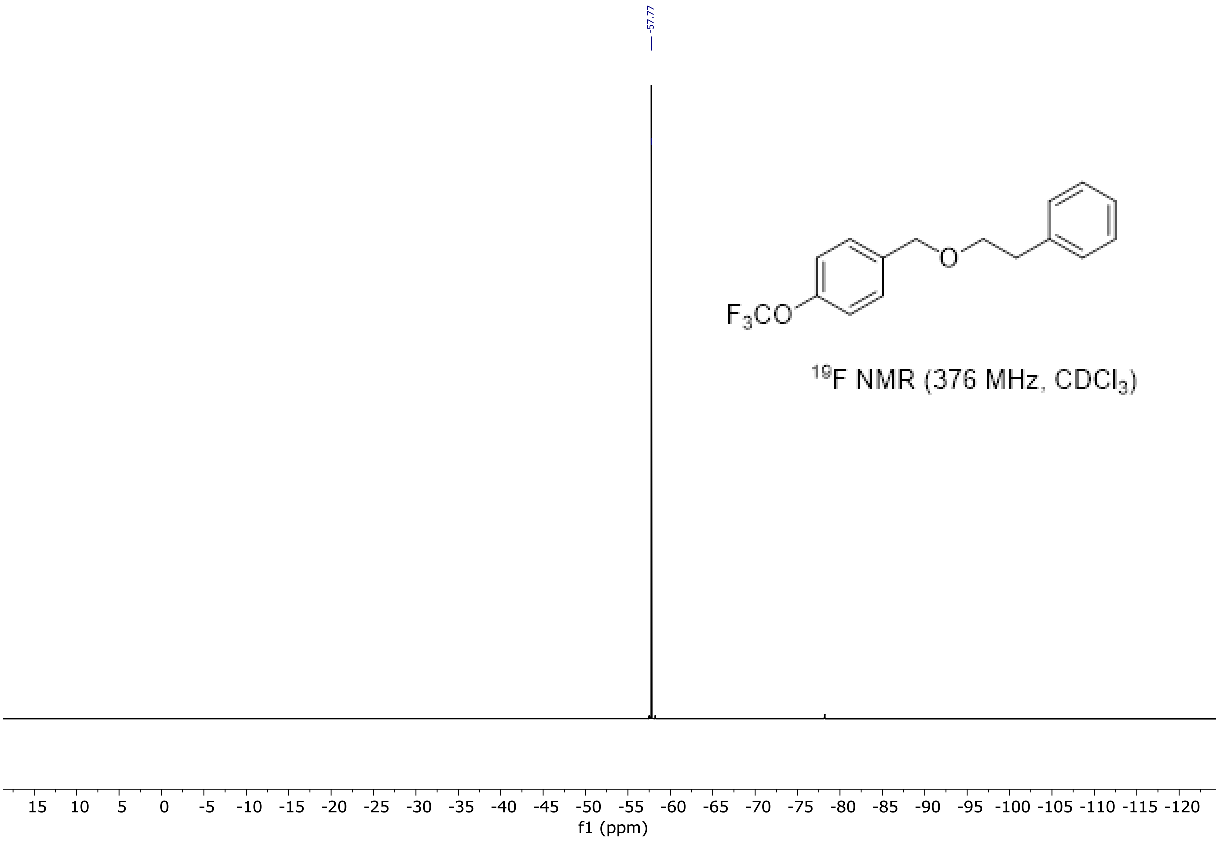
-57.77
F3CO
O
19F NMR (376 MHz, CDCl3)
15 10 5 0 -5 -10 -15 -20 -25 -30 -35 -40 -45 -50 -55 -60 -65 -70 -75 -80 -85 -90 -95 -100 -105 -110 -115 -120
f1 (ppm)

^{1}H NMR spectrum of compound **13d**

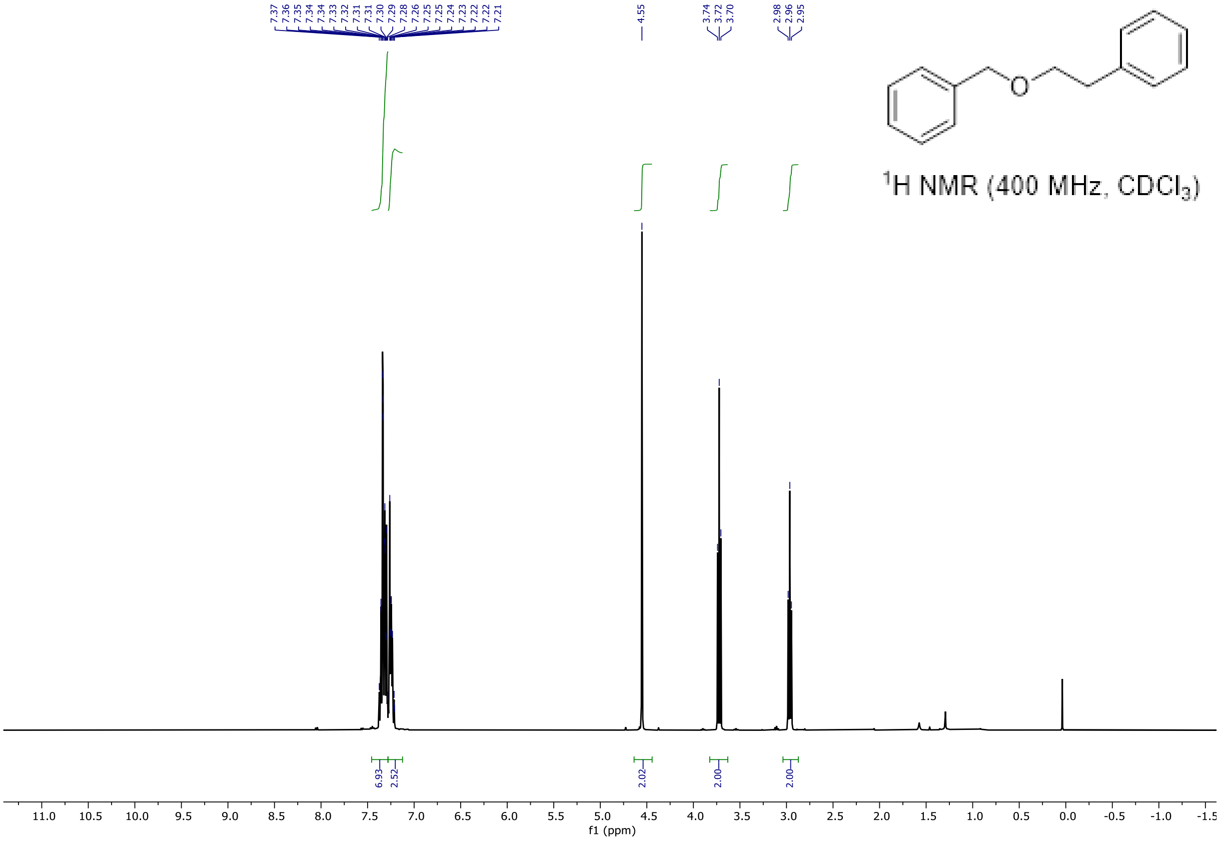

^{1}H NMR spectrum of compound **13e**

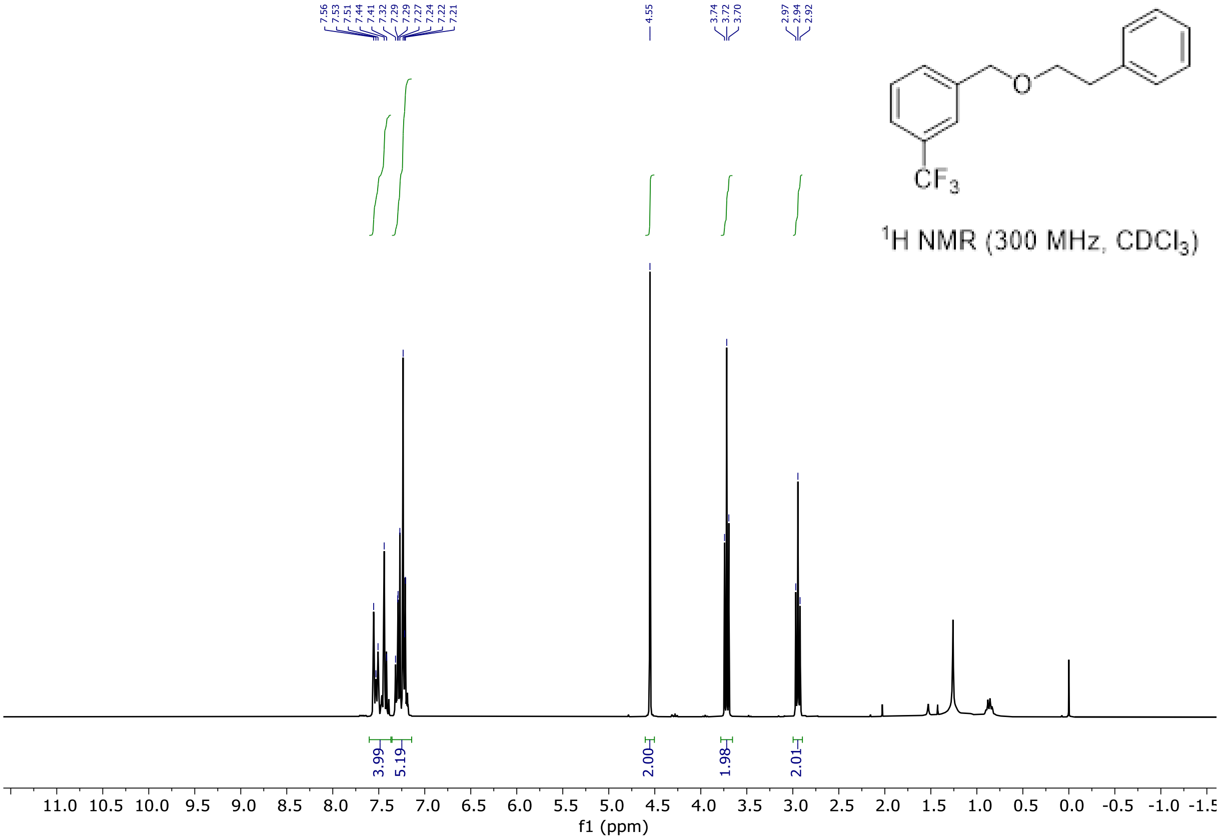

^{1}H NMR spectrum of compound **13f**

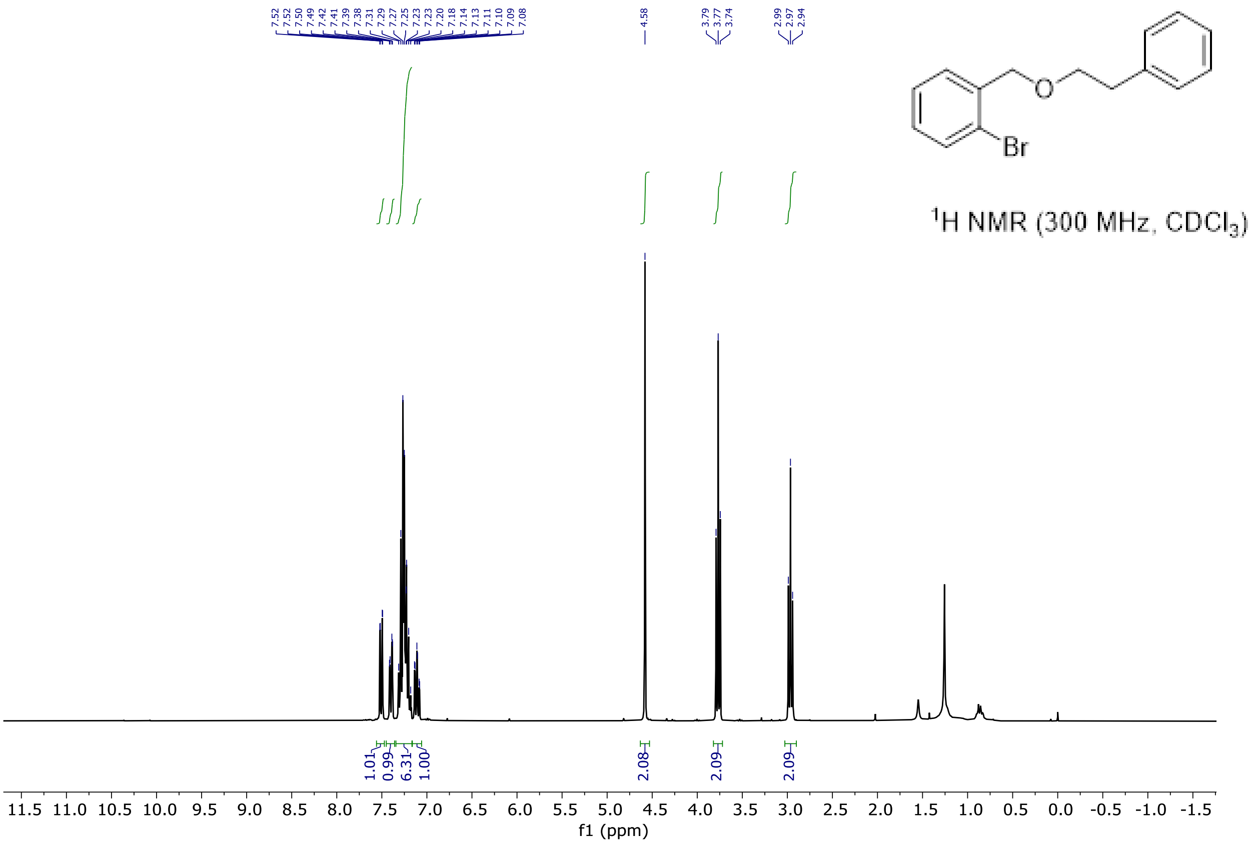

^{1}H NMR spectrum of compound **14a**

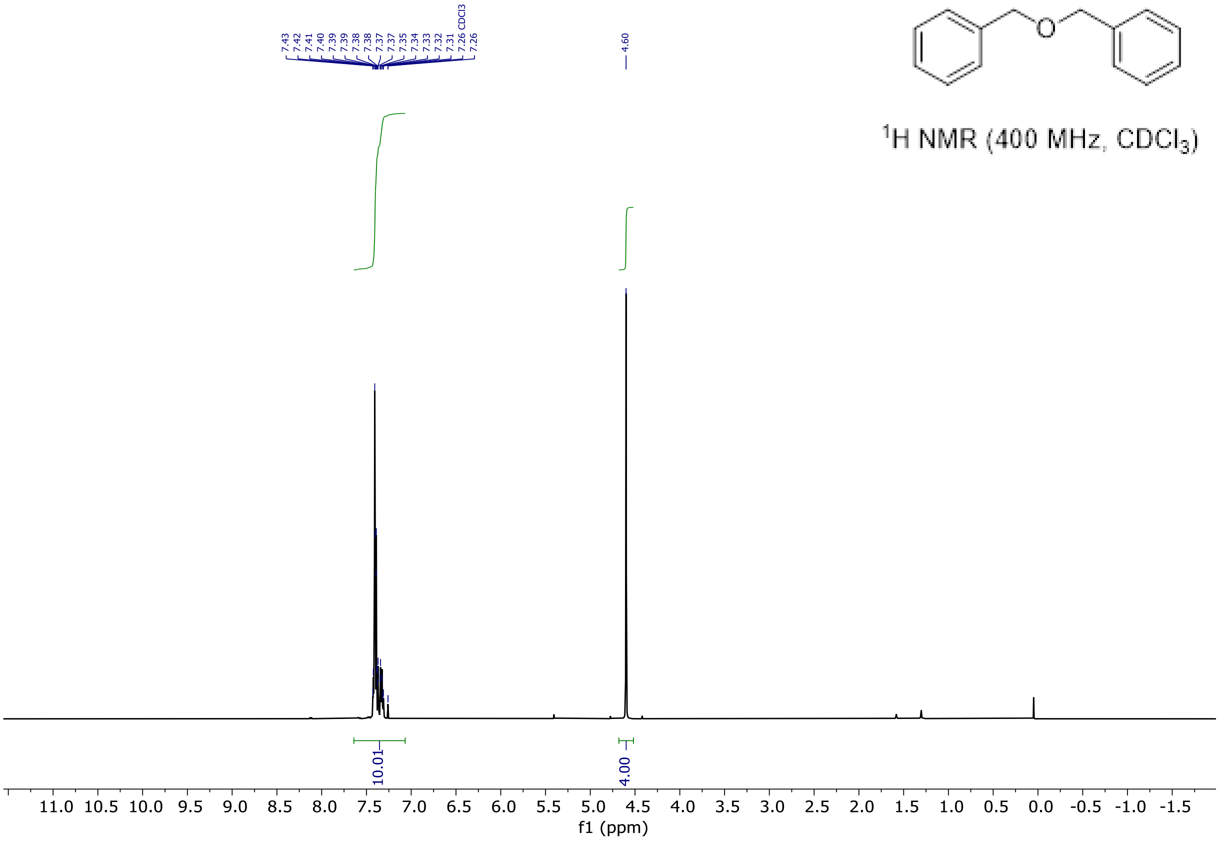

^{1}H NMR spectrum of compound **14b**

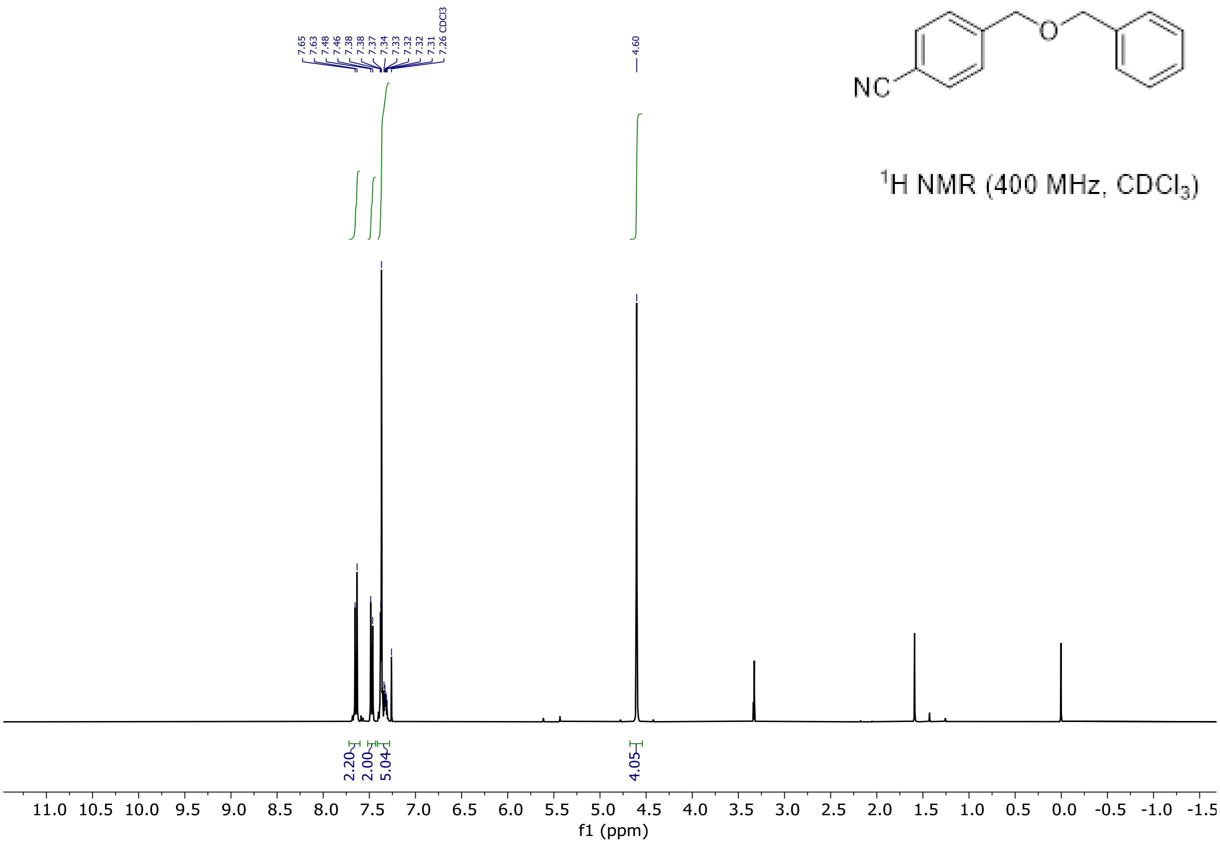

NMR spectra of compound **14c**

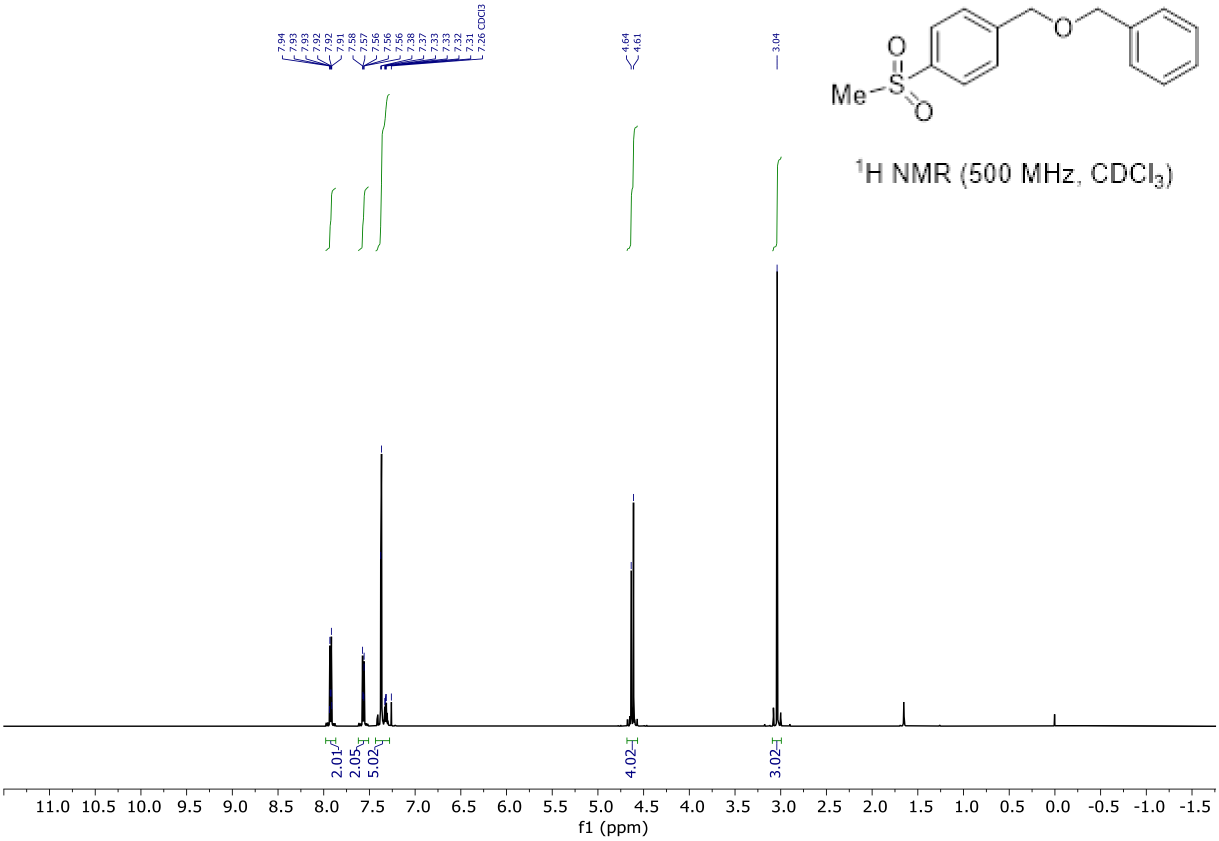

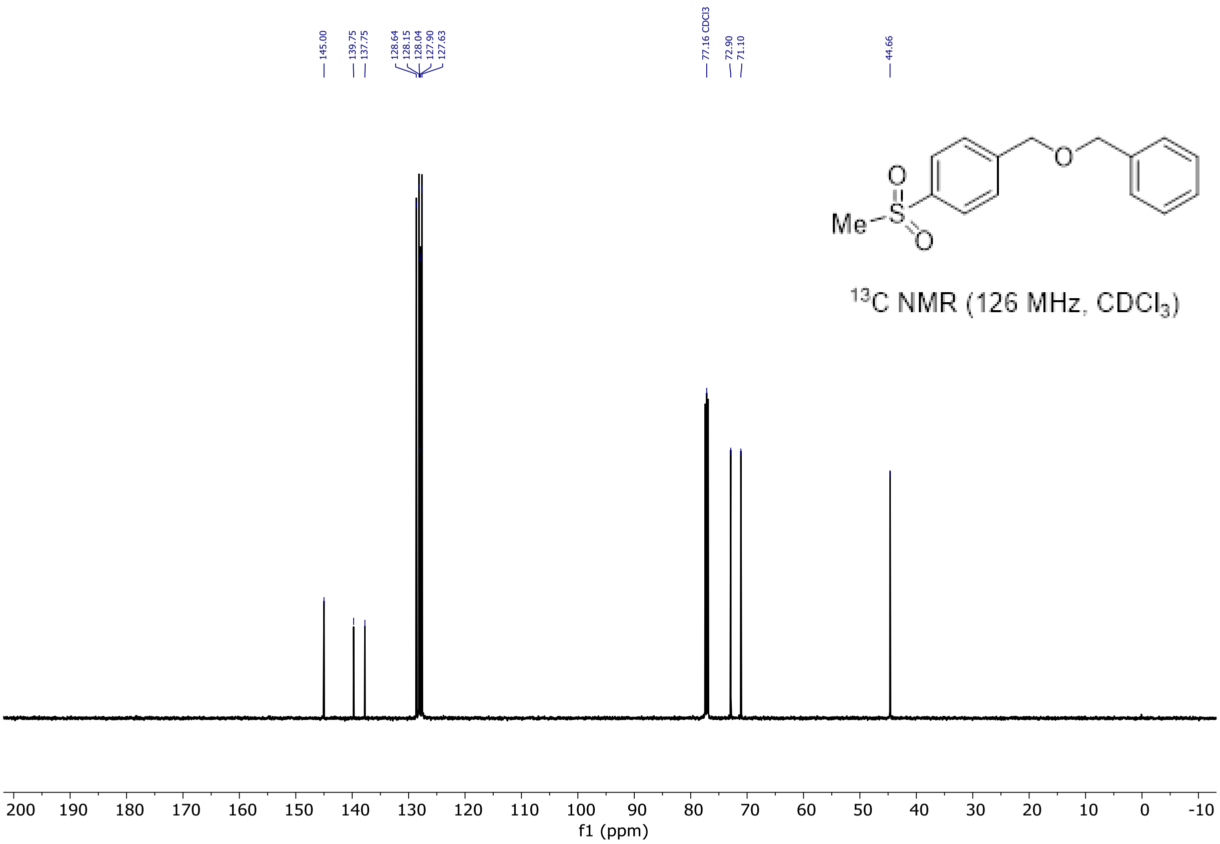

^{1}H NMR spectrum of compound **14d**

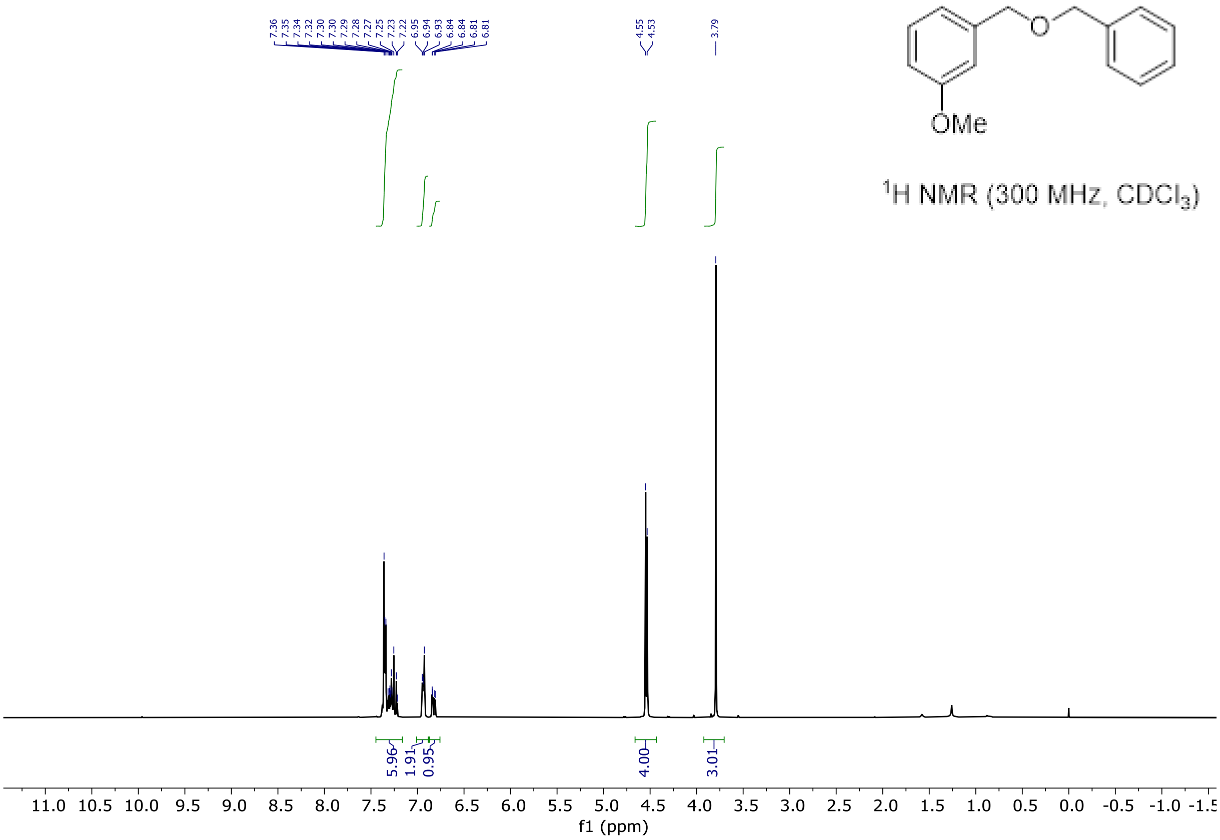

^{1}H NMR spectrum of compound **14e**

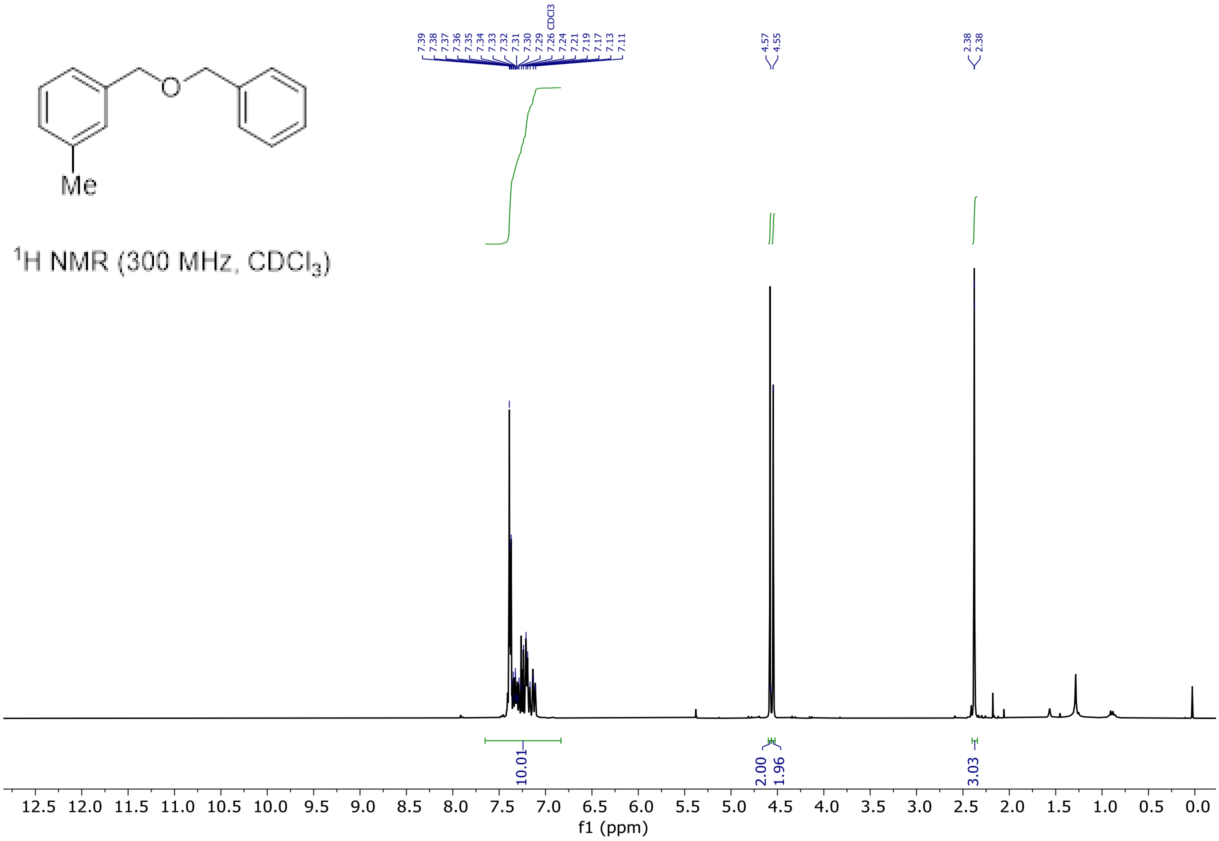

^{1}H NMR spectrum of compound **14f**

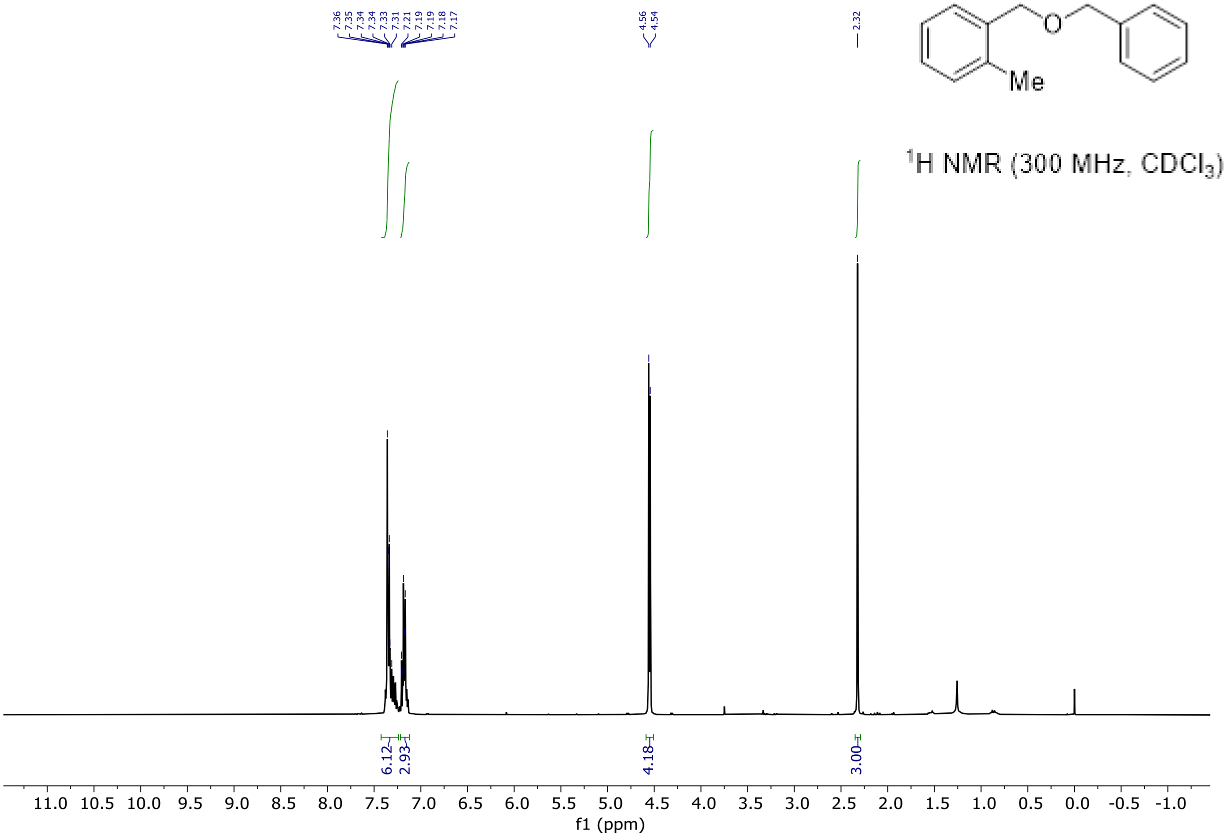

^{1}H NMR spectrum of compound **14g**

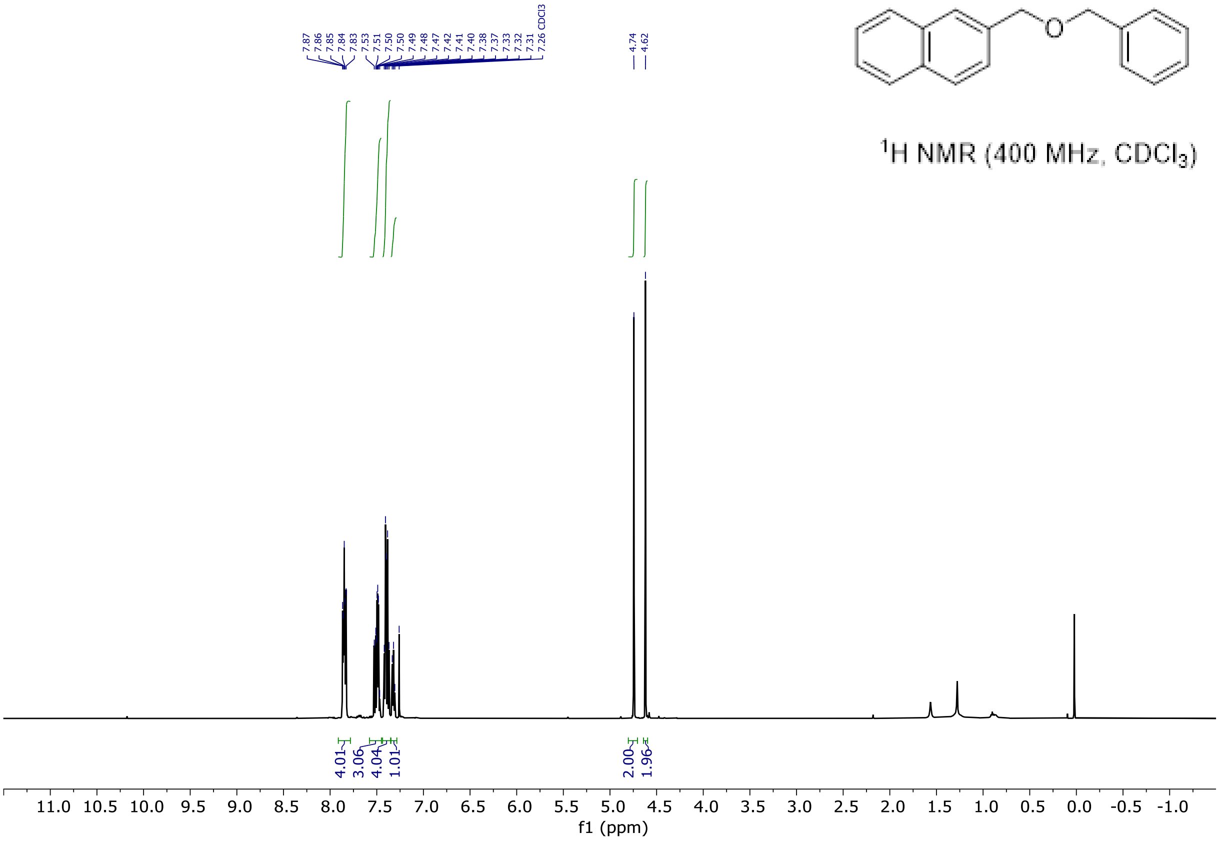

^{1}H NMR spectrum of compound **14h**

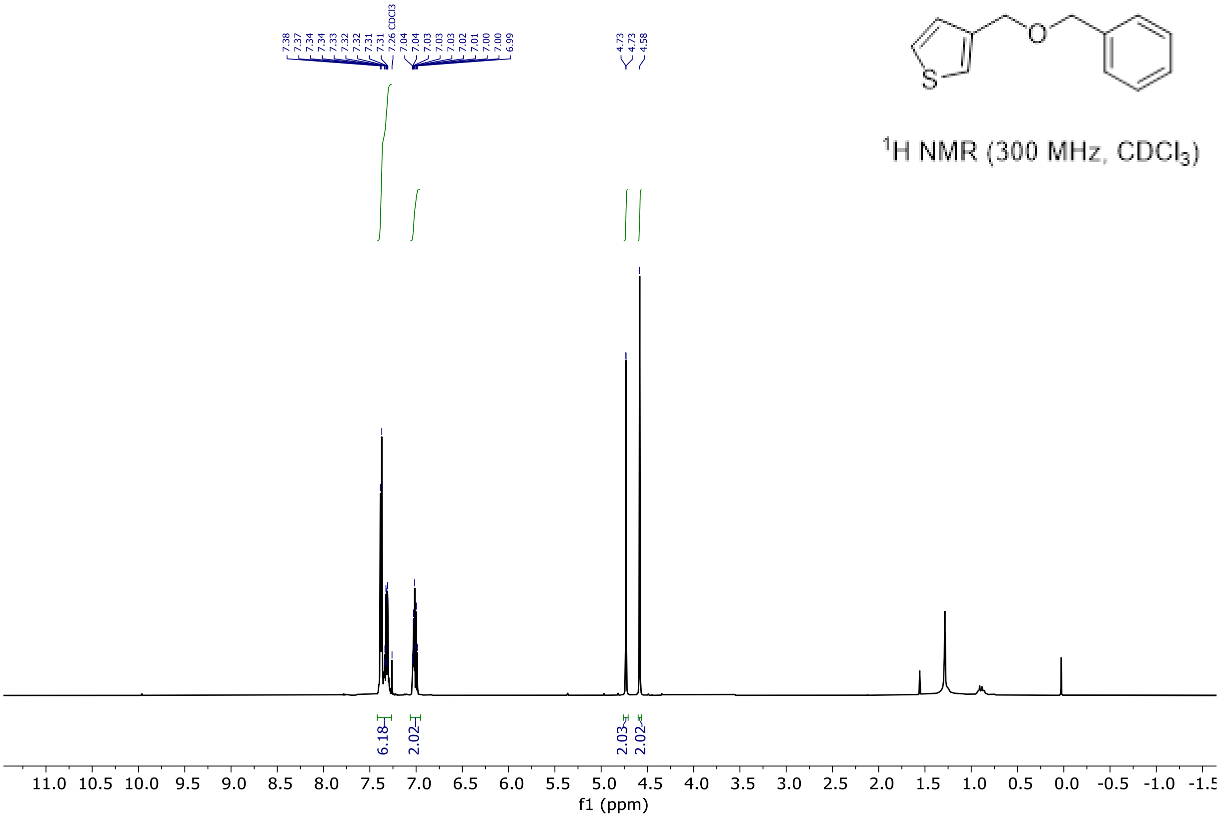

^{1}H NMR spectrum of compound **14i**

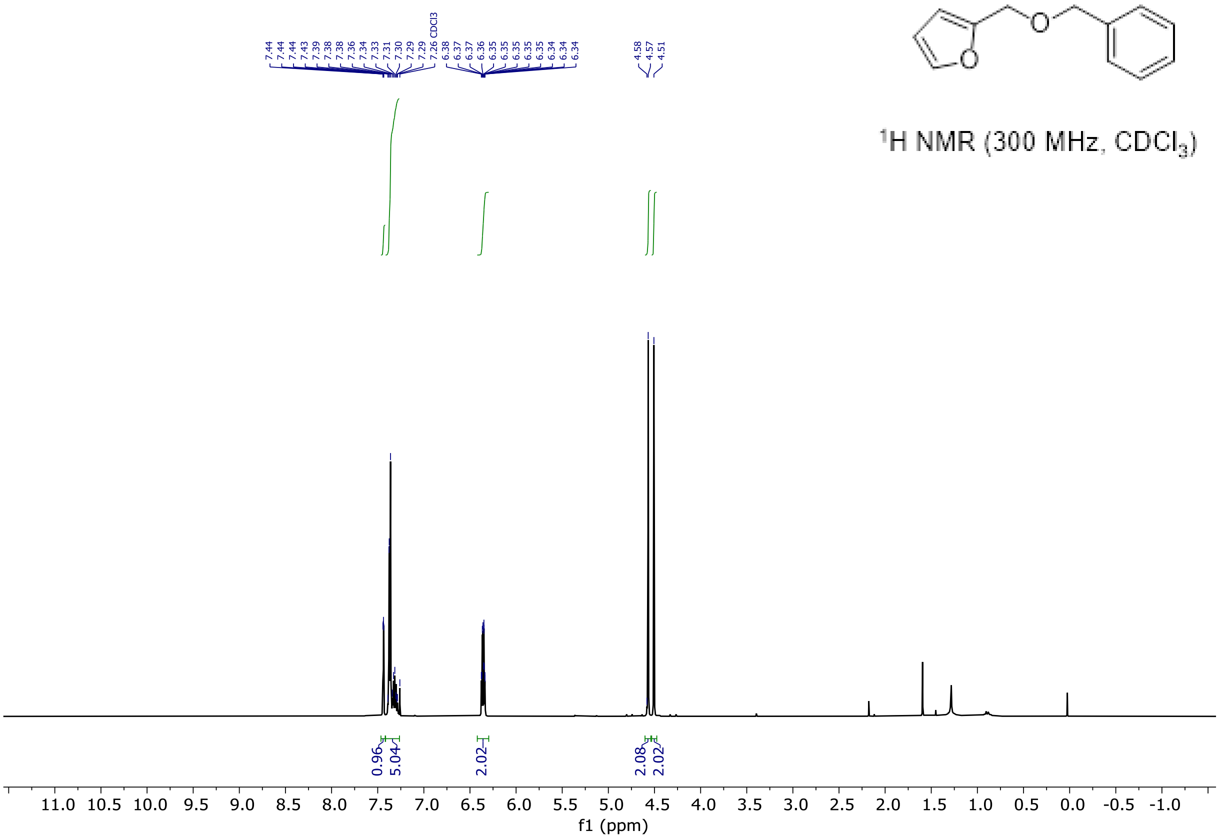

^{1}H NMR spectrum of compound **14j**

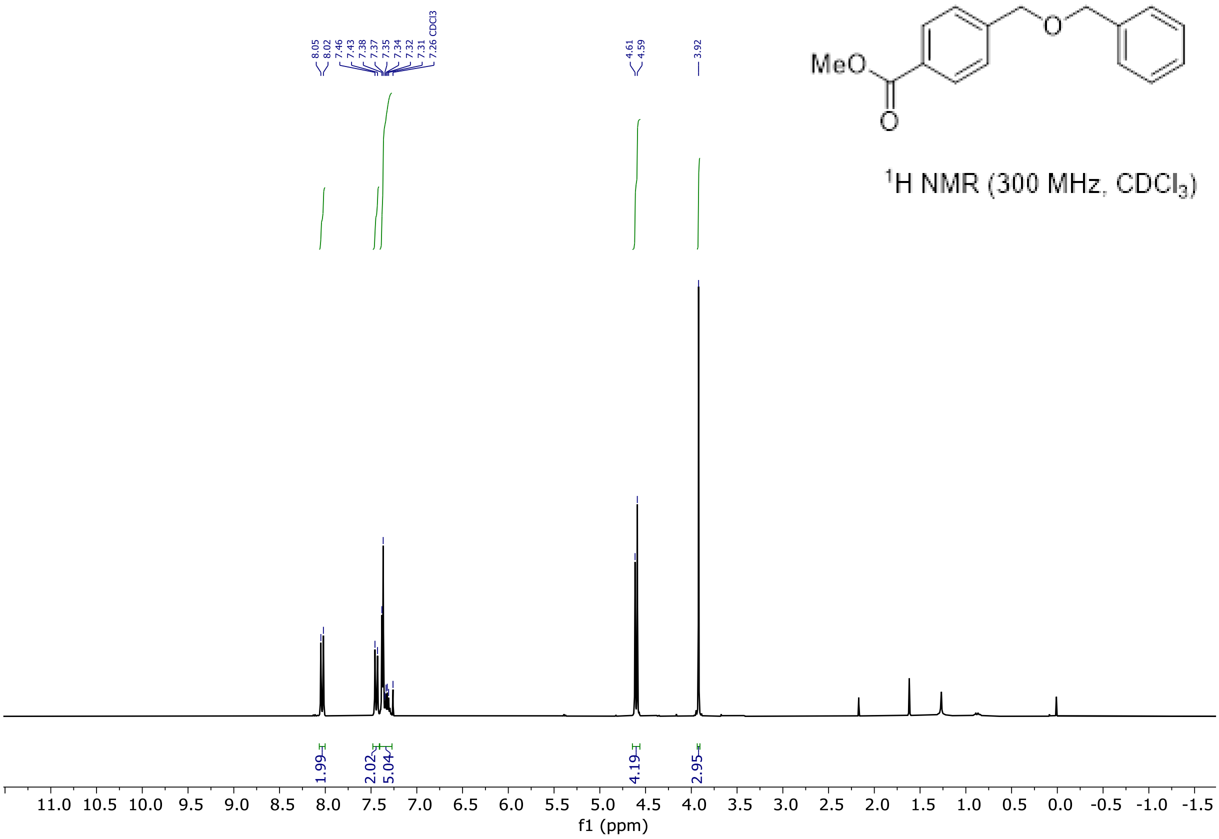

^{1}H NMR spectrum of compound **14k**

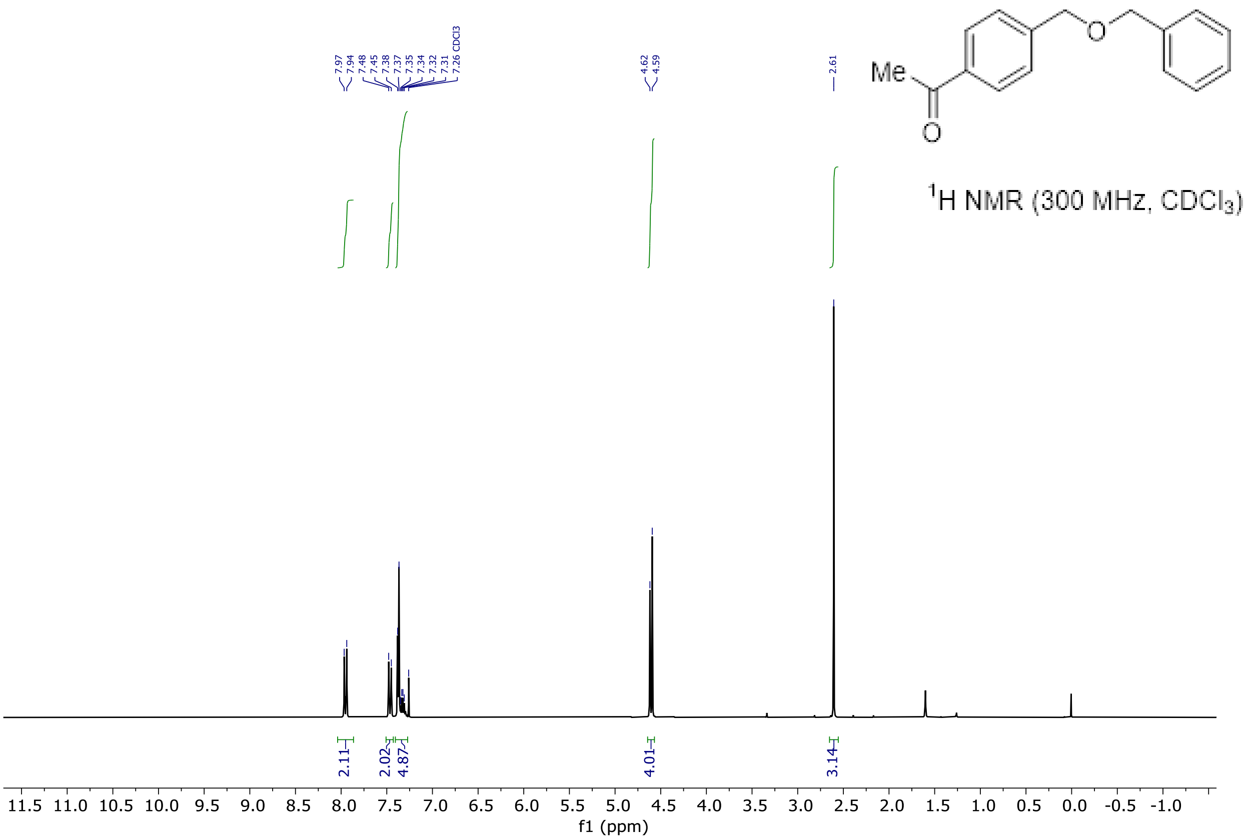

NMR spectra of compound **14l**

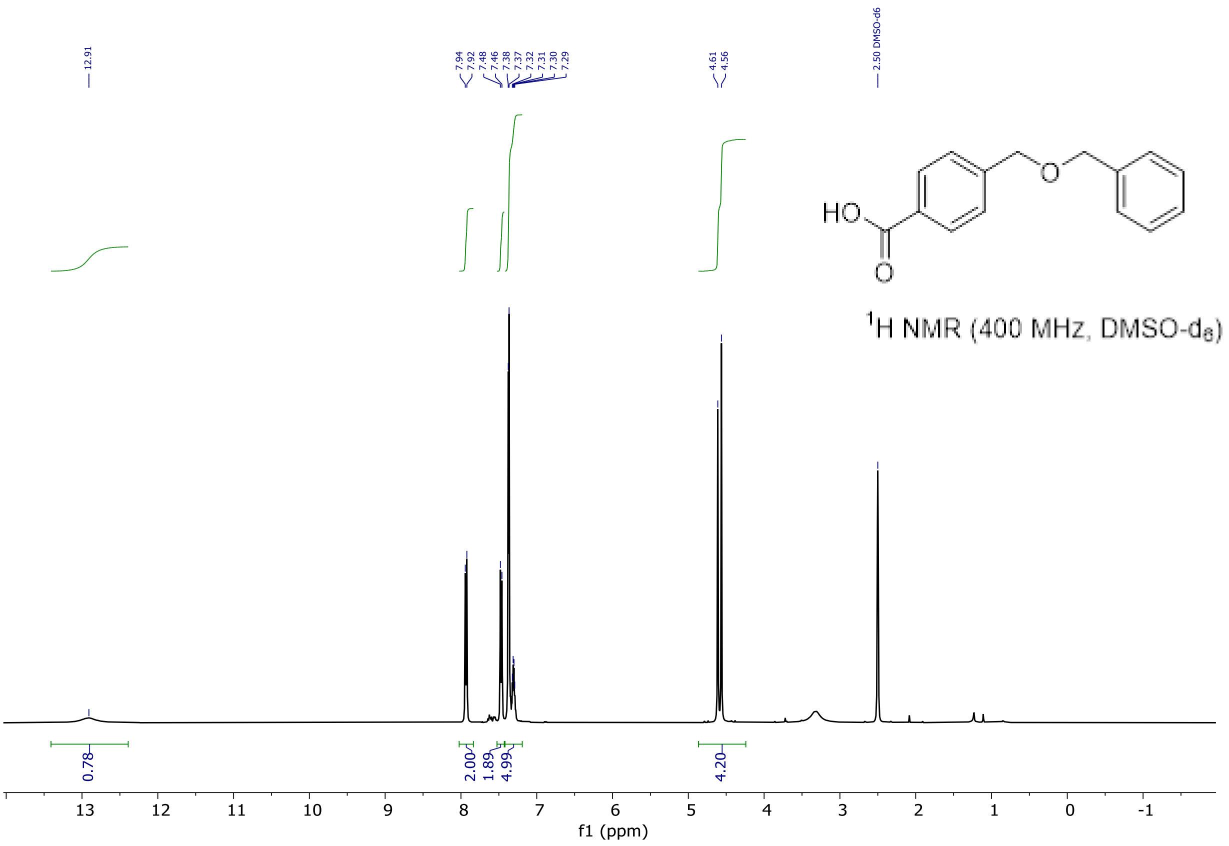

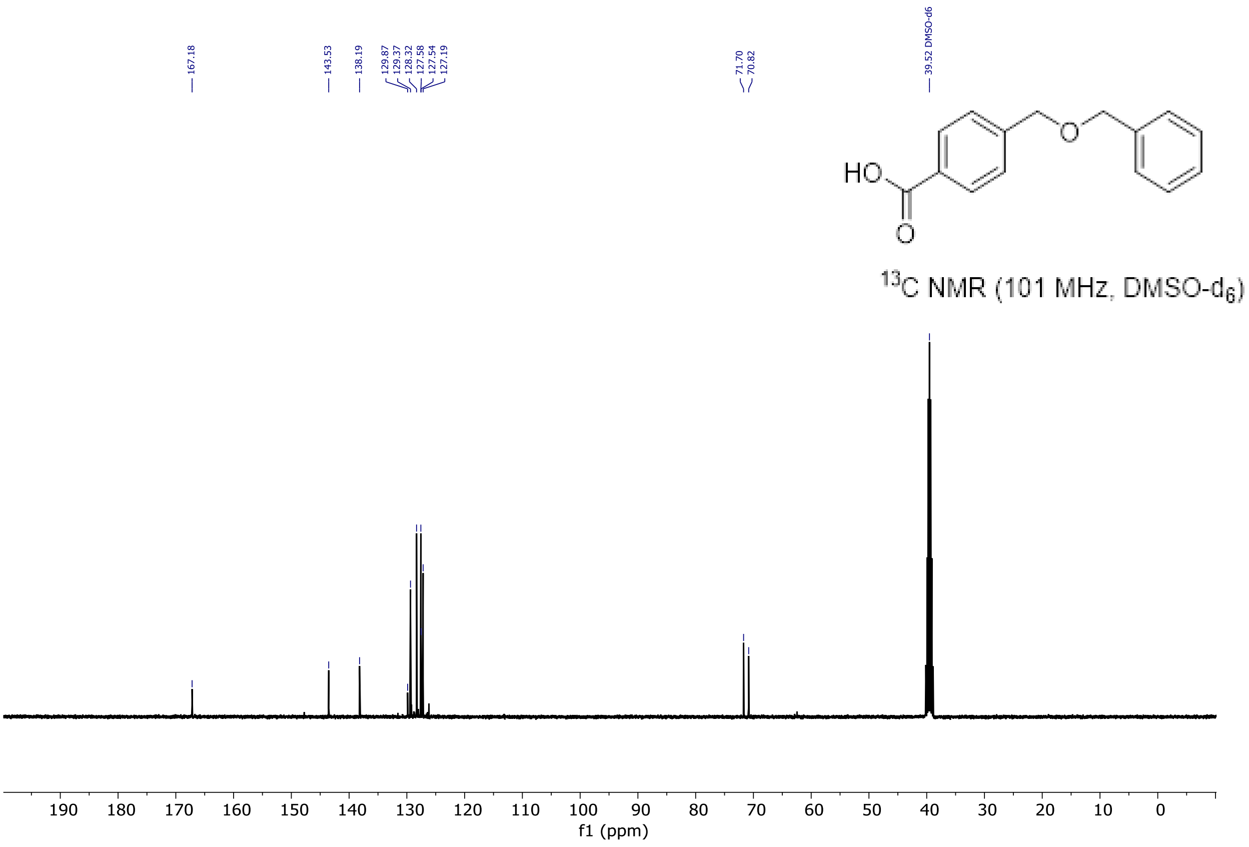

NMR spectra of compound **14m**

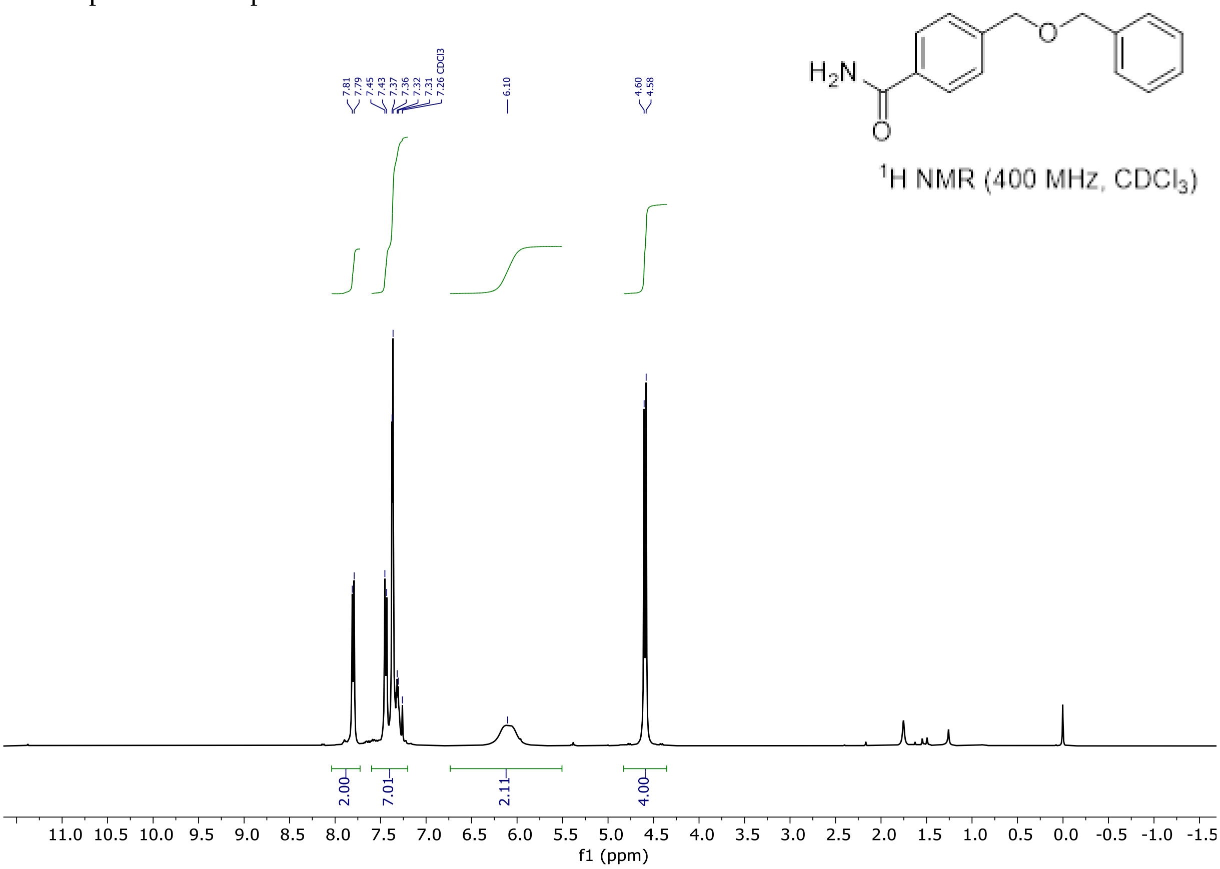

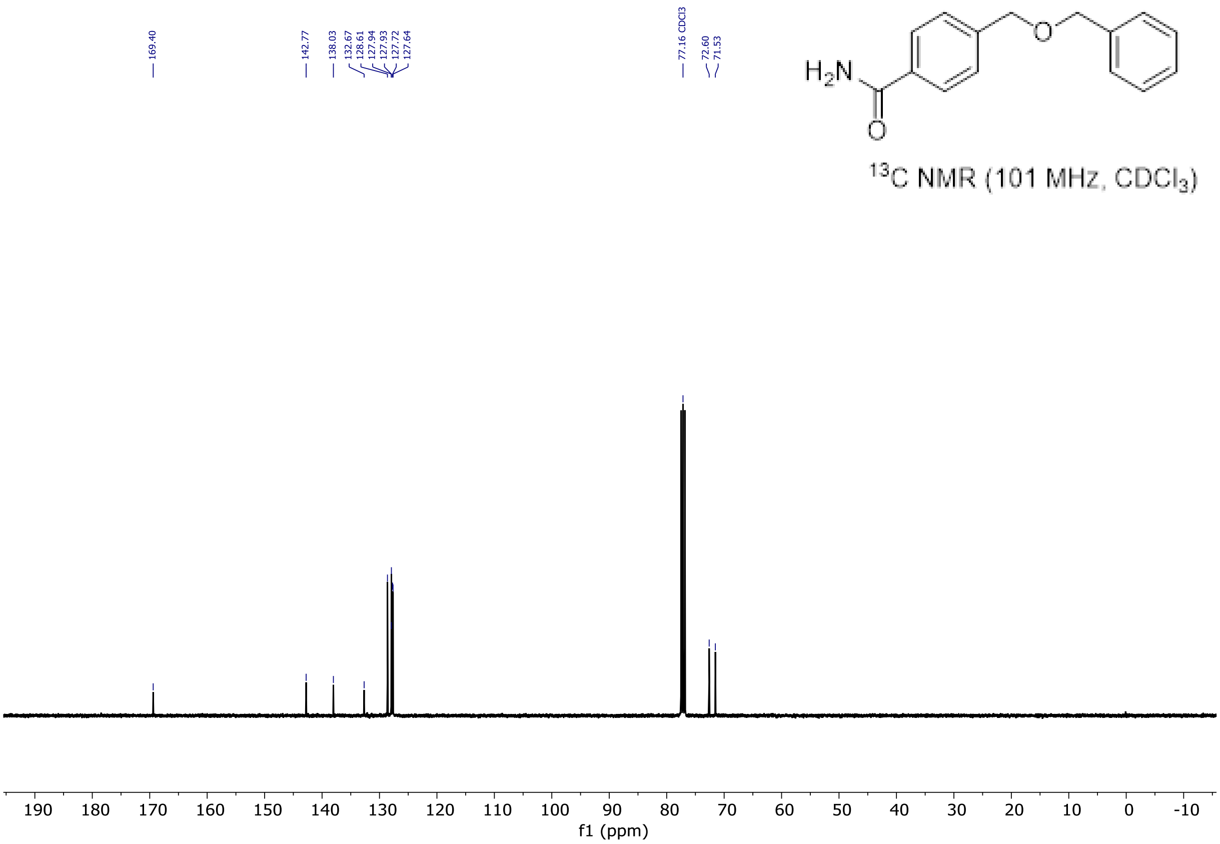

^{1}H NMR spectrum of compound **15**

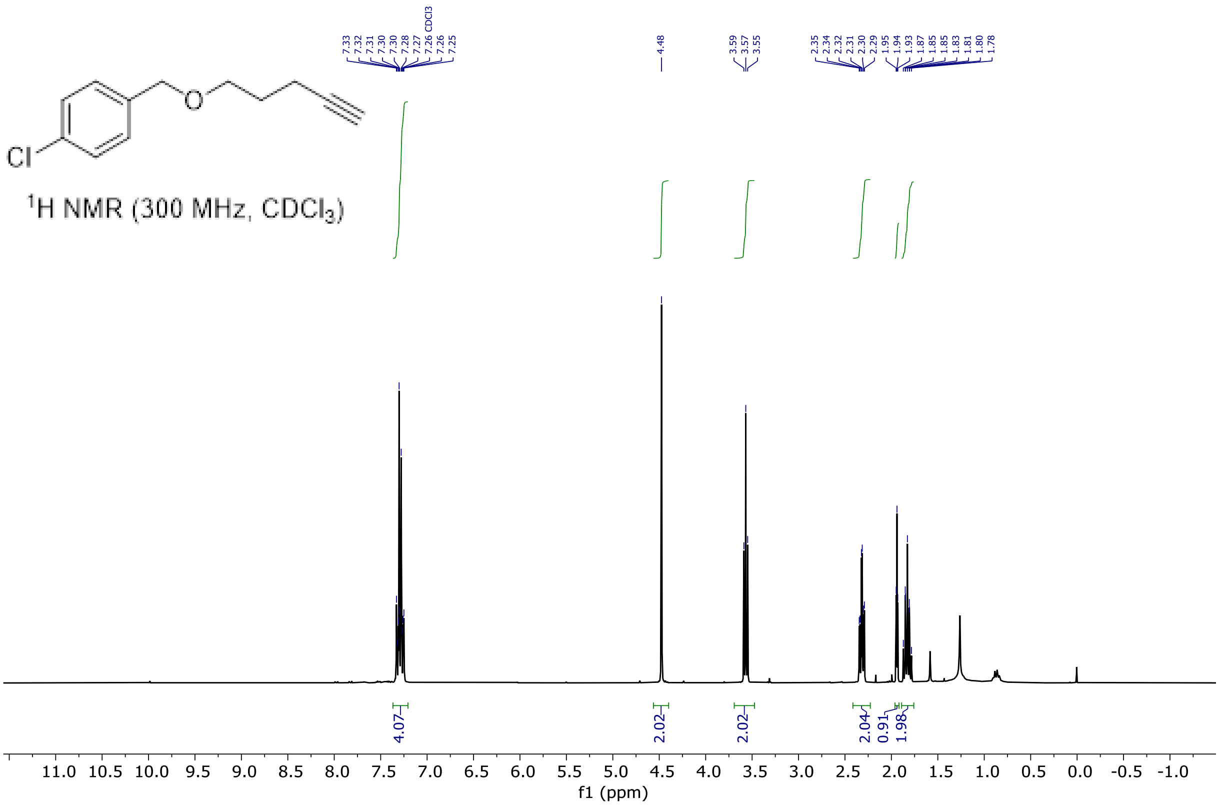

NMR spectra of compound **16**

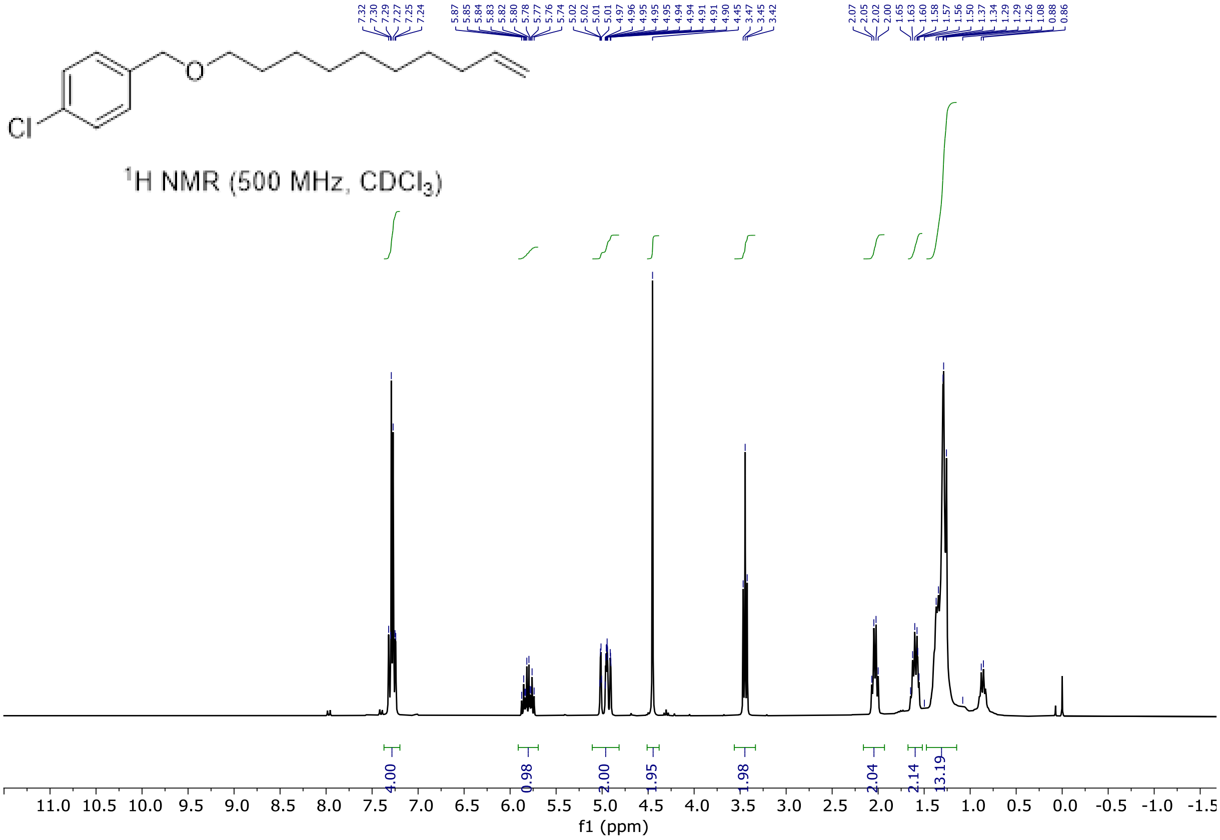

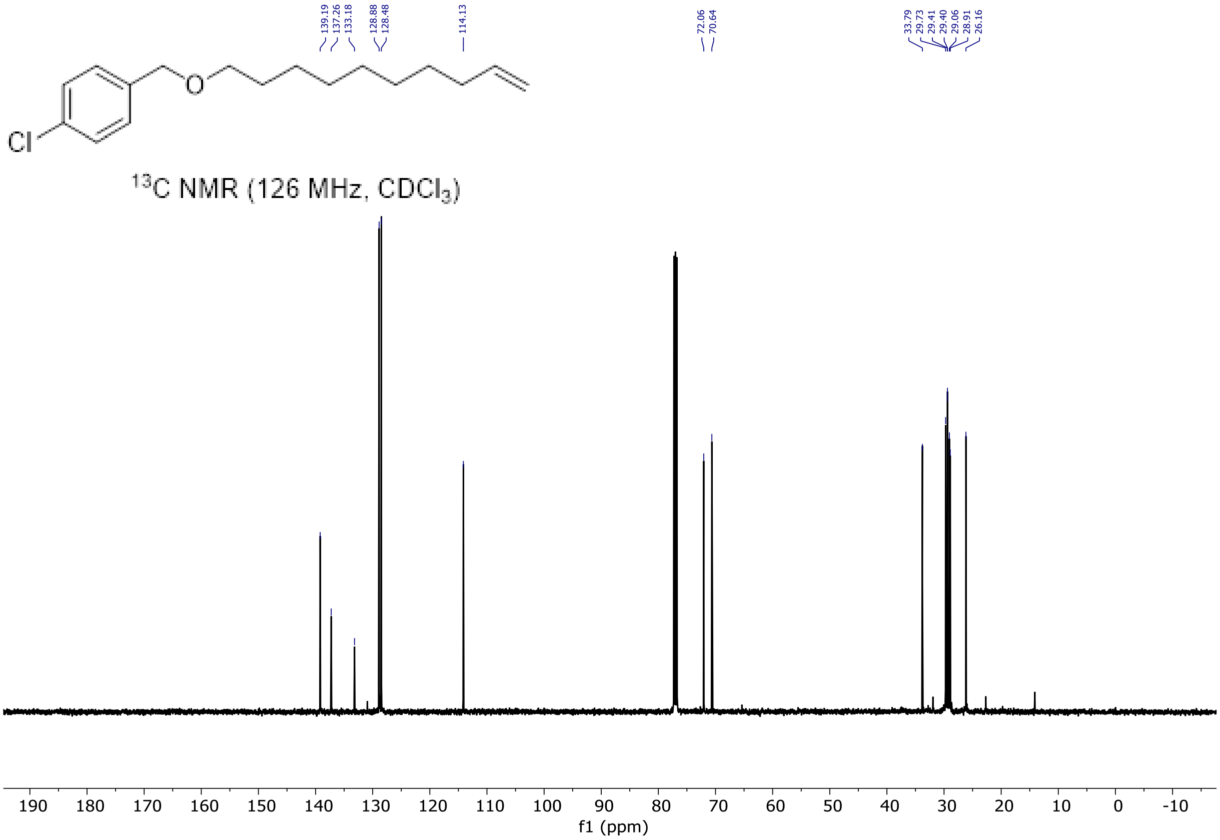

^{1}H NMR spectrum of compound **17**

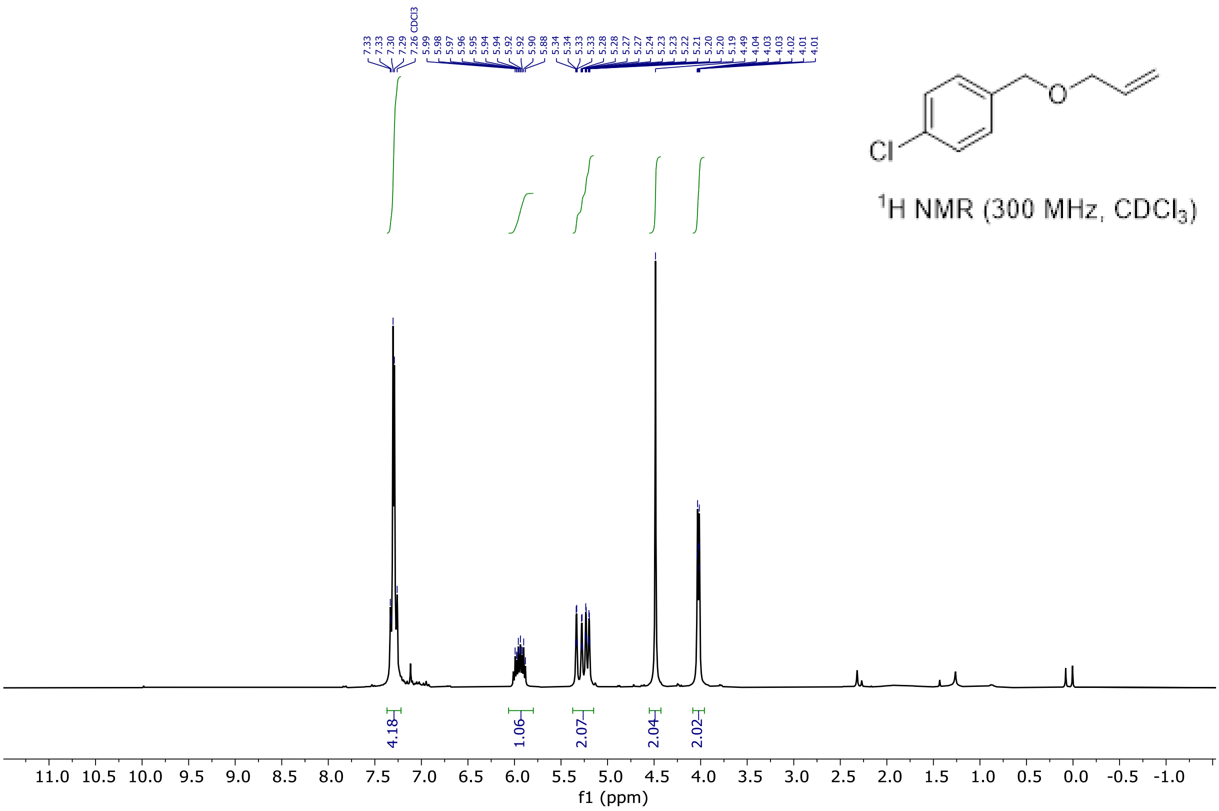

NMR spectra of compound **18**

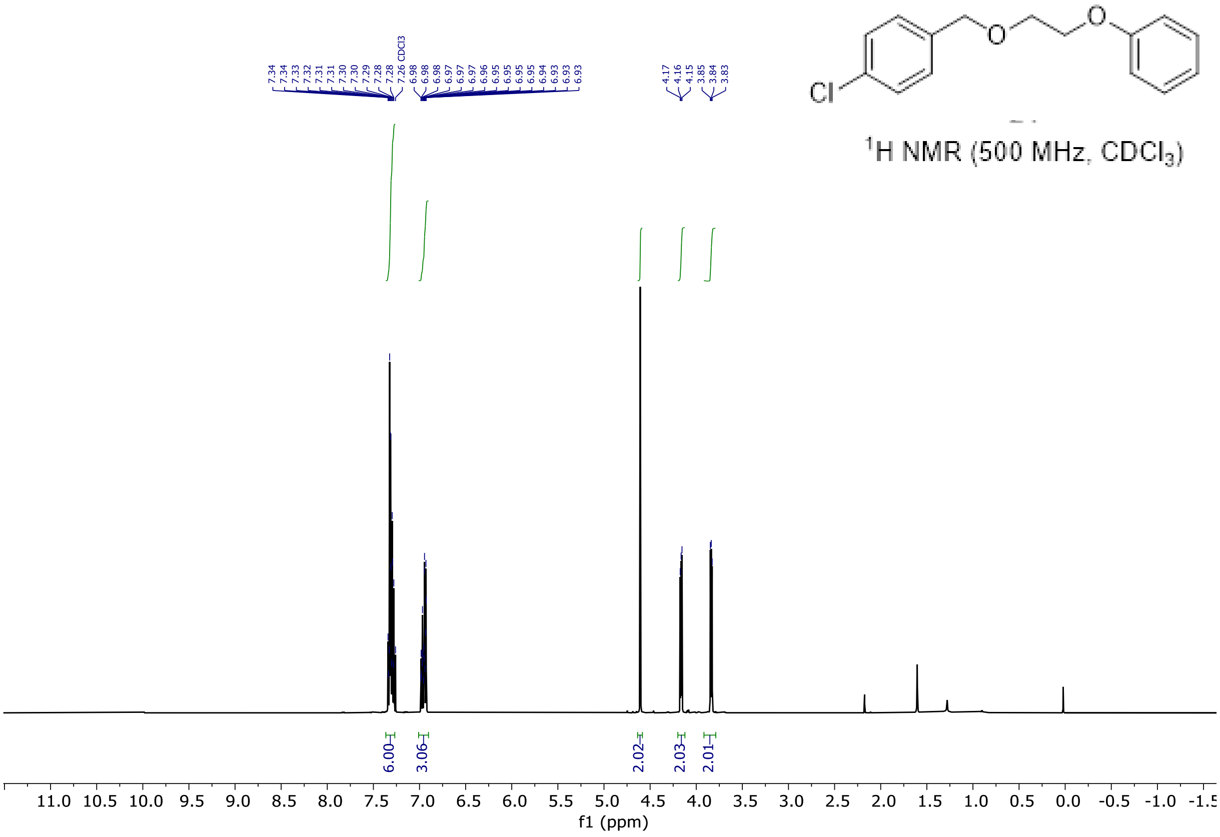

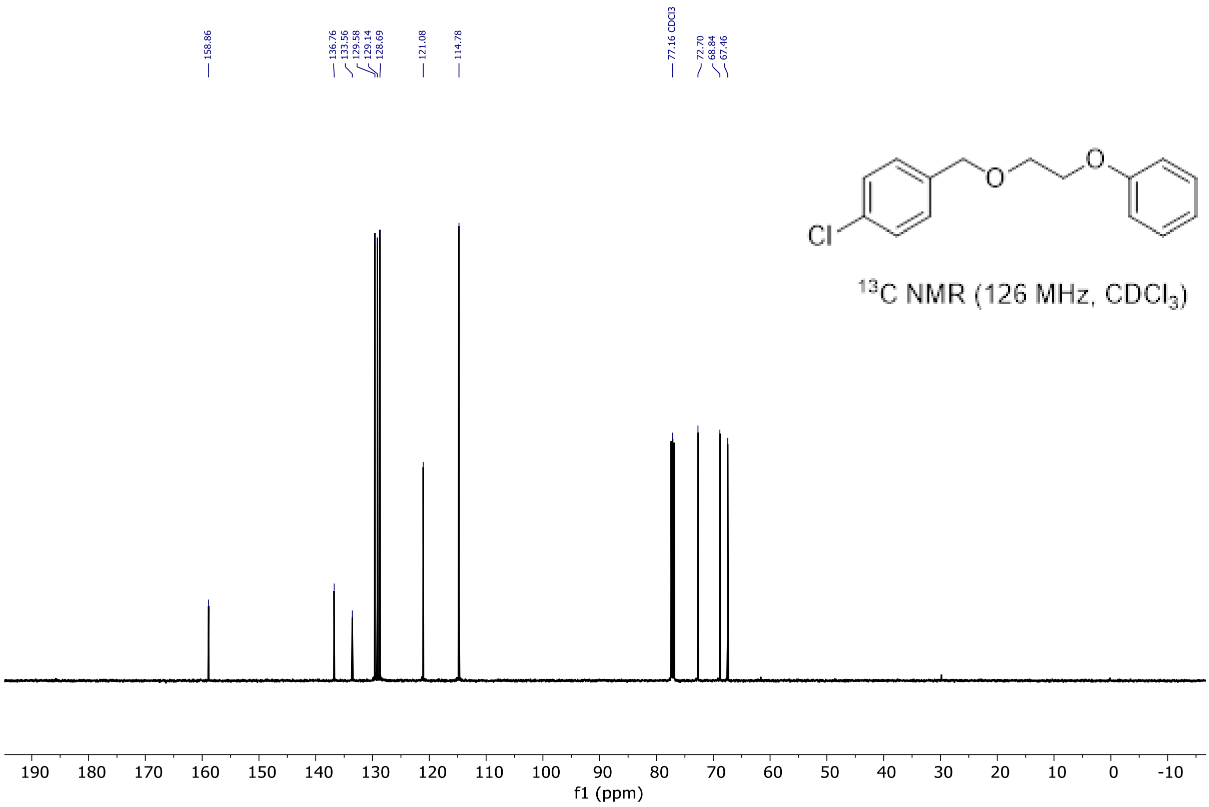

^{1}H NMR spectrum of compound **19**

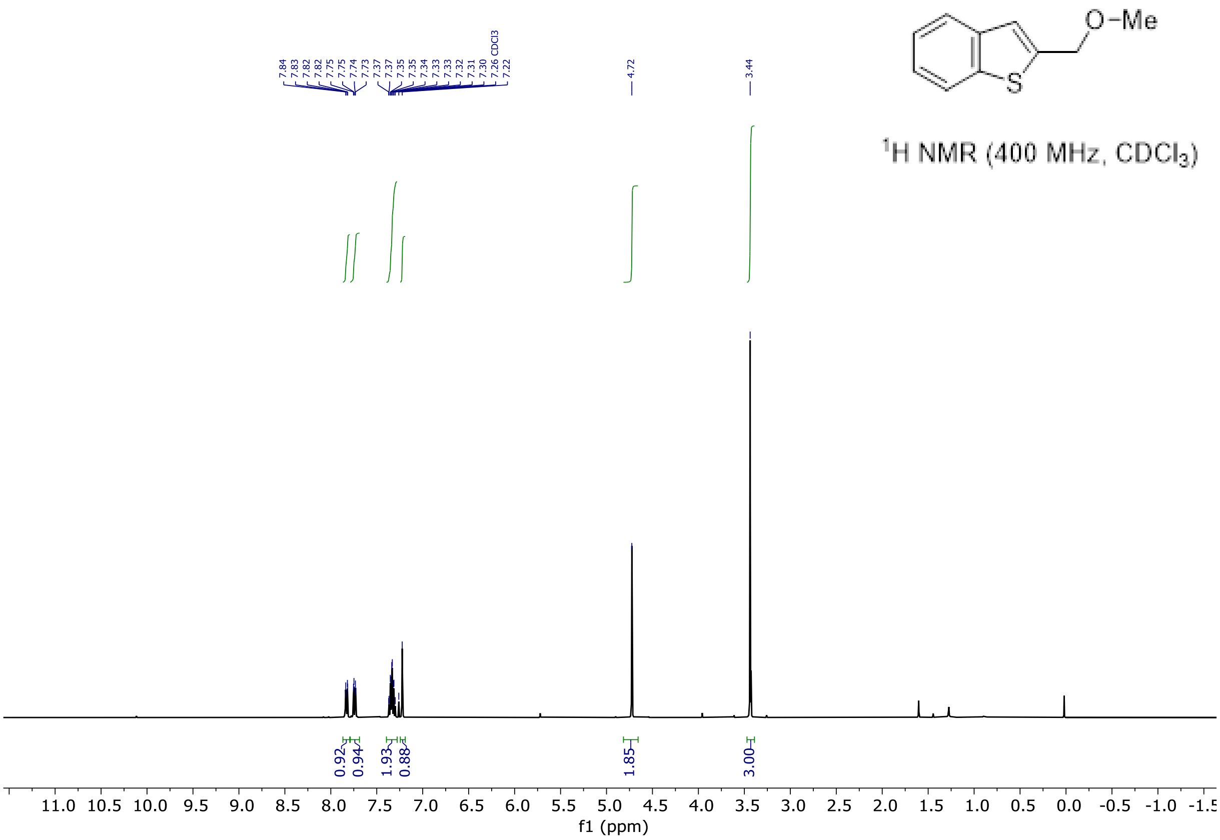

NMR spectrum of compound **20**

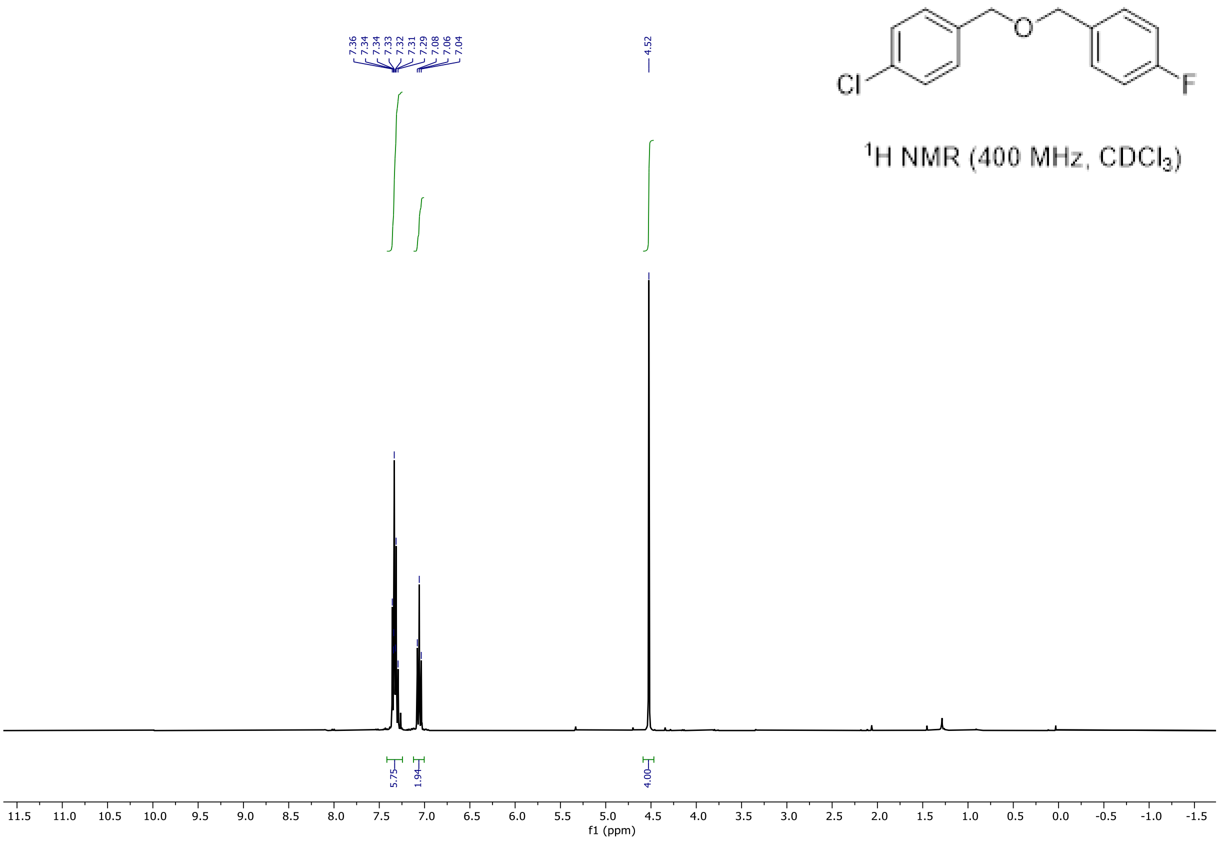

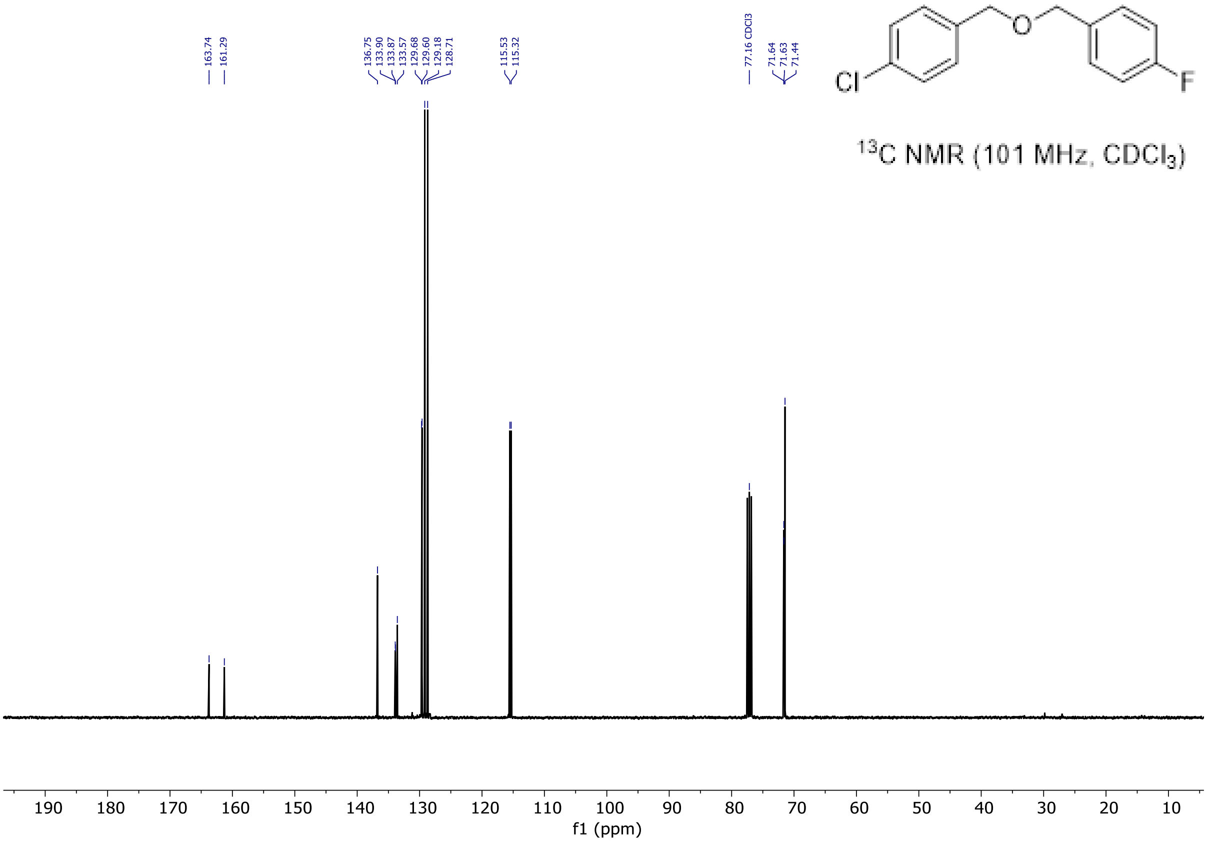

NMR spectrum of compound **21**

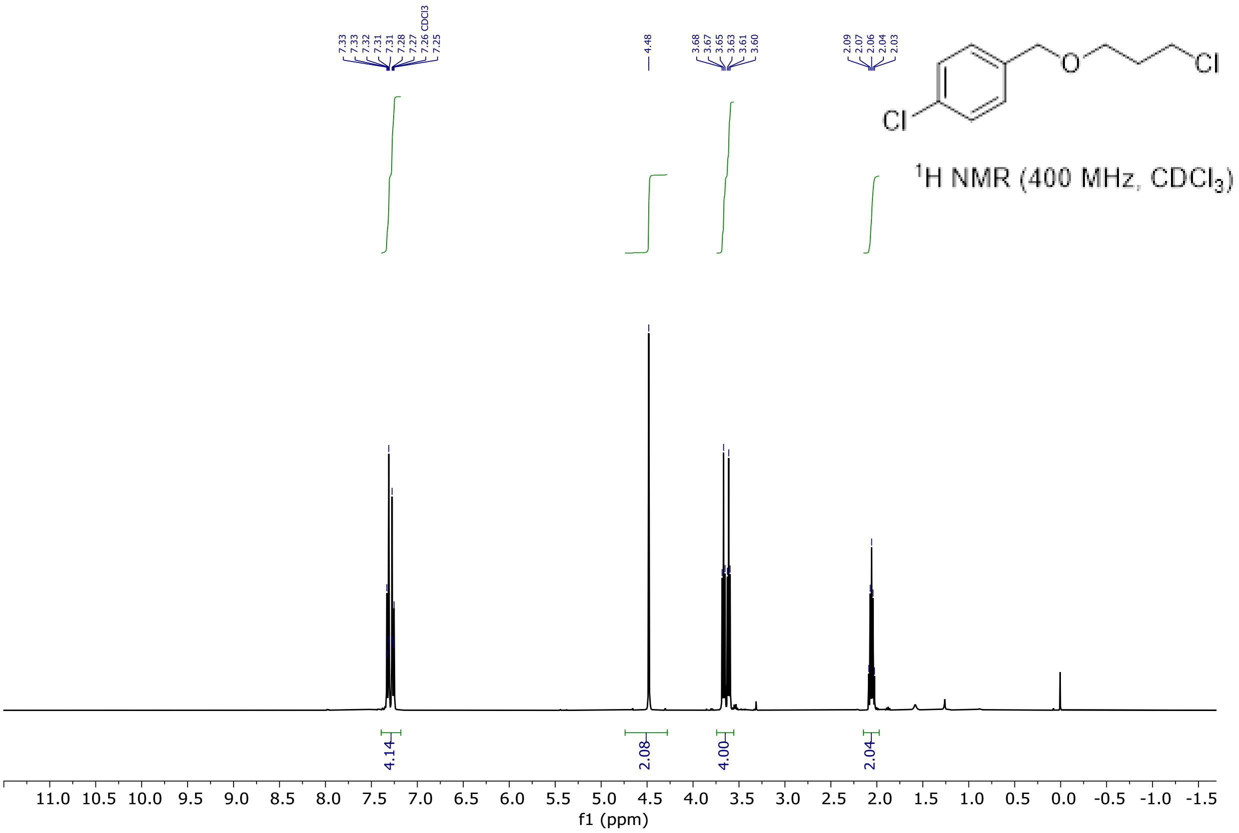

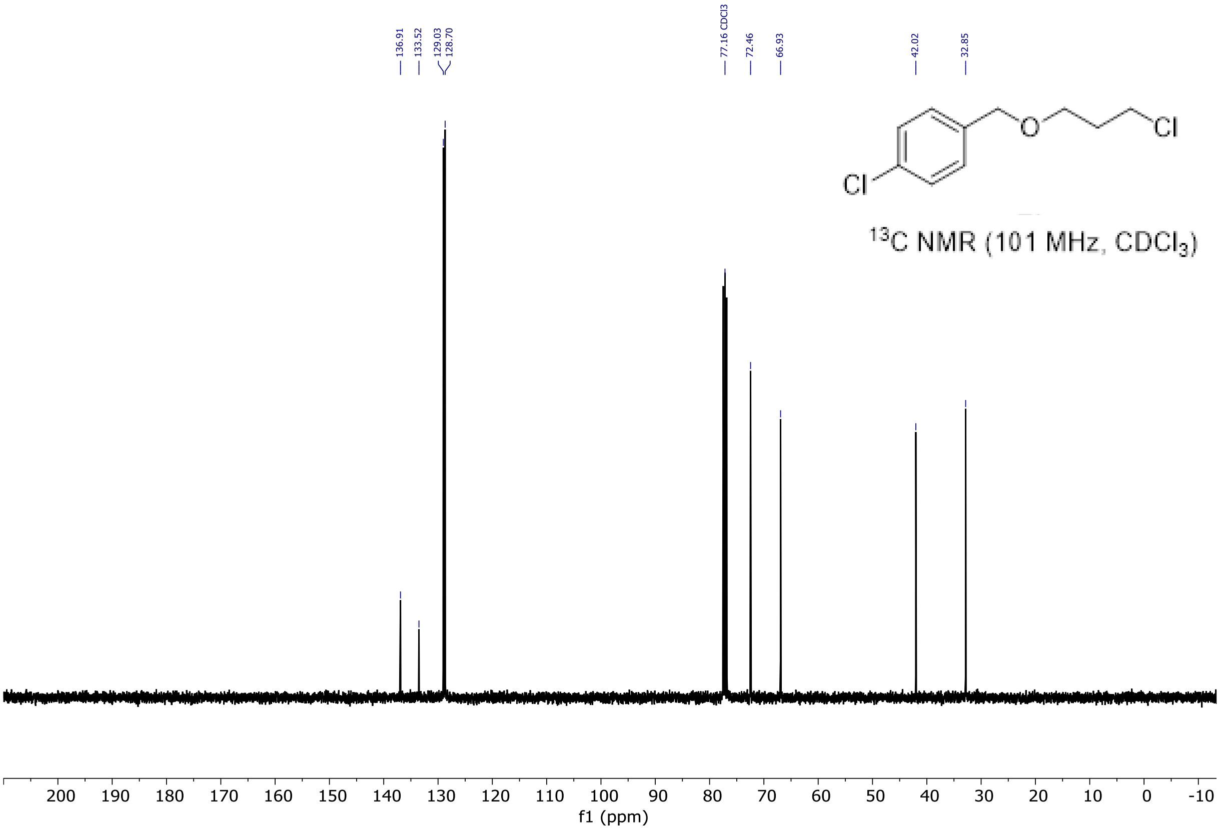

^{1}H NMR spectrum of compound **22**

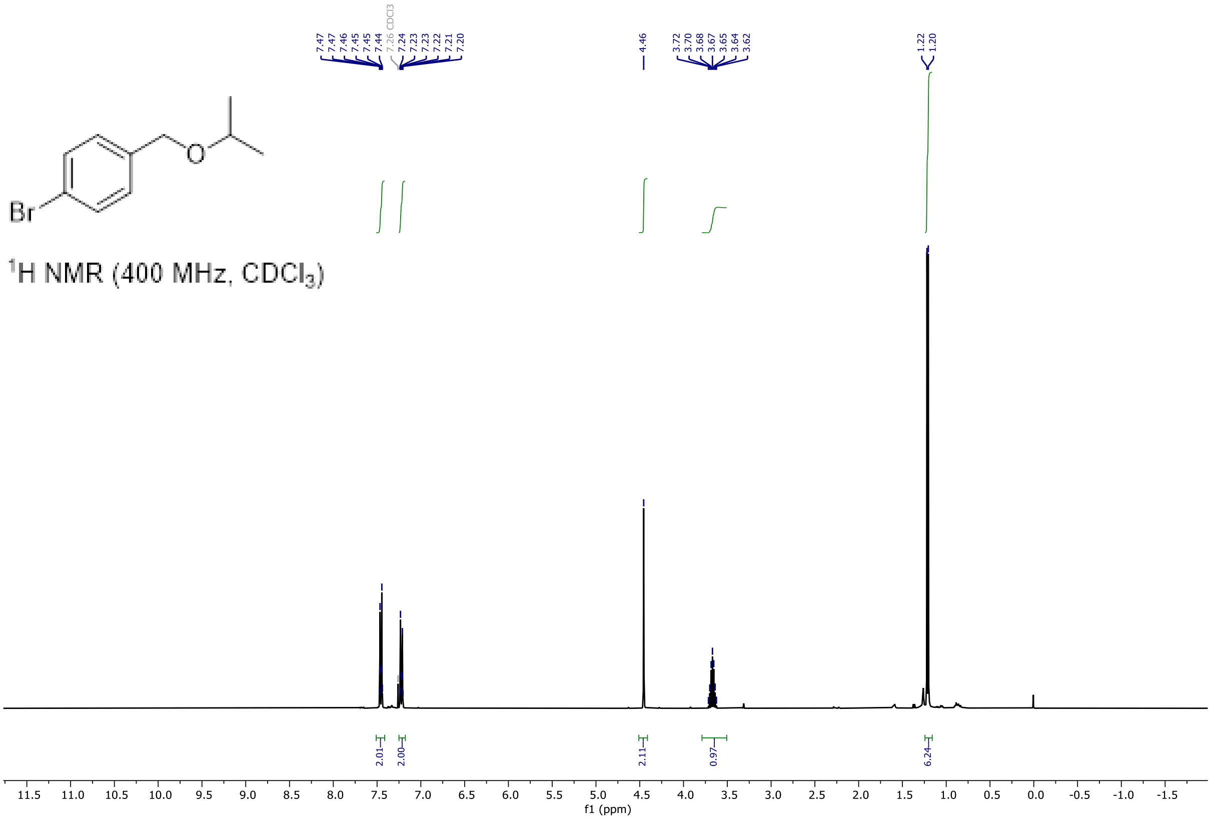

^{1}H NMR spectrum of compound **23**

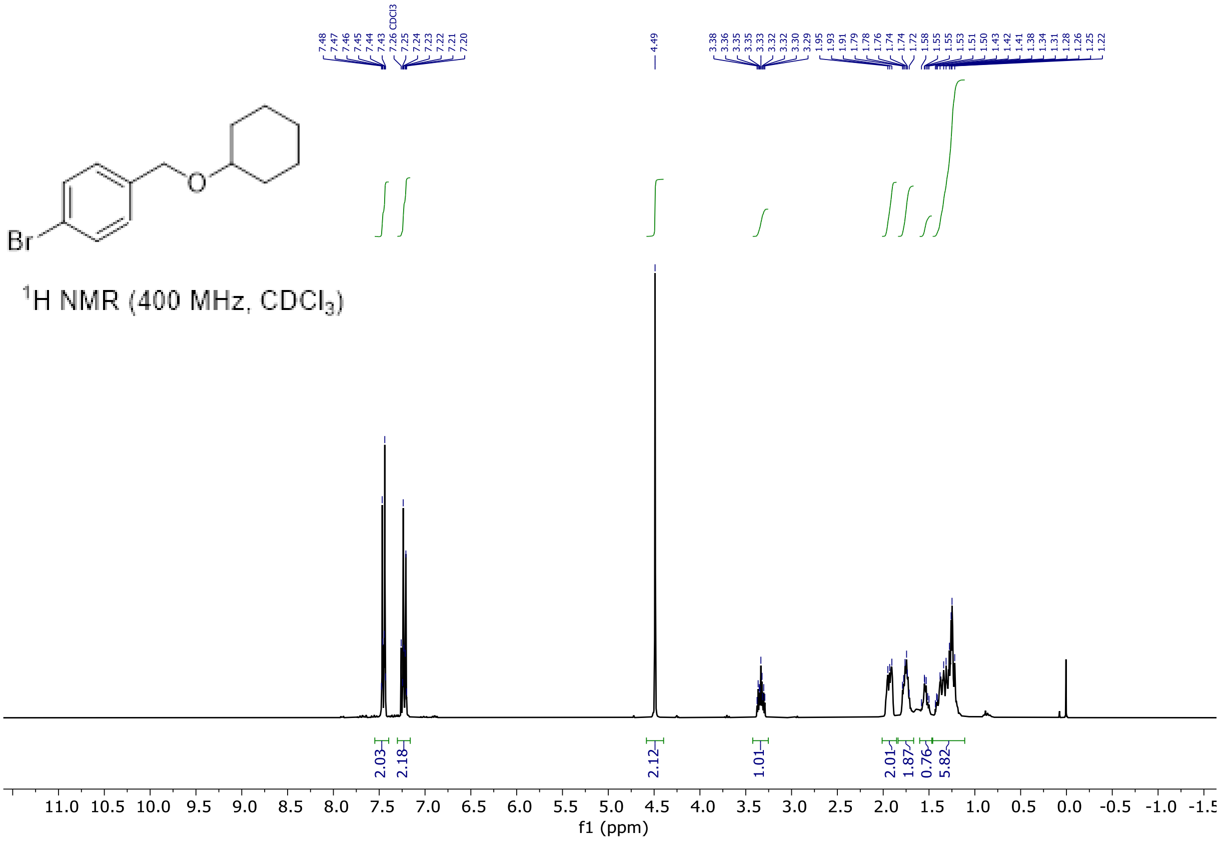

NMR spectra of compound **24**

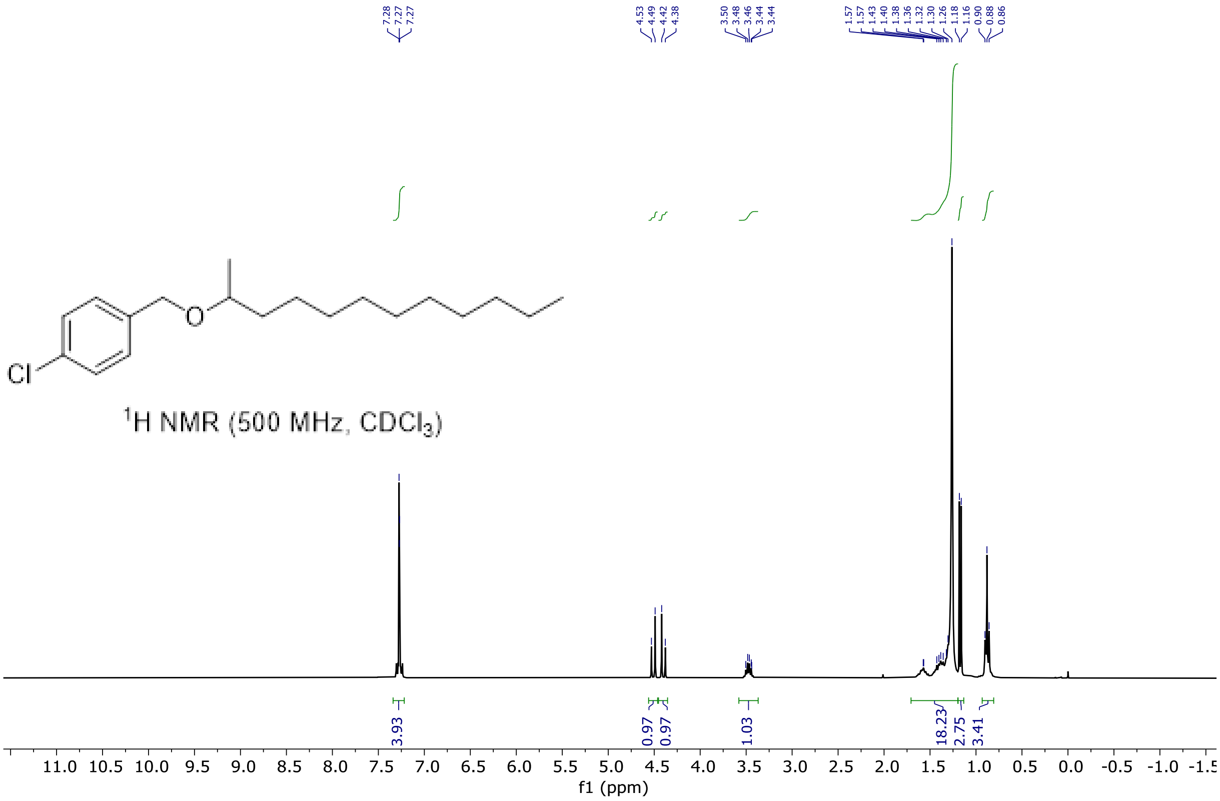

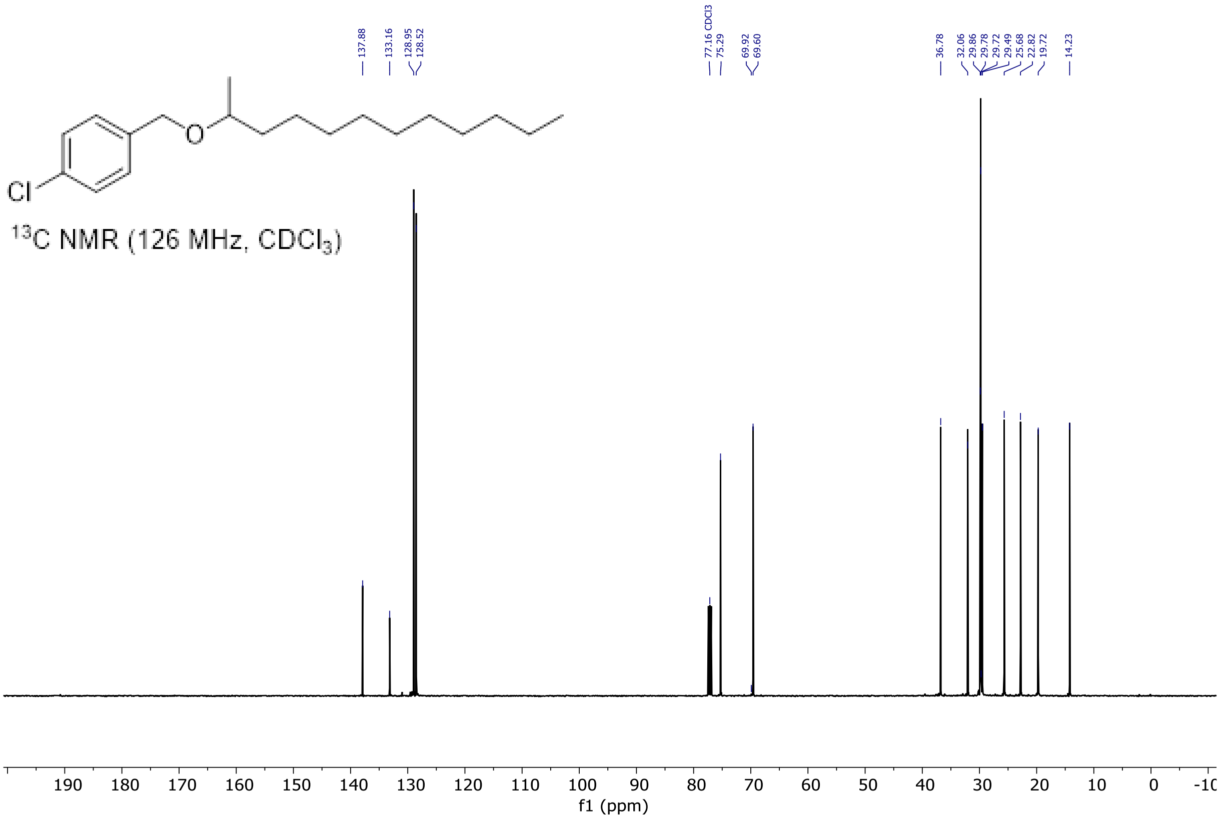

NMR spectra of compound **25**

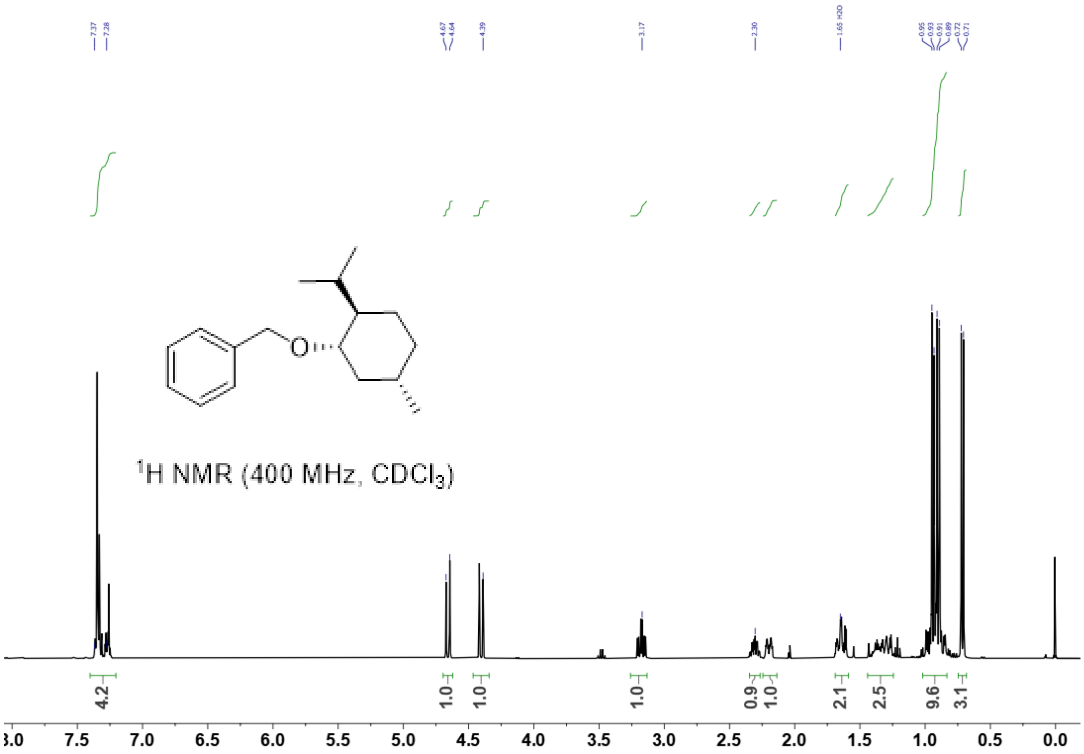

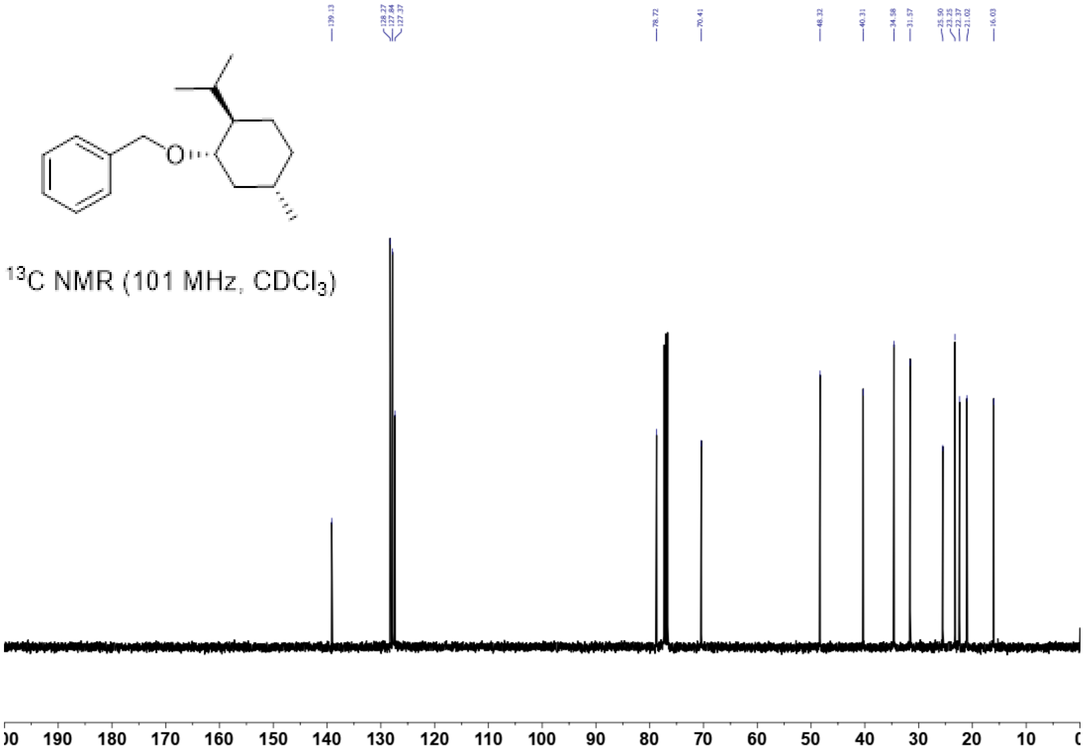

Synthesis of compound **26**

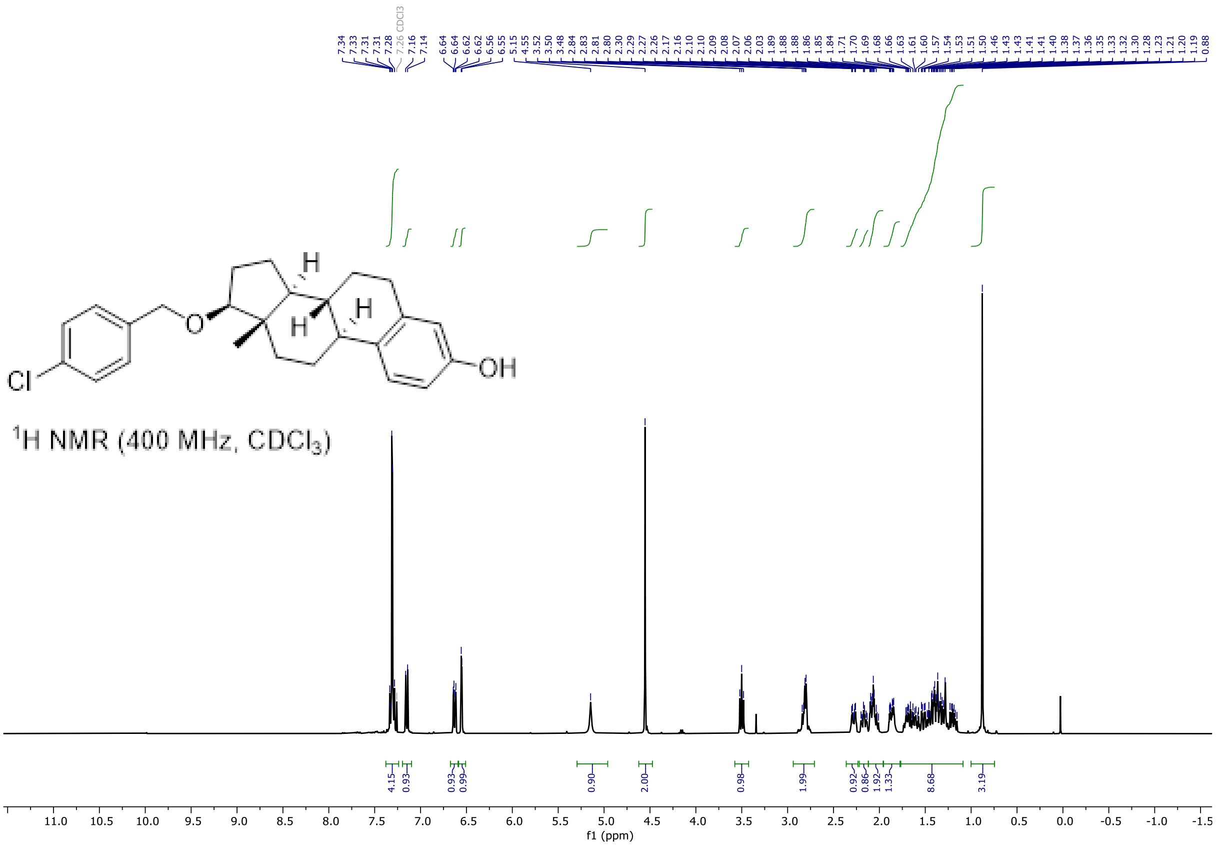

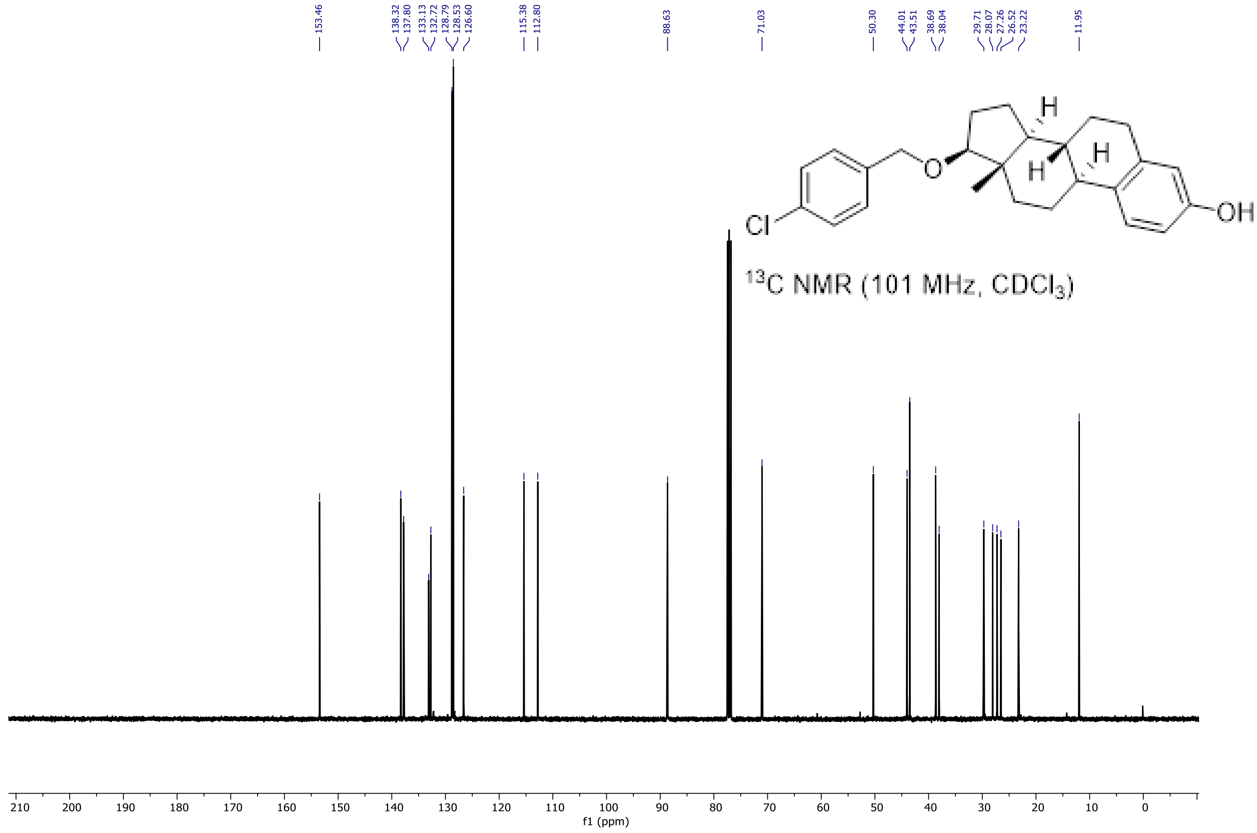

NMR spectra of compound **27**

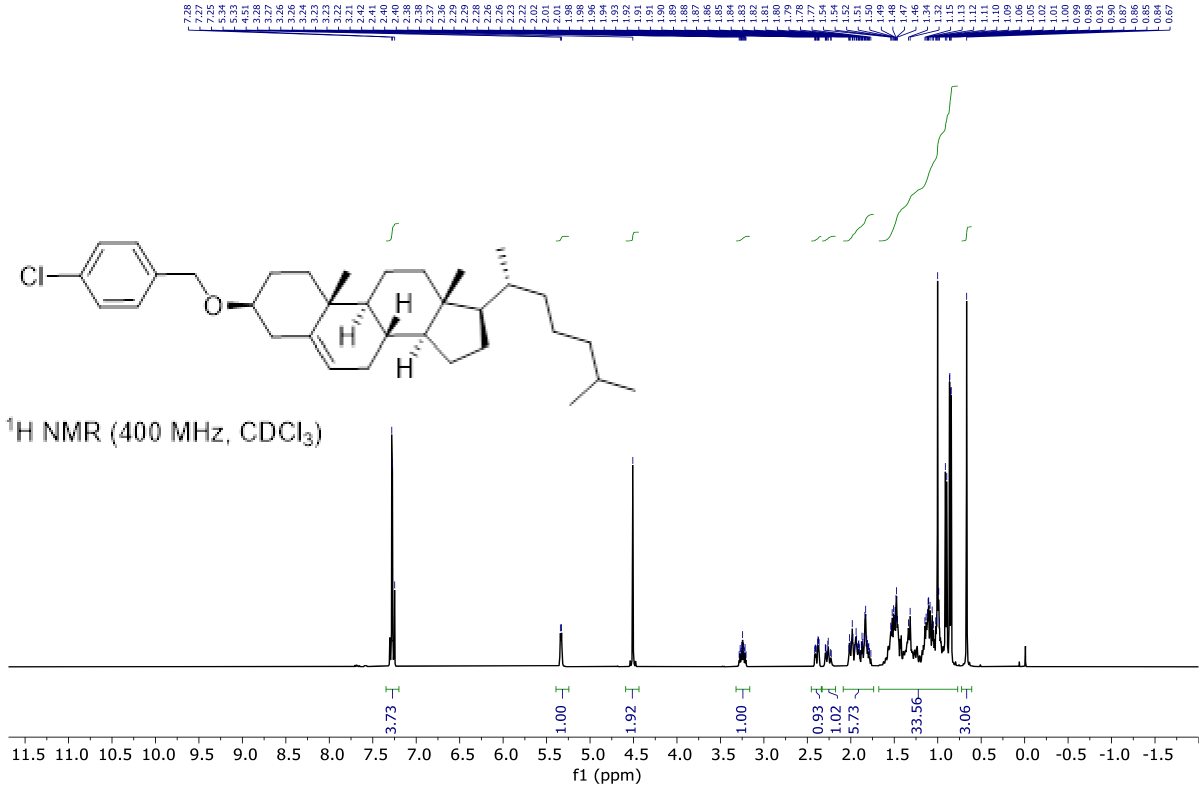

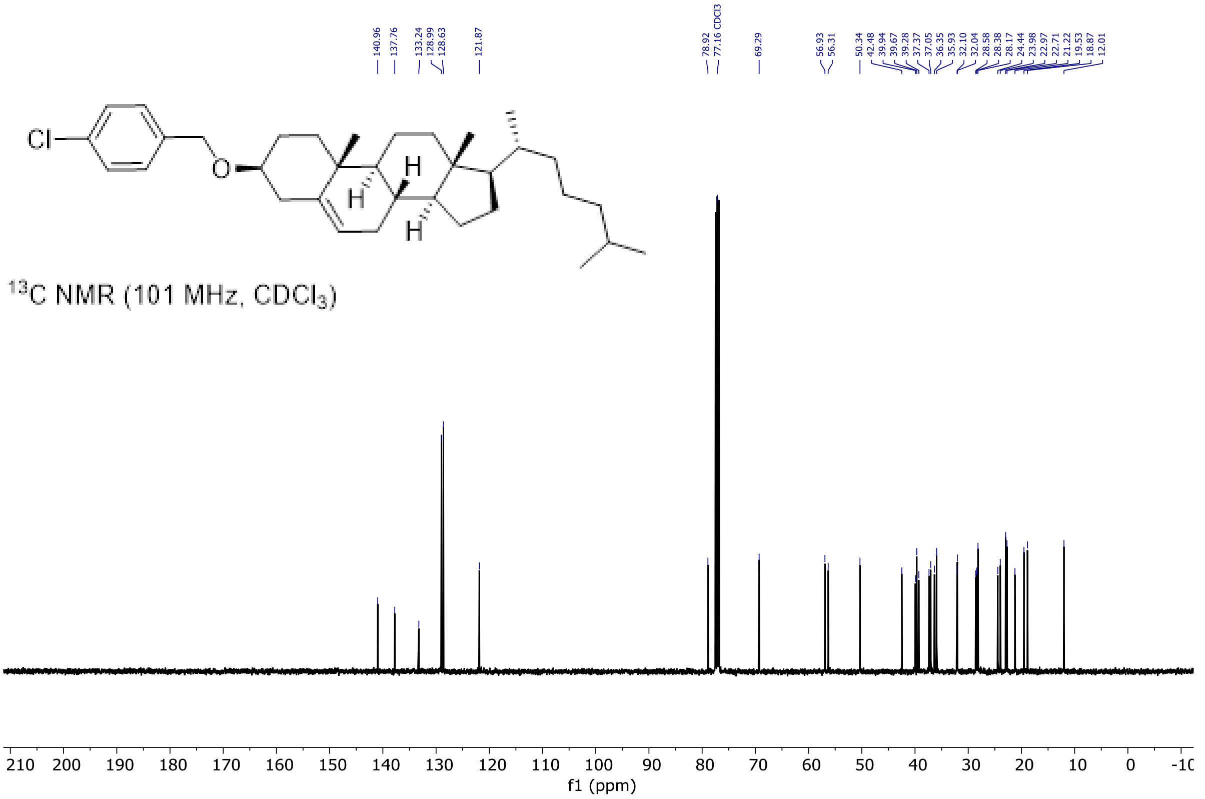

^{1}H NMR spectrum of compound **13g.**

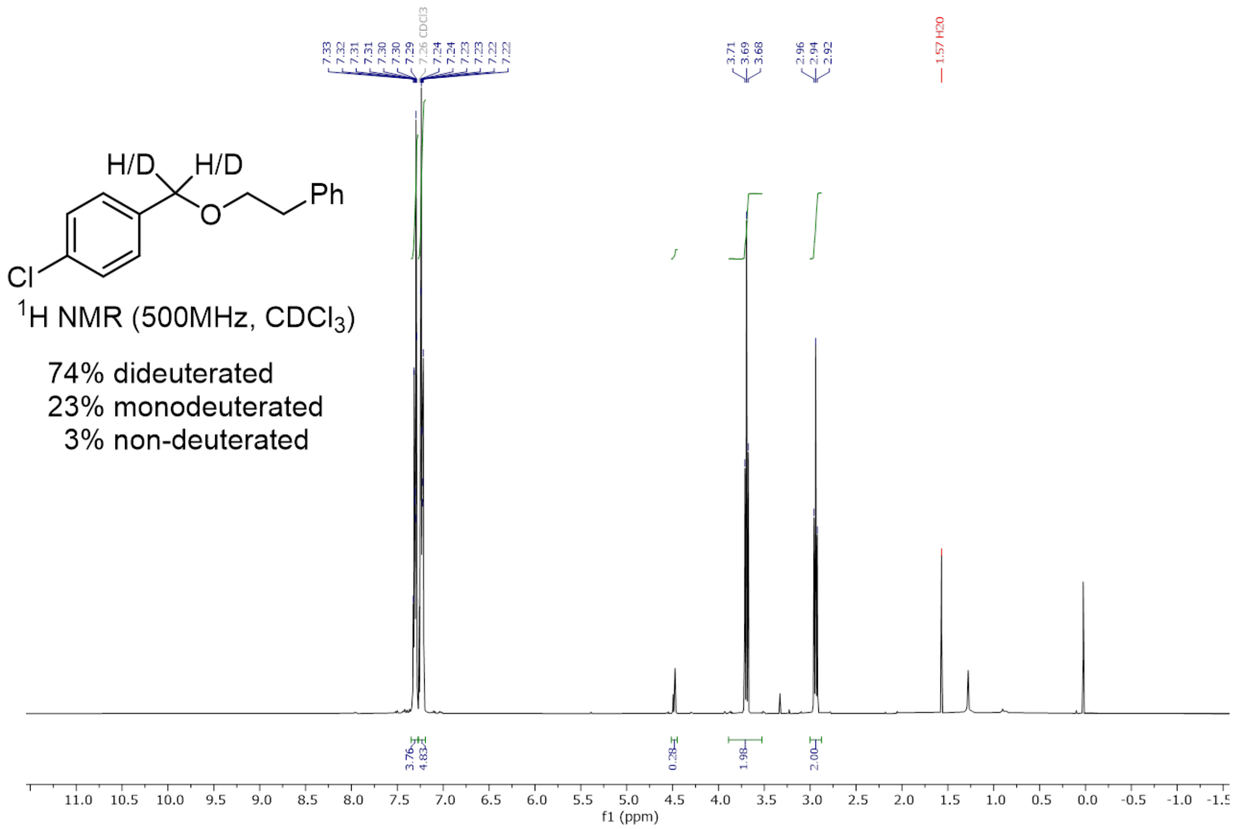

^{2}H NMR spectrum of compound **13g.**

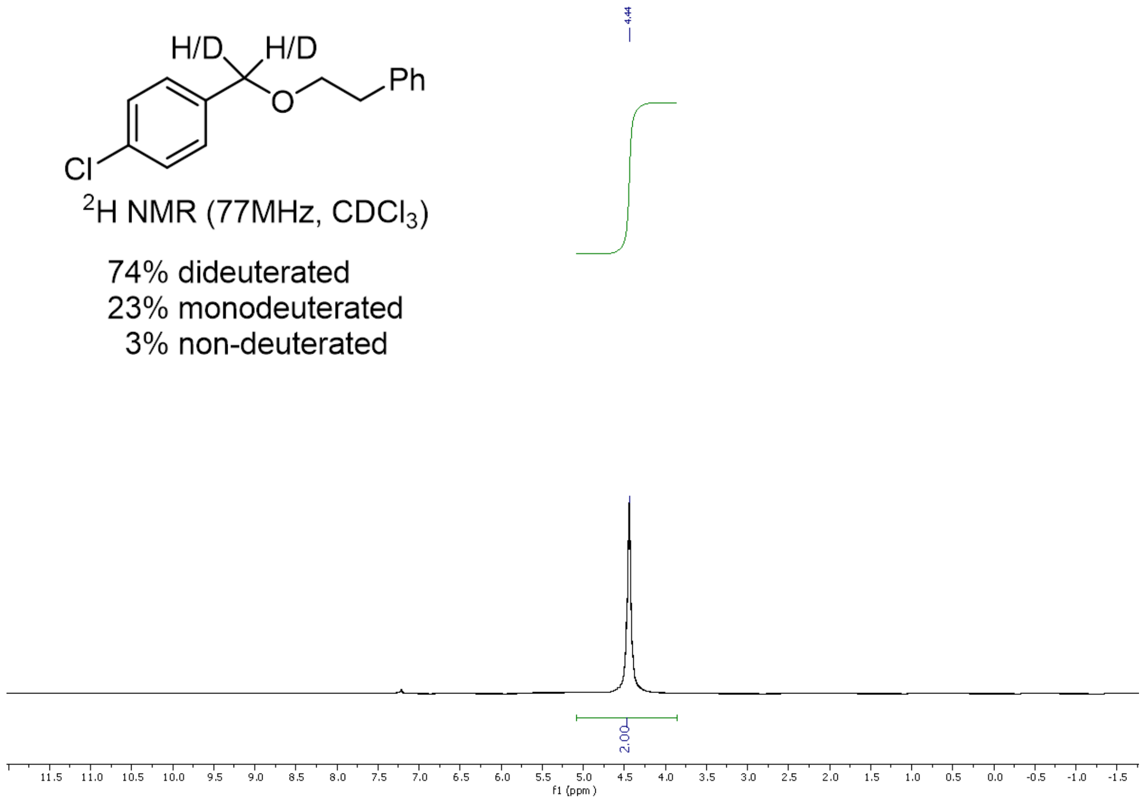

^{13}C NMR spectrum of compound **13g.**

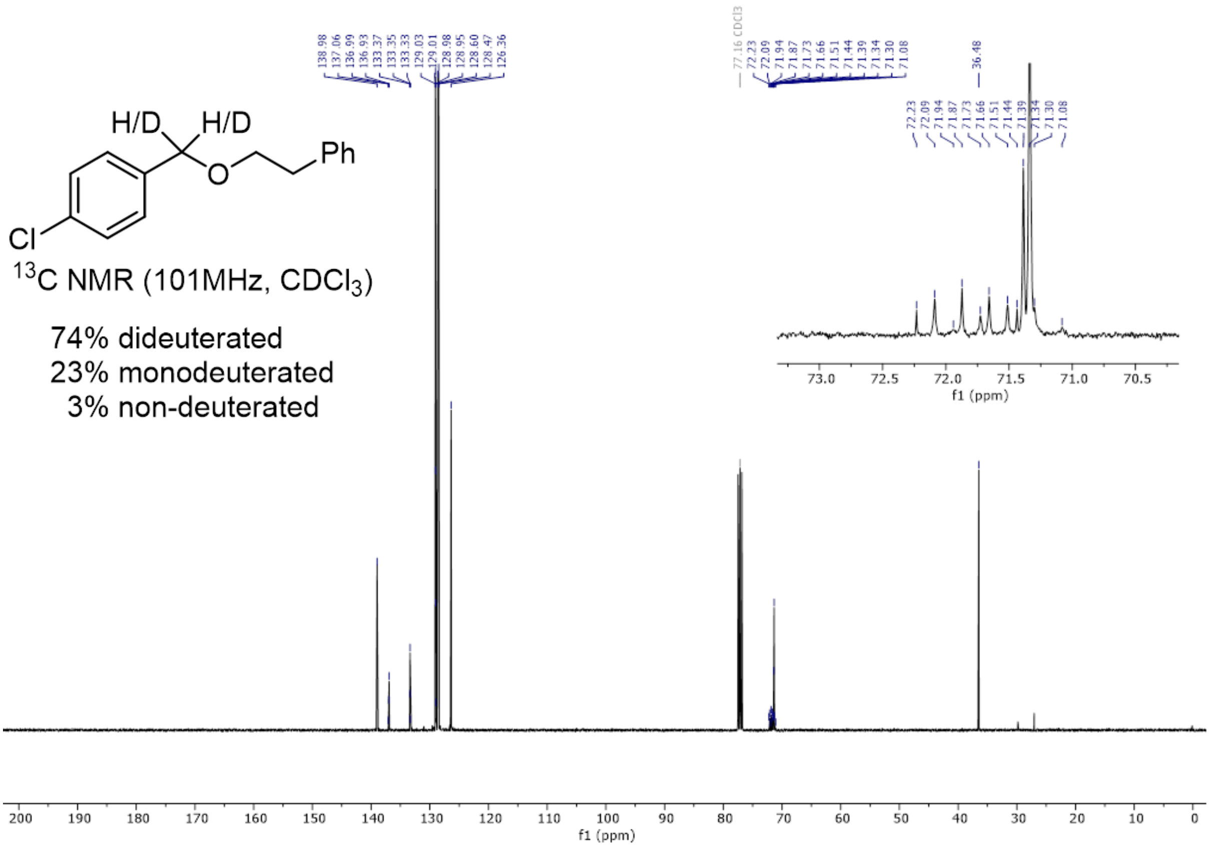

^{1}H NMR spectrum of compound **13h.**

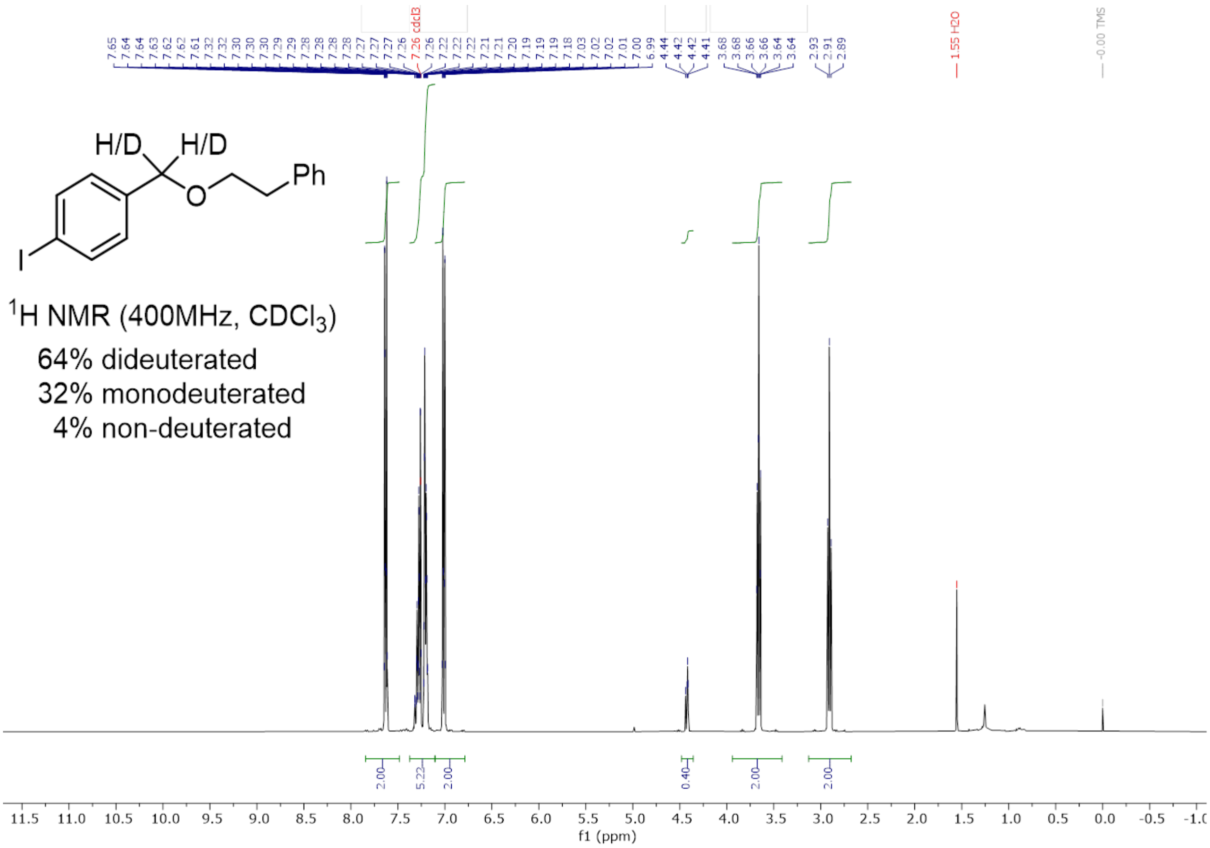

^{2}H NMR spectrum of compound **13h.**

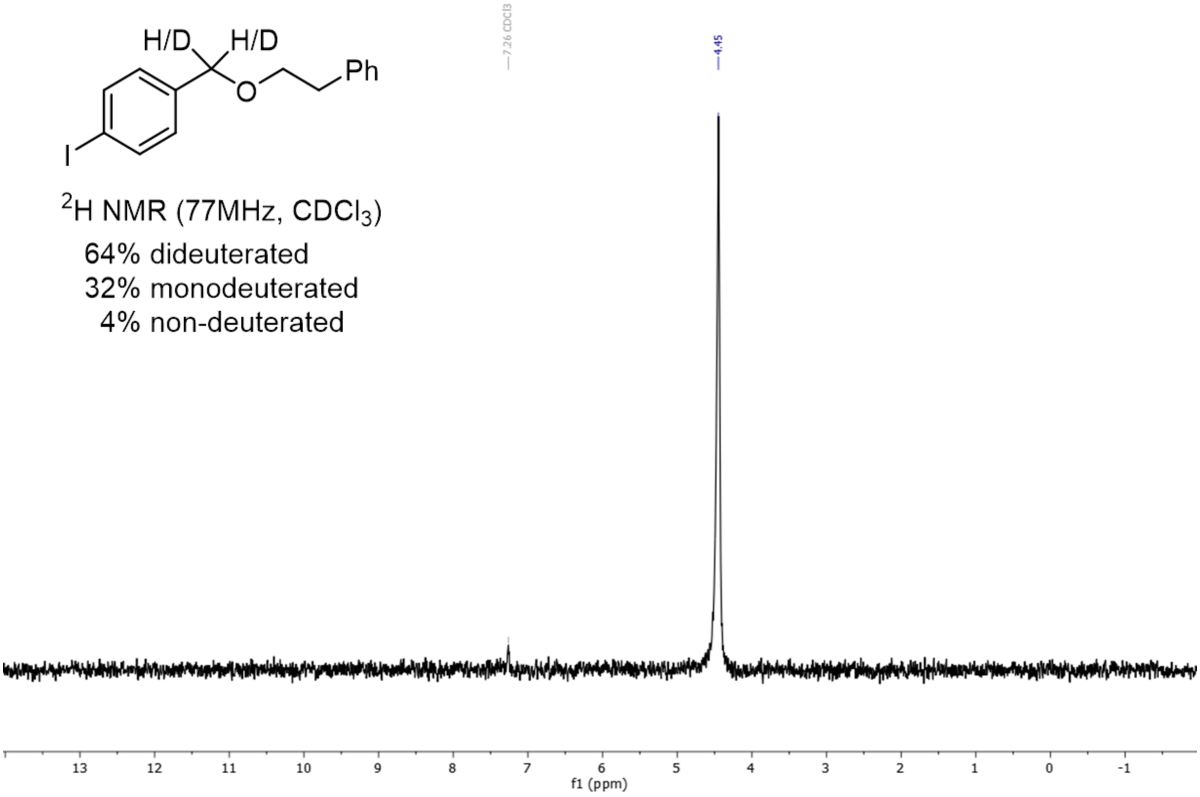

^{13}C NMR spectrum of compound **13h.**

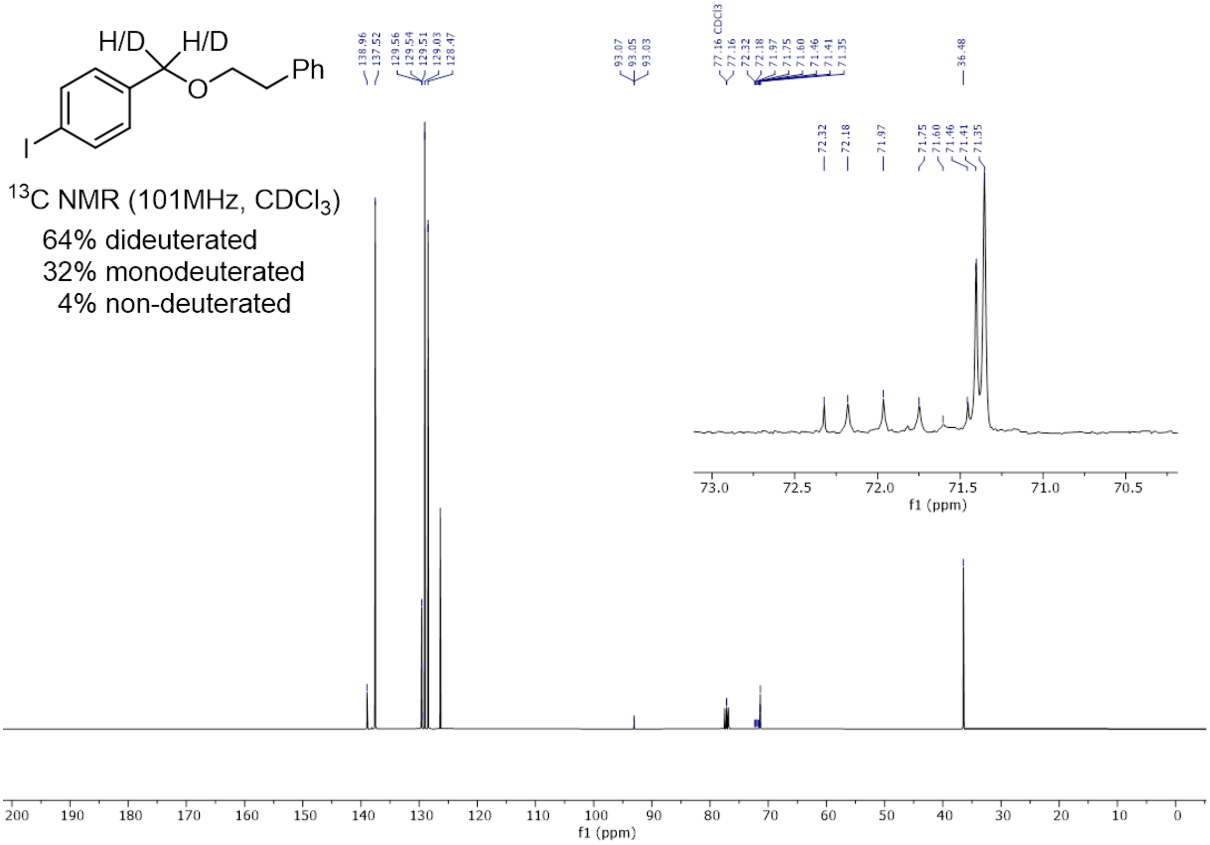

^{1}H NMR spectrum of compound **14n.**

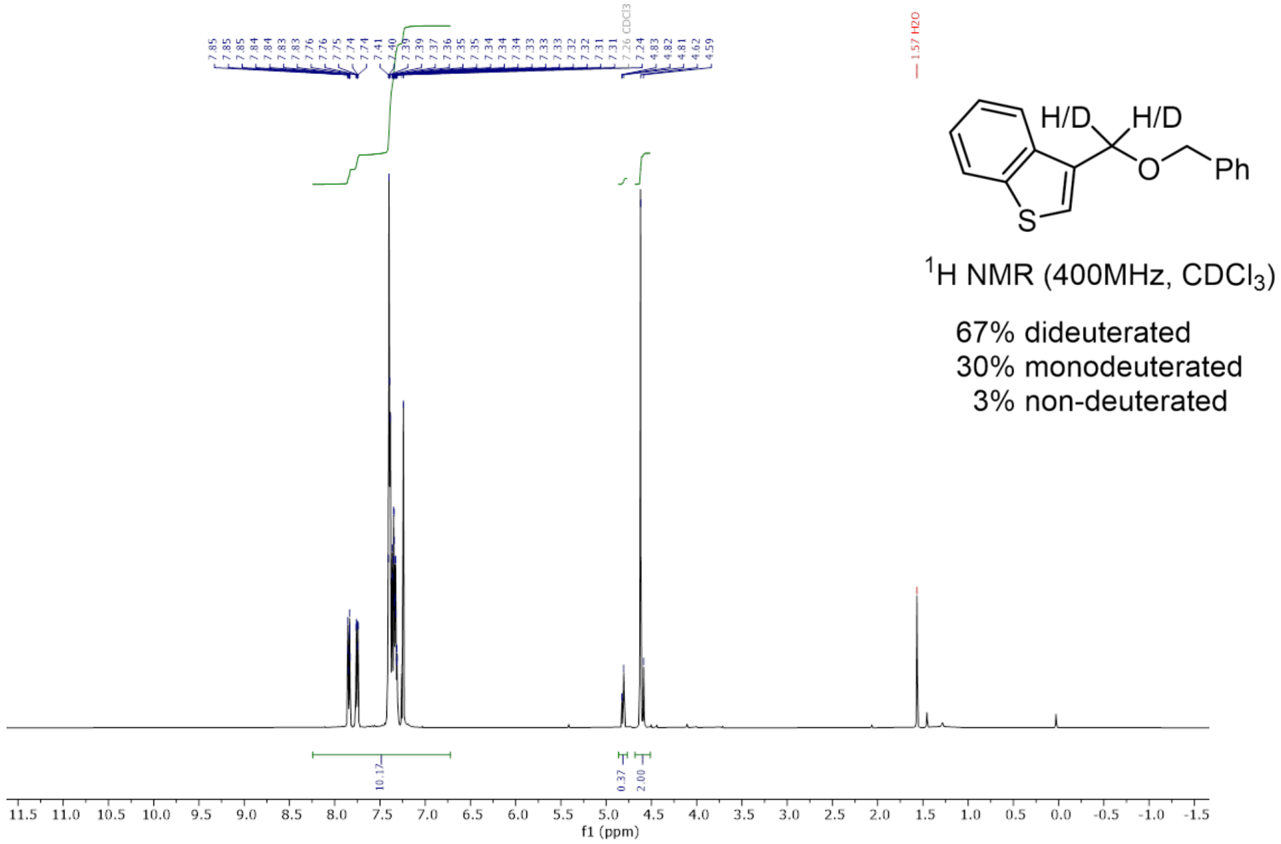

^{2}H NMR spectrum of compound **14n.**

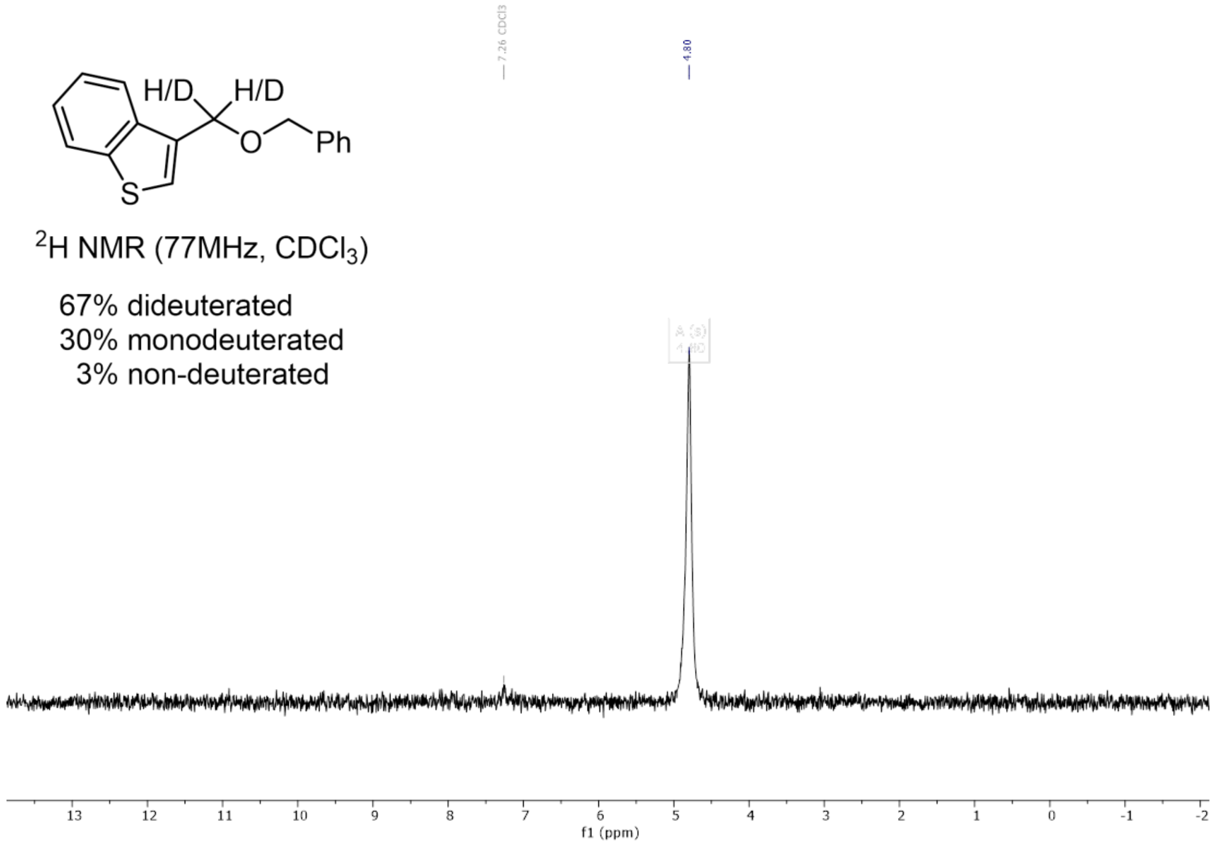

^{13}C NMR spectrum of compound **14n.**

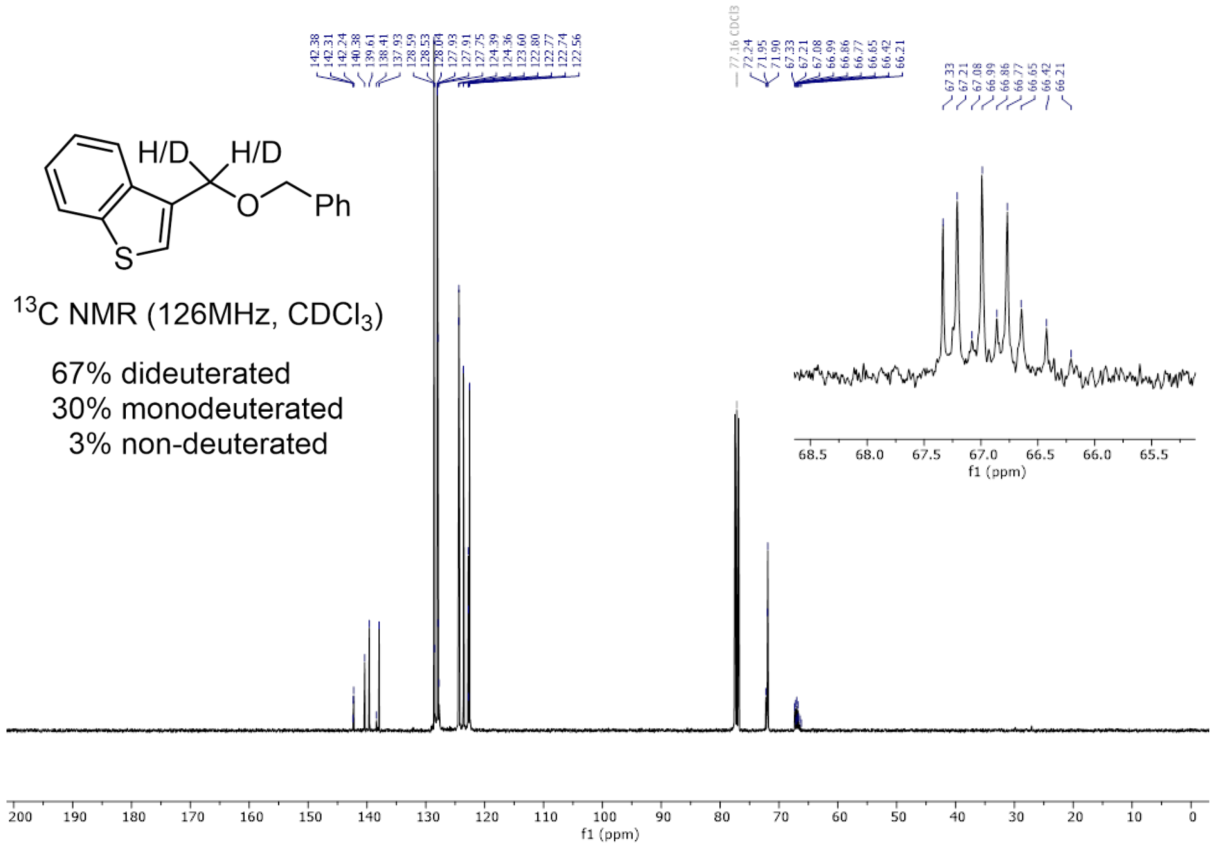

^{1}H NMR spectrum of compound **13i**

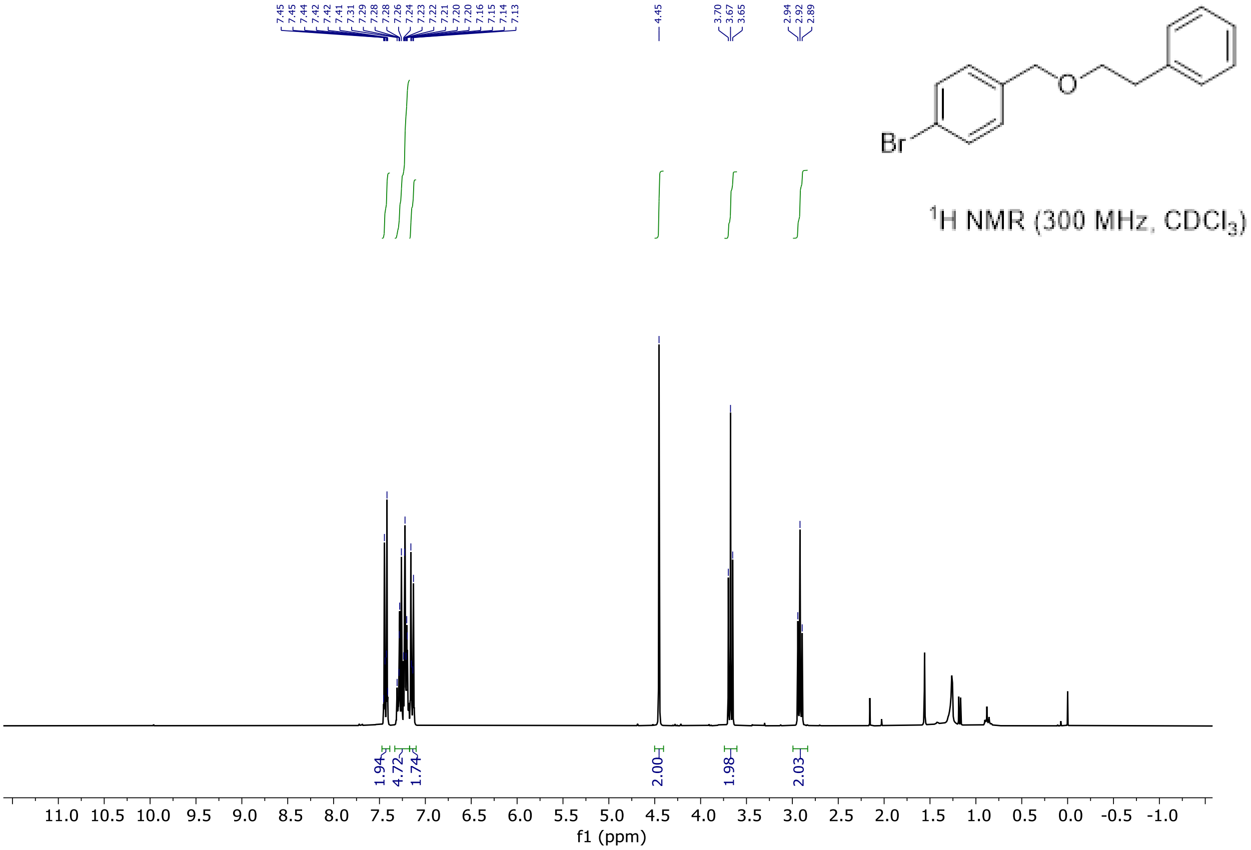

^{1}H NMR spectrum for compound **14o**

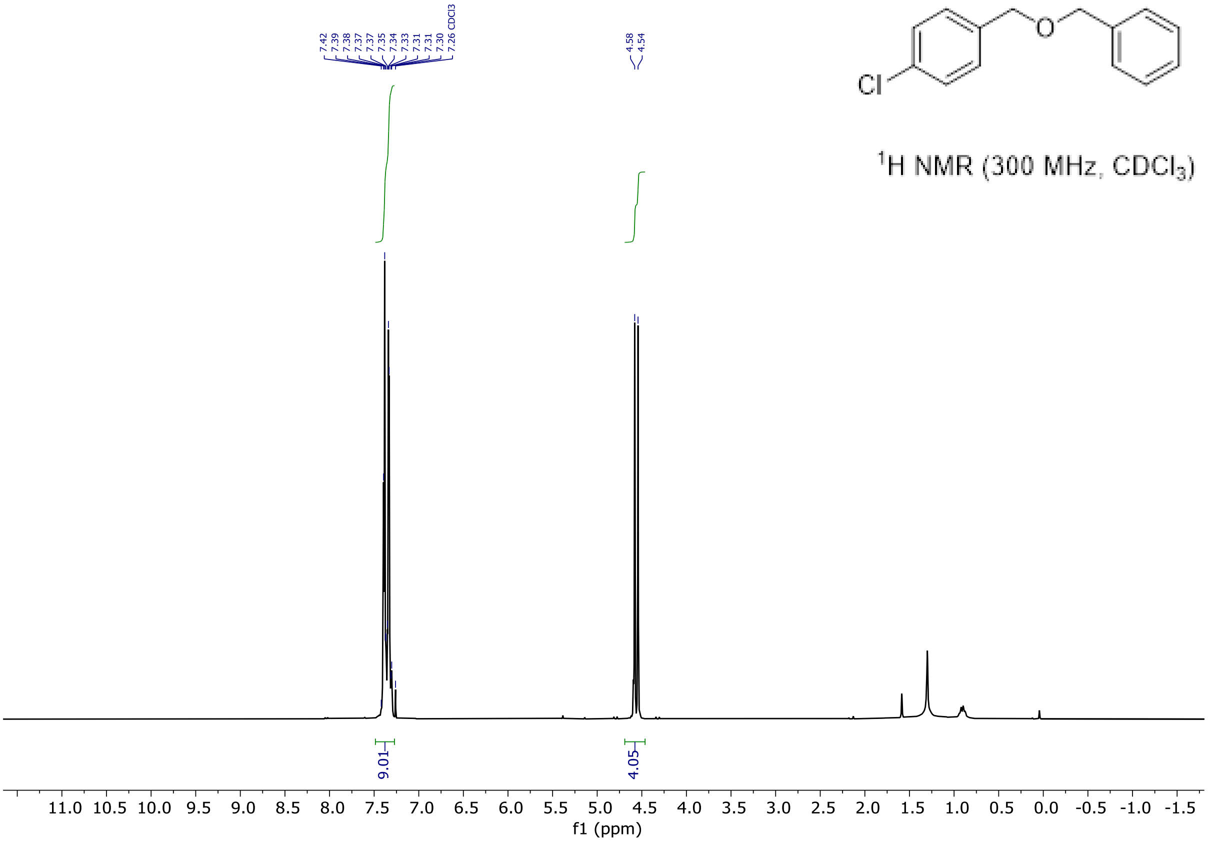

^{1}H NMR spectrum from compound **31**

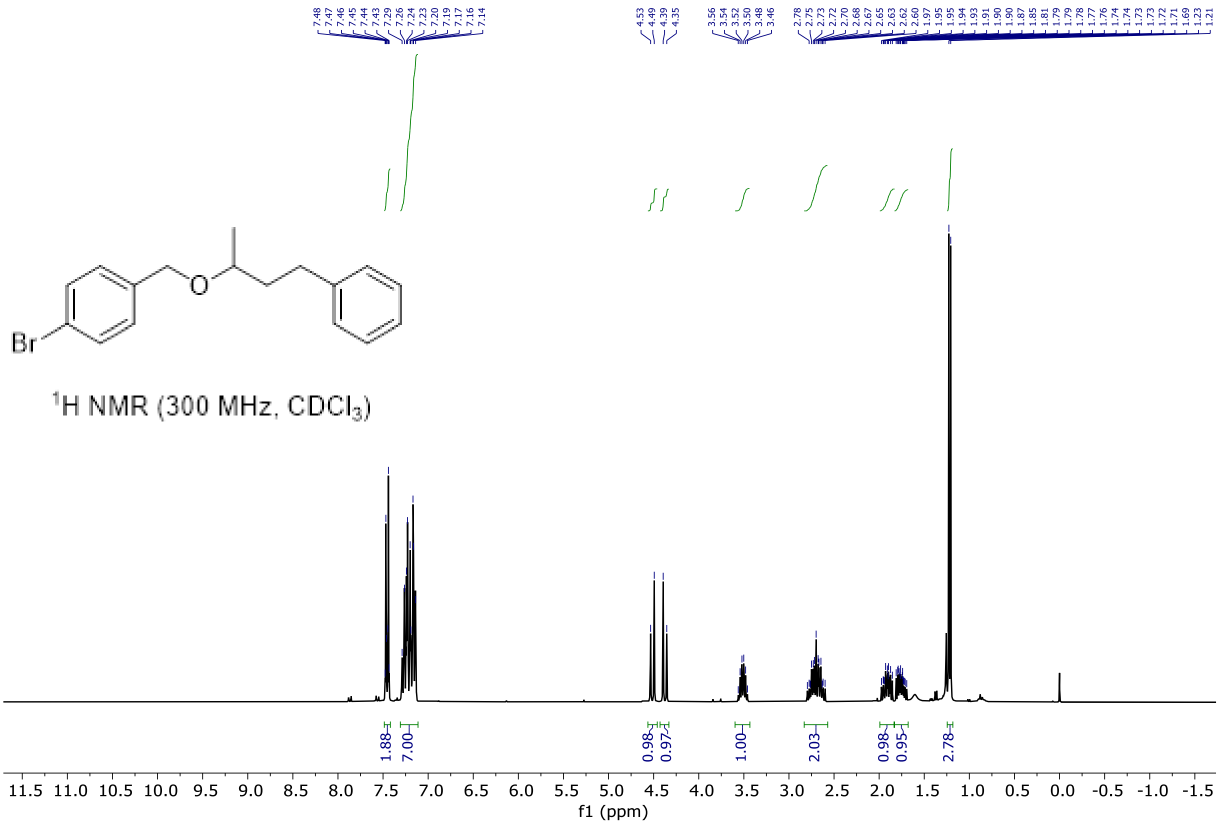

^{1}H NMR spectrum of compound **13j.**

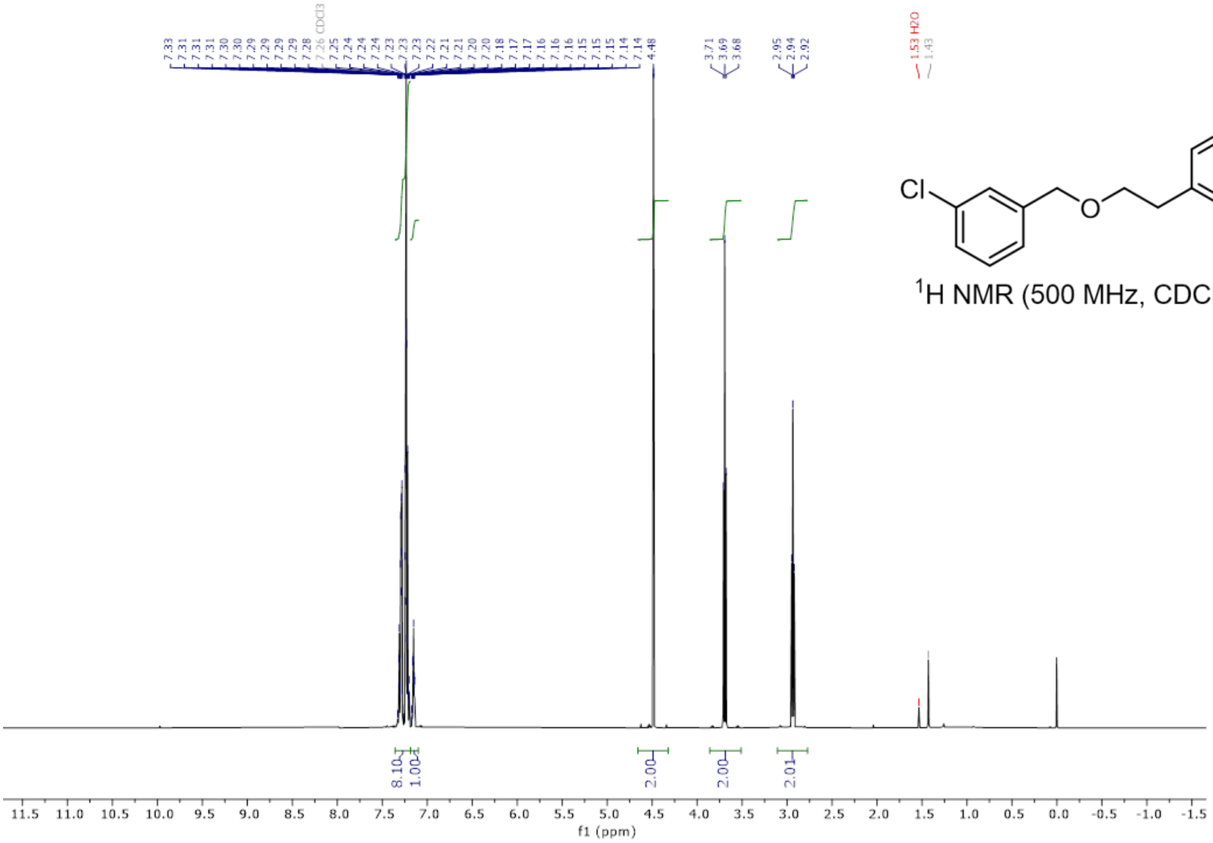

^{13}C NMR spectrum of compound **13j.**

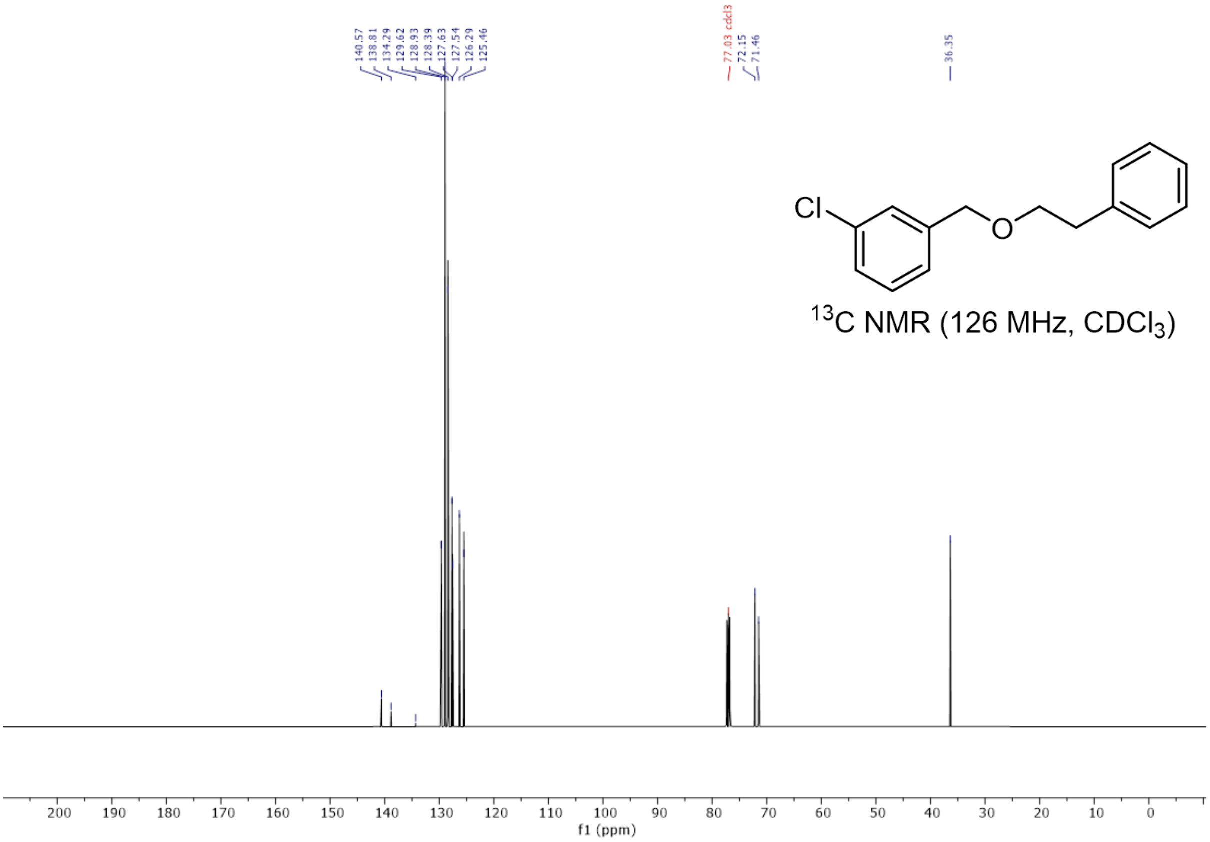

^1H NMR spectrum of compound **14p.**

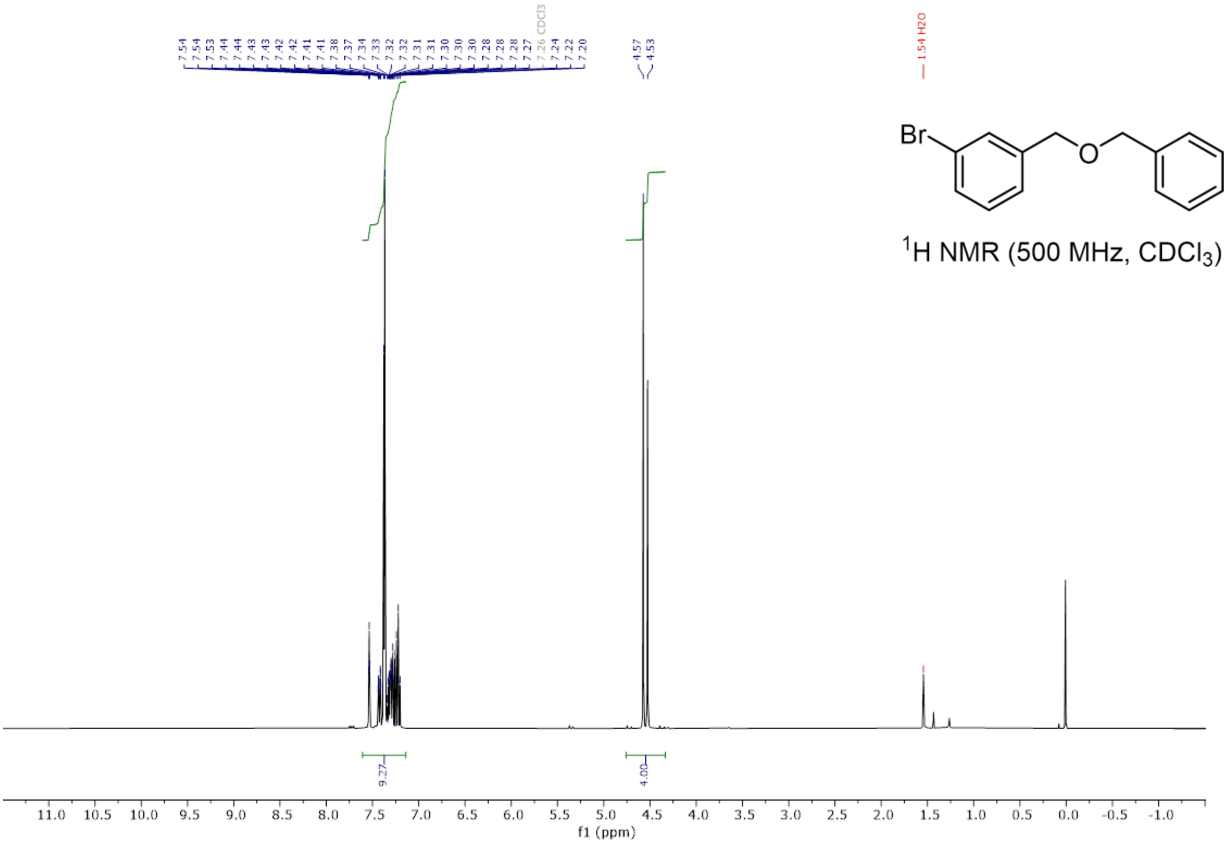

^{1}H NMR spectrum of compound **32**

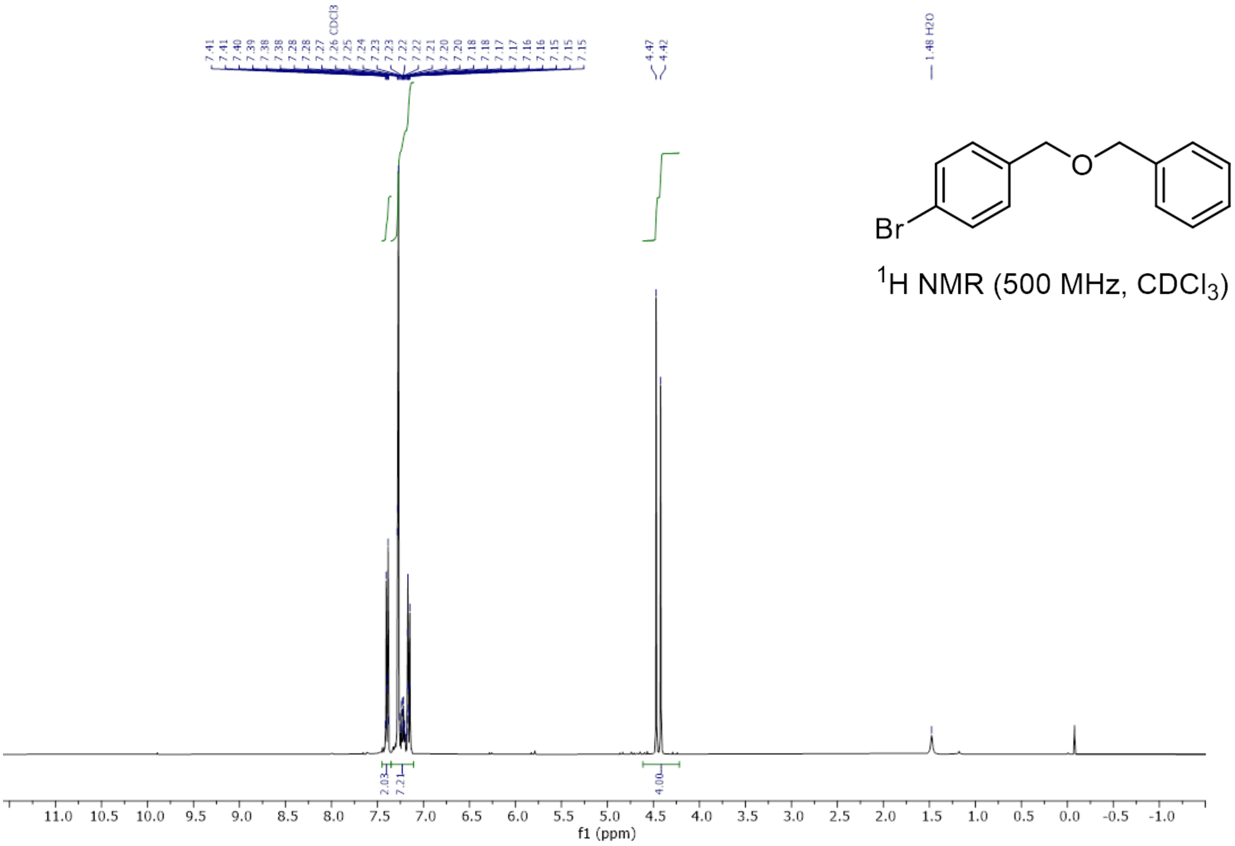

^{1}H NMR spectrum of compound **33.**

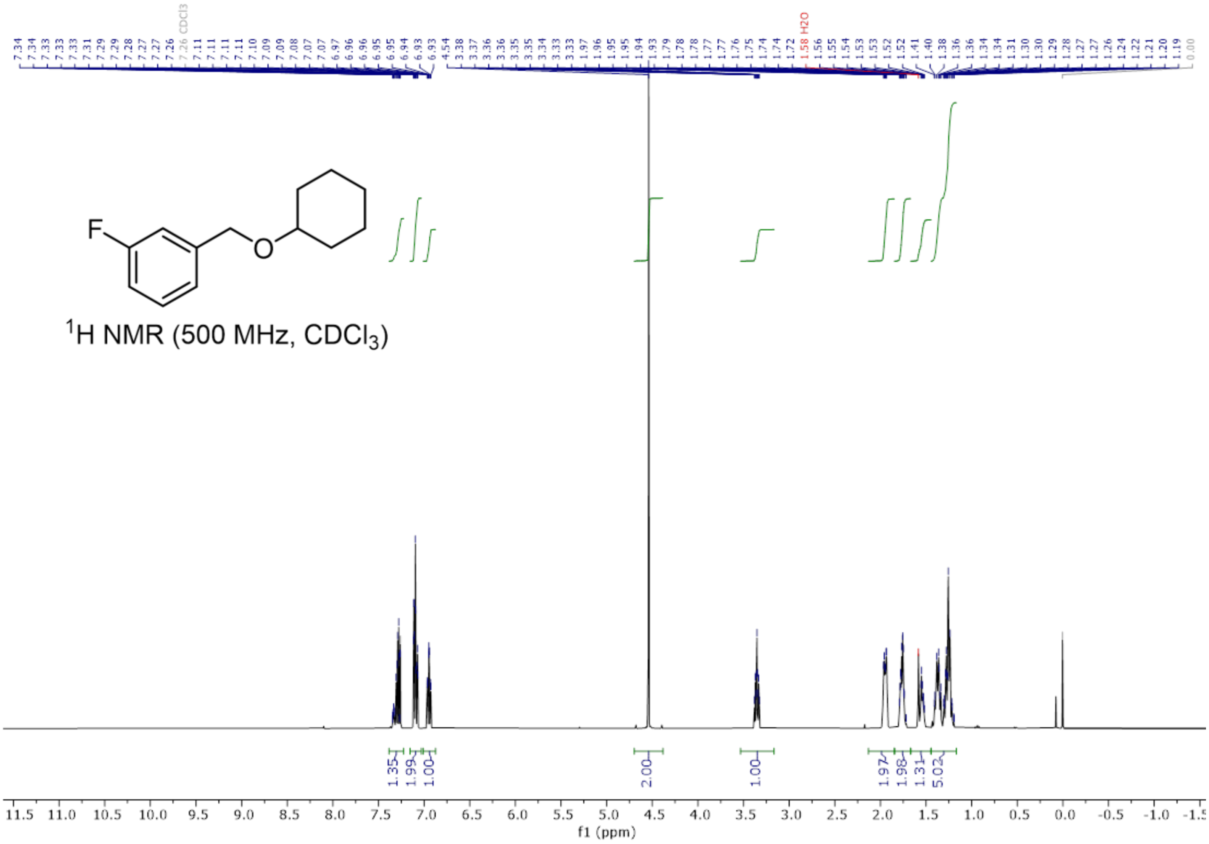

^{1}H NMR spectrum of compound **34.**

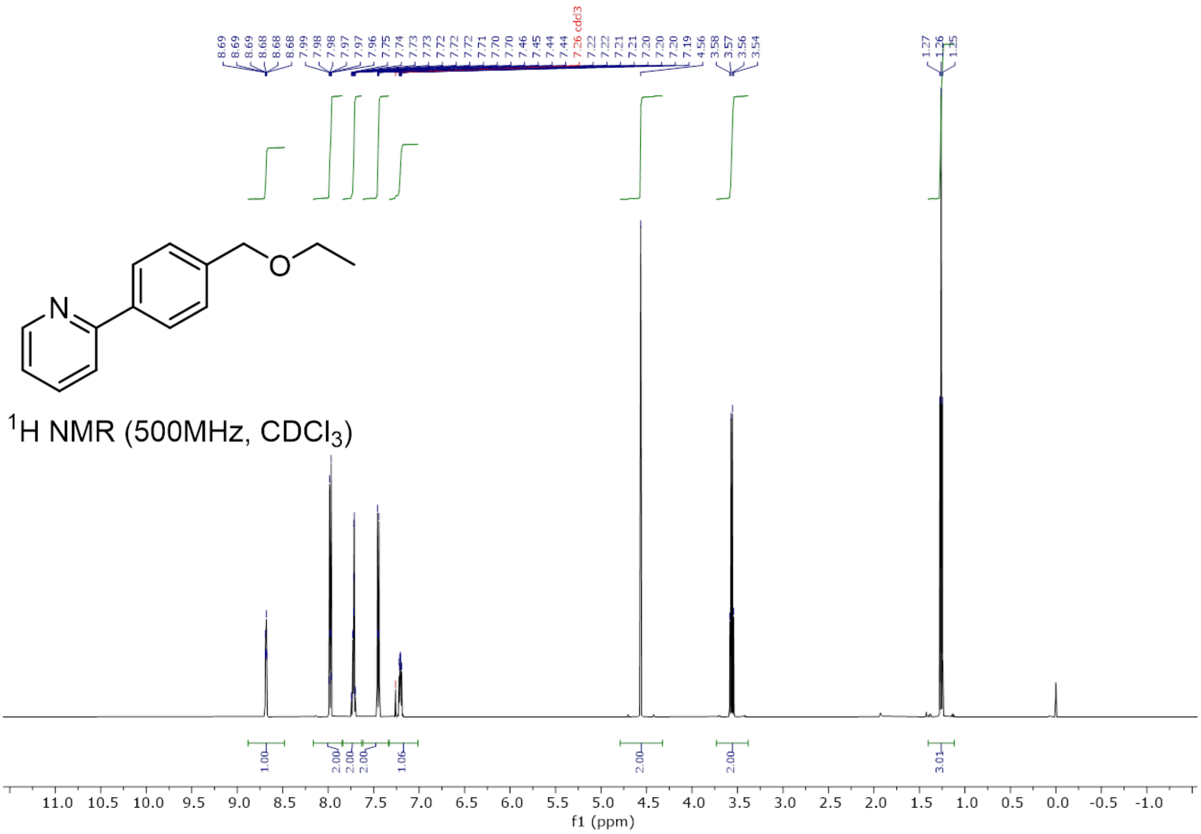

^{13}C NMR spectrum of compound **34.**

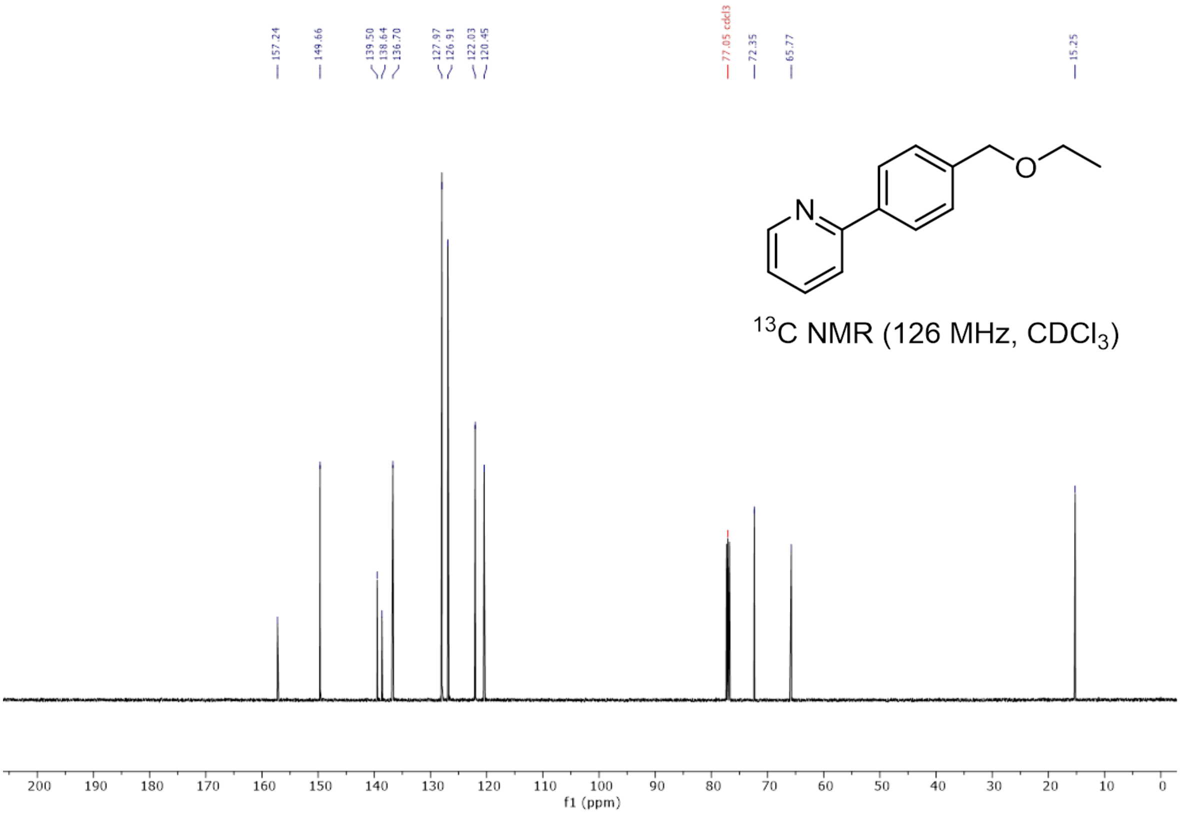

NMR spectra of compound **28**

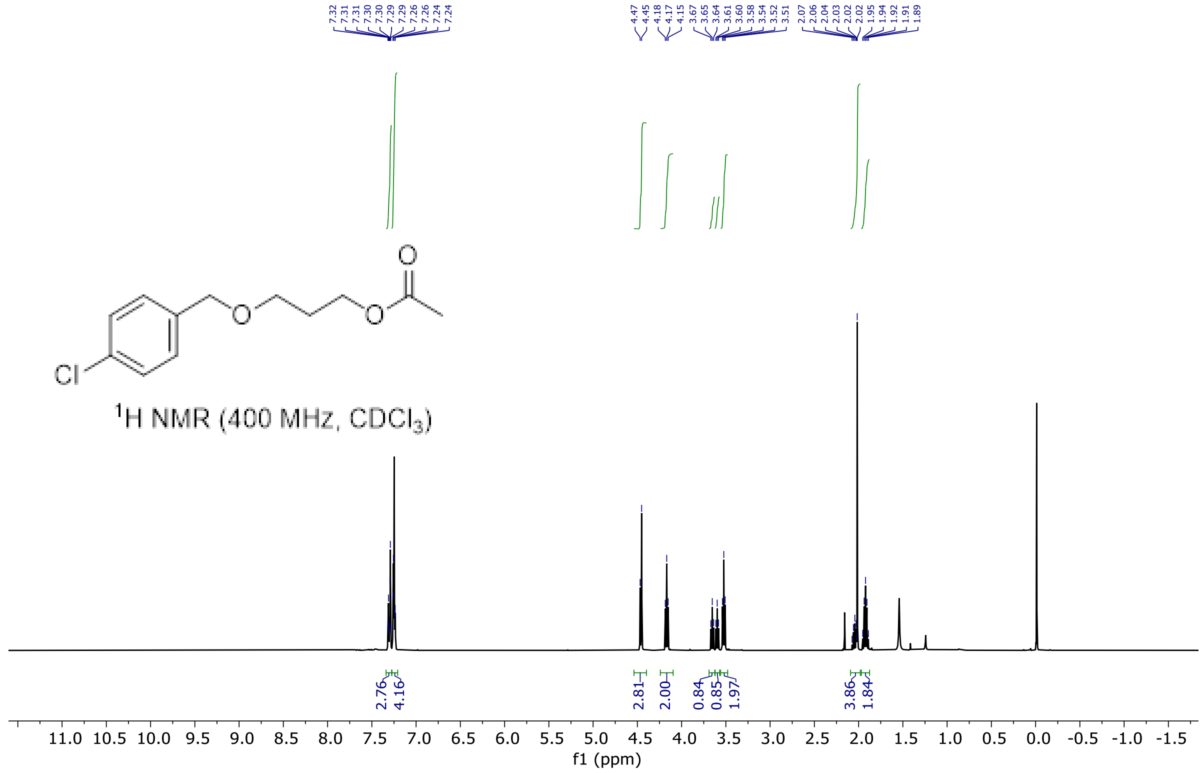

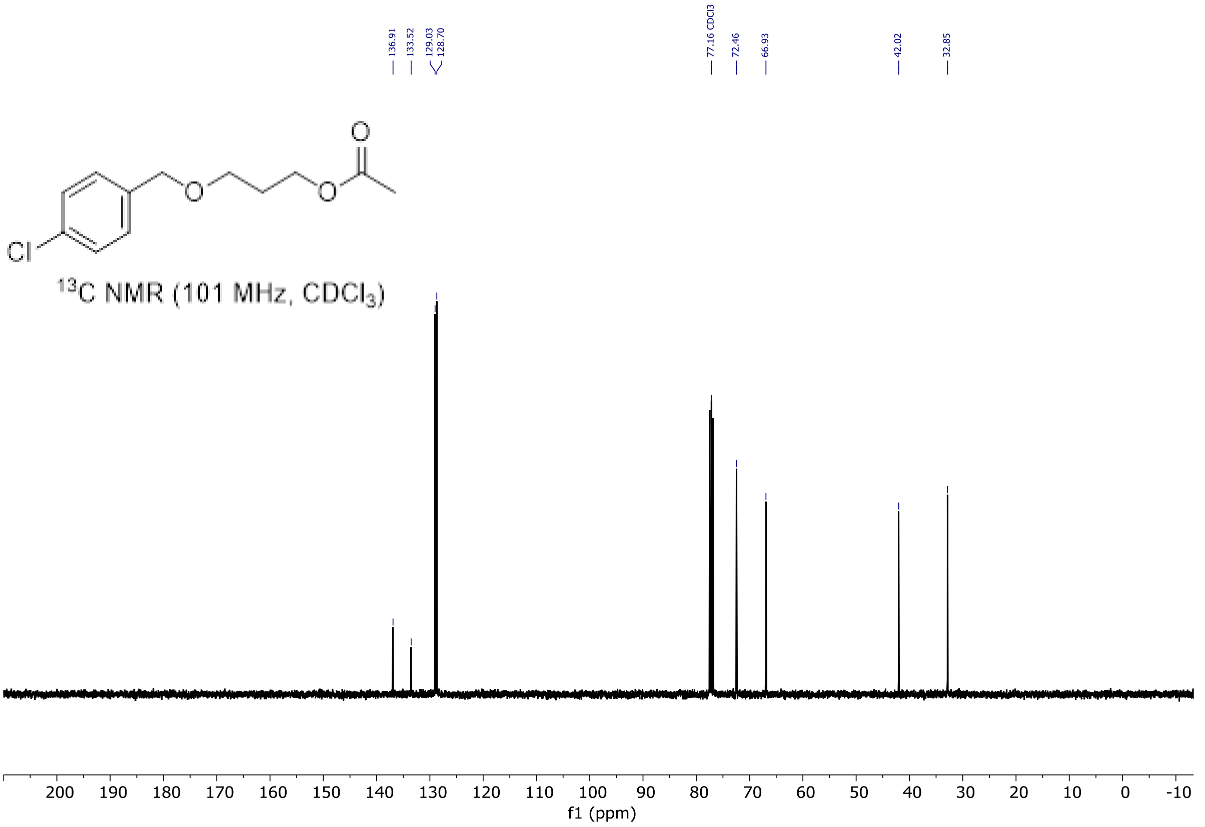

(iv) NMR spectra of (α-Alkoxyalkyl)phosphonium Mesylate **10H** and Ether **13a** Using Methanesulfonic Acid

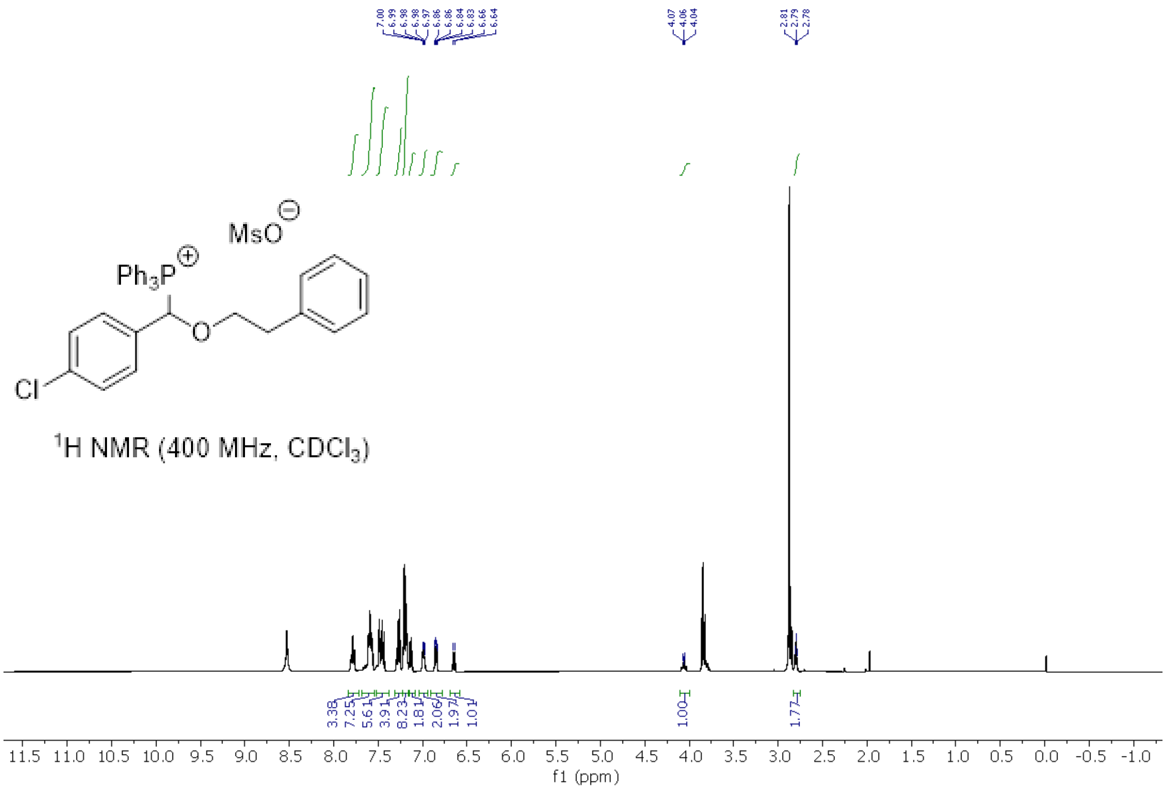

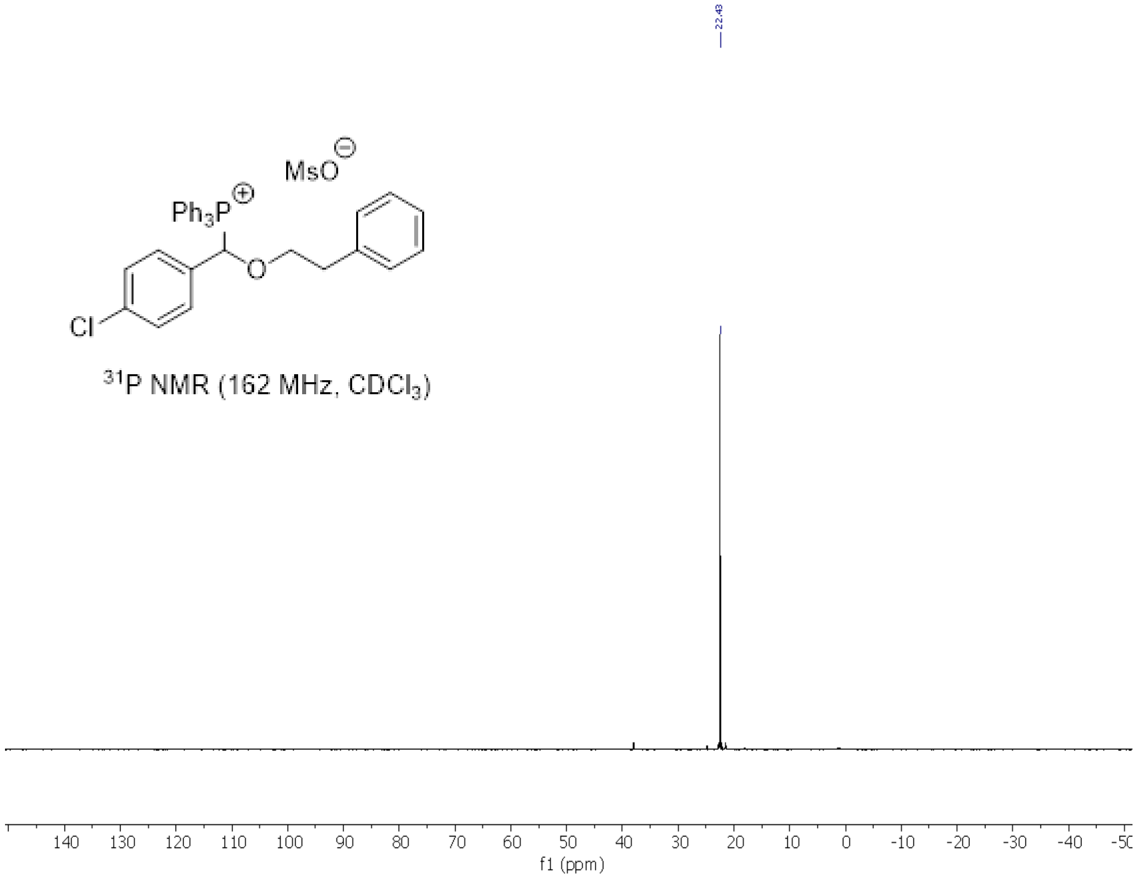

(v) Reaction with pre-made [Ph_3PH] OTf

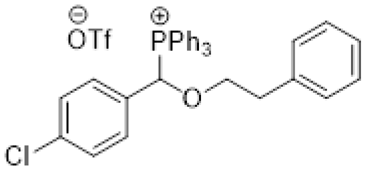

^{31}P NMR (162 MHz, $CDCl_3$)

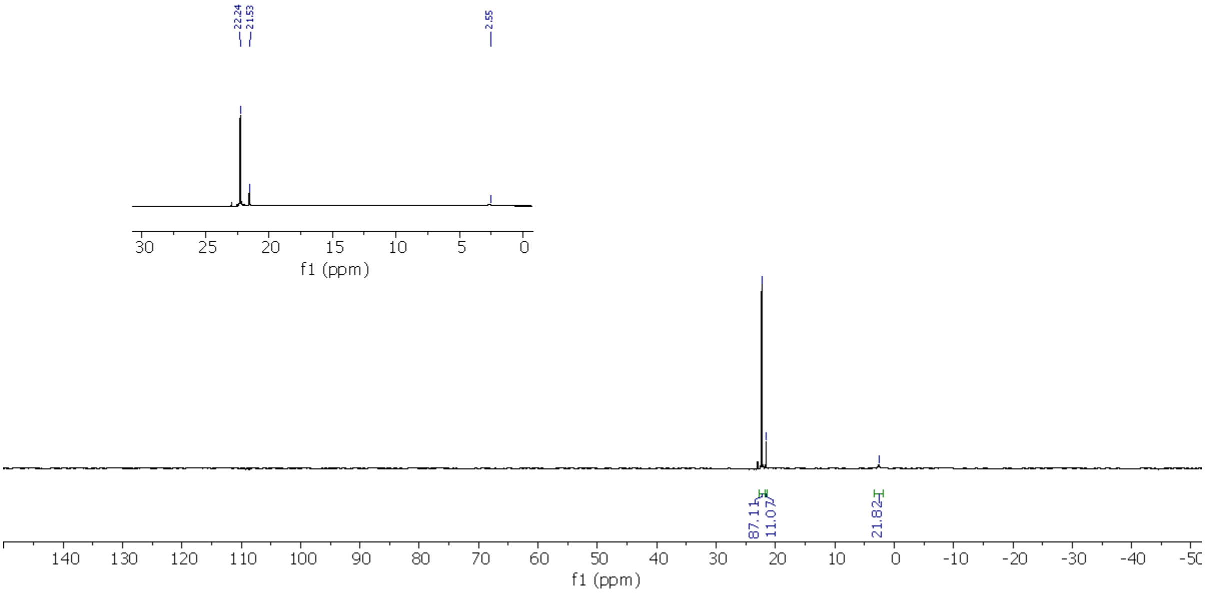

(vi) Incompatible substrates

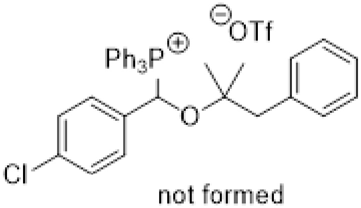

not formed

^{31}P NMR (162 MHz, $CDCl_3$)

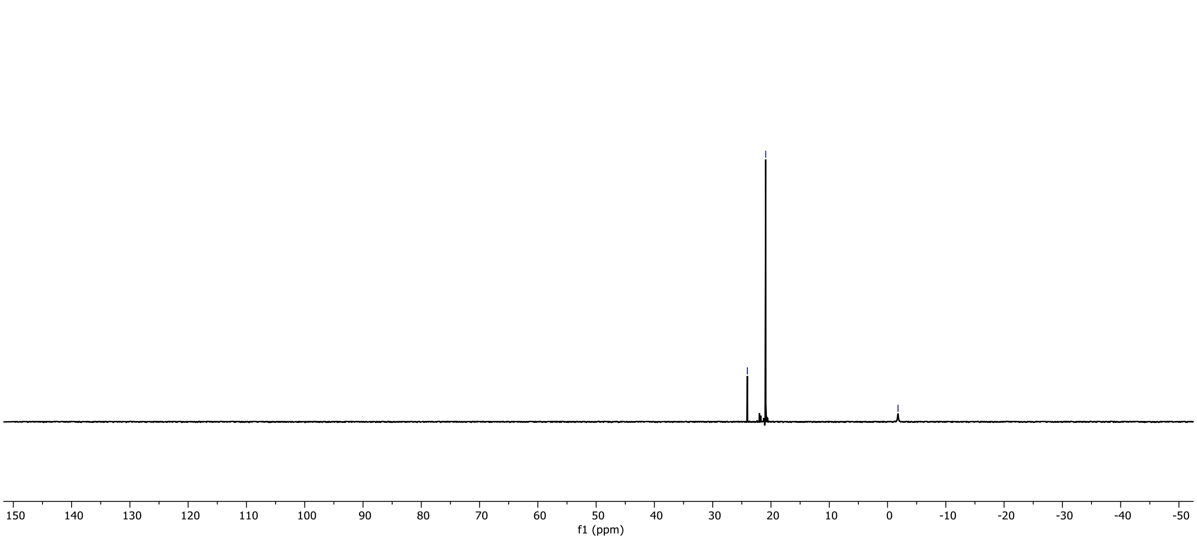

^{31}P NMR (162 MHz, $CDCl_3$) of reaction mixture post hydrolysis:

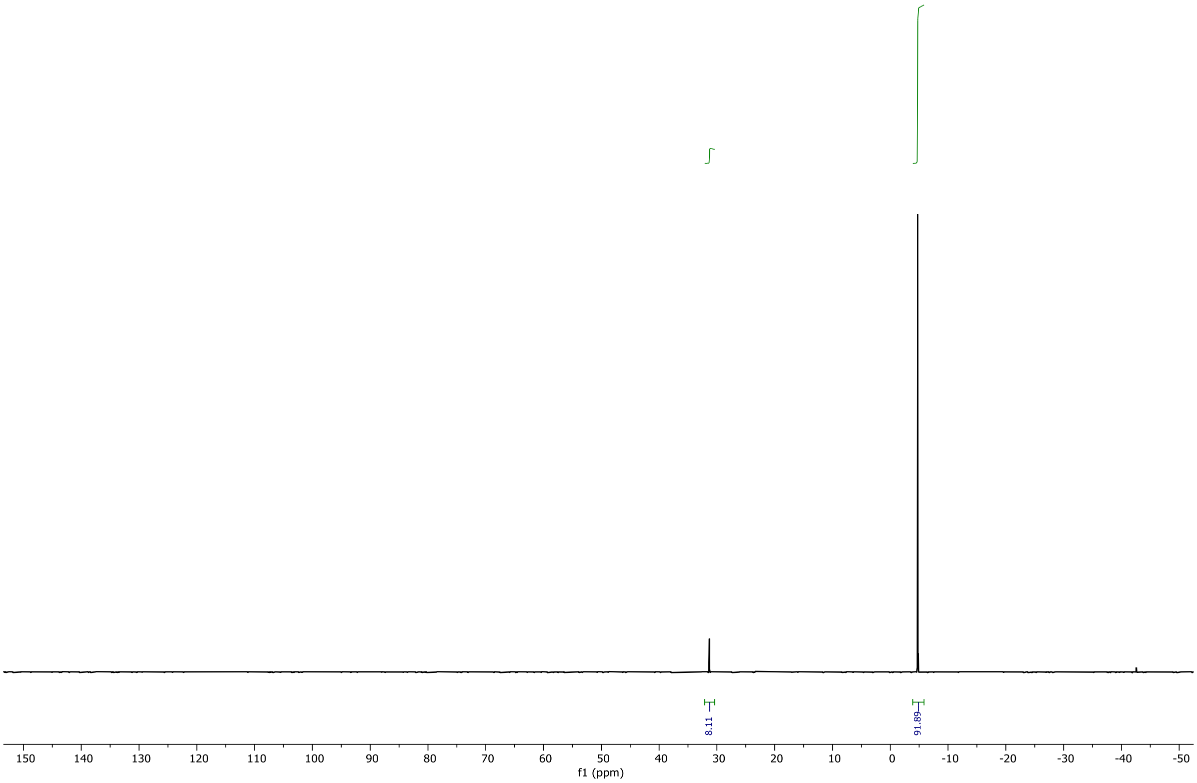

^{31}P NMR spectral analysis of reaction mixture post hydrolysis:

^{31}P NMR (162 MHz, $CDCl_3$)

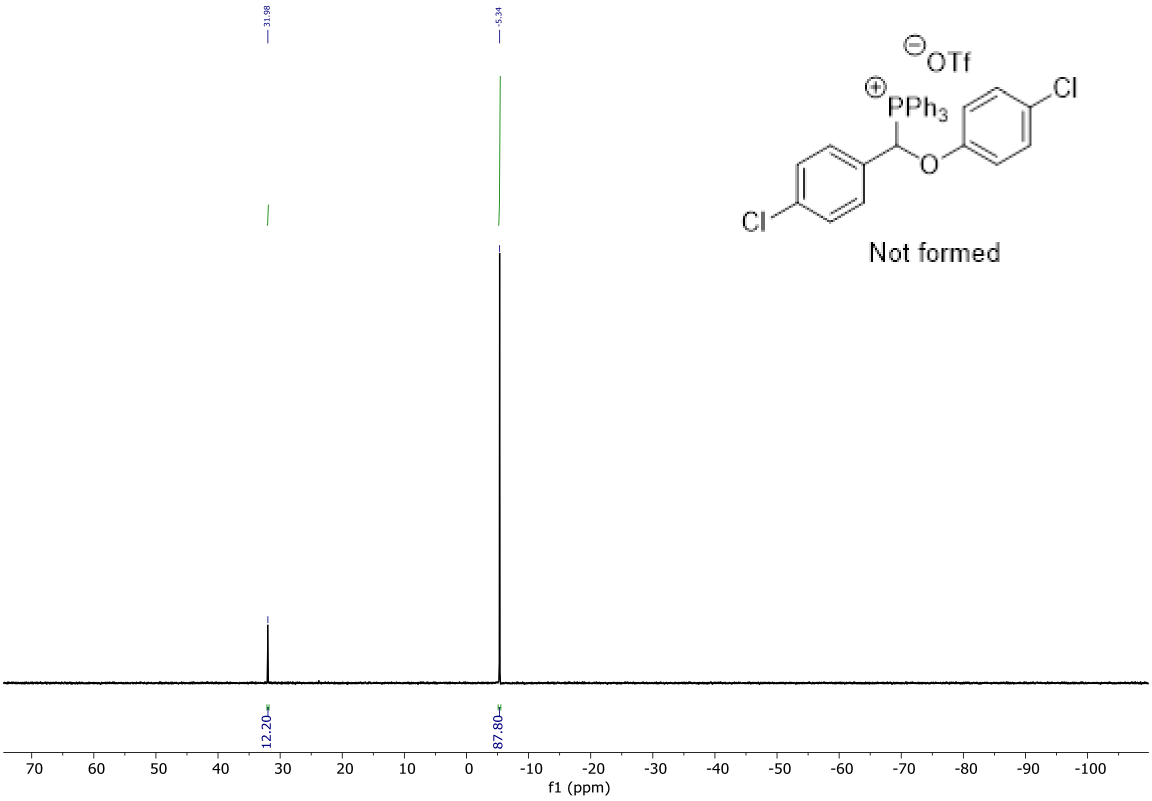

Not formed

(vii) Aldehydes with *para* electron donating substituents

^{31}P NMR spectral analysis of reaction mixture after aldehyde addition

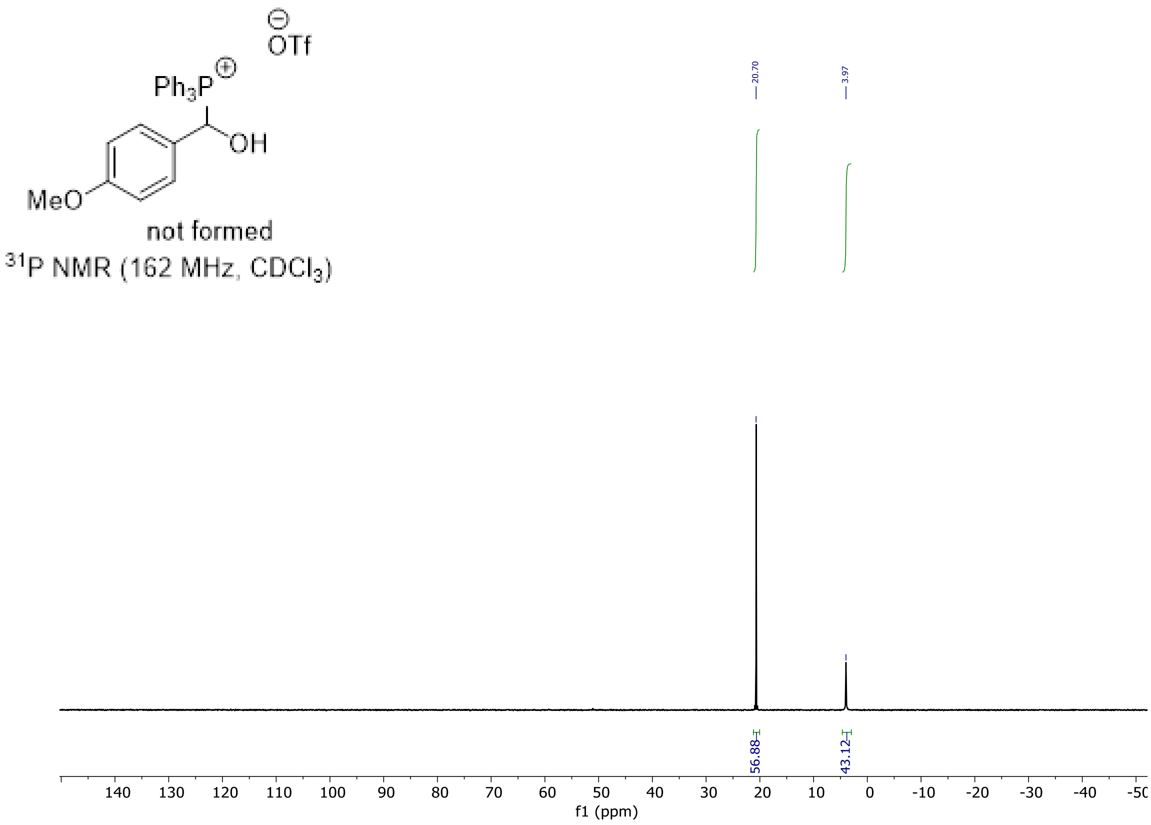

^{31}P NMR spectral analysis of reaction mixture after alcohol addition

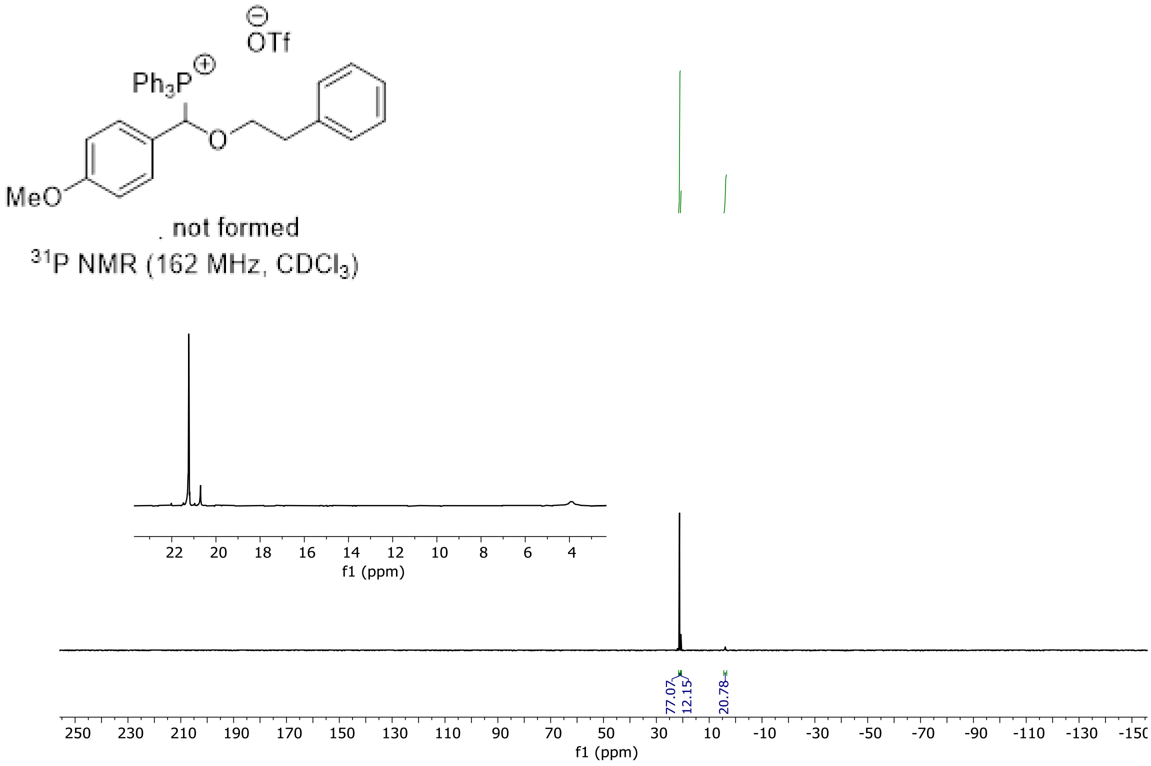

^{31}P NMR spectral analysis of reaction mixture after aldehyde addition

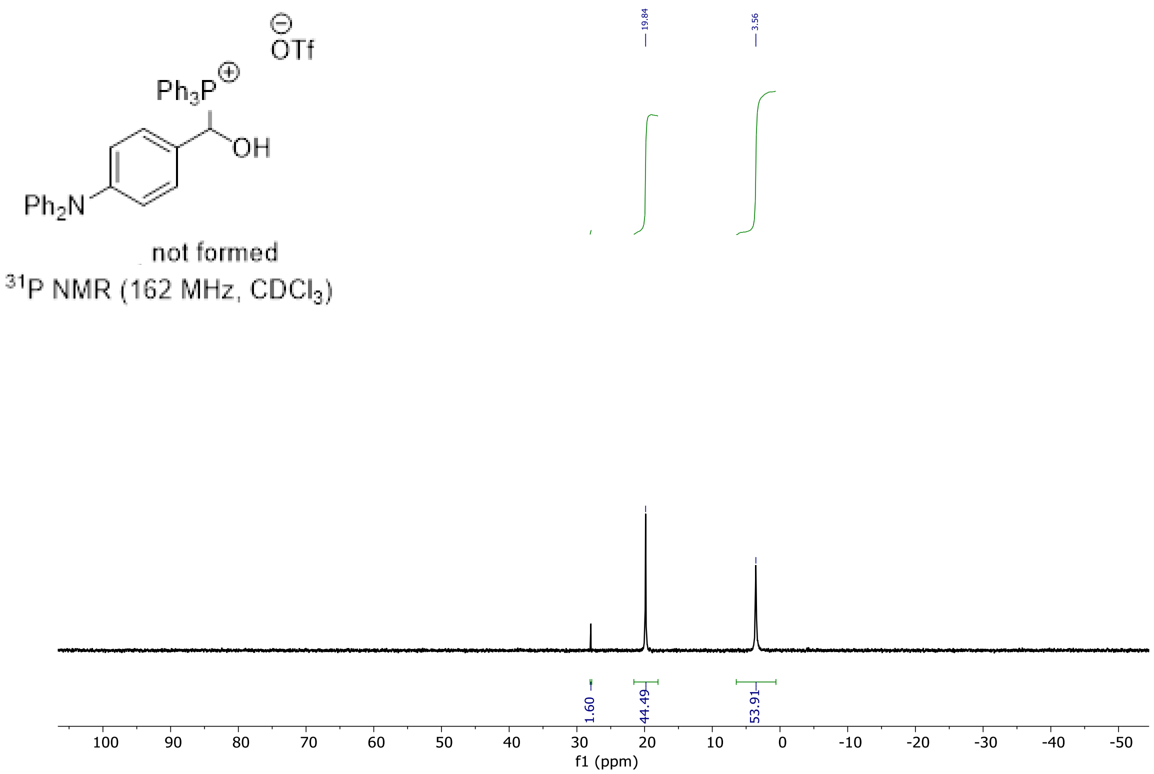

^{31}P NMR spectral analysis of reaction mixture after aldehyde addition

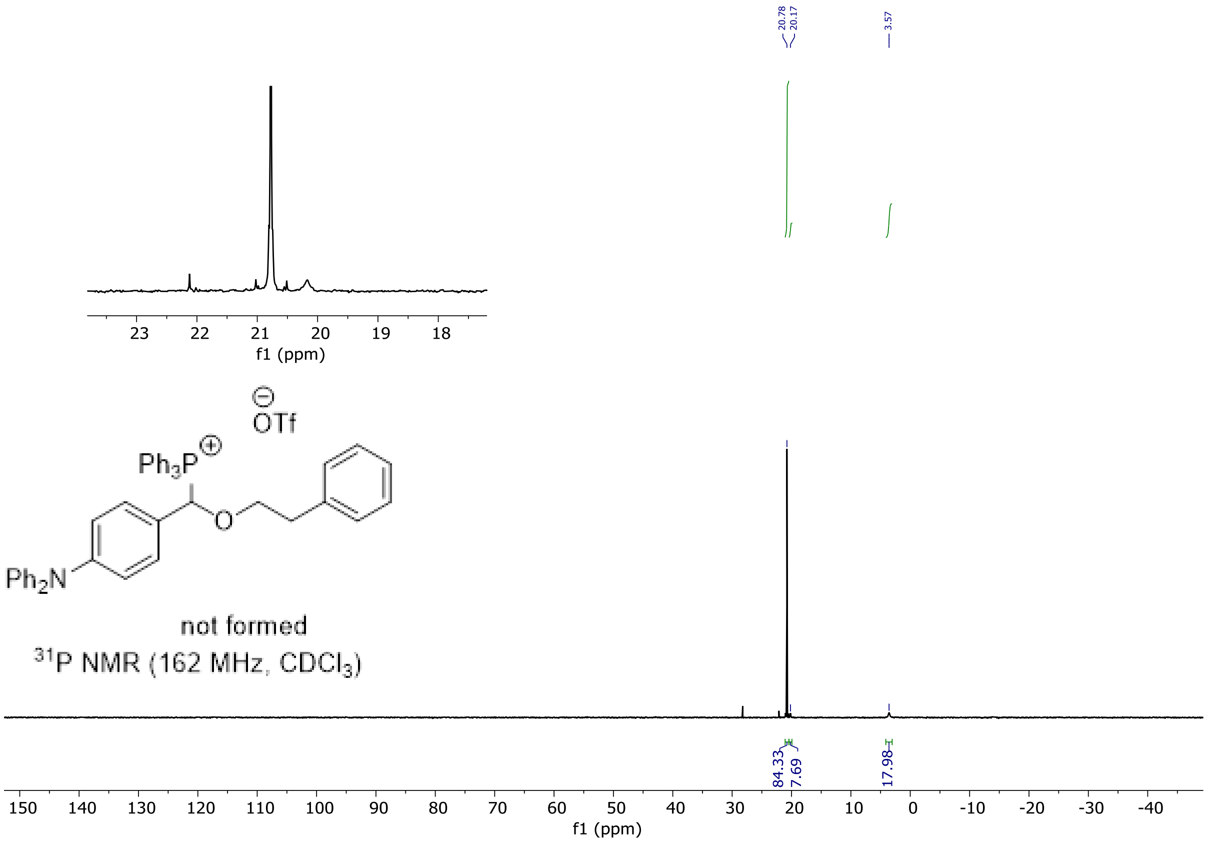

(viii) NMR Spectra for Control Experiments

Experiment 1: Requirement for acid (TfOH) for formation of **10D**

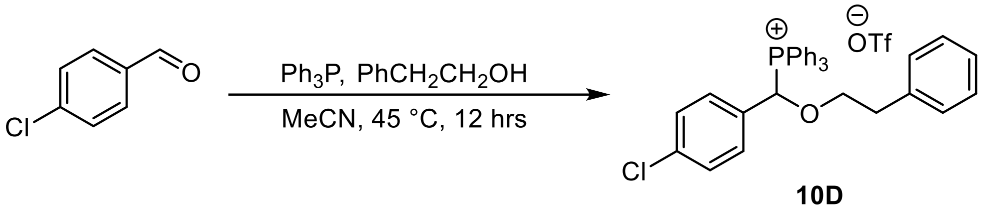

^{31}P NMR (121 MHz, $CDCl_3$)

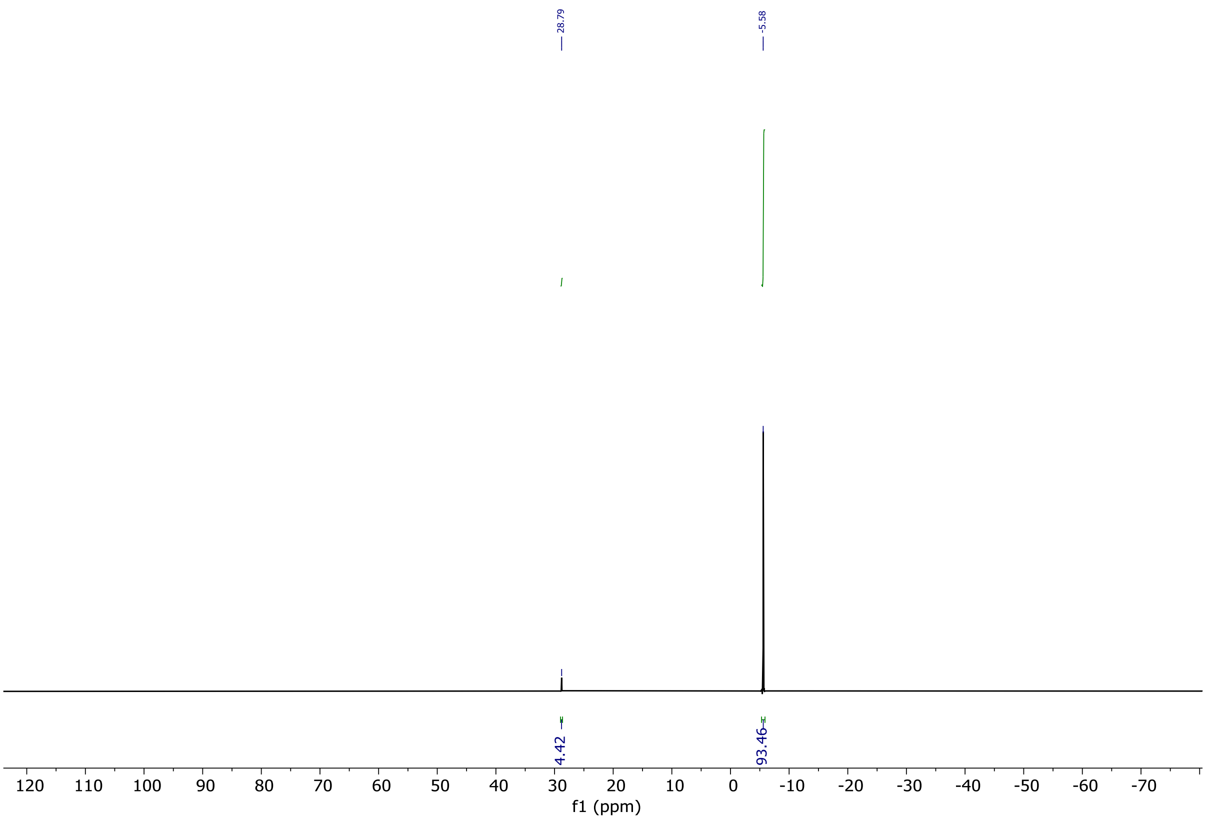

Experiment 2: Reaction of alcohol + 4-chlorobenzaldehyde in the absence of Ph_3P

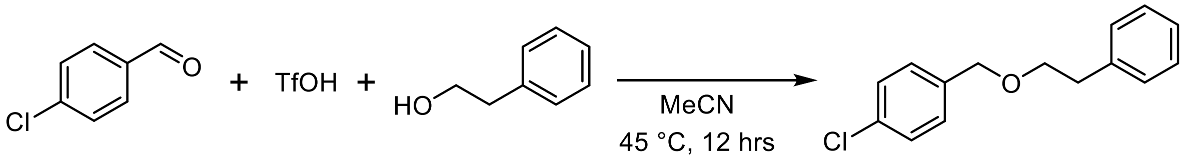

13a - Product not formed

^{1}H NMR (300 MHz $CDCl_3$)

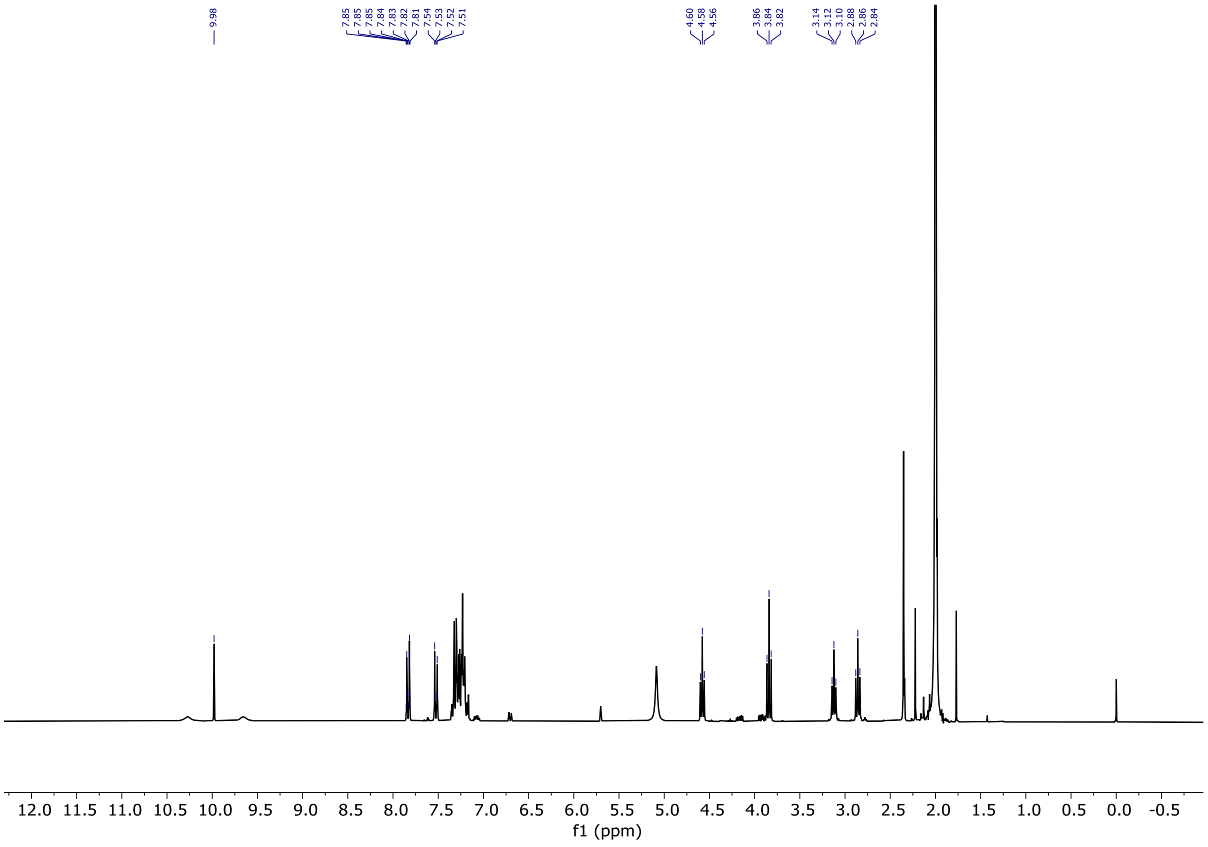

Experiment 3: Interception of oxocarbenium ion **6d** using Ph_3P

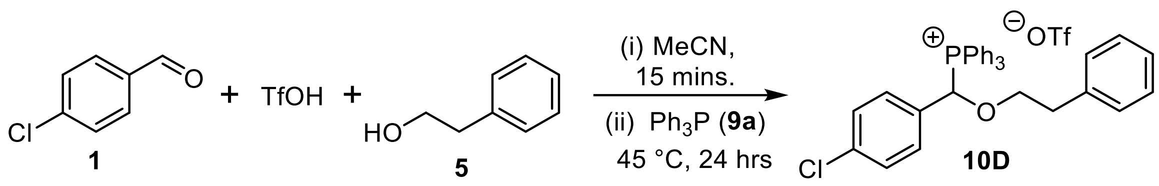

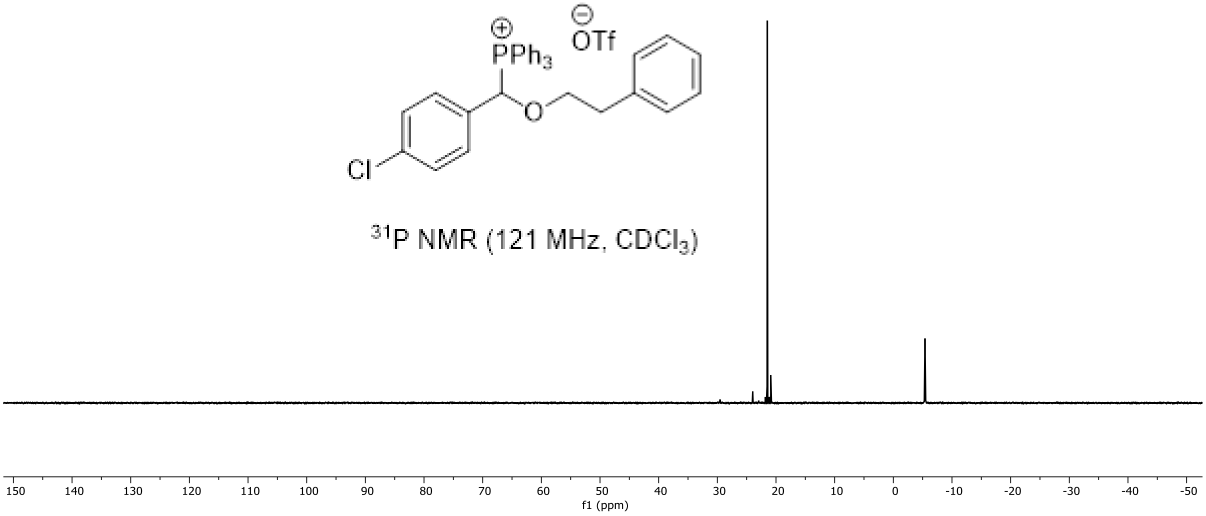

Experiment 4: Importance of Order of Addition for Formation of **10D**

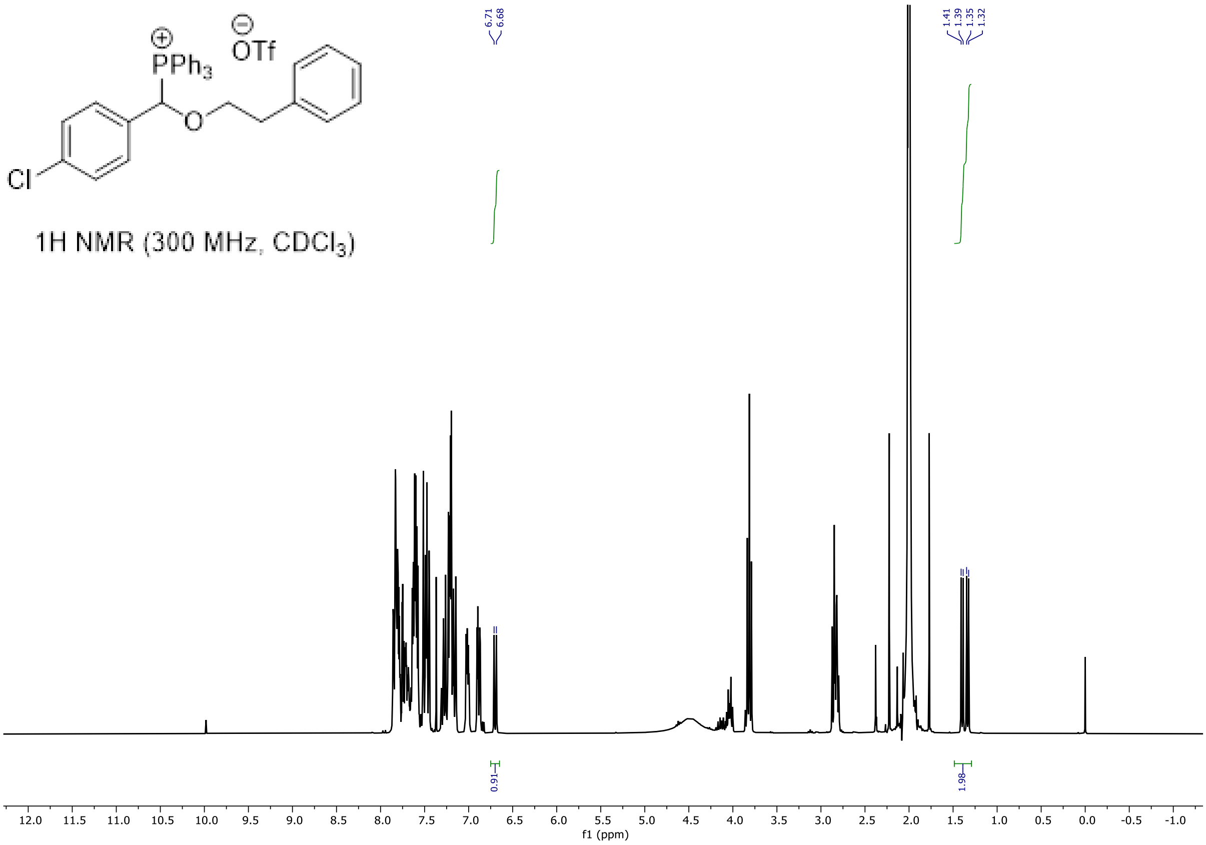

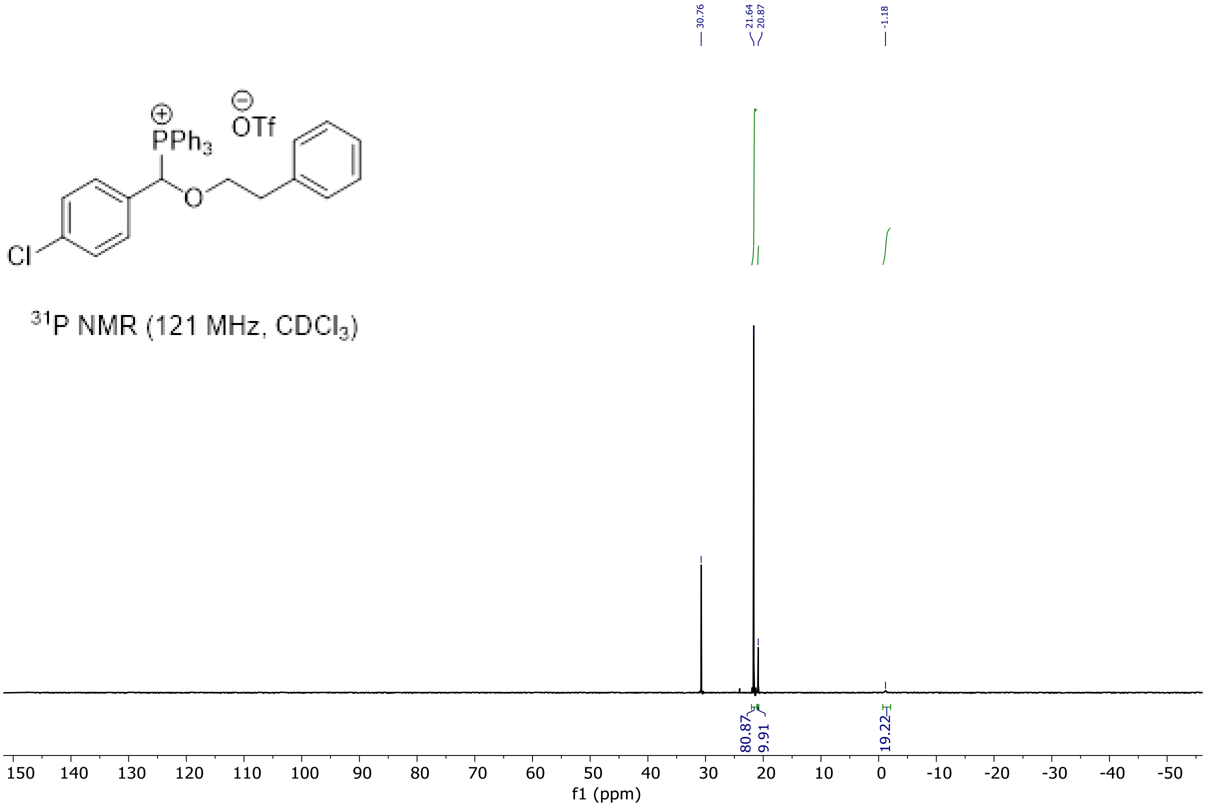

17. NMR Spectra for Mechanistic Investigations

Experiment 1: Formation of (α-hydroxyalkyl)phosphonium salt in the absence of alcohol, followed by alcohol addition to generate (α-alkoxyalkyl)phosphonium salt

^{31}P NMR (121 MHz, $CDCl_3$) of reaction mixture prior to addition of alcohol

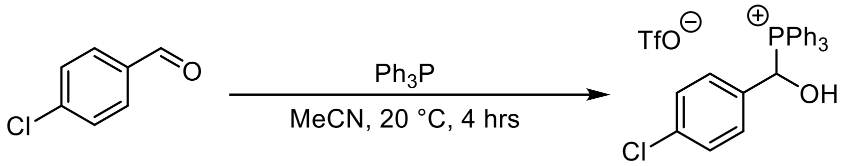

12D

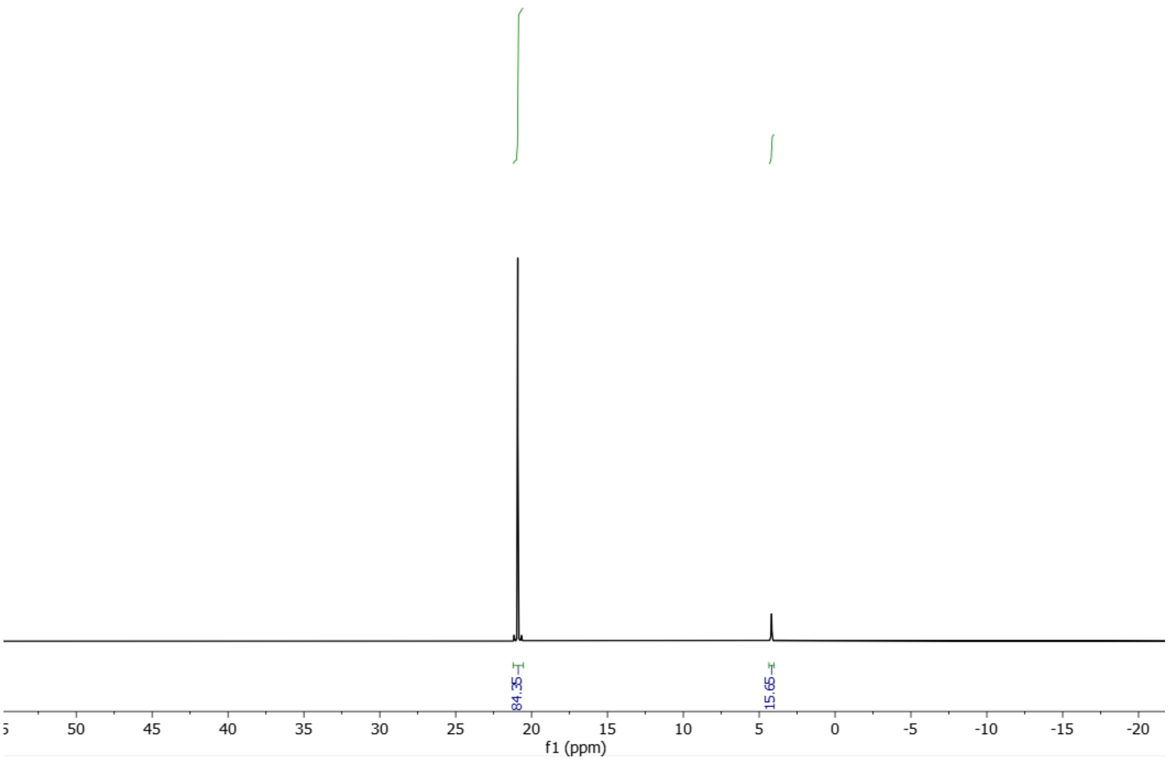

^{31}P NMR (121 MHz, $CDCl_3$) of reaction mixture subsequent to addition of alcohol

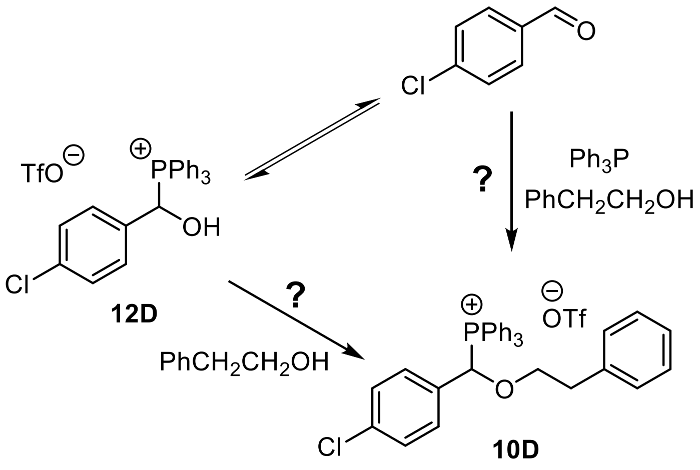

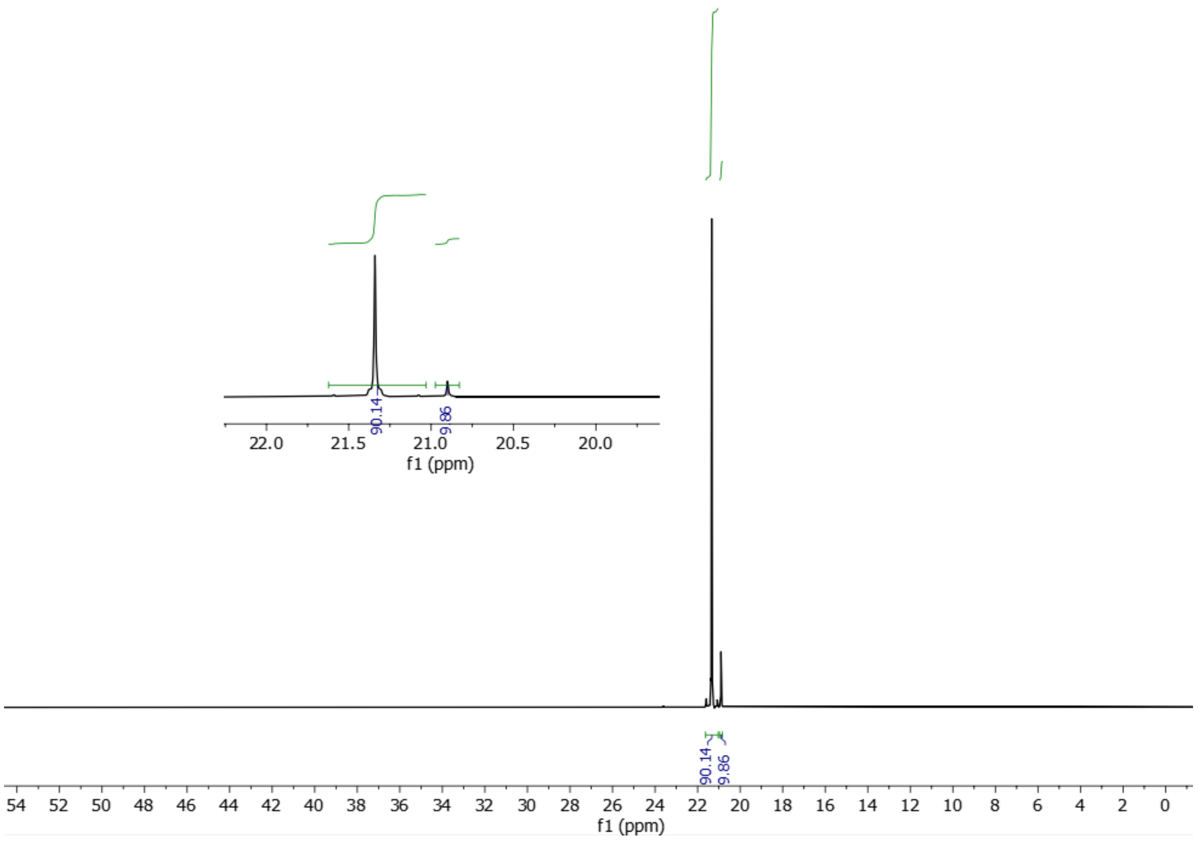

Experiment 2: Determination of the relative favourability of (α-alkoxyalkyl)phosphonium salt and (α-hydroxyalkyl)phosphonium salt formation at an early time-point of a reaction in toluene

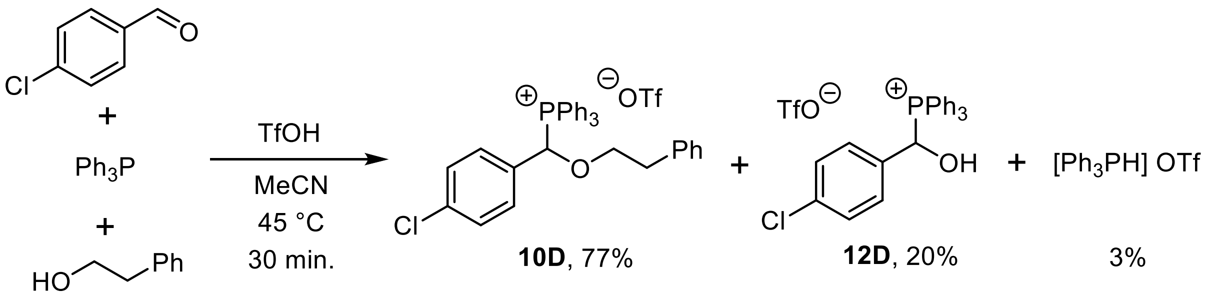

^{31}P NMR (162 MHz, $CDCl_3$)

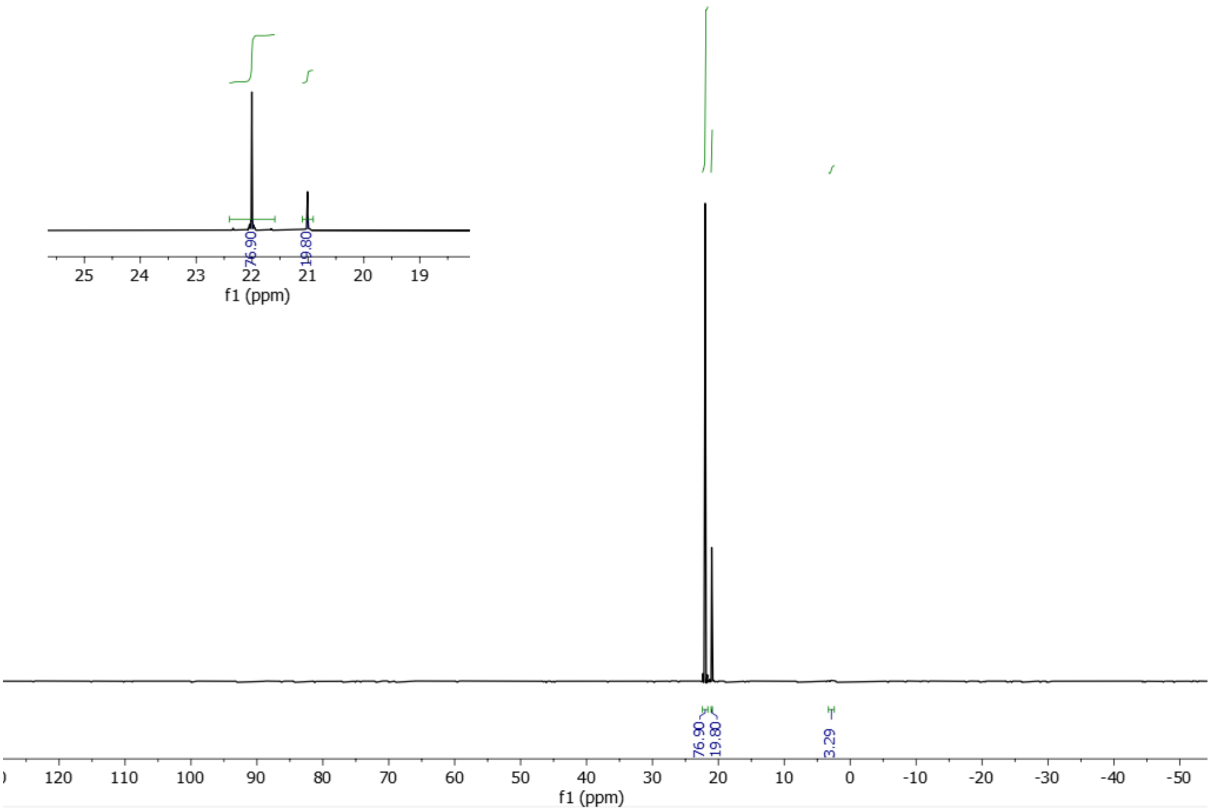

Experiment 3: Determination of the relative favourability of (α-alkoxyalkyl)phosphonium salt and (α-hydroxyalkyl)phosphonium salt formation at an early time-point of a reaction in toluene

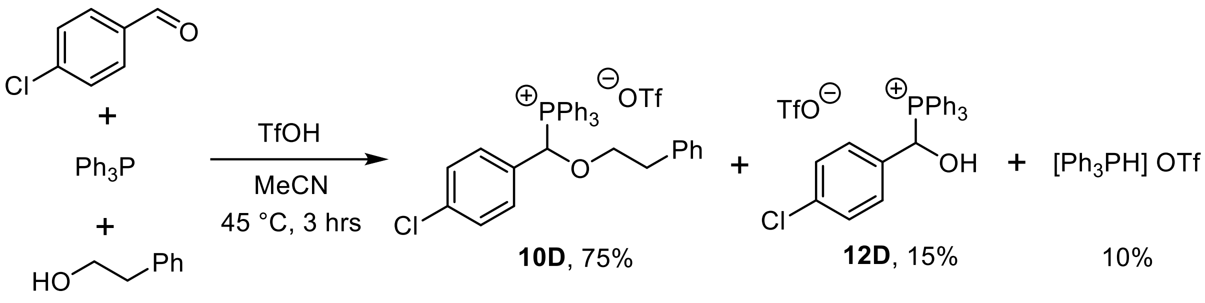

^{31}P NMR (162 MHz, $CDCl_3$)

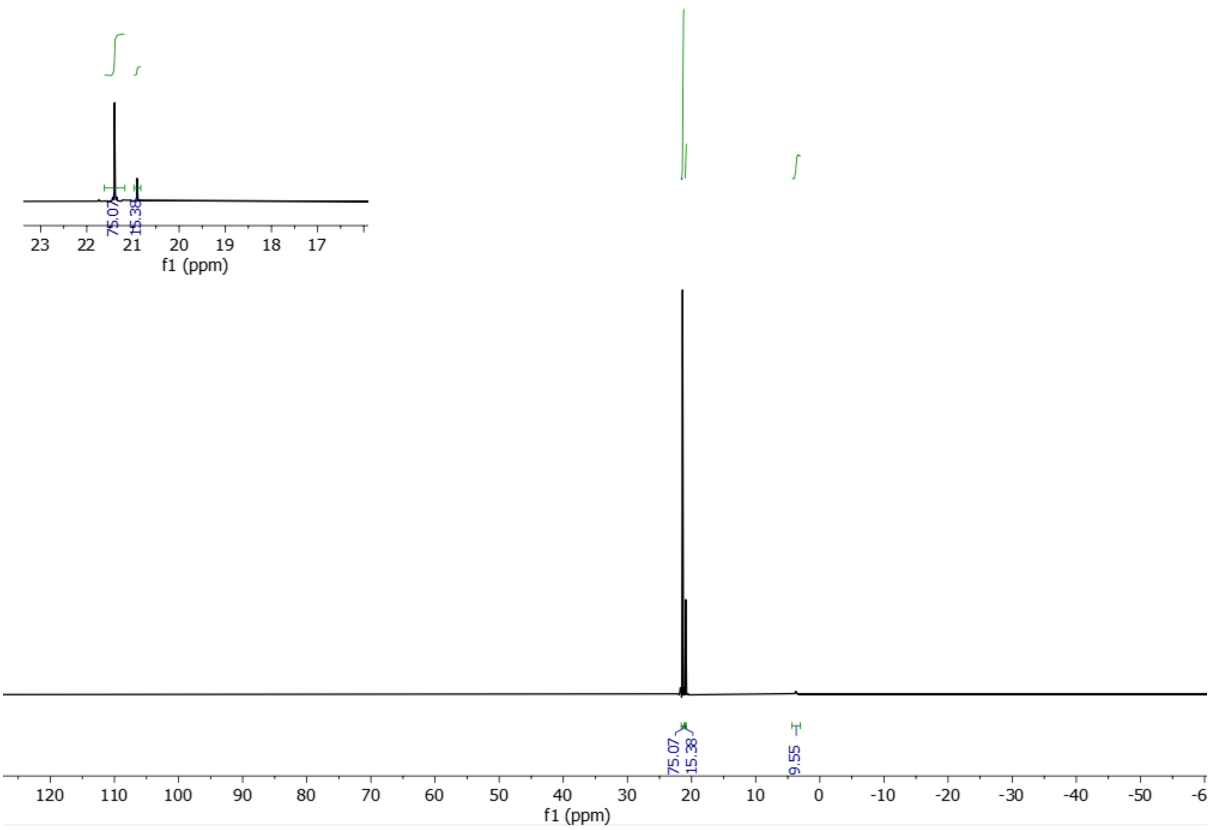

Experiment 4: Formation of **10D** in the presence of a radical trap

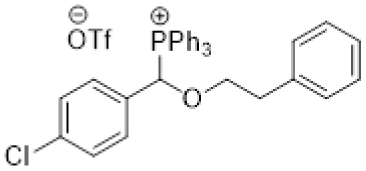

^{31}P NMR (162 MHz, $CDCl_3$)

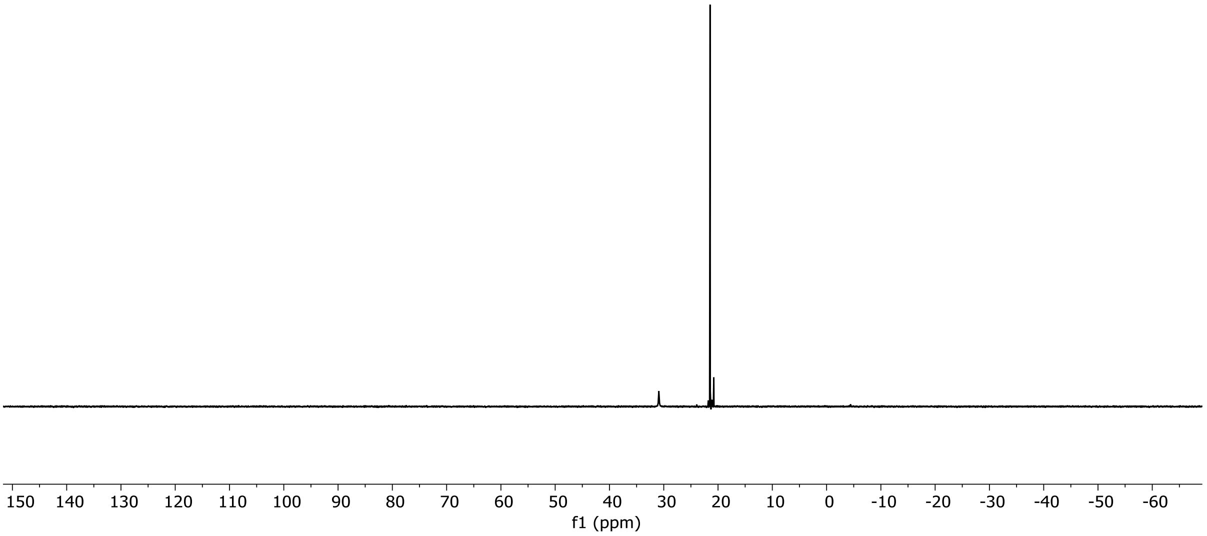

Experiment 5: Independent generation of O-alkyloxocarbenium ion from an acetal, and capture of the O-alkyloxocarbenium ion to generate (α-alkoxyalkyl)phosphonium salt

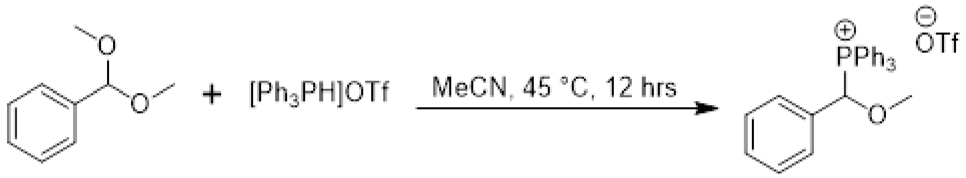

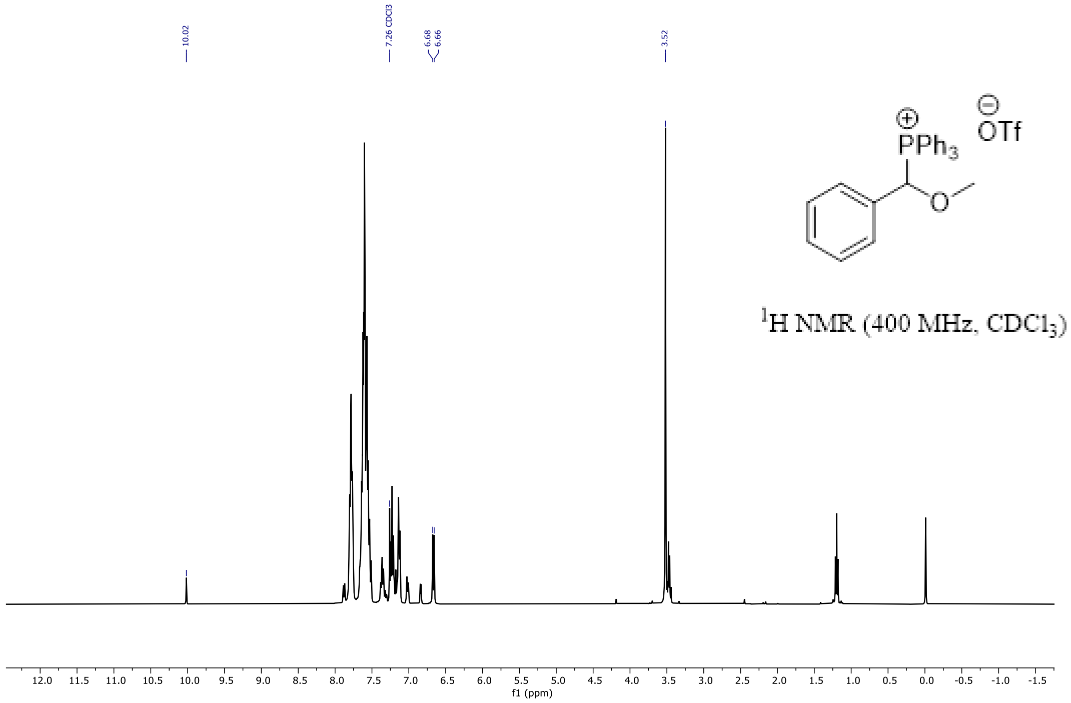

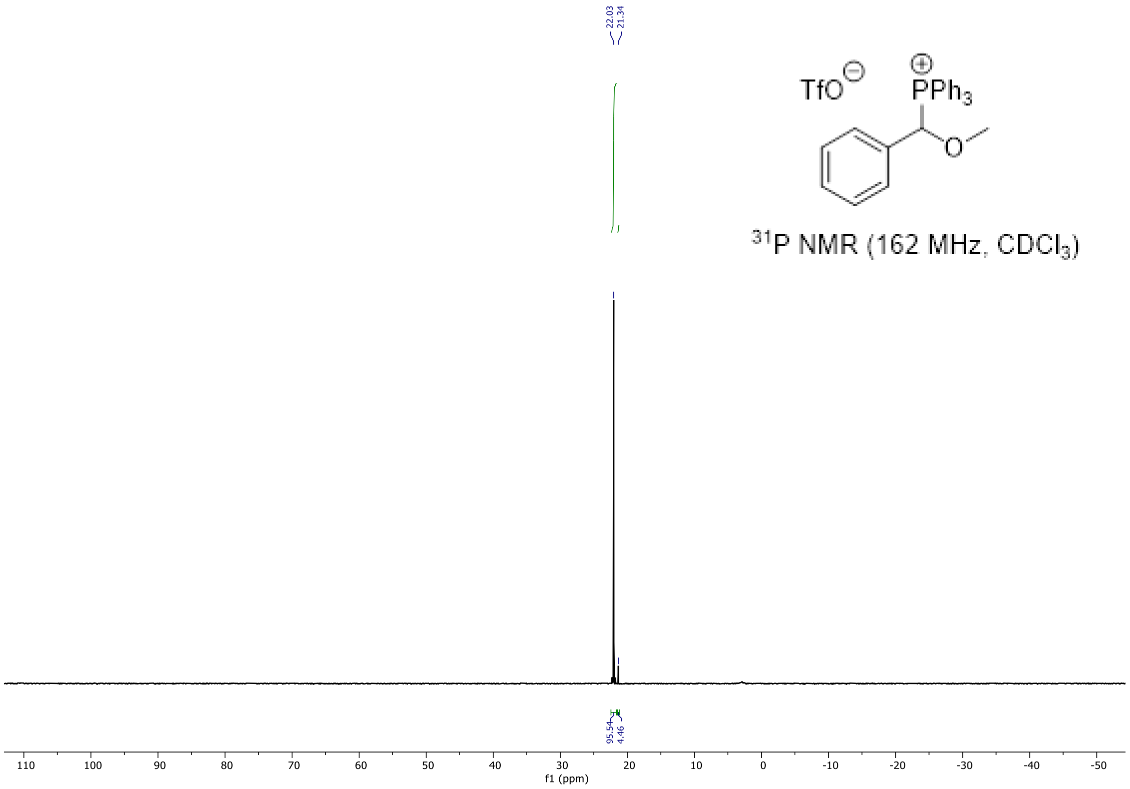

Experiment 5a: Spike experiment involving addition of [$MePh_3P$]OTf, **38-OTf**

^{31}P NMR (162 MHz, $CDCl_3$)

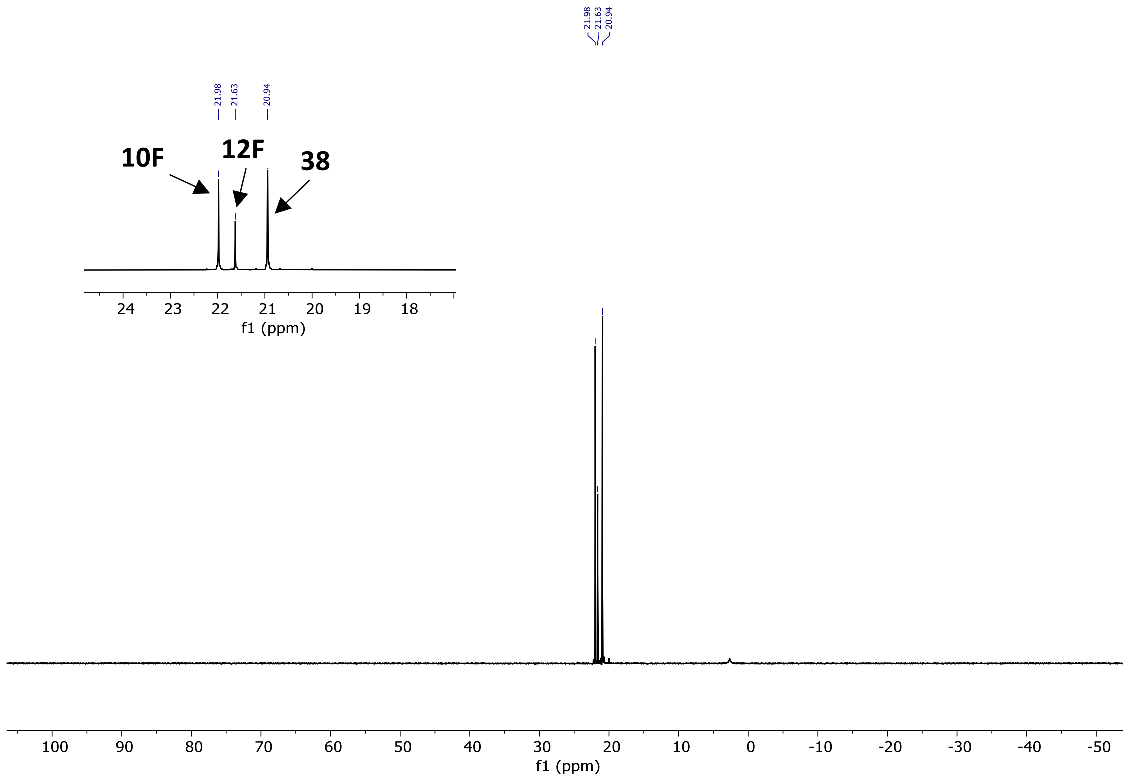

^{1}H NMR (400 MHz, $CDCl_3$)

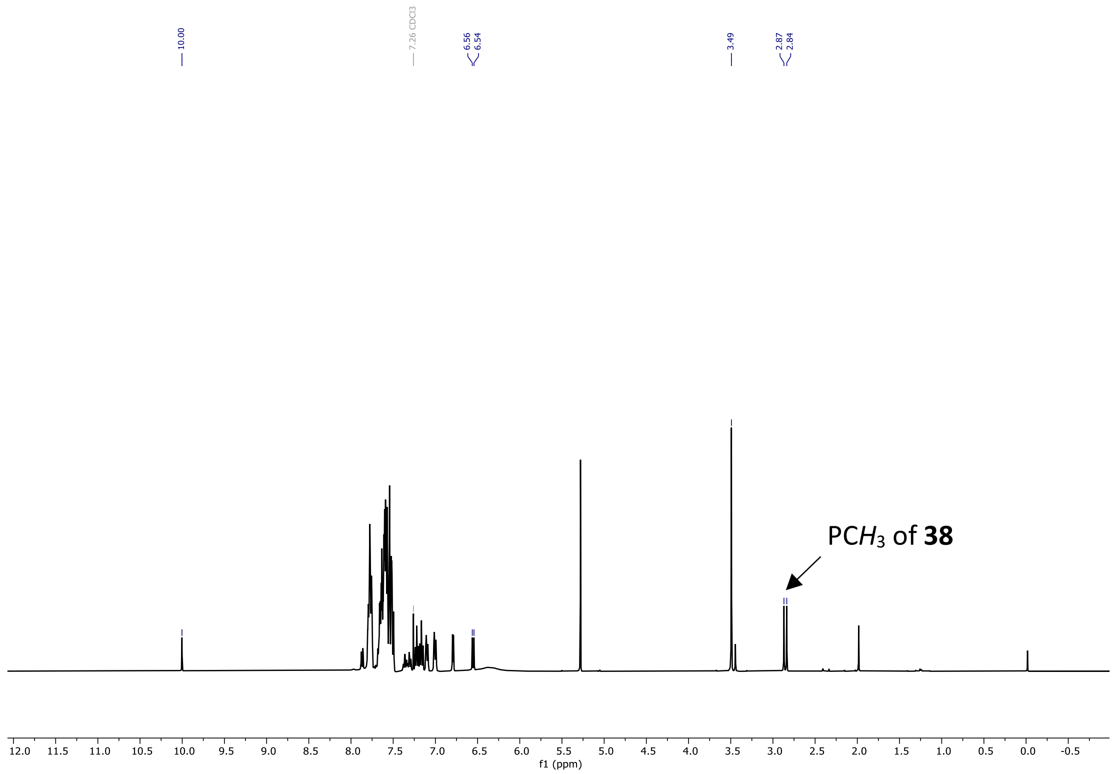

Experiment 5b: Formation of **10F** from PhCHO + MeOH + Ph_3P + TfOH (standard conditions)

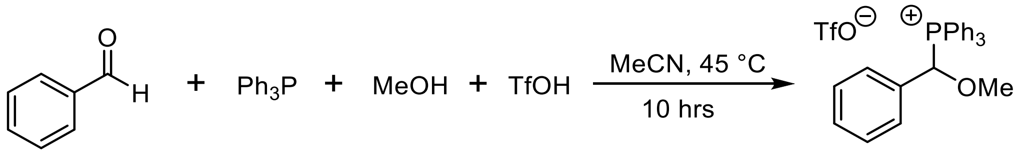

10F

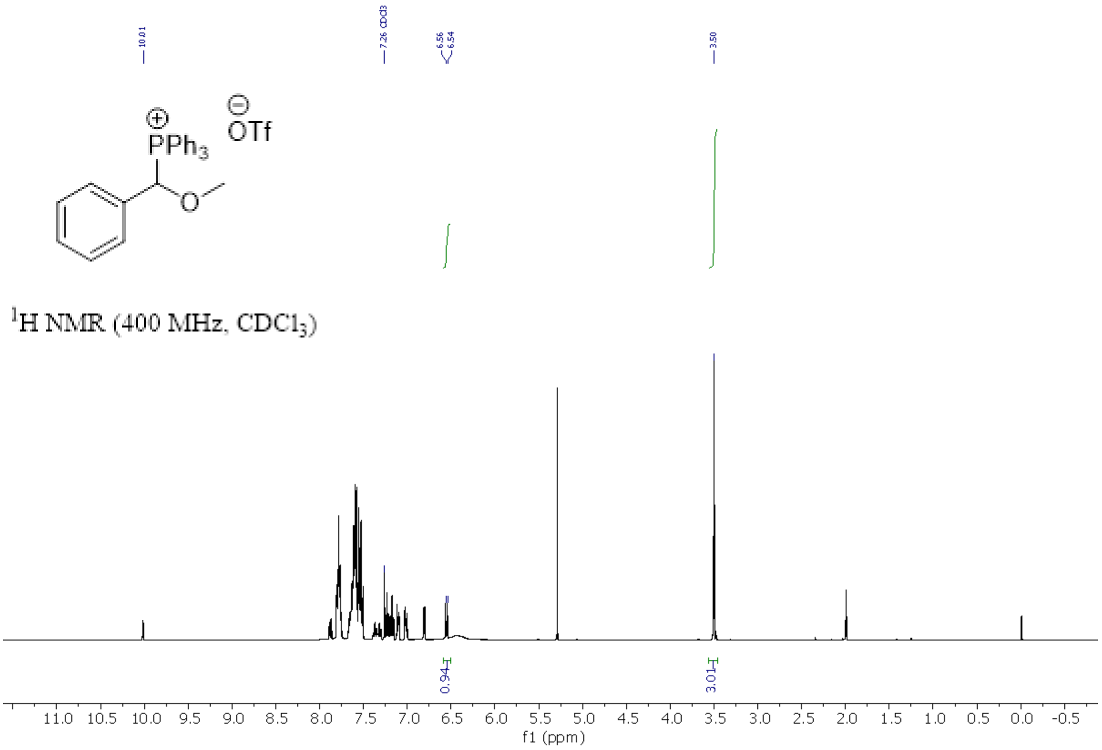

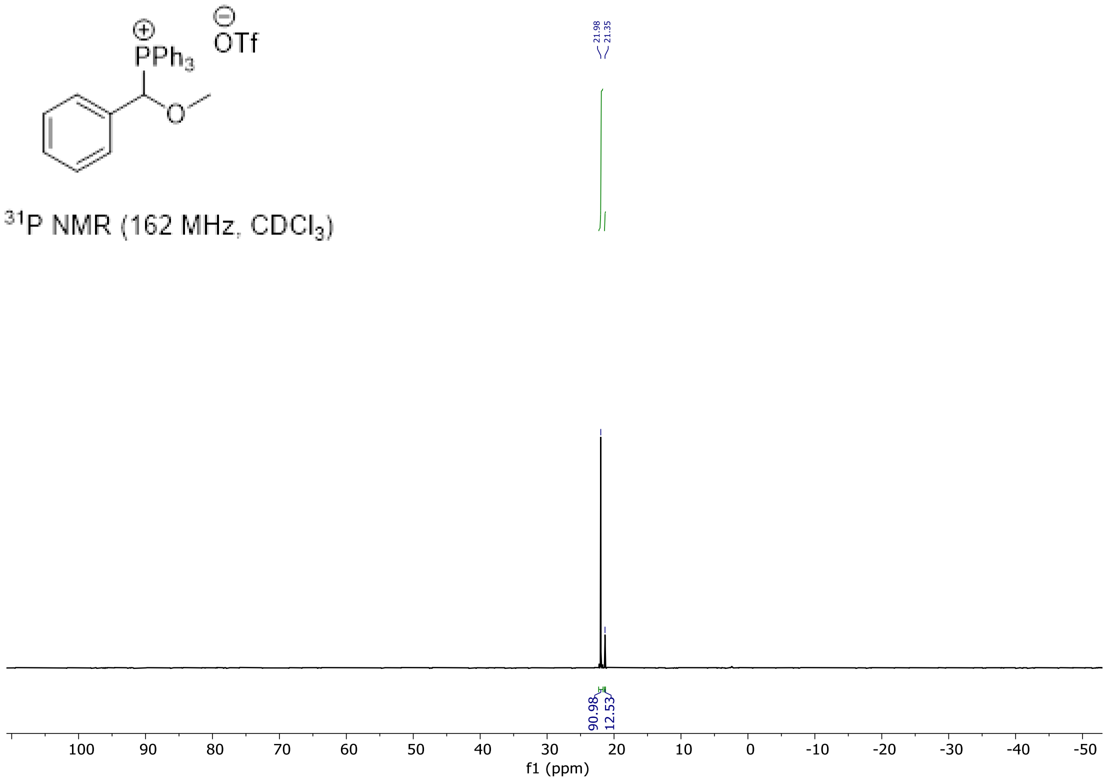

18. References

1 P. R. Hamann, A. Wissner, *Synth. Commun.* **1989**, *19*, 1509–1518.

2 ^{1}H NMR spectral data ($CDCl_3$ solvent) is reported for this compound in: S. A. Nuñez, K. Yeung, N. S. Fox, S. T. Phillips, *J. Org. Chem.* **2011**, *76*, 10099–10113.

3 ^{1}H NMR spectral data ($CDCl_3$ solvent) is reported for this compound in: Y. Nishimoto, A. Okita, A. Baba, M. Yasuda, *Molecules* **2016**, *21*, 1330.

4 ^{1}H NMR spectral data ($CDCl_3$ solvent) is reported for this compound in: Y. Chan, Y. Bai, W. Chen, H. Chen, C. Li, Y. Wu, M. Tseng, G. P. A. Yap, L.; Zhao, H. Chen, *et al. Angew. Chem. Int. Ed*, **2021**, *60*, 19949–19956.

5 ^{1}H NMR spectral data ($CDCl_3$ solvent) is reported for this compound in: S. C. Richter, M. Oestreich, *Eur. J. Org. Chem.* **2021**, *2021*, 2103–2106.

6 ^{1}H, ^{31}P and ^{19}F NMR spectral data ($CDCl_3$ solvent) is reported for this compound in: L. L. Tolstikova, A. V. Bel'skikh, B. A. Shainyan, *Russ. J. Org. Chem.* **2011**, *81*, 474–480.

7 H. C. Fisher, L. Prost, J. L. Montchamp, *Eur. J. Org. Chem.* **2013**, *35*, 7973–7978.

8 ^{31}P NMR spectral data ($CDCl_3$ solvent) is reported for this compound in: G. S. Ananthnag, J. T. Mague, M. S. Balakrishna, *Dalton Trans.* **2015**, *44*, 3785–3793

9 ^{31}P NMR spectral data ($CDCl_3$ solvent) is reported for this compound in: (a) P. A. Byrne, K. V. Rajendran, J. Muldoon, D. G. Gilheany, *Org. Biomol. Chem.* **2012**, *10*, 3531–3537; (b) T. Krachko, V. Lyaskovskyy, M. Lutz, K. Lammertsma, J. C. Slootweg, *Z. Anorg. Allg. Chem.* **2017**, *643*, 916–921.

10 M. M. Boucher, N. H. Furigay, P. K. Quach, C. S. Brindle, *Org. Process Res. Dev.* **2017**, *21*, 1394–1403.

11 ^{1}H NMR spectral data ($CDCl_3$ solvent) is reported for this compound in: T. Shintou, T. Mukaiyama, *J. Am. Chem. Soc.* **2004**, *126*, 7359–7367.

12 ^{1}H NMR spectral data ($CDCl_3$ solvent) is reported for this compound in: T. Yamada, S. Tsukagoshi, O. Kitagawa, *Tetrahedron Lett.* **2017**, *58*, 317–320.

13 ^{1}H NMR spectral data ($CDCl_3$ solvent) is reported for this compound in: G. Li, D. Leow, L. Wan, J.-Q. Yu, *Angew. Chem. Int. Ed.* **2013**, *52*, 1245–1247.

14 ^{1}H NMR spectral data ($CDCl_3$ solvent) is reported for this compound in: T. J. A. Corrie, L. T. Ball, C. A. Russell, G. C. Lloyd-Jones, *J. Am. Chem. Soc.* **2017**, *139*, 245–254.

15 ^{1}H NMR spectral data ($CDCl_3$ solvent) is reported for this compound in: L. Liu, Y. Tang, K. Wang, T. Huang, T. Chen, *J. Org. Chem.* **2021**, *86*, 4159–4170.

16 ^{1}H NMR spectral data ($CDCl_3$ solvent) is reported for this compound in: A. Pelosi, D. Lanari, A. Temperini, M. Curini, O. Rosati, *Adv. Synth. Catal.* **2019**, *361*, 4527–4539.

17 ^{1}H NMR spectral data ($CDCl_3$ solvent) is reported for this compound in: K. A. C. Bastick, A. J. B. Watson, *Synlett* **2023**, *34*, 2097–2102.

18 ^{1}H NMR spectral data ($CDCl_3$ solvent) is reported for this compound in: E. E. Kingston, J. S. Shannon, V. Diakiw, M. J. Lacey, *Org. Mass Spectrom.* **1981**, *16*, 428–440.

19 ^{1}H NMR spectral data ($CDCl_3$ solvent) is reported for this compound in: I. Karakaya, D. N. Primer, G. A. Molander, *Org. Lett.* **2015**, *17*, 3294–3297.

20 ^{1}H NMR spectral data ($CDCl_3$ solvent) is reported for this compound in: C. Zhao, C. A. Sojdak, W. Myint, D. Seidel, *J. Am. Chem. Soc*. **2017**, *139*, 10224–10227.

21 ^{1}H NMR spectral data ($CDCl_3$ solvent) is reported for this compound in: N.-N. Li, Y.-L. Zhang, S. Mao, Y.-R. Gao, D.-D. Guo, Y.-Q. Wang, *Org. Lett.* **2014**, *16*, 2732–2735.

22 ^{1}H NMR spectral data ($CDCl_3$ solvent) is reported for this compound in: F. G. Delolo, J. Fessler, H. Neumann, K. Junge, E. N. dos Santos, E. V. Gusevskaya, M. Beller *Chem. Eur. J.* **2022**, *28*, e202103903.

23 ^{1}H NMR spectral data ($CDCl_3$ solvent) is reported for this compound in: L. da Silva Santos, F. L. M. de Carvalho, C. A. de Souza Pinto, L. A. da Fonseca, C. J. Dias Lopes, F. de Pilla Varotti, P. R. de Freitas, B. R. Alves, *Med. Chem.* **2021**, *17*, 820–833.

24 ^{1}H NMR spectral data ($CDCl_3$ solvent) is reported for this compound in: C. Russo, M. C. Leech, J. M. Walsh, J. I. Higham, L. Giannessi, E. Lambert, C. Kiaku, D. L. Poole, J. Mason, C. A. I. Goodall, P. Devo, M. Giustiniano, M. Radi, K. Lam, *Angew. Chem. Int. Ed.* **2023**, *62*, e202309563.

25 ^{1}H NMR spectral data ($CDCl_3$ solvent) is reported for this compound in: P. Patel and S. A. L. Rousseaux, *Synlett* **2020**, *31*, 492–496.

26 ^{1}H NMR spectral data ($CDCl_3$ solvent) is reported for this compound in: J. Fang, T. Li, X. Ma, J. Sun, L. Cai, Q. Chen, Z. Liao, L. Meng, J. Zeng, Q. Wan, *Chin. Chem. Lett*. **2022**, *33*, 288–292.

27 ^{1}H NMR spectral data ($CDCl_3$ solvent) is reported for this compound in: C.-V. T. Vo, T. A. Mitchell and J. W. Bode, *J. Am. Chem. Soc.* **2011**, *133*, 14082–14089

28 For reviews on the effects of deuterium incorporation on chemical shifts and signal intensity of ^{1}H and ^{13}C NMR spectral signals see: (a) M. Sattler, S. W. Fesik, *Structure* **1996**, *4*, 1245–1249; (b) P. E. Hansen, *Annu. Rep. NMR Spectrosc*. **1984**, *15*, 105–234.

29 ^{1}H NMR spectral data ($CDCl_3$ solvent) is reported for this compound in: L. Wang, Y. Hashidoko, M. Hashimoto, *J. Org. Chem.* **2016**, *81*, 4464–4474.

30 ^{1}H NMR spectral data ($CDCl_3$ solvent) is reported for this compound in: K. Iwanami, K. Yano, T. Oriyama, *Synthesis* **2005**, *16*, 2669–2672

31 ^{1}H NMR spectral data ($CDCl_3$ solvent) is reported for this compound in: E. E. Kingston, J. S. Shannon, V. Diakiw, M. J. Lacey, *J. Mass Spectrom.***1981**, *16*, 428–440.

32 ^{1}H NMR spectral data ($CDCl_3$ solvent) is reported for this compound in: C. Zhao, C. A. Sojdak, W. Myint, D. Seidel, *J. Am. Chem. Soc* **2017**, *139*, 10224-10227

33 ^{1}H NMR spectral data ($CDCl_3$ solvent) is reported for this compound in: C.-V. T. Vo, T. A. Mitchell, J. W. Bode, *J. Am. Chem. Soc.* **2011**, *133*, 14082–14089.

34 ^{1}H and ^{31}P NMR spectral data ($CDCl_3$ solvent) is reported for this compound in: C. Xu, T. Li, P. Jiang, Y. J. Zhang, *Tetrahedron* **2020**, *76*, 131107.

35 M. J. Frisch, G. W. Trucks, H. B. Schlegel, G. E. Scuseria, M. A. Robb, J. R. Cheeseman, G. Scalmani, V. Barone, G. A. Petersson, H. Nakatsuji, X. Li, M. Caricato, A. V. Marenich, J. Bloino, B. G. Janesko, R. Gomperts, B. Mennucci, H. P. Hratchian, J. V. Ortiz, A. F. Izmaylov, J. L. Sonnenberg, Williams, F. Ding, F. Lipparini, F. Egidi, J. Goings, B. Peng, A. Petrone, T. Henderson, D. Ranasinghe, V. G. Zakrzewski, J. Gao, N. Rega, G. Zheng, W. Liang, M. Hada, M. Ehara, K. Toyota, R. Fukuda, J. Hasegawa, M. Ishida, T. Nakajima, Y. Honda, O. Kitao, H. Nakai, T. Vreven, K. Throssell, J. A. Montgomery Jr., J. E. Peralta, F. Ogliaro, M. J. Bearpark, J. J. Heyd, E. N. Brothers, K. N. Kudin, V. N. Staroverov, T. A. Keith, R. Kobayashi, J. Normand, K.

Raghavachari, A. P. Rendell, J. C. Burant, S. S. Iyengar, J. Tomasi, M. Cossi, J. M. Millam, M. Klene, C. Adamo, R. Cammi, J. W. Ochterski, R. L. Martin, K. Morokuma, O. Farkas, J. B. Foresman and D. J. Fox *Gaussian 16 Rev. B.01*, Wallingford, CT, **2016**.

36 J. D. Chai, M. Head-Gordon, *Phys. Chem. Chem. Phys.* **2008**, *10*, 6615–6620.

37 F. Weigend, R. Ahlrichs, *Phys. Chem. Chem. Phys.* **2005**, *7*, 3297–3305.
